# Connectivity between countries established by landbirds and raptors migrating along the African–Eurasian flyway

**DOI:** 10.1111/cobi.14002

**Published:** 2022-12-15

**Authors:** João L. Guilherme, Victoria R. Jones, Inês Catry, Martin Beal, Maria P. Dias, Steffen Oppel, Juliet A. Vickery, Chris M. Hewson, Stuart H. M. Butchart, Ana S. L. Rodrigues

**Affiliations:** ^1^ CEFE, Univ Montpellier, CNRS, EPHE, IRD Montpellier France; ^2^ BirdLife International Cambridge UK; ^3^ CIBIO/InBIO, Centro de Investigação em Biodiversidade e Recursos Genéticos, Laboratório Associado Universidade do Porto Vairão Portugal; ^4^ CIBIO/InBIO, Centro de Investigação em Biodiversidade e Recursos Genéticos, Instituto Superior de Agronomia, Laboratório Associado Universidade de Lisboa Lisbon Portugal; ^5^ BIOPOLIS Program in Genomics Biodiversity and Land Planning, CIBIO Vairão Portugal; ^6^ MARE – Marine and Environmental Sciences Centre ISPA – Instituto Universitário Lisbon Portugal; ^7^ cE3c ‐ Center for Ecology, Evolution and Environmental Changes & CHANGE ‐ Global Change and Sustainability Institute, Department of Animal Biology Faculty of Sciences of the University of Lisbon, 1749‐016 Lisboa, Campo Grande Lisbon Portugal; ^8^ RSPB Centre for Conservation Science Royal Society for the Protection of Birds, The Lodge Sandy UK; ^9^ British Trust for Ornithology, The Nunnery Thetford UK; ^10^ Department of Zoology University of Cambridge Cambridge UK; ^11^ School of Biological Sciences University of East Anglia Norwich UK

**Keywords:** bird migration, Convention on Migratory Species, geopolitical connectivity, migratory links, migratory species, science–policy interface, tracking data, conectividad geopolítica, conexiones migratorias, Convención de Especies Migratorias, datos de rastreo, especies migratorias, interconexión ciencia‐política, migración de aves, 鸟类迁徙, 《保护迁徙野生动物物种公约》, 地缘政治连接, 迁徙路径, 迁徙物种, 科学‐政策联系, 追踪数据

## Abstract

The conservation of long‐distance migratory birds requires coordination between the multiple countries connected by the movements of these species. The recent expansion of tracking studies is shedding new light on these movements, but much of this information is fragmented and inaccessible to conservation practitioners and policy makers. We synthesized current knowledge on the connectivity established between countries by landbirds and raptors migrating along the African–Eurasian flyway. We reviewed tracking studies to compile migration records for 1229 individual birds, from which we derived 544 migratory links, each link corresponding to a species’ connection between a breeding country in Europe and a nonbreeding country in sub‐Saharan Africa. We used these migratory links to analyze trends in knowledge over time and spatial patterns of connectivity per country (across species), per species (across countries), and at the flyway scale (across all countries and all species). The number of tracking studies available increased steadily since 2010 (particularly for landbirds), but the coverage of existing tracking data was highly incomplete. An average of 7.5% of migratory landbird species and 14.6% of raptor species were tracked per country. More data existed from central and western European countries, and it was biased toward larger bodied species. We provide species‐ and country‐level syntheses of the migratory links we identified from the reviewed studies, involving 123 populations of 43 species, migrating between 28 European and 43 African countries. Several countries (e.g., Spain, Poland, Ethiopia, Democratic Republic of Congo) are strategic priorities for future tracking studies to complement existing data, particularly on landbirds. Despite the limitations in existing tracking data, our data and results can inform discussions under 2 key policy instruments at the flyway scale: the African–Eurasian Migratory Landbirds Action Plan and the Memorandum of Understanding on the Conservation of Migratory Birds of Prey in Africa and Eurasia.

## Abstract (es)

Conectividad entre países establecida por aves terrestres y rapaces que migran a través del corredor aéreo africano‐euroasiático

**Resumen:**

La conservación de las aves que migran grandes distancias requiere de una coordinación entre los varios países conectados por los movimientos de estas especies. La expansión reciente de los estudios de rastreo está descubriendo novedades en estos movimientos, aunque gran parte de esta información está fragmentada y es inaccesible para quienes practican y elaboran las políticas de conservación. Sintetizamos el conocimiento actual sobre la conectividad establecida entre países por las aves terrestres y rapaces que migran a través del corredor aéreo africano‐euroasiático. Revisamos los estudios de rastreo para compilar los registros migratorios de 1229 aves, de los cuales derivamos 544 conexiones migratorias, con cada conexión correspondiendo a la conexión que tiene una especie entre un país europeo en donde se reproduce con un país de la África subsahariana en donde no se reproduce. Usamos estas conexiones migratorias para analizar las tendencias informativas en patrones espaciales y temporales de conectividad por país (en todas las especies), por especie (en todos los países) y a escala del corredor aéreo (en todas las especies y en todos los países). El número de estudios de rastreo disponibles incrementó gradualmente a partir de 2010 (particularmente para las aves terrestres), pero la cobertura de los datos de rastreo existentes estaba incompleta. Se rastreó en promedio 7.5% de especies de aves terrestres migratorias y 14.6% de aves rapaces por país. Existían más datos de los países del centro y oeste de Europa, los cuales estaban sesgados hacia las especies de mayor tamaño. Proporcionamos varias síntesis a nivel de especie y país de las conexiones migratorias que identificamos a partir de la revisión de estudios, las cuales involucran a 123 poblaciones de 43 especies que migran entre 28 países europeos y 43 países africanos. Varios países, como España, Polonia, Etiopía y la República Democrática del Congo son prioridades estratégicas para complementar los datos existentes en los siguientes estudios de rastreo, en especial para las aves terrestres. A pesar de las limitaciones que tienen los datos de rastreo existentes, nuestros datos y resultados pueden orientar las discusiones con dos instrumentos claves para las políticas: el Plan de Acción de las Aves Terrestres Migratorias Africanas‐Euroasiáticas y el Memorando de Entendimiento sobre la Conservación de las Aves Rapaces Migratorias de África y Eurasia.

## 【摘要】

《世界自然保护联盟 (IUCN) 红色名录》设定的标准是评估物种灭绝风险以及在适当的情况下宣布生物灭绝的黄金标准。然而, 与所有其它濒危等级不同的是, “灭绝”等级缺乏一个确定该等级的量化框架。鉴于划分这个等级存在主观性, 我们访问调查了研究多种类群的专家评估员, 以探究他们宣布物种灭绝时考虑的因素。本研究利用选择实验的方法, 调查了来自世界自然保护联盟物种生存委员会专家组和工作组的 674 名专家。我们发现, 在推断物种灭绝时, 数据可获得性、距离最后一次发现的时间、可监测性、栖息地可获得性和种群下降情况都是评估者关注的重要属性。曾参与红色名录工作的受访者在分析物种灭绝时, 更重视数据可获得性、距离最后一次发现的时间和可监测性, 而那些研究人们认识较多的类群的受访者则更重视距离最后一次发现的时间。没有参与过红色名录工作的受访者和研究人们认识较多的类群 (即哺乳动物和鸟类) 的受访者总体上更少考虑物种灭绝的问题。以上关于评估者对宣布物种灭绝影响因素的重视程度的结果, 可以为制定更准确地评估物种灭绝的具体标准提供依据。 **【翻译: 胡怡思; 审校: 聂永刚】**

## INTRODUCTION

Migratory birds undertake spectacular movements across continents and oceans, coupling distant ecosystems (Bauer & Hoye, 2014) and linking multiple political jurisdictions (Beal, Dias, et al., 2021; Harrison et al., 2018; Morrick et al., 2021). Over 2 billion landbirds (Hahn et al., 2009) and millions of raptors (Miller et al., 2016; Verhelst et al., 2011) migrate seasonally across the African–Eurasian flyway, one of the largest avian migratory systems in the world (Newton, 2008). Throughout their annual cycles, migratory birds face a suite of threats, including agricultural intensification on the breeding grounds (Reif & Vermouzek, 2019), energy infrastructure development along migratory routes (Marques et al., 2020), illegal taking at stopover sites (Brochet et al., 2016), habitat degradation in nonbreeding grounds (Zwarts et al., 2018), and climate change across their ranges (Zurell et al., 2018). As a result, many populations of African–Eurasian migrants are declining (Sanderson et al., 2006; Vickery et al., 2014).

The conservation of migratory birds is a challenge that requires concerted effort among the multiple countries connected by the movements of these birds. In the African–Eurasian flyway, 2 policy instruments focus on the conservation of migratory landbirds and raptors: the African–Eurasian Migratory Landbirds Action Plan (AEMLAP; UNEP/CMS, 2014) and the Memorandum of Understanding on the Conservation of Migratory Birds of Prey in Africa and Eurasia (Raptors MOU; UNEP/CMS, 2008). These agreements were adopted under the United Nations Convention on Migratory Species and provide frameworks for cooperation between governments and with other key stakeholders (including nongovernmental organizations, industry, and funding agencies), fostering collective action in tackling the conservation needs of migratory species, and guiding decision‐making (Baldwin, 2011; Hensz & Soberón, 2018). To be effective, however, such conservation efforts require a sound understanding of the spatial and temporal distributions of different migratory bird populations.

Bird migrations have fascinated people for millennia, but it was only with the development of ringing programs in the 20th century that the precise movements of individual birds started to become clearer (Bairlein, 2001), including their migratory connectivity patterns at the scale of the flyway (Spina et al., 2022). More recently, developments in tracking technologies (e.g., light‐level geolocators, i.e., global location sensors [GLS]; satellite transmitters, i.e., platform transmitter terminals [PTT]; and global positioning system [GPS] devices [Bridge et al., 2011]) have made it possible to follow birds with unprecedented detail and determine how long they stay at each location throughout their annual cycles. The resulting increase in bird‐tracking studies is revealing a progressively more detailed picture of the migratory behavior and connectivity patterns of many bird populations (e.g., Buechley et al., 2021; Finch et al., 2015). New opportunities are thus emerging for targeted international cooperation, wherein tracking data can play an important role in informing where and when conservation action for different populations might be most effective (e.g., Hewson et al., 2016; Knight et al., 2021).

Despite these advances, tracking studies are still far from realizing their potential to inform flyway‐scale conservation of migratory birds, including in terrestrial environments (Katzner & Arlettaz, 2020). First, and despite the increasing recognition of the utility of global data repositories, such as Movebank (Kays et al., 2021), much of the existing data are fragmented, confined to the academic literature (Fraser et al., 2018), and remain difficult to find and access (Davidson et al., 2020). Second, given that tracking studies are initiated with different underlying motivations (e.g., scientific, conservation) and their feasibility is constrained by a diversity of considerations (e.g., technology, species’ ecology, access to funding), existing data tend to be biased toward particular regions and species (Bernard et al., 2021). Even so, as the volume of data increases, it becomes progressively more important to bring them together, synthesize them into formats that are accessible to scientists and conservation practitioners, and translate their results into policy‐relevant scientific evidence (Dunn et al., 2019).

For the African–Eurasian flyway, previous studies integrating tracking records for multiple species have described general spatial and temporal patterns of migration (e.g., Briedis et al., 2020; Strandberg et al., 2009), connectivity (e.g., Finch et al., 2017), and mortality (e.g., Klaassen et al., 2014), as well as the potential impacts of threats on population dynamics (e.g., Cresswell et al., 2020). However, no study has attempted to bring together all the available tracking data in a format that can be useful to guide international cooperation at the flyway scale, namely, through the AEMLAP and the Raptors MOU. From a policy perspective, countries are the key spatial unit of analysis given that the implementation of policies steered in international fora depends on the decision‐making processes of each country (Dallimer & Strange, 2015), their national conservation priorities, and their differing capacities for implementation (Boardman, 2006). Understanding how migratory bird populations link countries throughout their annual cycle is thus key to highlighting shared conservation priorities across countries and guide effective, targeted, and equitable international cooperation efforts for their long‐term conservation.

We reviewed the tracking literature to assess the state of knowledge of the connectivity established among countries by birds migrating along the African–Eurasian migration flyway in support of international agreements for the conservation of migratory landbirds and raptors in this region (AEMLAP and Raptors MOU). We compiled all available tracking data on the links between countries created by landbirds and raptors as they migrate from breeding to nonbreeding grounds. We then synthesized the current knowledge regarding these connections at the level of individual countries, individual species, and at the flyway scale. Finally, we evaluated the extent of the remaining gaps in knowledge, proposing priorities for future bird‐tracking studies that can strategically reduce those gaps.

## METHODS

### Study region

Within the African–Eurasian migratory flyway, we focused on breeding grounds in Europe (including Turkey and excluding Russia) and on nonbreeding grounds in sub‐Saharan Africa (i.e., excluding Morocco, Western Sahara, Algeria, Tunisia, Libya, and Egypt) (Appendix S1). We did not include European Russia and Asian countries in the flyway because a preliminary examination of the literature (Briedis et al., 2020, 2019; Brlík et al., 2020; Cresswell et al., 2020; Finch et al., 2017) revealed very few studies in this region. We grouped countries into subregions: 4 in Europe (western Europe, central Europe, northern Europe, and eastern Europe) and 4 in sub‐Saharan Africa (western Africa, central Africa, southern Africa, and eastern Africa) (Appendix S1).

### Species and populations

We analyzed 118 long‐distance migratory bird species (91 landbirds covered by the AEMLAP and 27 raptors covered by the Raptors MOU), all breeding in Europe and spending the nonbreeding season in sub‐Saharan Africa (Appendix S2). We used the distribution maps from BirdLife International and Handbook of the Birds of the World (2018) to identify species’ breeding ranges in Europe and nonbreeding ranges in sub‐Saharan Africa.

We defined a population as the set of individuals of the same species that breed in a given European country (hence, we used the term *population* only to refer to a single species in a single country). We used national boundaries to define populations because our aim was to characterize links between countries. Although these national populations are not ecologically isolated, patterns of natal and breeding dispersal are likely negligible at this scale (Fandos et al., 2021; Paradis et al., 1998).

We used the terms *European population* to mean all individuals of a species that breed across Europe and *subregional population* to mean all individuals of a species in a given European or African subregion. Each of the analyzed species therefore has 1 or more (country‐level) populations, a single European population, and 1 or more European and African subpopulations.

We used the European Red List of Birds (BirdLife International, 2021), to obtain European‐level population trends (Appendix S2), the list of species (among those analyzed) per European country, and the respective country‐level population size estimates. We used the latter to calculate European and subregional populations’ sizes, from which we estimated the percentage of each species’ European or subregional populations in each country. We could not follow the same approach for sub‐Saharan countries because no country‐level population size estimates were available. Instead, we used the abovementioned distribution maps to obtain the list of migratory species per country and then calculated for each of these species the percentage of their (sub‐Saharan) nonbreeding range or subregional ranges in each country.

### Definition of migratory link

An individual migratory bird typically crosses the borders of multiple countries during its annual cycle, including where it breeds, stops over during migration, and spends the nonbreeding season. We focused on 2 countries per individual: the one where it breeds (hereafter *breeding country*) and the one where it spends the most time during the nonbreeding season (*nonbreeding country*). As we extracted data from available studies rather than from raw tracking data (see below), we were unable to extract finer details (e.g., on stopover sites) across all individuals.

We defined a *migratory link* as the connection between 2 countries established by birds from a population as they migrate from a European breeding country to a sub‐Saharan African nonbreeding country. We defined the strength of each migratory link as the proportion of individuals in the population that spend the nonbreeding season in a given country in sub‐Saharan Africa. Hence, if all individuals of a given population (breeding in a given European country) migrate to the same African country, they establish a single migratory link of 100% strength. If instead the birds spread across multiple African countries, they establish multiple links of lower strength. This measure of strength is directional (e.g., Morrick et al., 2021) and reflects the importance of an African country to the population breeding in a European country. We did not calculate the reverse (i.e., extent to which the European country is important to the nonbreeding population of the African country) because tracking studies were initiated in Europe and representativeness of African countries’ nonbreeding populations was therefore too low for broad inference.

### Compilation of migration records

We focused on tracking data obtained from birds fitted with GLS, PTT, or GPS devices, aiming to obtain as many migration records as possible for the analyzed species. A *migration record* corresponded to the minimum information needed to identify a migratory link (i.e., evidence that an individual of a given species migrated from its breeding country in Europe to its nonbreeding country in sub‐Saharan Africa). Although ringing data can provide robust insights on migratory connectivity (Ambrosini et al., 2009), we did not attempt to incorporate these data in our analyses because ring recoveries do not provide information on how long the individual spent at a given location. Indeed, ringing recoveries provide location information for single points in time and often only 1 recovery location is available for each individual (e.g., Strandberg et al., 2009), making it impossible to determine if the location is in the primary nonbreeding country (as defined above).

We conducted a review of published articles in ISI Web of Science core collection (https://www.webofknowledge.com/) and Google Scholar (https://scholar.google.com/), complemented by additional studies identified through extensive ad hoc searches on Google and consulting previous reviews (Briedis et al., 2020, 2019; Brlík et al., 2020; Cresswell et al., 2020; Finch et al., 2017) (details in Appendix S3).

From each selected study, we extracted as many migration records as possible. Each record corresponded to an individual bird for which we obtained the species, the breeding country (in Europe), and the nonbreeding country (i.e., where the bird stayed the longest in sub‐Saharan African) (details in Appendix S4, including how we dealt with highly mobile species).

For European nightjar (*Caprimulgus europaeus*), Eurasian bee‐eater (*Merops apiaster*), barn swallow (*Hirundo rustica*), and collared flycatcher (*Ficedula albicollis*), the tracking data revealed nonbreeding ranges covering more countries than those identified by BirdLife International and Handbook of the Birds of the World (2018). Therefore, we updated these ranges (details in Appendices S5 & S6) before using them in our analyses.

### Observed and inferred migratory links

We grouped all migration records by population (i.e., conspecifics breeding in the same European country) and excluded from further analysis any populations with fewer than 3 migration records. For each population, we then identified 1 or more migratory links (between a European and a sub‐Saharan country). Given the incompleteness of our data set, these *observed links* (derived from the migration records) underestimated the true number existing for each population. Nevertheless, some missing links were predictable and were inferred through interpolation between known links. For example, if for a given population breeding in European country A the migration records showed connections to 2 African countries, B and C (through observed links A–B and A–C), and if there was a third country D spatially located between B and C that was also within the species’ nonbreeding range, then link A–D likely also existed and, in such cases, we inferred that the population also migrates to country D (details of inference method in Appendix S7). Inferred links made up only 16% of all the links we analyzed, and they had a negligible effect on the results (Appendix S7).

### Estimating the strength of migratory links

Assuming tagged birds are representative of their populations, the strength of a migratory link can be estimated from the distribution of migration records among the migratory links in a population (akin to Morrick et al., 2021; van Wijk et al., 2018). Calculating this requires estimating the number of expected records for any inferred links, which we did by interpolating from number of observed migration records in neighboring countries (details in Appendix S7). We thus estimated the strength of migratory links in each population as the percentage of migration records (observed or interpolated) occurring in each country over the sum of all records across all migratory links (observed or inferred). All migratory links can be viewed in a virtual application at https://african‐eurasian‐migrants.shinyapps.io/migratory_links/.

### State of knowledge on migratory connectivity along the African–Eurasian flyway

We analyzed the data set to synthesize current knowledge of patterns of connectivity in the African–Eurasian flyway, including trends over time and spatial syntheses per country (across species), per species (across countries), and at the flyway scale (across all countries and all species). We analyzed data for landbirds and raptors separately.

We plotted the cumulative number of studies and migratory links (observed and inferred) over time, as indicators of trends in knowledge of migratory connectivity between countries along the African–Eurasian flyway. We also plotted the relationship between the number of migration records per population and the number of migratory links to investigate whether tracking effort per population appeared sufficient.

For each of the analyzed countries (European or African), we synthesized connectivity with other countries by plotting all the corresponding migratory links according to strength.

For each of the species in our data set, we synthesized the connectivity between breeding and nonbreeding countries by plotting all migratory links for each population and estimating how representative each link is of the species’ overall European population by weighting its strength relative to the percentage of the total European population breeding in each country. We then quantified the importance of each sub‐Saharan country as a nonbreeding destination for that particular species by summing the weighted values across all migratory links to each African country.

We synthesized current knowledge of spatial patterns of connectivity between countries in the flyway by generating a map of the known migratory links across all species, a map with the number of migratory links per country, and a map of the number of tracked species per country.

### Knowledge gaps

For each country in Europe or in sub‐Saharan Africa, we quantified the extent of knowledge gaps by calculating the percentage of long‐distance migratory species per country present in the country but for which we did not find migratory links. This value varied from 0% (no gaps) to 100% (all species missing).

We recommend that any flyway‐wide strategy for tracking long‐distance migratory birds to fill knowledge gaps should prioritize species with decreasing populations (as per the European Red List of Birds: 38 species, 31 landbirds, 7 raptors) (Appendix S2); prioritize countries across the flyway with the largest fractions of the population for which no migratory links are known; and aim to spread tracking effort across species’ ranges (i.e., across all subregions in both continents) to ensure new tracking data capture the main ecological gradients and a range of migratory strategies. Based on these recommendations, we identified for each species with decreasing European population, in each subregion (Appendix S1), a set of priority countries for future tracking, defined as those needed to complement existing studies to ensure that there will be records representative of at least 50% of the overall population of the subregion (Appendix S2). For example, the European turtle dove (*Streptopelia turtur*) breeds in western Europe and has the subregional population distributed across 7 countries: Spain (73.8%), France (24.9%), Portugal (0.9%), the United Kingdom (0.2%), Belgium (0.2%), the Netherlands (<0.1%), and Luxemburg (<0.1%). Our data set included migratory links for France and the United Kingdom (25.1% of the subregional population), so we highlight the turtle dove in Spain as a priority for future tracking. Through this process, we obtained a set of unique species–country combinations, each highlighting a particular species that we considered a priority for tracking in a particular country, which we synthesized into a list to support future tracking initiatives in each country.

### Software

All analyses were conducted in R (R Core Team, 2021) with a base world map at 1:50 m scale (https://www.naturalearthdata.com/) in the sf package (Pebesma, 2018). All figures were produced in ggplot2 (Wickham, 2009) with a base map at 1:110 m scale in orthographic projection.

## RESULTS

### Tracking studies, migration records, and migratory links

We identified 1496 unique studies in our literature search (Web of Science 776, Google Scholar 928) and 51 additional studies obtained through complementary searches. We retained 132 studies from which we gathered 1282 migration records (Guilherme, 2022) (Appendices S3 & S4). The final data set (excluding populations with <3 migration records) had 1229 migration records for 43 bird species (29 landbirds, 14 raptors), representing 123 populations. We had 361 records (38 populations) for western Europe, 470 (42 populations) for central Europe, 264 (26 populations) for northern Europe, and 134 (17 populations) for eastern Europe (Appendix S8). When translated into geopolitical space, the migration records revealed 544 migratory links (458 observed, 86 inferred) (Appendix S9).

### Trends in knowledge

**FIGURE 1:**
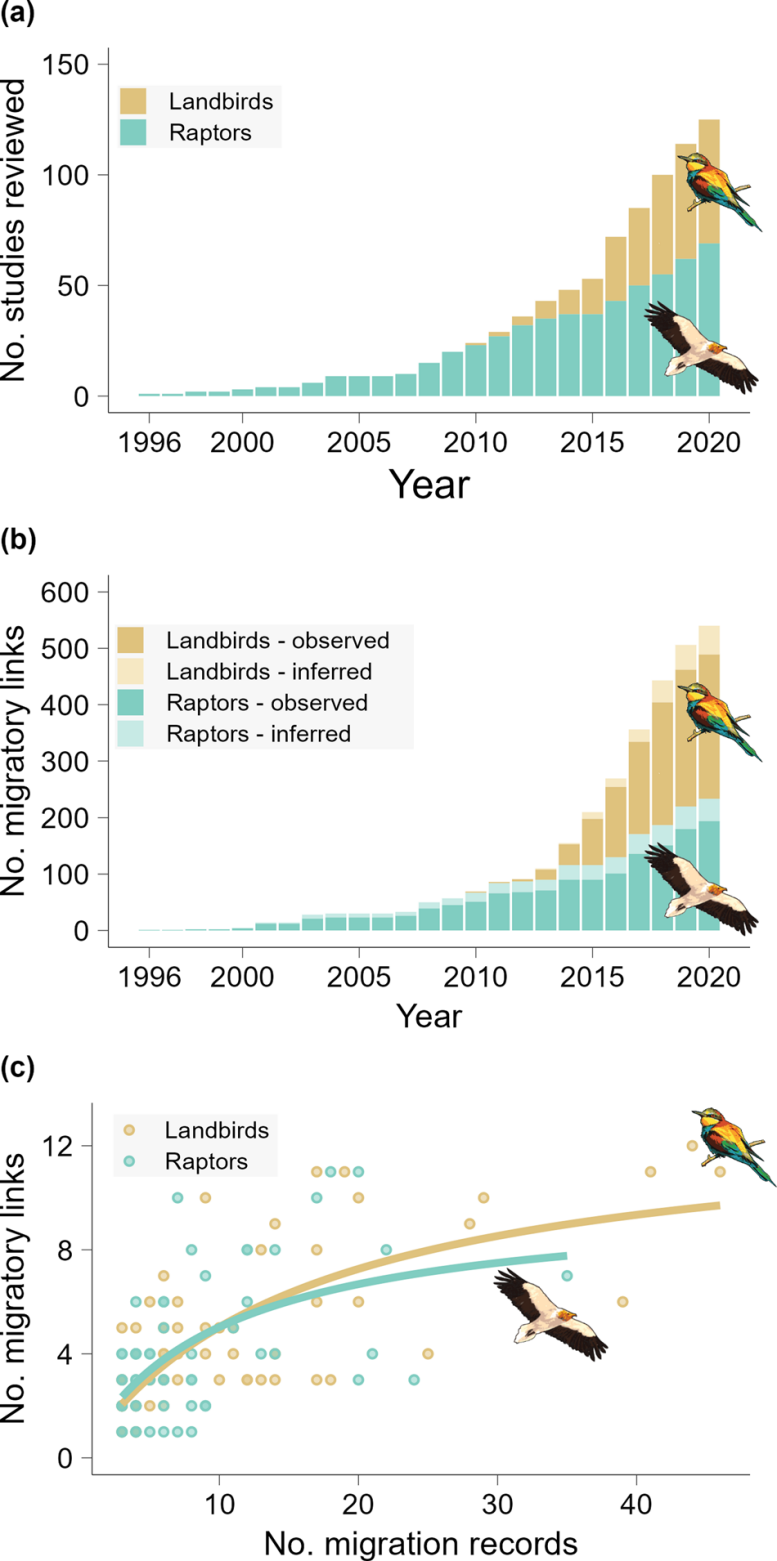
(a) Cumulative number of studies tracking migratory landbirds and raptors across the African–Eurasian flyway over time, (b) corresponding cumulative number of migratory links (connecting a breeding country in Europe to a nonbreeding country in sub‐Saharan Africa for a given species) over time (120 studies, 532 links; only years with complete data are shown [2 studies excluded; hence, 12 links excluded]), and (c) relationship between the number of migration records per population and the number of migratory links derived from them (solid lines, nonlinear regression results)

The first study (and thus migratory link) in our data set dated from 1996, and the cumulative number of studies and links increased steadily over time (Figure 1). Studies up to 2010 focused almost exclusively on raptors. Studies on landbirds increased greatly after 2010, corresponding to 48.7% of the studies we analyzed (Figure 1a) and to 57.2% of all links (Figure 1b).

### Tracking effort per population

The number of migratory links per population tended to increase with the number of migratory records, even if there was substantial variation around this trend (Figure 1c). The increase has occurred over a shorter period for landbirds than for raptors; none of the curves reached an asymptote.

### Connectivity

**FIGURE 2:**
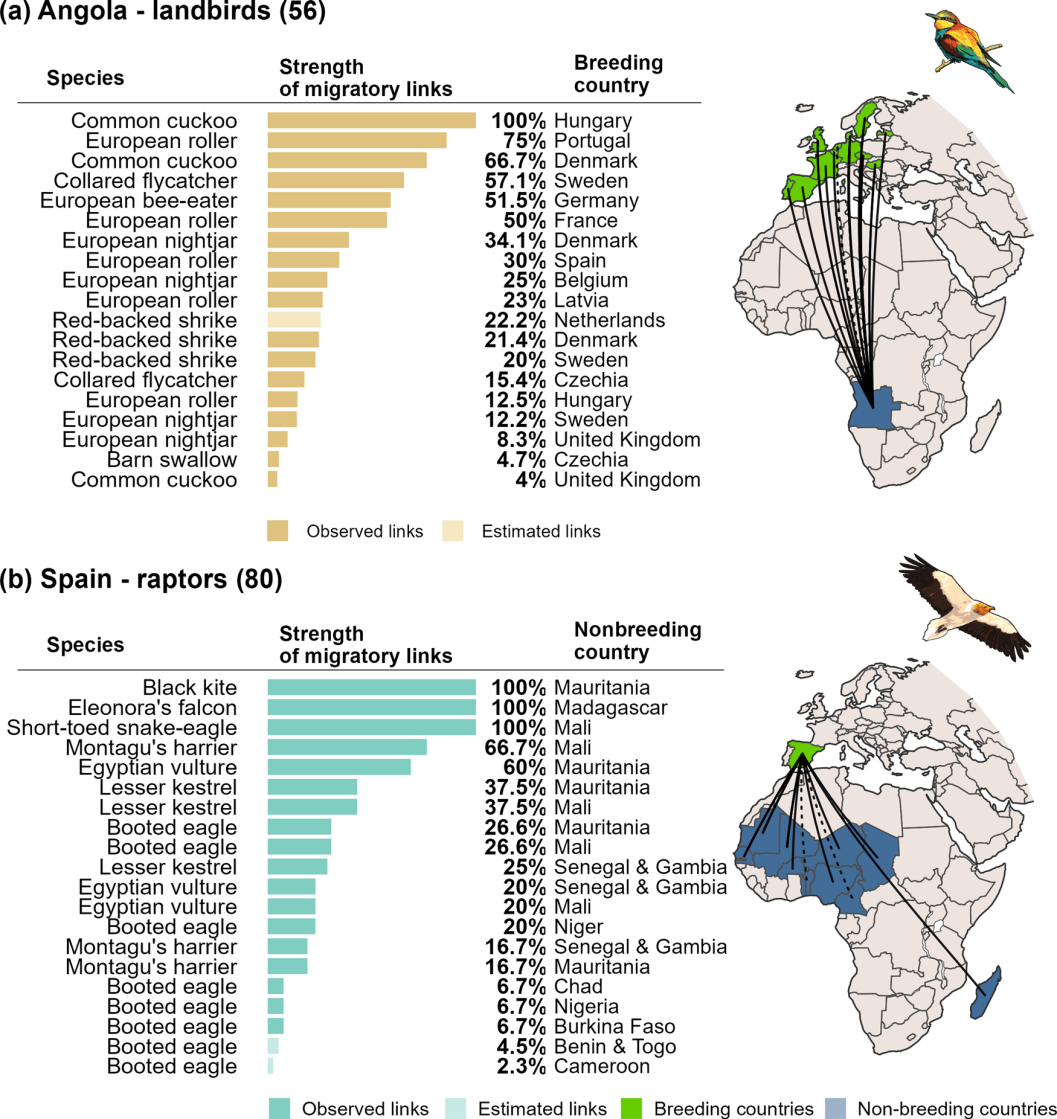
Country‐level connectivity for (a) landbirds in Angola and (b) raptors in Spain. Migratory links for species are in by decreasing order of strength of the link, indicating in each case the species creating the link and the country it connects to (numbers in parentheses, sample size of tracked individuals; lines on maps, observed [solid] and inferred [dotted] migratory links between countries in Europe [green] and countries in sub‐Saharan Africa [blue]). Detailed results for all countries in Appendix S10

Mapping migratory links per country revealed their connections to other countries through the long‐distance migrations of bird populations (Figure 2a). We found migratory links between 28 European countries and 43 sub‐Saharan African countries. There was substantial variation in the number of links and species tracked per country. On average, each of the European countries analyzed had 19.4 (range 1–63) migratory links, established by 4.4 (1–14) species that linked them to 12.5 (1–27) countries in sub‐Saharan Africa. Conversely, each of the African countries had on average 14.7 (1–47) migratory links, established by 8.6 (1–21) species that linked them to 9.5 (1–17) European countries (Table 1; Appendix S10).

**TABLE 1:** Summary of the state of knowledge regarding country‐level and species‐level connectivity established by landbirds and raptors migrating along the African–Eurasian flyway*

Connectivity		Landbirds	Raptors
Country level			
Europe	Number of known migratory links	14.1 (1–42)	10.1 (1–31)
	Number of tracked species	3.0 (1–7)	2.4 (1–7)
Africa	Number of known migratory links	8.9 (1–22)	6.5 (1–25)
	Number of tracked species	5.8 (1–13)	3.2 (1–8)
Species level			
Species	Number of known migratory links	10.7 (1–52)	16.6 (2–44)
	Number of European countries (i.e., populations) tracked	2.3 (1–6)	4.0 (1–7)
	Number of African countries tracked	6.97 (1–27)	8.3 (1–25)
Population	Number of known migratory links	4.6 (1–12)	4.2 (1–11)

* Values are mean with range in parentheses.

**FIGURE 3:**
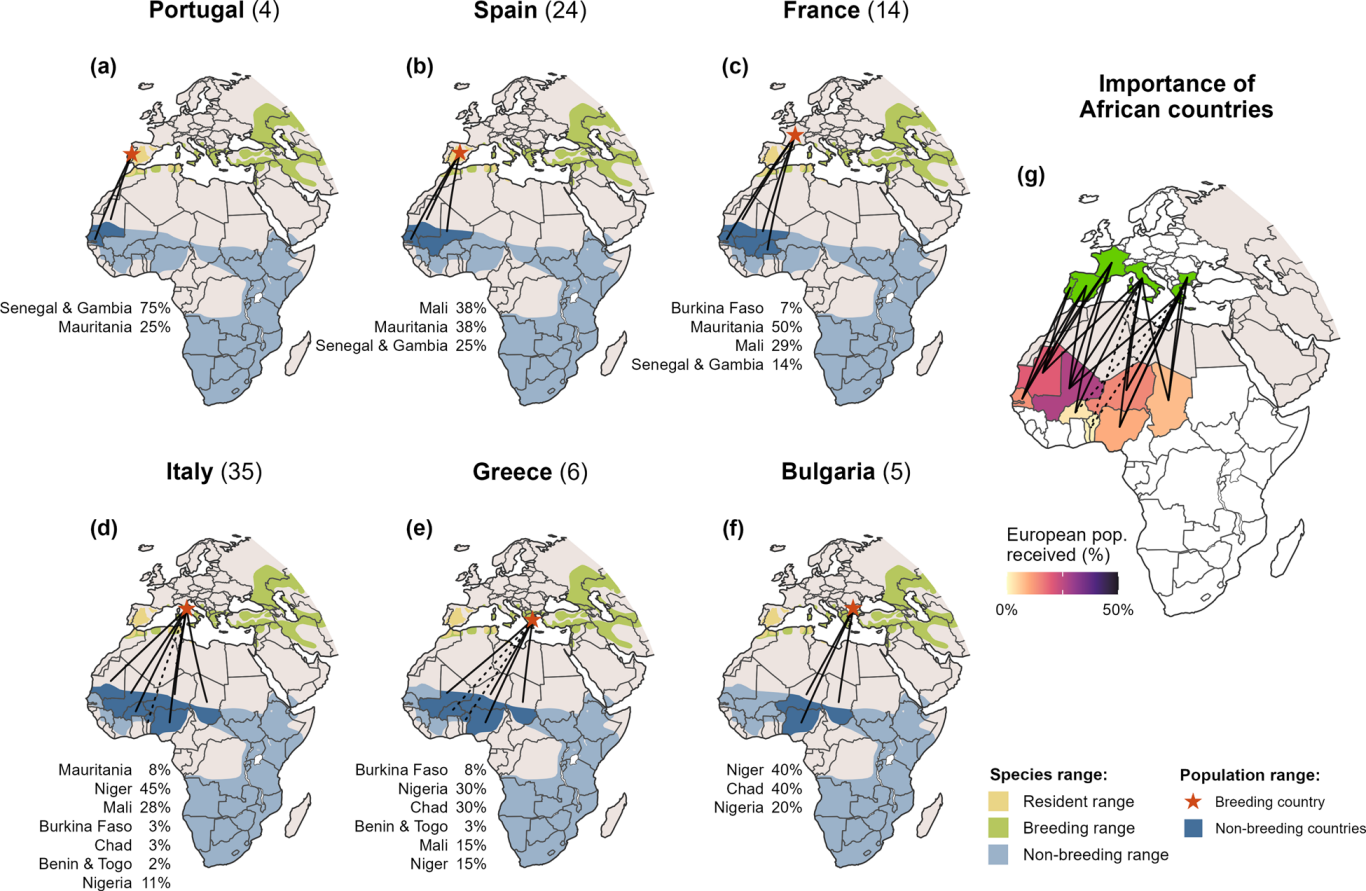
Connectivity between countries for each of the 6 populations in our data set of lesser kestrel (*Falco naumanni*) (summarised in Sarà et al., 2019), each map corresponding to the set of birds that breed in a given European country: (a) Portugal, (b) Spain, (c) France, (d) Italy, (e) Greece, and (f) Bulgaria (numbers in parentheses, sample size of tracked individuals; lines on maps, observed [solid] and inferred [dotted] migratory links; percentages, strength of links; colors, species’ resident range [yellow], species’ breeding range [green], species’ nonbreeding range [light blue], and populations’ nonbreeding countries [dark blue]), and (g) importance of each country in sub‐Saharan Africa as nonbreeding grounds for the European population of lesser kestrel in our data set, as revealed by the migratory links (lines coded as in the other panels). Detailed results for all species in Appendix S11

The number of migratory links per species varied substantially and was generally higher for raptors (average 16.6) than for landbirds (10.7; Table 1). Each species was tracked on average in 2.8 (1–7) populations (i.e., countries) across its European breeding range and the respective migratory records showed nonbreeding grounds in 7.4 (1–27) African countries (Table 1). On average, across all populations of all species analyzed, we found 4.4 (1–12) migratory links per population. Mapping the migratory links for each population of each species separately (Figure 3a–f) showed how they connect to countries in sub‐Saharan Africa (Appendix S11).

The patterns of relative importance of each country in sub‐Saharan Africa as a nonbreeding ground for each species varied substantially across species. For example, great reed‐warblers (*Acrocephalus arundinaceus*) from the 5 populations in our data set spread across 21 African countries, which were estimated to receive from <1% (Liberia) to 9.3% (Sierra Leone) of the European population (Appendix S11). Montagu's harriers (*Circus pygargus*) from 7 populations in Europe concentrated in 9 African countries, which were estimated to receive from <1% (Ghana) to 23.9% (Niger) of the European population. Mapping all migratory links per species (Figure 3g) showed the importance of the nonbreeding grounds across countries in sub‐Saharan Africa (Appendix S11).

**FIGURE 4:**
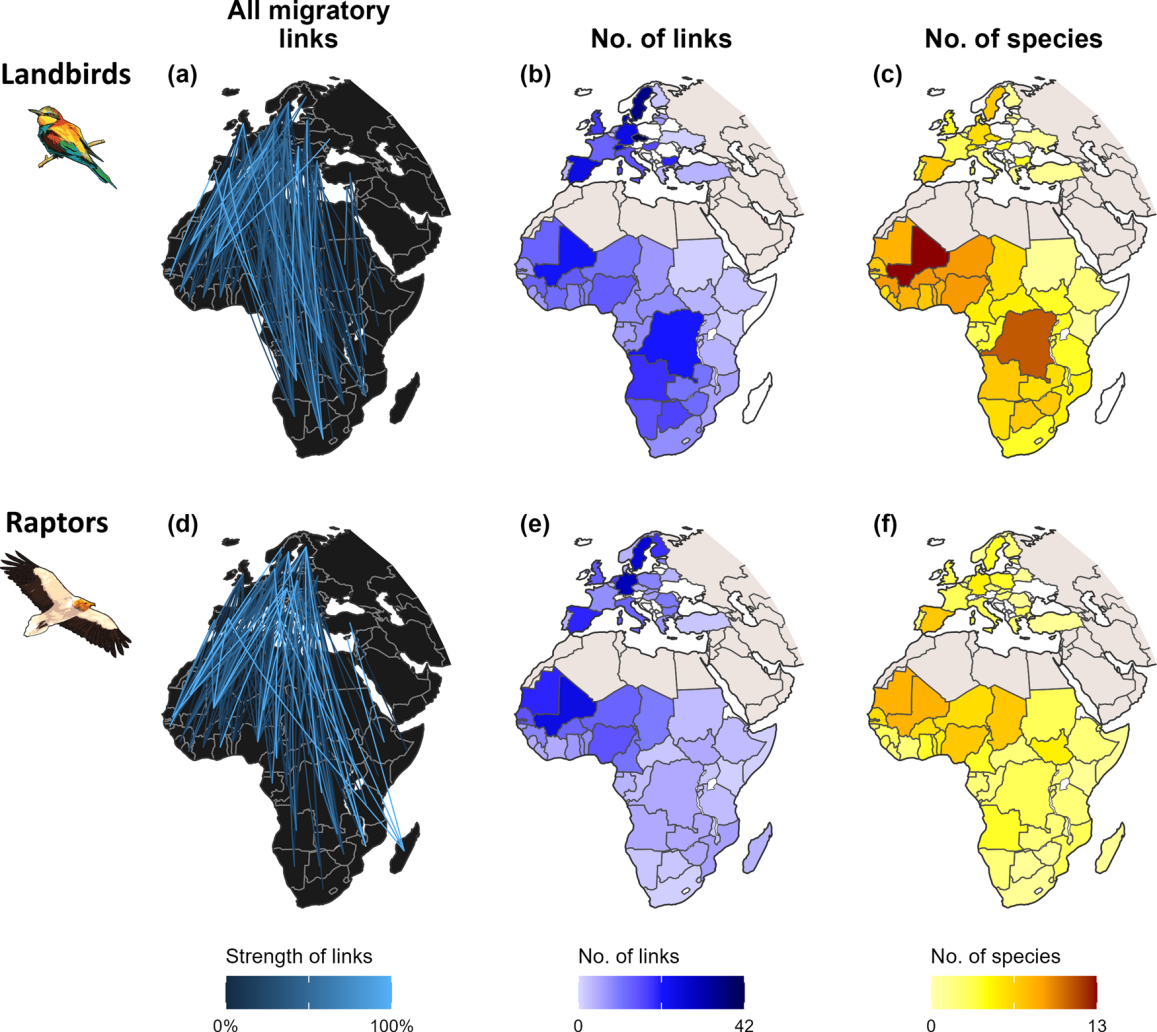
Connectivity established between countries by landbirds and raptors migrating along the African–Eurasian flyway: (a, d) all migratory links (observed and inferred) obtained from the tracking studies reviewed, (b, e) number of migratory links per country, and (c, f) number of species tracked per country

Mapping all known migratory links across species (Figure 4a,d) revealed a complex network of ecological connectivity between European and African countries created by landbirds (Figure 4a) and raptors (Figure 4d). The number of migratory links (Figure 4b,e) and species tracked (Figure 4c,f) varied substantially across countries. In Europe, 4 countries stood out in number of links and species tracked: Sweden (63 links, 11 species), Germany (56, 11), the Czech Republic (47, 8), and Spain (45, 14). For most eastern European countries, we found relatively few links and few species tracked.

In Africa, the western subregion stood out in terms of the number of migratory links for both landbirds and raptors, in particular Mali (47 links, 21 species), but also Mauritania (33, 16), Nigeria (29, 16), and Burkina Faso (27, 15). Countries in central and southern Africa also stood out for links for landbirds (but not for raptors), in particular the Democratic Republic of Congo (26 links, 14 species), Angola (24, 11), Botswana (19, 8), and Namibia (17, 8). We found few links and few tracked species for countries in eastern Africa (e.g., Somalia, Kenya).

### Knowledge gaps

**FIGURE 5:**
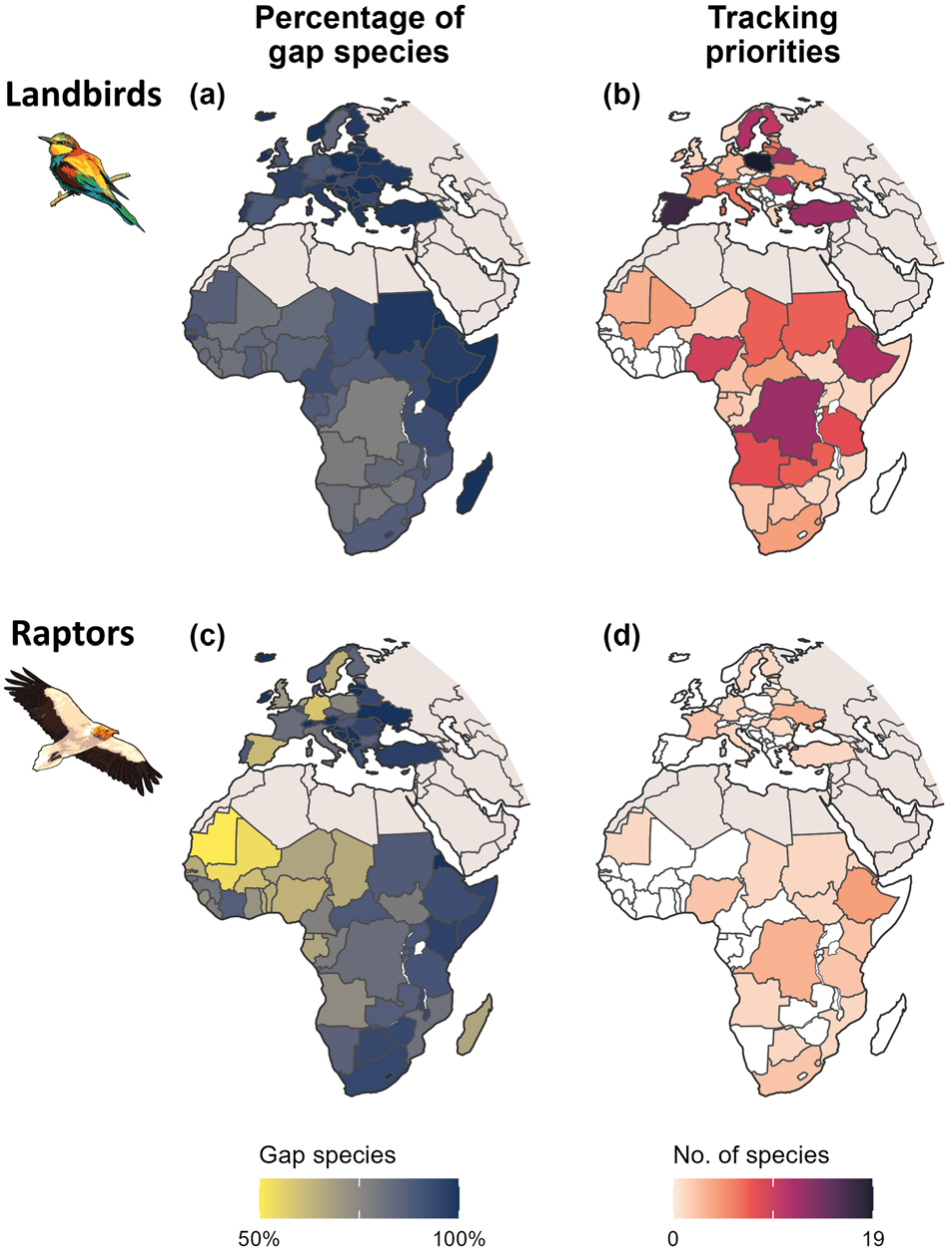
(a, c) Percentage of migratory species of landbirds and raptors that had no migratory links between countries in the African–Eurasian flyway in our data set (i.e., gap species) and (b, d) priority countries for future tracking studies, based on the number of species with decreasing European populations for which the country is a priority for covering at least 50% of the species’ population in each subregion in Europe or in Africa (map of subregions in Appendix S1). Detailed results for all priority species–country combinations in Appendix S12

The vast majority of long‐distance migratory bird species in each country had not been tracked (Figure 5a,c; Appendix S12). In Europe, the average percentage of gap species per country was 96.7% for landbirds (minimum 83.7% in Denmark, maximum 100% in 21 countries) and 90.4% for raptors (minimum 58.3% in Germany, maximum 100% in 19 countries). In Africa, there were on average 87.8% gap species per country for landbirds (minimum 76.6% in the Democratic Republic of the Congo, maximum 100% in 3 countries) and 79.7% for raptors (minimum 50% in Mauritania, maximum 100% in Eritrea and Lesotho).

### Priorities for future tracking

We identified 287 species–country combinations as priorities for future tracking (6.5% of the gaps) (Appendix S12), mostly for landbirds (248) (Figure 5b) but also for raptors (39) (Figure 5d). These were spread across the study region in countries that concentrated relatively large population numbers in each subregion. In Europe, these countries included Poland (19 species) in central Europe, Spain (17) in western Europe, Turkey and Belarus in eastern Europe (13), and Sweden in northern Europe (12) (Figure 4b,d). In African countries, these included the Democratic Republic of Congo (15 species) in central Africa, Ethiopia (15) in eastern Africa, Nigeria (11) in western Africa, and Angola (9) in southern Africa (Figure 5b,d).

## DISCUSSION

### Wealth of data on the African–Eurasian flyway

Our study sheds light on the wealth of data acquired from the tracking of thousands of African–Eurasian migratory landbirds and raptors from 1996 to 2021 (Appendix S4; Figure 1a). Compiled into migratory links, these data revealed how migratory birds connect countries in breeding areas in Europe and nonbreeding grounds in sub‐Saharan Africa. Further synthesized per country (Appendix S10), per species (Appendix S11), and at the flyway scale (Figure 4), these data can inform international cooperation efforts for conserving migratory birds, particularly for well‐studied species and well‐sampled countries, and they highlight potential priorities for future tracking efforts.

For example, existing data revealed how the Danish population of willow warblers (*Phylloscopus trochilus*) disperses across 9 countries in western and central Africa (Lerche‐Jørgensen et al., 2017), establishing migratory links of relatively low strength (average 11.1%), whereas European rollers (*Coracias garrulus*) tagged in 6 European countries established fewer but stronger links (26%) with 6 southern African countries (Finch et al., 2015). Ospreys (*Pandion haliaetus*) had contrasting patterns across populations. Finish breeding birds dispersed broadly across 11 countries in Africa (Saurola, 2020), whereas birds from the United Kingdom appeared to concentrate in just 5 western African countries (Mackrill, 2017), particularly Senegal and Gambia (together hosting 62% of that population) (Appendix S11). These syntheses provide key information to support species‐focused international cooperation efforts, including through species’ action plans. For example, the Flyway Action Plan for the European roller strongly recommends habitat protection (e.g., through agri‐environment schemes) and additional research and monitoring in nonbreeding areas (Tokody et al., 2017). Our results indicate that focusing those efforts on Namibia, Angola, and Botswana would benefit populations breeding across Europe (Appendix S11).

Our country‐level syntheses (Appendix S10) revealed opportunities for governments and other stakeholders to prioritize bilateral or multilateral cooperation among countries sharing important migratory links. For example, tracking data for 11 species breeding in Germany revealed 56 migratory links with 27 African countries, with Mali standing out as particularly important for 4 of these populations (link strength ≥40%). For Angola, 11 species created 24 migratory links with 16 countries in Europe, including major links (strength ≥67%) with Hungary, Portugal, and Denmark. Knowledge of migratory links between countries can foster strategic conservation action, including scientific and monitoring programs, capacity building, technical exchanges, and education and social empowerment initiatives. For example, the expertise of conservationists on how to reduce electrocution and poisoning of Egyptian vultures (*Neophron percnopterus*) in the Balkans is now being applied to reduce these threats along the eastern flyway after tracking identified where these threats were most prominent (Oppel et al., 2021). For some countries, these collaborations may be a cost‐effective way to deliver on national conservation priorities and could therefore be explicitly incorporated into national biodiversity plans and strategies.

At the flyway scale, the data we synthesized (Appendices S8–S11; Figure 4) can directly inform the 2 key policy instruments under the Convention on Migratory species already promoting the coordinated conservation of African–Eurasian migratory landbirds and raptors: the AEMLAP and the Raptors MOU. Even though these results are based on current knowledge (thus on incomplete and biased data), they indicated that cooperation between countries in Europe and in western Africa is strategic for the effective implementation of both agreements. Mauritania and Mali, in particular—2 countries with poor protection measures for migratory birds (Runge et al., 2015)—are connected by important migratory links (≥33%) to 14 European countries for 19 and 22 populations of landbirds and raptors, respectively (Appendix S11). Given the generally poor knowledge of the conservation needs of and threats faced by migratory bird populations on their nonbreeding grounds, prioritizing countries, such as Mali and Mauritania, for on‐the‐ground research can greatly enhance understanding of threats across multiple populations and inform direct conservation action (Vickery et al., 2014). Conversely, countries such as Spain and Sweden host relatively high numbers of species that spend their nonbreeding season in African countries (Figure 4c,f; Appendices S10 & S11); thus, they have a great responsibility for the conservation of this shared heritage.

### Knowledge gaps

Our results highlight that existing tracking data are incomplete (Appendix S12). Across all 2565 populations (1982 of landbirds, 583 of raptors) of long‐distance migratory landbirds and raptors in Europe (i.e., 118 species across 43 countries), only 123 (4.8%) have been tracked (3.4% for landbirds, 9.6% for raptors). Across the populations analyzed (i.e., with at least 3 migration records), sample sizes were generally small (on average 11.1 individuals for landbirds, 8.6 for raptors), which means that for many of them the number of migratory links is likely to have been underestimated (Figure 1c). Among the species tracked, only a fraction of the total European population was represented in our data set (19.6% [range 0.012–100] for landbirds; 48.8% [3.34–99.2] for raptors). Moreover, coverage of tracked populations is biased toward just a few countries in western and central Europe; 50% of the migration records we collated (translating into 44% of the migratory links) came from birds tagged in 5 countries (Spain, Sweden, Czech Republic, Germany, and Italy) (Appendix 10). Eastern European countries tend to be less studied, as testified by the fewer links (Figure 4b,f; Appendix S10) and higher percentages of gap species (Figure 5a,d) we found per country. The paucity of tracking data from central Asian countries led us to exclude this region altogether.

The incompleteness of and biases in our data set mean there are caveats to our interpretation of results. Estimates of relative strength of migratory links per population (Appendix S11) need to be interpreted as approximations, particularly for populations with small numbers of tracked birds. For example, we estimated for the population of great spotted cuckoo (*Clamator glandarius*) breeding in Spain that 66% migrates to Mauritania and 33% to Senegal and Gambia, but this was based on just 3 individuals. Furthermore, for those populations tracked with archival tags (mainly GLS [birds must be recaptured to recover the tracking data]), spatial variation in mortality during the nonbreeding season can affect the distribution and strength of migratory links.

Insufficient and biased coverage of tracked populations across Eurasia may have led to strong underestimates of the importance of parts of the nonbreeding range for many species. For example, all 25 migratory links we found for the lesser kestrel pointed to western African countries as major nonbreeding grounds (Figure 3). However, because only 6 populations were tracked, this does not indicate other parts of the nonbreeding range of this species are less important. Indeed, lesser kestrels also form important congregations in southern Africa, likely corresponding to populations breeding in eastern Europe and Asia (Rodríguez et al., 2011). More broadly, landbirds (Briedis et al., 2020) and raptors (e.g., Buechley et al., 2021) from western and central European countries tend to migrate along westerly routes and spend the nonbreeding season in the western half of the sub‐Saharan region, whereas birds from eastern breeding countries tend to migrate and spend the nonbreeding season in the eastern half of the region. As a result, the tracking bias toward western European populations likely played a substantial role in the spatial patterns we identified for sub‐Saharan Africa, including the dominance of links (Figure 4b,e) and species tracked per country in western Africa (Figure 4c,f) and the high numbers of gap species in eastern Africa (Figure 5a,d). Our flyway‐level syntheses (Figure 4) thus need to be interpreted with caution: results reflect only those populations for which tracking data were available and may not represent broader European populations and even less so the overall flyway population.

Our data set is also taxonomically biased. It covered only 32% of the landbirds analyzed and 52% of raptors for which we could find tracking records. Besides the number of species, body size played a major role in this bias because devices for tracking smaller species have only been developed recently and, even today, larger devices have many advantages, such as reliability, longer battery life, and remote data transmission (Bridge et al., 2011). This explains why raptors were tracked earlier than landbirds (Figure 1) and why raptors are better covered per country in terms of species tracked (on average, 7.5% landbirds, 14.6% raptors) (Figure 4c,g) and number of migratory links (Figure 4b,f). Thus, it also explains why countries have lower percentages of gap species for raptors than for landbirds (Figure 5a,c).

### Toward a flyway‐scale understanding of geopolitical connectivity

Obtaining a more complete understanding of the connectivity patterns created by migratory birds along the African–Eurasian flyway will necessarily involve collecting more tracking data. This needs a strategic approach involving all stakeholders—from scientists to conservationists, policy makers, and funders—because the associated costs and technical expertise are not trivial. Here, we devised a set of priorities for extending the coverage of tracking studies (Appendix S12) that can contribute substantially to a more representative understanding of the international connectivity patterns of migratory species along the African–Eurasian flyway. Despite corresponding to a small fraction (6.5%) of current knowledge gaps (Figure 5a,c), these priorities focus on those species most in need of conservation action (i.e., with decreasing European population) for which such understanding could make more of a difference.

Our proposed priorities are intended as an illustration of how the available data can underpin a strategic plan to guide research for filling knowledge gaps. Although we recommend the general principles proposed (i.e., prioritizing species most in need of conservation and tracking a demographically and ecologically representative sample of individuals in each case), stakeholders may well want or need to incorporate other factors into their decision‐making process (e.g., economic costs, technical constraints, or expertise availability). We believe the data we collated and synthesized, integrated with complementary ringing data (e.g., EURING Eurasian African Bird Migration Atlas [Spina et al., 2022]), can support such strategic planning, namely, through the AEMLAP and the Raptors MOU, as well as by the scientific community through initiatives like the Migratory Landbird Study Group (https://migrantlandbirds.org/).

Very few tracking studies have thus far been initiated in Africa (but see Blackburn et al., 2017; Meyburg et al., 2001), and we recommend that this imbalance be redressed. Focusing tracking efforts in African countries will help complement the information obtained from birds tagged in their European breeding areas, giving us a better picture of the migratory links between the 2 continents and thus creating a fairer information base for all countries in the flyway to make decisions for the establishment of international collaborations. Moreover, some of the birds tracked in Africa will migrate to eastern breeding grounds (e.g., Rodríguez et al., 2011; Sokolovskis et al., 2018), providing much needed information of the eastern part of the flyway.

For tracking data to contribute to conservation policy, they need to be findable and accessible. We focused on just the breeding and the main nonbreeding countries for each bird. However, much more detailed information could be obtained from reanalyses of full tracks, including distributions across the annual cycle (Carneiro et al., 2020), stopover sites along migration routes (Knight et al., 2021), and identification of key sites for conservation (Beal, Oppel, et al., 2021; Morrick et al., 2021), mortality hotspots (Klaassen et al., 2014), and threats along the flyway (Oppel et al., 2021) across species and populations. Repositories such as Movebank (https://www.movebank.org/) and the Seabird Tracking Database (http://seabirdtracking.org/) host billions of animal locations (Kays et al., 2021) from across the globe in standardized formats, facilitating scientific collaborations (e.g., Beal, Dias, et al., 2021; Davidson et al., 2020) and providing a crucial link between scientists, practitioners, and policy makers. We therefore encourage researchers to deposit all tracking data in appropriate repositories such as these.

## Supporting information

Appendix S1: Map of study region
Figure S1. Map of the study region, with countries grouped into subregions.
Appendix S2: Species list and population trends ‐ Table S2. List of the 118 species of African–Eurasian long‐distance migratory landbirds and raptors analyzed. Common name, scientific name and synonyms as used in the literature reviews search strings (details in Appendix S3). European population trend (‘decreasing’, ‘other’) according the European Red List of Birds (BirdLife International, 2021). Taxonomy follows the Handbook of the Birds of the World and BirdLife International's checklist (version 5; http://datazone.birdlife.org/species/taxonomy).
Appendix S3: Review of studies ‐ Figure S3. Flowchart illustrating the steps followed to identify the studies used as sources of bird migration records, showing the number of studies at each step.
Appendix S4: Migration records compiled ‐ Table S4. List of the migration records obtained from the identified studies. The data set is available from Guilherme (2022).
Appendix S5: Updated no‐nbreeding range maps ‐ Figure S5. Updated nonbreeding range maps used in this study for: (a) European nightjar, (b) European beeeater, (c) barn swallow, and (d) collared flycatcher ‐ shapefiles in Appendix S6 (separate file)
Appendix S7: Estimation of migratory links' strength ‐ Figure S7. Taking as example the lesser kestrel (Falco naumanni) population breeding in Greece, the figure below illustrates: (a) the observed migratory links, with the number of migration records (i.e., tracked individuals) as obtained from the literature. The area in dark blue corresponds to the species’ nonbreeding range as mapped in BirdLife International and Handbook of the Birds of the World (2018) within the countries with migration records; in light blue the nonbreeding range elsewhere. For each migratory link, the position in the nonbreeding country is the centroid of the respective dark blue polygon. (b) Minimum convex polygon encompassing the centroids in nonbreeding countries. This touches the nonbreeding range within 2 additional countries (in violet), resulting in 2 new inferred migratory links (dashed lines). (c) Number of migratory records for the inferred migratory links (see formula in main text), estimated from the density of records in neighboring countries. (d) Estimated strength of migratory links created by a population of lesser kestrels migrating between Greece and each of 6 countries within the species’ nonbreeding range in Sub‐Saharan Africa.
Appendix S8: Migration records by European region ‐ Figure S8. Number of migration records compiled in this study and respective populations organized by region in Europe.

Supporting Material

Appendix S9. List of migratory links; also available in a virtual application at https://african‐eurasian‐migrants.shinyapps.io/migratory_links/

Appendix S10. Mapping country‐level connectivity for all countries in Europe and sub‐Saharan Africa.

Appendix S11. Mapping species‐level connectivity for species of long‐distance migratory landbirds and raptors

Appendix S12: List of species‐country combinations identified as priorities for future tracking
