# Supplementary material for: Connectivity between countries established by landbirds and raptors migrating along the African–Eurasian flyway
Source: Conserv Biol. 2022 Dec 15;37(1):e14002. doi: 10.1111/cobi.14002 (PMC10107209; doi:10.1111/cobi.14002)
Supplement: Supplementary file 1 — Appendix S1: Map of study region Figure S1. Map of the study region, with countries grouped into subregions. Appendix S2: Species list and population trends ‐ Table S2. List of the 118 species of African–Eurasian long‐distance migratory landbirds and raptors analyzed. Common name, scientific name and synonyms as used in the literature reviews search strings (details in Appendix S3). European population trend (‘decreasing’, ‘other’) according the European Red List of Birds (BirdLife International, 2021). Taxonomy follows the Handbook of the Birds of the World and BirdLife International's checklist (version 5; http://datazone.birdlife.org/species/taxonomy). Appendix S3: Review of studies ‐ Figure S3. Flowchart illustrating the steps followed to identify the studies used as sources of bird migration records, showing the number of studies at each step. Appendix S4: Migration records compiled ‐ Table S4. List of the migration records obtained from the identified studies. The data set is available from Guilherme (2022). Appendix S5: Updated no‐nbreeding range maps ‐ Figure S5. Updated nonbreeding range maps used in this study for: (a) European nightjar, (b) European beeeater, (c) barn swallow, and (d) collared flycatcher ‐ shapefiles in Appendix S6 (separate file) Appendix S7: Estimation of migratory links' strength ‐ Figure S7. Taking as example the lesser kestrel (Falco naumanni) population breeding in Greece, the figure below illustrates: (a) the observed migratory links, with the number of migration records (i.e., tracked individuals) as obtained from the literature. The area in dark blue corresponds to the species’ nonbreeding range as mapped in BirdLife International and Handbook of the Birds of the World (2018) within the countries with migration records; in light blue the nonbreeding range elsewhere. For each migratory link, the position in the nonbreeding country is the centroid of the respective dark blue polygon. (b) Minimum convex polygon encompassing the cent [file COBI-37-0-s004.pdf]

Connectivity between countries established by landbirds and raptors migrating along the African-Eurasian flyway

Guilherme et al. (2022). Conservation Biology. DOI: 10.1111/cobi.14002

List of Appendices

Appendix S1: Map of study region ... 2

Figure S1. ... 3

Appendix S2: Species list and population trends ... 4

Table S2. ... 5

Appendix S3: Review of studies ... 8

A. Review of published studies ... 8

B. Complementary searches ... 9

Figure S3. ... 10

Appendix S4: Migration records compiled ... 11

Table S4. ... 12

Appendix S5: Updated nonbreeding range maps ... 20

Appendix S7. Estimation of migratory links' strength ... 22

A. Details of the method ... 22

B. Sensitivity of results to including inferred migratory links ... 23

Figure S7. ... 24

Appendix S8: Migration records by European region ... 25

Figure S8. ... 26

References ... 27

Appendix S1 ... 27

Appendix S2 ... 27

Appendix S3 ... 28

Appendix S4 ... 29

Appendix S5 ... 42

Appendix S7 ... 44

Appendix S1: Map of study region

Map of the study region with the countries analyzed structured into subregions

Our broad study region is the African-Eurasian migration flyway. Within this, we focused on breeding grounds in European countries (west of 40º E; including Turkey and excluding Russia) and on nonbreeding grounds in sub-Saharan Africa (south of 18º N; i.e., excluding Morocco, Western Sahara, Algeria, Tunisia, Libya, and Egypt).

For analysis, we merged the smaller sub-Saharan countries (those with latitudinal and/or longitudinal extents <200 km) to their neighboring countries: Gambia to Senegal; Togo to Benin; Equatorial Guinea to Gabon; Burundi to Rwanda; Djibouti to Ethiopia; Swaziland to South Africa; and Angola's enclave Cabinda to the Republic of Congo (see below; Fig. S1). This was because some of the tracking data are not sufficiently precise to confidently assign the presence of an individual bird to one of these smaller countries (notably those data obtained from geolocators, with location errors ~200 km; Lisovski et al., 2020).

We grouped European countries into four subregions (western Europe, central Europe, northern Europe, and eastern Europe) and African countries into other four (western Africa, central Africa, southern Africa, and eastern Africa) (Fig. S1).

We defined the northern limit of the sub-Saharan African region as following the northern boundary of the 'Sahelian Acacia Savanna' ecoregion according to the Terrestrial Ecoregions of the World (Olson et al., 2001).

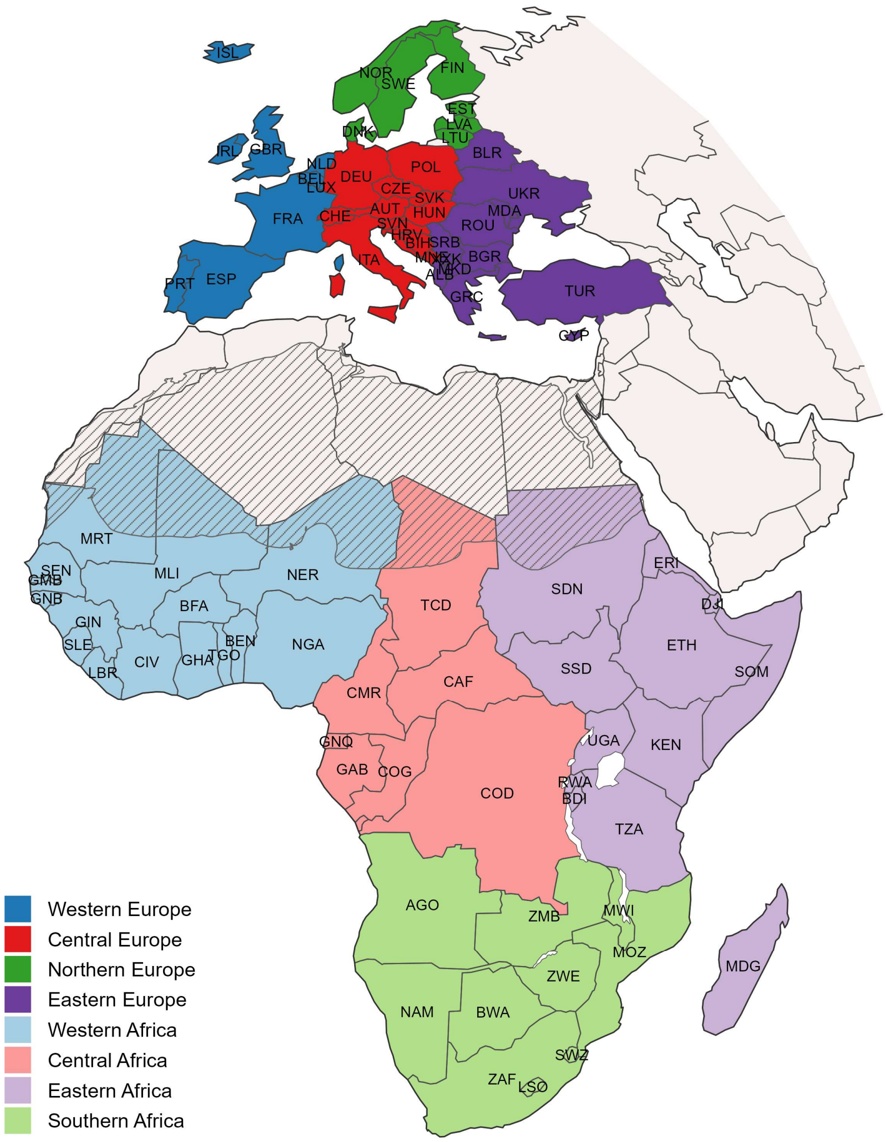

Figure S1. Map of the study region, with countries grouped into subregions. Countries are labelled after the ISO3 international standard. **Western Europe: AND** - Andorra, **BEL** - Belgium, **FRA -** France, **ISL** - Iceland, **IRL** - Ireland, **LUX** - Luxembourg, **NLD** – The Netherlands, **PRT** - Portugal, **ESP** - Spain, **GBR** – United Kingdom. **Central Europe: AUT** - Austria, **BIH** - Bosnia and Herzegovina, **HRV** - Croatia, **CZE** - Czech Republic, **DEU** - Germany, **HUN** - Hungary, **ITA** - Italy, **MNE** - Montenegro, **POL** - Poland, **SVK** - Slovakia, **SVN** - Slovenia, **CHE** – Switzerland. **Northern Europe: DNK** - Denmark, **EST** - Estonia, **FIN** - Finland, **KAL** - Kaliningrad, **LVA** - Latvia, **LTU** - Lithuania, **NOR** - Norway, **SWE** – Sweden. **Eastern Europe: ALB** - Albania, **BLR** - Belarus, **BGR** - Bulgaria, **CYP** - Cyprus, **GRC** - Greece, **KOS** - Kosovo, **MKD** - Macedonia, **MLT** - Malta, **MDA** - Moldova, **ROU** - Romania, **SRB** - Serbia, **TUR** - Turkey, **UKR** – Ukraine. **Western Africa: BEN** - Benin, **BFA** - Burkina Faso, **CIV** - Côte d'Ivoire, **GHA** - Ghana, **GIN** - Guinea, **GMB** – Gambia, **GNB** - Guinea Bissau, **LBR** - Liberia, **MLI** - Mali, **MRT** - Mauritania, **NER** - Niger, **NGA** - Nigeria, **SEN** - Senegal, **SLE** - Sierra Leone, **TGO** - Togo. **Central Africa: CAF** - Central African Republic, **CMR** - Cameroon, **TCD** - Chad, **COD** - Democratic Republic of Congo, **GAB** – Gabon, **GNQ** - Equatorial Guinea, **COG** – Republic of Congo & Cabinda. **Southern Africa: AGO** - Angola, **BWA** - Botswana, **LSO** - Lesotho, **MWI** - Malawi, **MOZ** - Mozambique, **NAM** - Namibia, **SWZ** – Swaziland, **ZAF** - South Africa, **ZMB** - Zambia, **ZWE** – Zimbabwe. **Eastern Africa: BDI** – Burundi, **ERI** - Eritrea, **ETH** - Ethiopia & Djibouti, **KEN** - Kenya, **MDG** - Madagascar, **RWA** - Rwanda, **SOM** - Somalia, **SSD** - South Sudan, **SDN** - Sudan, **TZA** - Tanzania, **UGA** – Uganda.

Appendix S2: Species list and population trends

List of the 118 species of African-Eurasian long-distance migratory landbirds and raptors analyzed and European population trend

We analyzed long-distance migratory landbirds and raptors covered by the African-Eurasian Migratory Landbirds Action Plan (AEMLAP; UNEP/CMS, 2014) and the Memorandum of Understanding on the Conservation of Migratory Birds of Prey in Africa and Eurasia (Raptors MOU; UNEP/CMS, 2008), which breed in European countries (west of 40°E) and spend the nonbreeding season in sub-Saharan Africa (south of 18° N).

For selecting species for this analysis, we used species' distribution maps form BirdLife International and Handbook of the Birds of the World (2018), which represent known occurrences of bird species through broad polygons coded according to season: 'breeding', 'nonbreeding', 'passage' or 'resident'. First, from the list of species covered by the AEMLAP and the Raptors MOU, we selected those with 'breeding' and/or 'resident' occurrence in Europe and 'nonbreeding' and/or 'resident' occurrence in sub-Saharan Africa. Second, we selected only those species that perform migratory movements between Europe and sub-Saharan Africa, following Walther (2005). This selection process resulted in a final list encompassing 118 species of long-distance migratory species, of which 91 are landbirds and 27 are raptors (see below Table S2).

We used the European Red List of Birds (BirdLife International, 2021) to extract European population trend for all species analyzed, categorized into 'decreasing' or 'other' (including all other categories: stable, increasing, fluctuating and unknown).

Table S2. List of the 118 species of African-Eurasian long-distance migratory landbirds and raptors analyzed. Common name, scientific name and synonyms as used in the literature reviews search strings (details in Appendix S3). European population trend ('decreasing', 'other') according the European Red List of Birds (BirdLife International, 2021). Taxonomy follows the Handbook of the Birds of the World and BirdLife International's checklist (version 5; http://datazone.birdlife.org/species/taxonomy).

Group	Family	Common name	Scientific name	Synonym	Agreement	European population trend
landbirds	Phasianidae	Common quail	*Coturnix coturnix*		AEMLAP	decreasing
landbirds	Columbidae	European turtle-dove	*Streptopelia turtur*		AEMLAP	decreasing
landbirds	Caprimulgidae	Red-necked nightjar	*Caprimulgus ruficollis*		AEMLAP	decreasing
landbirds	Caprimulgidae	European nightjar	*Caprimulgus europaeus*		AEMLAP	other
landbirds	Apodidae	Alpine swift	*Tachymarptis melba*	*Apus melba*	AEMLAP	other
landbirds	Apodidae	White-rumped swift	*Apus caffer*		AEMLAP	other
landbirds	Apodidae	Little swift	*Apus affinis*		AEMLAP	decreasing
landbirds	Apodidae	Pallid swift	*Apus pallidus*		AEMLAP	other
landbirds	Apodidae	Common swift	*Apus apus*		AEMLAP	decreasing
landbirds	Cuculidae	Great spotted cuckoo	*Clamator glandarius*		AEMLAP	decreasing
landbirds	Cuculidae	Common cuckoo	*Cuculus canorus*		AEMLAP	decreasing
landbirds	Burhinidae	Eurasian thick-knee	*Burhinus oedicnemus*		AEMLAP	decreasing
landbirds	Glareolidae	Cream-colored courser	*Cursorius cursor*		AEMLAP	other
landbirds	Upupidae	Common hoopoe	*Upupa epops*		AEMLAP	other
landbirds	Meropidae	Blue-cheeked bee-eater	*Merops persicus*		AEMLAP	other
landbirds	Meropidae	European bee-eater	*Merops apiaster*		AEMLAP	other
landbirds	Coraciidae	European roller	*Coracias garrulus*		AEMLAP	decreasing
landbirds	Picidae	Eurasian wryneck	*Jynx torquilla*		AEMLAP	other
landbirds	Oriolidae	Eurasian golden oriole	*Oriolus oriolus*		AEMLAP	other
landbirds	Laniidae	Red-backed shrike	*Lanius collurio*		AEMLAP	decreasing
landbirds	Laniidae	Lesser grey shrike	*Lanius minor*		AEMLAP	decreasing
landbirds	Laniidae	Great grey shrike	*Lanius excubitor*	*Lanius meridionalis*	AEMLAP	decreasing
landbirds	Laniidae	Woodchat shrike	*Lanius senator*		AEMLAP	decreasing
landbirds	Laniidae	Masked shrike	*Lanius nubicus*		AEMLAP	other
landbirds	Alaudidae	Bimaculated lark	*Melanocorypha bimaculata*		AEMLAP	other
landbirds	Alaudidae	Greater short-toed lark	*Calandrella brachydactyla*		AEMLAP	other
landbirds	Acrocephalidae	Olivaceous warbler	*Iduna pallida*	*Hippolais pallida*	AEMLAP	other
landbirds	Acrocephalidae	Isabelline warbler	*Iduna opaca*	*Hippolais opaca*	AEMLAP	other
landbirds	Acrocephalidae	Upcher's warbler	*Hippolais languida*		AEMLAP	other
landbirds	Acrocephalidae	Olive-tree warbler	*Hippolais olivetorum*		AEMLAP	other
landbirds	Acrocephalidae	Melodious warbler	*Hippolais polyglotta*		AEMLAP	other
landbirds	Acrocephalidae	Icterine warbler	*Hippolais icterina*		AEMLAP	decreasing
landbirds	Acrocephalidae	Aquatic warbler	*Acrocephalus paludicola*		AEMLAP	decreasing

landbirds	Acrocephalidae	Sedge warbler	*Acrocephalus schoenobaenus*		AEMLAP	other
landbirds	Acrocephalidae	Marsh warbler	*Acrocephalus palustris*		AEMLAP	other
landbirds	Acrocephalidae	Common reed-warbler	*Acrocephalus scirpaceus*		AEMLAP	other
landbirds	Acrocephalidae	Great reed-warbler	*Acrocephalus arundinaceus*		AEMLAP	other
landbirds	Locustellidae	Savi's warbler	*Locustella luscinioides*		AEMLAP	other
landbirds	Locustellidae	River warbler	*Locustella fluviatilis*		AEMLAP	decreasing
landbirds	Locustellidae	Common grasshopper-warbler	*Locustella naevia*		AEMLAP	decreasing
landbirds	Hirundinidae	Northern house martin	*Delichon urbicum*	*Delichon urbica*	AEMLAP	other
landbirds	Hirundinidae	Red-rumped swallow	*Cecropis daurica*	*Hirundo daurica*	AEMLAP	other
landbirds	Hirundinidae	Barn swallow	*Hirundo rustica*		AEMLAP	decreasing
landbirds	Hirundinidae	Eurasian crag martin	*Ptyonoprogne rupestris*	*Hirundo rupestris*	AEMLAP	other
landbirds	Hirundinidae	Collared sand martin	*Riparia riparia*		AEMLAP	decreasing
landbirds	Phylloscopidae	Eastern Bonelli's warbler	*Phylloscopus orientalis*		AEMLAP	other
landbirds	Phylloscopidae	Western Bonelli's warbler	*Phylloscopus bonelli*		AEMLAP	other
landbirds	Phylloscopidae	Wood warbler	*Phylloscopus sibilatrix*		AEMLAP	decreasing
landbirds	Phylloscopidae	Willow warbler	*Phylloscopus trochilus*		AEMLAP	decreasing
landbirds	Phylloscopidae	Iberian chiffchaff	*Phylloscopus ibericus*	*Phylloscopus brehmii*	AEMLAP	other
landbirds	Phylloscopidae	Common chiffchaff	*Phylloscopus collybita*		AEMLAP	other
landbirds	Sylviidae	Eurasian blackcap	*Sylvia atricapilla*		AEMLAP	other
landbirds	Sylviidae	Garden warbler	*Sylvia borin*		AEMLAP	decreasing
landbirds	Sylviidae	Barred warbler	*Sylvia nisoria*		AEMLAP	other
landbirds	Sylviidae	Western orphean warbler	*Sylvia hortensis*		AEMLAP	other
landbirds	Sylviidae	Eastern orphean warbler	*Sylvia crassirostris*	*Sylvia hortensis*	AEMLAP	other
landbirds	Sylviidae	Lesser whitethroat	*Sylvia curruca*		AEMLAP	other
landbirds	Sylviidae	Menetries's warbler	*Sylvia mystacea*		AEMLAP	other
landbirds	Sylviidae	Subalpine warbler	*Sylvia cantillans*		AEMLAP	other
landbirds	Sylviidae	Moltoni's warbler	*Sylvia subalpina*	*Sylvia cantillans*	AEMLAP	other
landbirds	Sylviidae	Rüppell's warbler	*Sylvia ruppeli*	*Sylvia rueppelli*	AEMLAP	other
landbirds	Sylviidae	Common whitethroat	*Sylvia communis*		AEMLAP	other
landbirds	Muscicapidae	Rufous-tailed scrub-robin	*Cercotrichas galactotes*	*Erythropygia galactotes*	AEMLAP	decreasing
landbirds	Muscicapidae	Spotted flycatcher	*Muscicapa striata*		AEMLAP	decreasing
landbirds	Muscicapidae	White-throated robin	*Irania gutturalis*		AEMLAP	other
landbirds	Muscicapidae	Bluethroat	*Cyanecula svecica*	*Luscinia svecica*	AEMLAP	other
landbirds	Muscicapidae	Thrush nightingale	*Luscinia luscinia*		AEMLAP	other
landbirds	Muscicapidae	Common nightingale	*Luscinia megarhynchos*		AEMLAP	other
landbirds	Muscicapidae	Semi-collared flycatcher	*Ficedula semitorquata*		AEMLAP	other
landbirds	Muscicapidae	European pied flycatcher	*Ficedula hypoleuca*		AEMLAP	decreasing
landbirds	Muscicapidae	Collared flycatcher	*Ficedula albicollis*		AEMLAP	other
landbirds	Muscicapidae	Common redstart	*Phoenicurus phoenicurus*		AEMLAP	other
landbirds	Muscicapidae	Rufous-tailed rock-thrush	*Monticola saxatilis*		AEMLAP	other
landbirds	Muscicapidae	Blue rock-thrush	*Monticola solitarius*		AEMLAP	other
landbirds	Muscicapidae	Whinchat	*Saxicola rubetra*		AEMLAP	decreasing

landbirds	Muscicapidae	Common stonechat	*Saxicola torquatus*	*Saxicola torquata*	AEMLAP	decreasing
landbirds	Muscicapidae	Northern wheatear	*Oenanthe oenanthe*		AEMLAP	other
landbirds	Muscicapidae	Isabelline wheatear	*Oenanthe isabellina*		AEMLAP	other
landbirds	Muscicapidae	Desert wheatear	*Oenanthe deserti*		AEMLAP	other
landbirds	Muscicapidae	Black-eared wheatear	*Oenanthe hispanica*		AEMLAP	other
landbirds	Muscicapidae	Cyprus wheatear	*Oenanthe cypriaca*		AEMLAP	other
landbirds	Muscicapidae	Pied wheatear	*Oenanthe pleschanka*		AEMLAP	other
landbirds	Muscicapidae	Kurdish wheatear	*Oenanthe xanthoprymna*		AEMLAP	other
landbirds	Motacillidae	Tree pipit	*Anthus trivialis*		AEMLAP	decreasing
landbirds	Motacillidae	Red-throated pipit	*Anthus cervinus*		AEMLAP	other
landbirds	Motacillidae	Tawny pipit	*Anthus campestris*		AEMLAP	other
landbirds	Motacillidae	Western yellow wagtail	*Motacilla flava*		AEMLAP	decreasing
landbirds	Motacillidae	Grey wagtail	*Motacilla cinerea*		AEMLAP	other
landbirds	Motacillidae	White wagtail	*Motacilla alba*		AEMLAP	other
landbirds	Emberizidae	Cinereous bunting	*Emberiza cineracea*		AEMLAP	decreasing
landbirds	Emberizidae	Ortolan bunting	*Emberiza hortulana*		AEMLAP	decreasing
raptors	Strigidae	Eurasian scops-owl	*Otus scops*		raptors-MOU	other
raptors	Strigidae	Short-eared owl	*Asio flammeus*		raptors-MOU	other
raptors	Pandionidae	Osprey	*Pandion haliaetus*		raptors-MOU	other
raptors	Accipitridae	European honey-buzzard	*Pernis apivorus*		raptors-MOU	other
raptors	Accipitridae	Egyptian vulture	*Neophron percnopterus*		raptors-MOU	decreasing
raptors	Accipitridae	Short-toed snake-eagle	*Circaetus gallicus*		raptors-MOU	other
raptors	Accipitridae	Griffon vulture	*Gyps fulvus*		raptors-MOU	other
raptors	Accipitridae	Lesser spotted eagle	*Clanga pomarina*	*Aquila pomarina*	raptors-MOU	other
raptors	Accipitridae	Greater spotted eagle	*Clanga clanga*	*Aquila clanga*	raptors-MOU	decreasing
raptors	Accipitridae	Steppe eagle	*Aquila nipalensis*		raptors-MOU	decreasing
raptors	Accipitridae	Eastern imperial eagle	*Aquila heliaca*		raptors-MOU	other
raptors	Accipitridae	Booted eagle	*Hieraaetus pennatus*	*Aquila pennata*	raptors-MOU	other
raptors	Accipitridae	Western marsh-harrier	*Circus aeruginosus*		raptors-MOU	other
raptors	Accipitridae	Pallid harrier	*Circus macrourus*		raptors-MOU	other
raptors	Accipitridae	Montagu's harrier	*Circus pygargus*		raptors-MOU	decreasing
raptors	Accipitridae	Levant sparrowhawk	*Accipiter brevipes*		raptors-MOU	other
raptors	Accipitridae	Eurasian sparrowhawk	*Accipiter nisus*		raptors-MOU	other
raptors	Accipitridae	Black kite	*Milvus migrans*		raptors-MOU	other
raptors	Accipitridae	Eurasian buzzard	*Buteo buteo*		raptors-MOU	other
raptors	Accipitridae	Long-legged buzzard	*Buteo rufinus*		raptors-MOU	other
raptors	Falconidae	Lesser kestrel	*Falco naumanni*		raptors-MOU	other
raptors	Falconidae	Common kestrel	*Falco tinnunculus*		raptors-MOU	decreasing
raptors	Falconidae	Red-footed falcon	*Falco vespertinus*		raptors-MOU	decreasing
raptors	Falconidae	Eleonora's falcon	*Falco eleonorae*		raptors-MOU	other
raptors	Falconidae	Eurasian hobby	*Falco subbuteo*		raptors-MOU	other
raptors	Falconidae	Saker falcon	*Falco cherrug*		raptors-MOU	decreasing
raptors	Falconidae	Peregrine falcon	*Falco peregrinus*		raptors-MOU	other

Appendix S3: Review of studies

Steps followed to identify the studies used as sources of bird migration records

We aimed to compile migration records from as many landbird and raptor populations as possible. Therefore, we first conducted a review of tracking studies from the peer-reviewed literature, which we complemented with information from other studies (workflow in Fig. S3).

A. Review of published studies

We conducted a review of published, peer-reviewed studies in ISI Web of Science core collection (https://www.webofknowledge.com/) and Google Scholar (considering up to the first 20 results; https://scholar.google.com/), on 29-30 April 2021. For each species analyzed, we used the search string [(*common name* OR *scientific name* OR *synonyms*) AND migra* AND (tracking OR gps OR gsm OR ptt OR geoloc* OR light OR logger OR satellite)], where the *common name*, *scientific name* and *synonyms* terms varied in each search (Appendix S2). We merged the results obtained from the two databases (ISI Web of Science, 776 studies; Google Scholar, 826) to obtain a list of 1496 unique published studies.

We then screened each of these studies to identify those among them that met our three inclusion criteria: (1) reported results of a tracking study, (2) of at least one of our study species (i.e., long-distance migratory African-Eurasian landbirds or raptors; Appendix S2), (3) including at least one migration record of a bird breeding in Europe and spending the nonbreeding season in Sub-Saharan Africa. We first screened each of the studies by reading the title and, if inconclusive, the abstract to highlight the most promising ones based on the first two criteria. We then read the full text of the promising studies to verify if it contained in the text, tables or figures migration records of individuals migrating between a breeding country in Europe and a nonbreeding country in sub-Saharan Africa. We excluded one study where birds were tagged during migration and for which it was not possible to ascertain what their breeding country was (Thorup et al., 2020). Having found very few studies tracking birds from Africa (n = 3; Meyburg et al., 2001; Blackburn et al., 2017, 2019) we opted not to include them to avoid over-complicating the methods and interpretation of the results. This yielded 173 eligible studies.

Finally, we screened the 173 studies to identify those that were redundant in terms of data (e.g., studies that used the same tracking dataset to address different ecological questions). This screening was done by carefully comparing studies tracking the same species from the same countries, with studies using the same tracking data identified based on the authors of the studies, the site where birds were tagged, the identity of the individuals tracked (i.e., code used by the authors) and the number of tracked individuals. Whenever we found studies that had used the same tracking data, we selected the one among them with migration records available for a larger number of individuals. This yielded 81 studies with unique data.

B. Complementary searches

We performed complementary searches for studies (including gray literature) that could add new information to those identified through the review of peer-reviewed publications. First, we added 10 studies (1 technical report and nine papers) that had been referenced by previous reviews (Finch et al., 2017; Briedis et al., 2019, 2020; Brlík et al., 2020; Cresswell et al., 2020). Second, we identified additional studies through extensive *ad hoc* searches on Google (completed on 30 April 2021), using the same type of keywords as above (i.e., species' common and scientific name, migra*, and type of tracking device). These searches were likely biased towards studies in English, although the use of the scientific name and of the tracking devices allowed us to find studies in other languages (e.g., Spanish, German, French, Polish). These searches yielded an additional set of 41 studies, including one book, one magazine article, one thesis, two technical reports, two conference posters, five peer-reviewed papers and 29 other studies gathered from 16 websites. Regarding the websites, we considered for analytical purposes (i.e., to produce Figure 1) that: each tracked species in each website corresponds to a different study; and, that the study year corresponded to the year the first bird was tracked (given that the dates the tracks were made available were not readily available from the websites).

The 51 studies added through these searches were all complementary to those obtained through the review of scientific literature databases, i.e., each one of them added a different set of data.

We obtained a final list of 132 studies (81 obtained from literature review and 51 from complementary searches; Fig. S3), from which we gathered 1282 migration records (Appendix S4). The final dataset analyzed (excluding

populations with < 3 migration records), comprised 1229 migration records covering 43 bird species (29 landbirds, 14 raptors), belonging to 123 populations. The data set is available from Guilherme (2022).

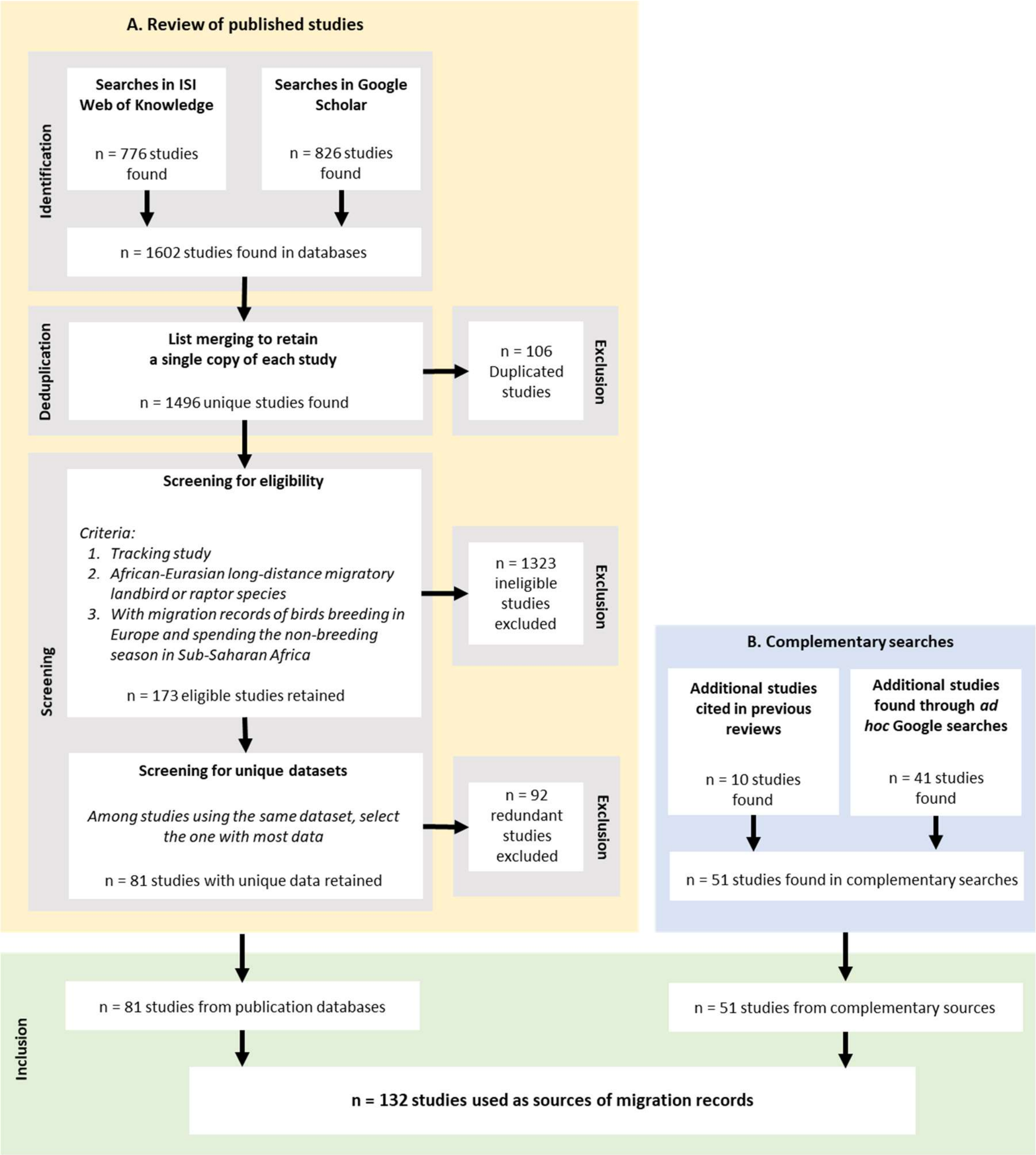

Figure S3. Flowchart illustrating the steps followed to identify the studies used as sources of bird migration records, showing the number of studies at each step.

Appendix S4: Migration records compiled

Details on the migration records obtained from the identified studies

From each study obtained in the literature review (Appendix S3), we extracted as many migration records as possible. Each migration record corresponds to a single individual bird, and includes the following:

- Species
- Breeding country; i.e., the country in Europe where the bird was tagged during the breeding season
- Nonbreeding country, i.e., the country in sub-Saharan African where the bird stayed the longest

We extracted this information preferably from text and tables. When not explicitly reported, we estimated locations from the plotted maps (e.g., through density of positions). In cases where it was unclear which of several nonbreeding countries should be considered as the most important, we selected the southernmost country (e.g., Eurasian cuckoo [*Cuculus canorus*]; Hewson et al., 2016). This said, we excluded common and pallid swifts (*Apus apus* and *A. pallidus*) because individuals of these highly-mobile species cover vast areas crossing multiple countries during their stay in Sub-Saharan Africa (Klaassen et al., 2014; Wellbrock et al., 2017; Jacobsen et al., 2017; Norevik et al., 2019b, 2019a; Åkesson et al., 2020), and it was not appropriate to pinpoint a single country where each tracked bird spent the majority of its nonbreeding season.

For birds tracked over multiple years, we only considered the first complete migration reported. We excluded individuals whose nonbreeding country was not possible to extract (e.g., location was obscured in the study's map); this resulted in 13 records excluded, representing 1% of the final migration records obtained (see below; Table S4). We also excluded records whose nonbreeding geographical positions (i.e., longitude/latitude) reported in the studies were >1000 km outside of the known nonbreeding range of the species and in a country where the species is not known to occur following (BirdLife International and Handbook of the Birds of the World 2018), as we considered these likely to correspond to vagrant individuals (n = 3 records excluded, 0.2%) (see below; Table S4). Finally, we excluded from the analyses populations with fewer than three migration records. The data set is available from Guilherme (2022).

Table S4. List of the migration records obtained from the identified studies. The data set is available from Guilherme (2022).

Species	Population	No. migration records extracted from study	No. migration records compiled per population	Tracking device	Population included in analysis (Y/N)	Tracks published with study (Y/N)	Study type	Reference
**European turtle-dove** (*Streptopelia turtur*)	France	5	18	GLS	Y	Y	journal article	Eraud et al., 2013
		2		PTT		N	journal article	Lormee et al., 2016
		11		PTT		N	website	ONCFS, 2016
	Germany	4	4	PTT	Y	N	website	NABU, 2019
	UK	4	4	PTT	Y	N	website	RSPB, 2014
**European nightjar** (*Caprimulgus europaeus*)	Belgium	4	4	GLS/GPS	Y	N	journal article	Evens et al., 2017
	Denmark	6	6	GLS	Y	Y	journal article	Jacobsen et al., 2017
	France	1	1	GLS	N	N	journal article	Evens et al., 2017
	Sweden	28	28	GLS/GPS	Y	N	journal article	Norevik et al., 2019a
	UK	5	12	GLS	Y	N	journal article	Evens et al., 2017
		7		GPS		N	website	BTO, 2018
**alpine swift** (*Tachymarptis melba*)	Switzerland	3	3	GLS	Y	N	journal article	Liechti et al., 2013
**white-rumped swift** (*Apus caffer*)	Spain	3	3	GLS	Y	N	journal article	Vega et al., 2019
**pallid swift** (*Apus pallidus*) [(1)]	Italy	-	-	GLS	N	N	journal article	Norevik et al., 2019b
	Switzerland	-	-	GLS	N	N	journal article	Norevik et al., 2019b
**common swift** (*Apus apus*) [(1)]	Denmark	-	-	GLS	N	N	journal article	Jacobsen et al., 2017
	Germany	-	-	GLS	N	N	journal article	Wellbrock et al., 2017
	Netherlands	-	-	GLS	N	N	journal article	Klaassen et al., 2014
	Sweden	-	-	GLS	N	N	journal article	Åkesson et al., 2020
**great spotted cuckoo** (*Clamator glandarius*)	Spain	3	3	PTT	Y	Y	journal article	Ibáñez-Álamo et al., 2019
**common cuckoo** (*Cuculus canorus*)	Denmark	3	3	GLS	Y	Y	journal article	Willemoes et al., 2014
	Finland	2	2	PTT	N	Y	journal article	Vega et al., 2016

	Hungary	3	3	GPS	Y	N	journal article	Bán et al., 2018
	Sweden	3	3	PTT	Y	Y	journal article	Willemoes et al., 2014
	UK	25	25	PTT	Y	N	journal article	Hewson et al., 2016
**osprey** (*Pandion haliaetus*)	Belarus	2	2	GPS	N	N	website	Kotkaklubi, 2016
	Estonia	4	6	GPS	Y	N	journal article	Väli et al., 2016
		2		GPS		N	website	Kotkaklubi, 2016
	Finland	18	18	GPS	Y	N	website	LUOMUS, 2017
	Germany	12	12	GPS	Y	N	conference poster	Meyburg et al., 2011c
	Latvia	1	1	GPS	N	N	website	Kotkaklubi, 2016
	Norway	5	5	GPS	Y	N	journal article	Østnes et al., 2019
	Spain	1	1	GPS	N	N	journal article	Monti et al., 2018
	Sweden	7	20	GPS	Y	N	journal article	Monti et al., 2018
		13		PTT		N	journal article	Hake et al., 2001
	UK	12	21	GPS	Y	N	thesis	Mackrill, 2017
		9		GPS		N	website	RSPB, 2008
**European honey-buzzard** (*Pernis apivorus*)	Finland	22	22	GPS	Y **(2)**	Y	journal article	Vansteelant et al., 2017
	Germany	9	9	GPS	Y	N	conference poster	Meyburg et al., 2011a
	Hungary	1	1	GPS	N	N	journal article	Agostini et al., 2019
	Netherlands	12	12	GPS	Y	Y	journal article	Vansteelant et al., 2017
	Poland	4	4	GPS	Y	N	website	KOO, 2019
	Spain	1	1	GPS	N	N	website	SEO Birdlife, 2012a
	Sweden	8	8	PTT	Y	N	journal article	Hake et al., 2003
	UK	4	4	PTT	Y	N	website	Roy Dennis Wildlife Foundation, 2003
**Egyptian vulture** (*Neophron percnopterus*)	Bulgaria **(3)**	13	13	GPS	Y	N	journal article	Phipps et al., 2019
	France	2	3	PTT	Y	N	journal article	Meyburg et al., 2004a
		1		GPS		N	website	VCF, 2017
	Portugal	6	6	GPS	Y	N	website	VCF, 2017

	Spain	20	20	GPS	Y	N	journal article	Phipps et al., 2019
	Turkey	9	9	GPS	Y	N	journal article	Phipps et al., 2019
**Short-toed snake-eagle** (*Circaetus gallicus*)		1		PTT		N	journal article	Meyburg et al., 1998
	France	1	3	PTT	Y	N	journal article	Meyburg et al., 1996
		1		GPS		N	journal article	Jiguet et al., 2012
	Hungary	2	2	GPS	N	N	website	MME Birdlife Hungary, 2018
	Italy	5	5	GPS	Y	N	journal article	Mellone et al., 2016
	Spain	2	5	GPS	Y	N	journal article	Pavón et al., 2010
		3		GPS		N	website	SEO Birdlife, 2008
**Griffon vulture** (*Gyps fulvus*)	Spain	1	1	GPS	N	N	conference paper	Muñoz et al., 2016
	Bulgaria	1	1	GPS	N	N	journal article	Arkumarev et al., 2019
**Lesser spotted eagle** (*Clanga pomarina*)	Estonia	8	8	GPS	Y	N	journal article	Väli et al., 2018
		2		GPS		N	journal article	Meyburg et al., 2009
	Germany	3	17	PTT	Y	N	journal article	Meyburg et al., 2008
		12		GPS		N	website	WWGBP, 2013
	Hungary	2	2	GPS	N	N	website	MME Birdlife Hungary, 2016
	Poland	4	4	GPS	Y	N	journal article	Väli et al., 2018
	Romania	7	7	PTT	Y	N	website	Milvus Group, 2010
	Slovakia	1	3	PTT	Y	N	journal article	Meyburg et al., 2004b
		2		PTT		N	journal article	Meyburg et al., 2000
**Greater spotted eagle** (*Clanga clanga*)	Belarus	2	2	GPS	N	N	website	Kotkaklubi, 2018
	Poland	8	8	GPS	Y	N	journal article	Väli et al., 2018
**Eastern imperial eagle** (*Aquila heliaca*)	Bulgaria	2	2	GPS	N	N	website	BSPB, 2013
**Booted eagle** (*Hieraaetus pennatus*)	Spain	14	14	PTT/GPS	Y	N	book	Bermejo et al., 2017
**Western marsh-harrier** (*Circus aeruginosus*)	Belgium	4	4	GPS	Y	Y	journal article	Vansteelant et al., 2020
	Czech Republic	4	4	GPS	Y	N	website	Bird Telemetry, 2020
	Germany	3	3	GPS	Y	N	website	GKA, 2020

	Hungary	3	3	GPS	Y	N	website	MME Birdlife Hungary, 2014
	Netherlands	2	4	GPS	Y	Y	journal article	Vansteelant et al., 2020
		2		GPS		N	website	GKA, 2020
	Slovakia	6	6	GPS	Y	N	website	MME Birdlife Hungary, 2014
	Sweden	8	12	PTT/GPS	Y	N	journal article	Strandberg et al., 2008
		4		GPS		N	journal article	Vansteelant et al., 2020
	UK	1	1	PTT	N	N	website	Roy Dennis Wildlife Foundation, 2004
**Pallid harrier** (*Circus macrourus*)	Finland	2	2	GPS	N	-	website	LUOMUS, 2019
**Montagu's harrier** (*Circus pygargus*)	Belarus	3	3	PTT	Y **(2)**	Y	journal article	Trierweiler et al., 2014
	Denmark	6	6	PTT	Y	Y	journal article	Trierweiler et al., 2014
	Germany	4	4	PTT	Y **(4)**	Y	journal article	Trierweiler et al., 2014
	Netherlands	11	11	PTT	Y **(5)**	Y	journal article	Trierweiler et al., 2014
	Poland	3	3	PTT	Y	Y	journal article	Trierweiler et al., 2014
	Spain	6	6	PTT	Y	N	journal article	Limiñana et al., 2007
	UK	4	4	PTT	Y	N	website	RSPB, 2016
**Black kite** (*Milvus migrans*)	Bulgaria	3	3	GPS	Y	N	website	Bird Telemetry, 2018
	Czech Republic	1	8	GPS	Y	N	journal article	Literák et al., 2020
		7		GPS		N	website	Bird Telemetry, 2018
	Germany	1	2	PTT	N	N	journal article	Meyburg and Meyburg 2009
		1		GPS		N	journal article	Ovčiariková et al., 2020
	Slovakia	1	2	GPS	N	N	website	Bird Telemetry, 2018
		1		GPS		N	journal article	Ovčiariková et al., 2020
	Spain	4	4	GPS	Y	N	journal article	Tanferna et al., 2012
	Ukraine	1	1	GPS	N	N	website	Bird Telemetry, 2018
**Common hoopoe** (*Upupa epops*)	Czech Republic	4	4	GLS	Y	N	journal article	van Wijk et al., 2018
	Germany	10	10	GLS	Y	N	journal article	van Wijk et al., 2018

	Spain	2	2	GLS	Y [2]	N	journal article	van Wijk et al., 2018
	Switzerland	3	20	GLS	Y2	N	journal article	Bächler et al., 2010
		17		GLS		N	journal article	van Wijk et al., 2018
**European bee-eater** (*Merops apiaster*)	Bulgaria	6	6	GLS	Y	N	journal article	Hahn et al., 2020
	Germany	17	17	GLS	Y [2]	N	journal article	Hahn et al., 2020
	Portugal	5	5	GLS	Y	N	journal article	Hahn et al., 2020
**European roller** (*Coracias garrulus*)	Austria	1	1	GLS	N	Y	journal article	Finch et al., 2015
	Cyprus	1	1	GLS	N	Y	journal article	Finch et al., 2015
	France	9	9	GLS	Y	Y	journal article	Finch et al., 2015
	Hungary	7	7	PTT	Y	N	website	MME Birdlife Hungary, 2015
	Latvia	4	4	GLS	Y	Y	journal article	Finch et al., 2015
	Montenegro	3	3	GLS	Y	Y	journal article	Finch et al., 2015
	Portugal	4	4	GLS	Y	Y	journal article	Finch et al., 2015
	Spain	10	10	GLS/PTT	Y	Y	journal article	Finch et al., 2015
**Lesser kestrel** (*Falco naumanni*)	Bulgaria	5	5	PTT	Y	N	journal article	Sarà et al., 2019
	France	13	14	GLS	Y	N	journal article	Pilard et al., 2017
		1		GLS		N	journal article	Sarà et al., 2019
	Greece	6	6	GLS	Y	N	journal article	Sarà et al., 2019
	Italy	35	35	GLS/PTT/GPS	Y	N	journal article	Sarà et al., 2019
	Portugal	4	4	GLS	Y	N	journal article	Catry et al., 2011
	Spain	5	24	PTT	Y	N	journal article	Limiñana et al., 2012
		3		GLS		N	journal article	Rodríguez et al., 2009
		16		PTT/GPS		N	journal article	Sarà et al., 2019
**Red-footed falcon** (*Falco vespertinus*) [7]	Hungary	8	8	PTT	Y	N	technical report / website	Softpro Kft., 2016 / MME Birdlife Hungary, 2009
	Italy	1	1	PTT	N	N	technical report / website	Softpro Kft., 2016 / MME Birdlife Hungary, 2009

**Eleonora's falcon** (*Falco eleonorae*)	Croatia	2	2	PTT	N	N	journal article	Kassara et al., 2017
	Cyprus	6	6	PTT/GPS	Y (2)	N	journal article	Hadjikyriakou et al., 2020a
	Greece	4	8	PTT	Y (6)	N	journal article	Kassara et al., 2012
		4		PTT		N	journal article	Hadjikyriakou et al., 2020b
	Italy	9	9	PTT	Y	N	journal article	Gschweng et al., 2008
	Spain	7	7	PTT	Y	N	journal article	Mellone et al., 2013
**Eurasian hobby** (*Falco subbuteo*)	Germany	1	1	PTT	N	N	journal article	Meyburg et al., 2011b
	Sweden	3	3	PTT	Y	N	journal article	Strandberg et al., 2009
	UK	1	1	PTT	N	N	website	Roy Dennis Wildlife Foundation, 2010
**Red-backed shrike** (*Lanius collurio*)	Denmark	13	14	GLS	Y	Y	journal article	Thorup et al., 2017
		1		GPS		N	journal article	Pedersen et al., 2019
	Greece	3	3	GLS	Y	Y	journal article	Pedersen et al., 2020
	Netherlands	3	3	GLS	Y	Y	journal article	Pedersen et al., 2020
	Spain	6	6	GLS	Y	Y	journal article	Tøttrup et al., 2017
	Sweden	5	5	GLS	Y (6)	Y	journal article	Thorup et al., 2017
**Aquatic warbler** (*Acrocephalus paludicola*)	Belarus	2	2	GLS	N	N	journal article	Salewski et al., 2018
	Ukraine	4	4	GLS	Y	N	journal article	Salewski et al., 2018
**Common reed-warbler** (*Acrocephalus scirpaceus*)	Czech Republic	9	9	GLS	Y	N	journal article	Procházka et al., 2018
	Germany	6	6	GLS	Y	N	journal article	Procházka et al., 2018
**Great reed-warbler** (*Acrocephalus arundinaceus*)	Bulgaria	17	17	GLS	Y	N	journal article	Koleček et al., 2018
	Czech Republic	29	29	GLS	Y	N	journal article	Koleček et al., 2018
	Spain	3	3	GLS	Y	N	journal article	Koleček et al., 2016
	Sweden	9	17	GLS	Y	N	journal article	Koleček et al., 2016
		8		GLS		N	journal article	Lemke et al., 2013
	Turkey	5	9	GLS	Y	N	journal article	Horns et al., 2016
		4		GLS		N	journal article	Koleček et al., 2016
**Northern house martin** (*Delichon urbicum*)	Hungary	5	5	GLS	Y	N	journal article	Szép et al., 2017

**Barn swallow** (*Hirundo rustica*)	Czech Republic	19	19	GLS	Y (5)	Y	journal article	Klvaňa et al., 2018
	Italy	46	46	GLS	Y	N	journal article	Liechti et al., 2015
	Lithuania	6	6	GLS	Y	N	journal article	Briedis et al., 2018a
	Spain	4	41	GLS	Y	N	journal article	Arizaga et al., 2015
		35		GLS		N	journal article	López-Calderón et al., 2021
		2		GLS		N	website	SEO Birdlife, 2012b
	Switzerland	44	44	GLS	Y	N	journal article	Liechti et al., 2015
**Collared sand martin** (*Riparia riparia*)	Hungary	4	6	GLS	Y	N	journal article	Szép et al., 2017
		2		GLS		N	journal article	Hahn et al., 2021
	Germany	2	2	GLS	N	N	journal article	Hahn et al., 2021
**Wood warbler** (*Phylloscopus sibilatrix*)	Denmark	1	1	GLS	N	Y	journal article	Tøttrup et al., 2018
**Willow warbler** (*Phylloscopus trochilus*)	Denmark	14	14	GLS	Y	Y	journal article	Lerche-Jørgensen et al., 2017
**Rufous-tailed scrub-robin** (*Cercotrichas galactotes*)	Spain	3	3	GLS	Y	N	journal article	Vega et al., 2019
**Bluethroat** (*Cyanecula svecica*)	Spain	2	2	GLS	N	N	journal article	Vega et al., 2019
**Thrush nightingale** (*Luscinia luscinia*)	Denmark	13	13	GLS	Y	Y	journal article	Thorup et al., 2017
	Sweden	2	2	GLS	N	N	journal article	Stach et al., 2012
**Common nightingale** (*Luscinia megarhynchos*)	Bulgaria	11	11	GLS	Y	N	journal article	Hahn et al., 2014
	France	11	11	GLS	Y	N	journal article	Hahn et al., 2014
	Italy	6	6	GLS	Y	N	journal article	Hahn et al., 2014
	UK	1	1	GLS	N	N	magazine article	Stancliffe, 2011
**Semi-collared flycatcher** (*Ficedula semitorquata*)	Bulgaria	11	11	GLS	Y	N	journal article	Briedis et al., 2016a
**European pied flycatcher** (*Ficedula hypoleuca*)	Finland	3	3	GLS	Y (6)	N	journal article	Ouwehand et al., 2016
	Netherlands	7	7	GLS	Y	N	journal article	Ouwehand et al., 2016
	Norway	1	1	GLS	N	N	journal article	Ouwehand et al., 2016
	UK	2	2	GLS	N	N	journal article	Ouwehand et al., 2016
**Collared flycatcher** (*Ficedula albicollis*)	Czech Republic	39	39	GLS	Y	Y	journal article	Briedis et al., 2018b

	Sweden	14	14	GLS	Y	N	journal article	Briedis et al., 2016b
**Common redstart** (*Phoenicurus phoenicurus*)	Denmark	5	5	GLS	Y	N	journal article	Kristensen et al., 2013
	Switzerland	3	3	GLS	Y	N	journal article	Gersten et al., 2016
**Whinchat** (*Saxicola rubetra*)	UK	20	20	GLS	Y	Y	journal article	Burgess et al., 2020
**Northern wheatear**(*Oenanthe oenanthe*)	Austria	1	1	GLS	N	Y	journal article	Schmaljohann et al., 2016
	Germany	6	6	GLS	Y	Y	journal article	Schmaljohann et al., 2016
	Netherlands	2	2	GLS	N	Y	journal article	Schmaljohann et al., 2016
	Sweden	14	14	GLS	Y	Y	journal article	Schmaljohann et al., 2016
	Switzerland	7	7	GLS	Y	Y	journal article	Schmaljohann et al., 2016
**Cyprus wheatear** (*Oenanthe cypriaca*)	Cyprus	13	13	GLS	Y	N	journal article	Patchett and Cresswell, 2020
**Tawny pipit** (*Anthus campestris*)	Czech Republic	6	12	GLS	Y	N	journal article	Briedis et al., 2016c
		6		GLS		N	journal article	Briedis et al., 2020
**Ortolan bunting** (*Emberiza hortulana*)	Belarus	1	1	GLS	N	N	technical report	Jiguet et al., 2019
	Germany	17	17	GLS	Y	N	technical report	Bernardy, 2016
	Sweden	7	7	GLS	Y	N	journal article	Selstam et al., 2015

(1) Highly-mobile species excluded from the dataset

(2) One individual reported in the study was excluded, as it was not possible to extract its nonbreeding country.

(3) Five individuals tagged in Greece were merged into Bulgaria, as it was not possible to separate them.

(4) Four individuals reported in the study were excluded, as it was not possible to extract their nonbreeding country.

(5) Two individuals reported in the study were excluded, as it was not possible to extract their nonbreeding country.

(6) One individual excluded as it was considered vagrant, as the reported nonbreeding geographical position (i.e., latitude/longitude) was >1000 km outside of the known nonbreeding range of the species and in a country where the species does not occur during the nonbreeding season.

(7) All birds tagged in Romania and two birds tagged in Hungary were excluded, because they were captured in post-breeding roosts (Softpro Kft., 2016) and their breeding country could not be confirmed.

Appendix S5: Updated nonbreeding range maps

Updated nonbreeding range maps for the European nightjar, European bee-eater, barn swallow and collared flycatcher

We obtained species distribution maps from BirdLife International and Handbook of the Birds of the World (2018), which correspond to coarse polygons encompassing known occurrences of the species coded according to season. In the particular cases of the European nightjar (*Caprimulgus europaeus*), Eurasian bee-eater (*Merops apiaster*), barn swallow (*Hirundo rustica*) and collared flycatcher (*Ficedula albicollis*), we concluded that the tracking data reveal nonbreeding ranges covering more countries than those identified in the range maps: European nightjar (BTO, 2018; Evens et al., 2017; Jacobsen et al., 2017; Norevik et al., 2017, 2019), European bee-eater (Dhanjal-Adams et al., 2018; Hahn et al., 2020); barn swallow (Arizaga et al., 2015; Briedis et al., 2018b; Klvaňa et al., 2018; Liechti et al., 2015; López-Calderón, 2019; SEO/BirdLife, 2012); and collared flycatcher (Briedis et al., 2016; Briedis et al., 2018a).

For these four species, we updated the nonbreeding ranges based on the geographical locations (i.e., longitude/latitude) of all individual birds reported in the studies. For each species, we determined the individuals' combined nonbreeding range using kernel density analyses, using a smoothing parameter of 200 km to account for the inherent error of the locations obtained with geolocators (~200 km; Lisovski et al., 2020). We chose the 80% kernel density isopleths pragmatically (through trial-and-error), as a compromise between larger isopleths delineating very large apparent ranges (increasing commission errors; i.e., species considered present when it is absent) and smaller isopleths delineating ranges with too convoluted boundaries (increasing omission errors; i.e., species considered absent when it is present).

The resulting areas were clipped with a land mask of Africa and merged with the original species' maps to obtain the nonbreeding ranges analyzed in this study (see below Fig. S5; shapefiles of the records used and the updated species maps are provided in Appendix S6). Analyses were performed using the packages adehabitatHR (Calenge, 2006) and sf (Pebesma, 2018) in R (R Core Team, 2021).

The new areas in the updated nonbreeding range maps obtained for the European nightjar (Central Africa), Eurasian bee-eater (Central-Western Africa and Western Africa) and barn swallow (Western Africa up to Senegal) closely match those mapped in Cramp (1985, 1988). A larger range was obtained for the collared flycatcher.

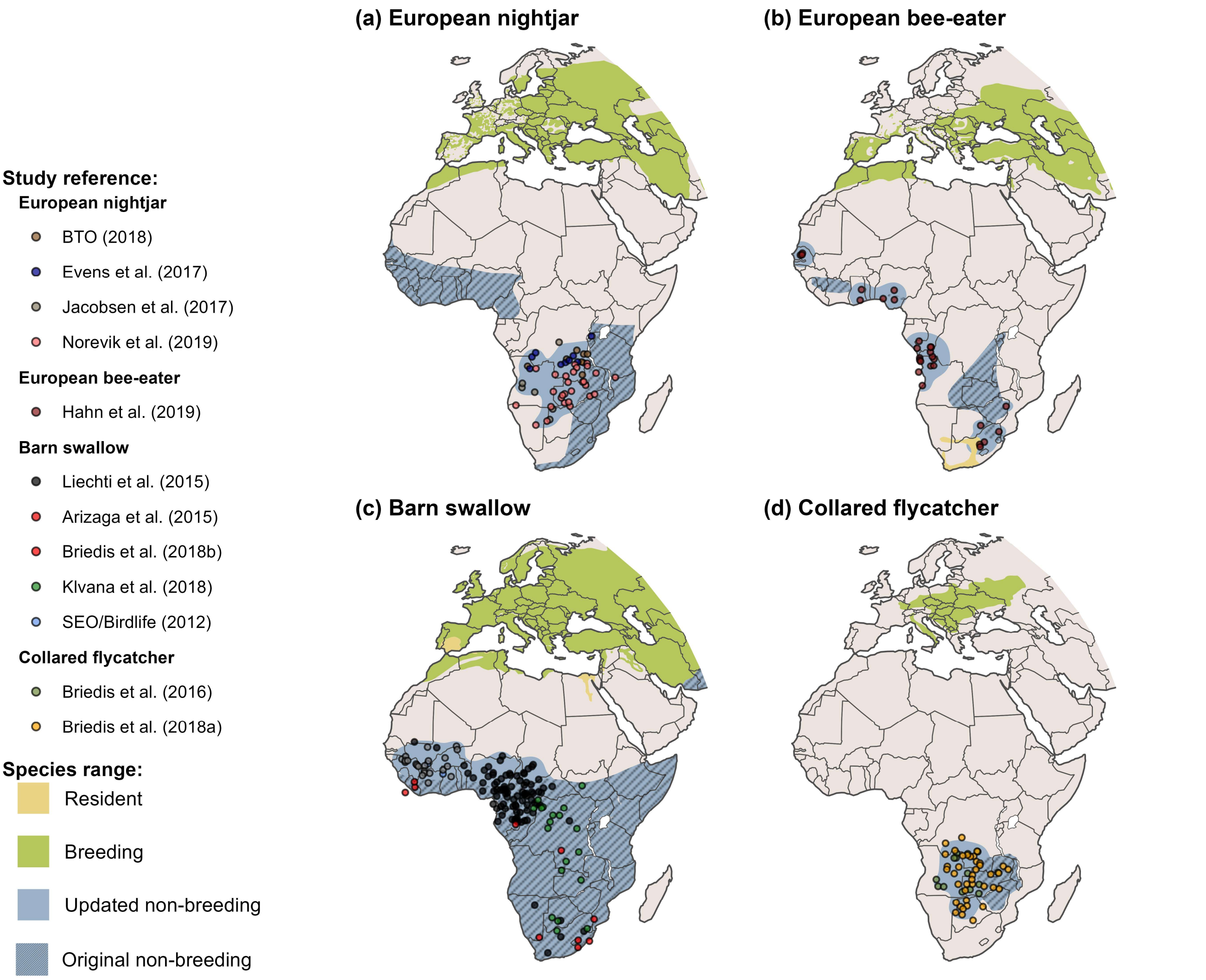

Figure S5. Updated nonbreeding range maps used in this study for: (a) European nightjar, (b) European bee-eater, (c) barn swallow, and (d) collared flycatcher - shapefiles in Appendix S6 (separate file)

Appendix S7. Estimation of migratory links' strength

Details and illustration of the method used for defining, for any given population, the nonbreeding range countries in sub-Saharan Africa and for estimating the strength of the migratory links

A. Details of the method

For each population represented by the migration records in our dataset, we identified one or more migratory links: one connecting to each of the sub-Saharan countries represented by the migration records. However, given the generally low number of migration records available, these 'observed links' (derived from observed records) certainly underestimate how many exist in reality for each population. Therefore, for each population, we attempted to infer migratory links not covered by our dataset.

First, we considered that for each observed migratory link, the specific nonbreeding location is the centroid of the nonbreeding range of the species within the focal African country (see example below, Fig. S7a). In the cases where the species mapped nonbreeding range in a given country is fragmented (e.g., nonbreeding range of the Egyptian Vulture [*Neophron percnopterus*] in Somalia; Appendix S11), we used the largest polygon of the species' nonbreeding range in the focal country. Second, we built from all these nonbreeding locations (for all observed links), to generate a minimum convex polygon (MCP; Fig. S7b). Seventeen populations in our dataset, had tracked individuals spending the nonbreeding season in disjunct areas in Africa (e.g., Osprey [*Pandion haliaetus*] breeding in Finland; Appendix S11 – separate file); therefore, for these populations, we considered different nonbreeding ranges (i.e., separated MCPs) whenever the closest nonbreeding geographical positions (i.e., longitude/latitude) reported in the studies were >1000 km apart and in noncontiguous countries. Third, we used this MCP to check for possible inferred links: all additional countries within the species' nonbreeding range that touch the MCP (Fig. S7c).

Then, we calculated the strength of each migratory link (observed or inferred), which represents the proportion of individuals in a population that spend the nonbreeding season in a given country in sub-Saharan Africa. The

strength can be estimated from the distribution of migration records among the migratory links in a population (akin to van Wijk et al., 2018; Morrick et al., 2021), but it requires estimating the number of expected records for any inferred links. We have done so by interpolating from the number of observed migration records for the observed links in neighboring countries, based on the relative area of the nonbreeding range in each country (Fig. S7c,d). Specifically, we interpolated the number of migratory records n_f in a given focal nonbreeding country f as:

$$n_f = \left(\prod_i^N \frac{n_i}{A_i}\right)^{\frac{1}{N}} \cdot A_f$$

where: n_i is the number of observed migration records in each neighboring nonbreeding country i; N is the set of neighboring countries within the MCP; A_i is the nonbreeding range area within each neighboring country i; and A_f is the nonbreeding range area within the focal country f. The expected number of migratory records was thus interpolated from the area of nonbreeding range within the focal country by assuming that the density of records there is the geometric mean of the densities of records in the neighboring countries (see example below; Fig. S7). To avoid inflating the number of interpolated migratory records, whenever the nonbreeding range in the focal country was larger than the largest among its neighbors, we set the former as equal to the latter (so for all i, $A_f \leq A_i$).

B. Sensitivity of results to including inferred migratory links

Inferred links corresponded to 16% of all links analyzed and were typically weak (average 10.4, range 0.7 – 32.2). They had a negligible effect on the results: results on priorities identified (Appendix S9) were the same when inferred links were excluded, with the single exception that Egyptian vultures in South Sudan appear as a new priority. There is a strong correlation between the number of gap species per country when these are obtained by including versus excluding inferred links (Spearman correlation coefficient equals 0.94 for landbirds and 0.90 for raptors).

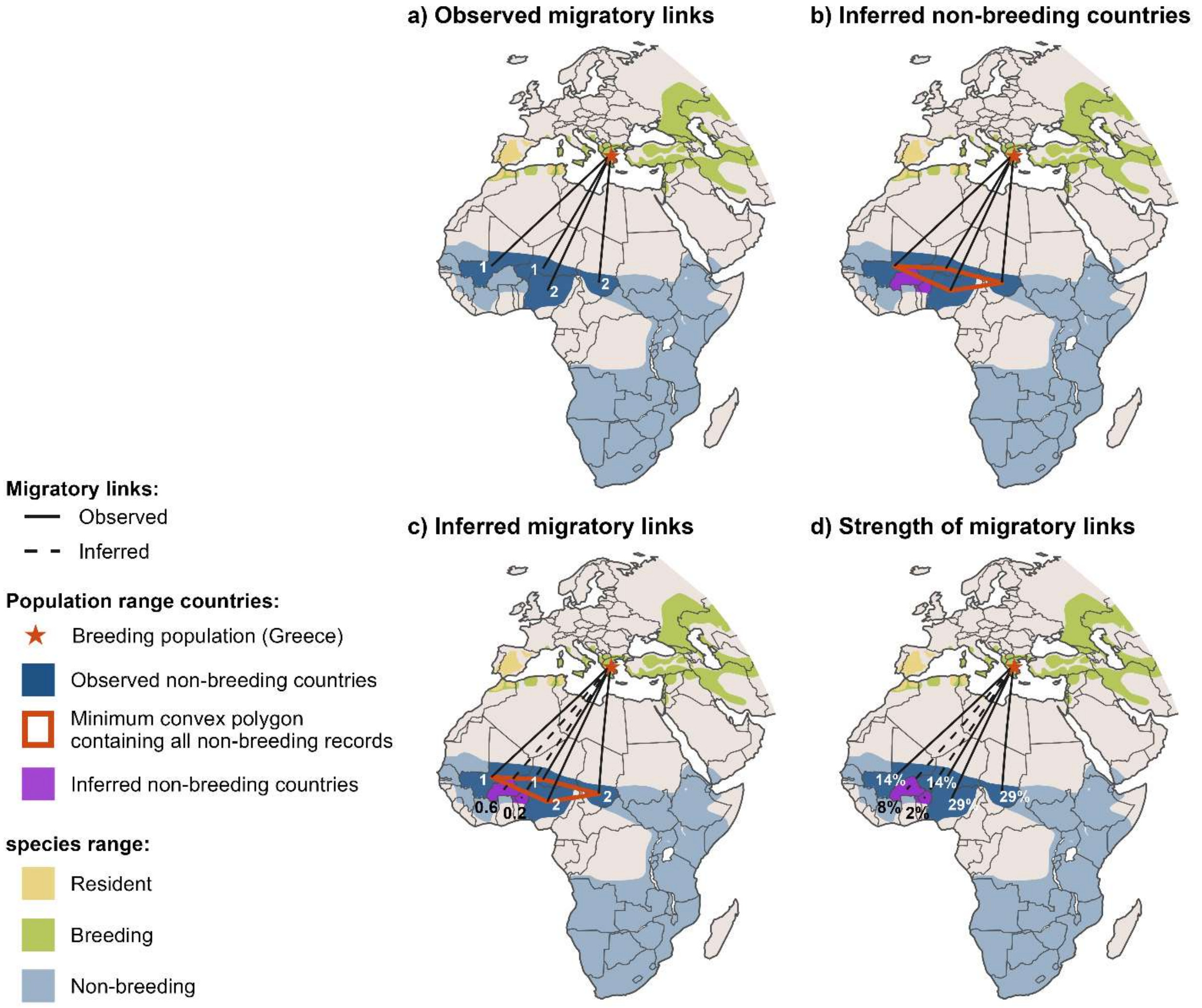

Figure S7. Taking as example the lesser kestrel (*Falco naumanni*) population breeding in Greece, the figure below illustrates: (a) the observed migratory links, with the number of migration records (i.e., tracked individuals) as obtained from the literature. The area in dark blue corresponds to the species' nonbreeding range as mapped in BirdLife International and Handbook of the Birds of the World (2018) within the countries with migration records; in light blue the nonbreeding range elsewhere. For each migratory link, the position in the nonbreeding country is the centroid of the respective dark blue polygon. (b) Minimum convex polygon encompassing the centroids in nonbreeding countries. This touches the nonbreeding range within two additional countries (in violet), resulting in two new inferred migratory links (dashed lines). (c) Number of migratory records for the inferred migratory links (see formula in main text), estimated from the density of records in neighboring countries. (d) Estimated strength of migratory links created by a population of lesser kestrels migrating between Greece and each of six countries within the species' nonbreeding range in Sub-Saharan Africa.

Appendix S8: Migration records by European region

Number of migration records compiled and respective populations organized by region in Europe.

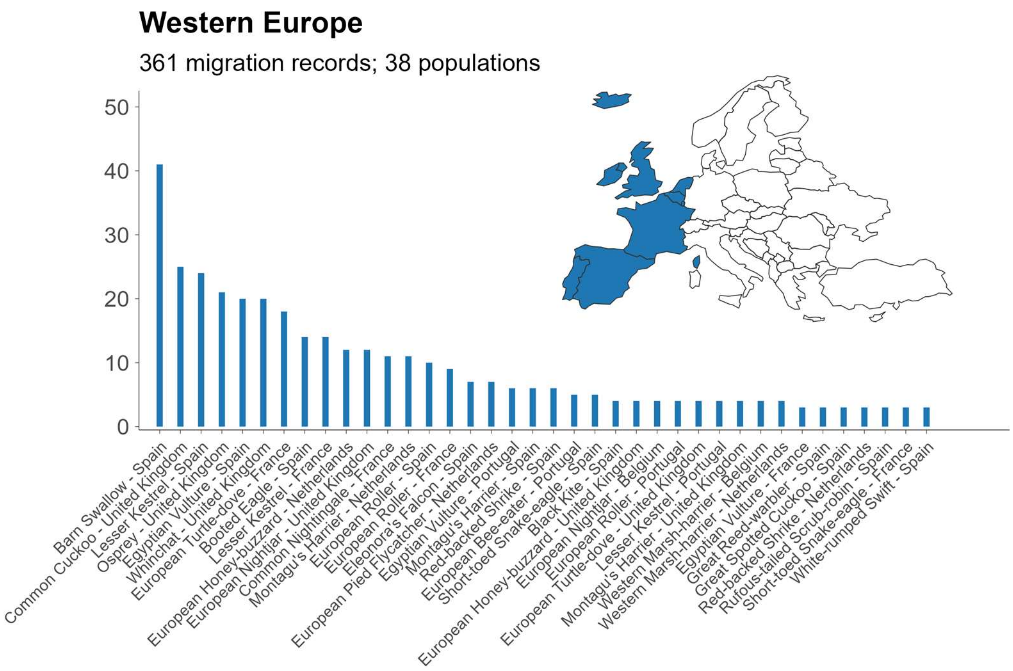

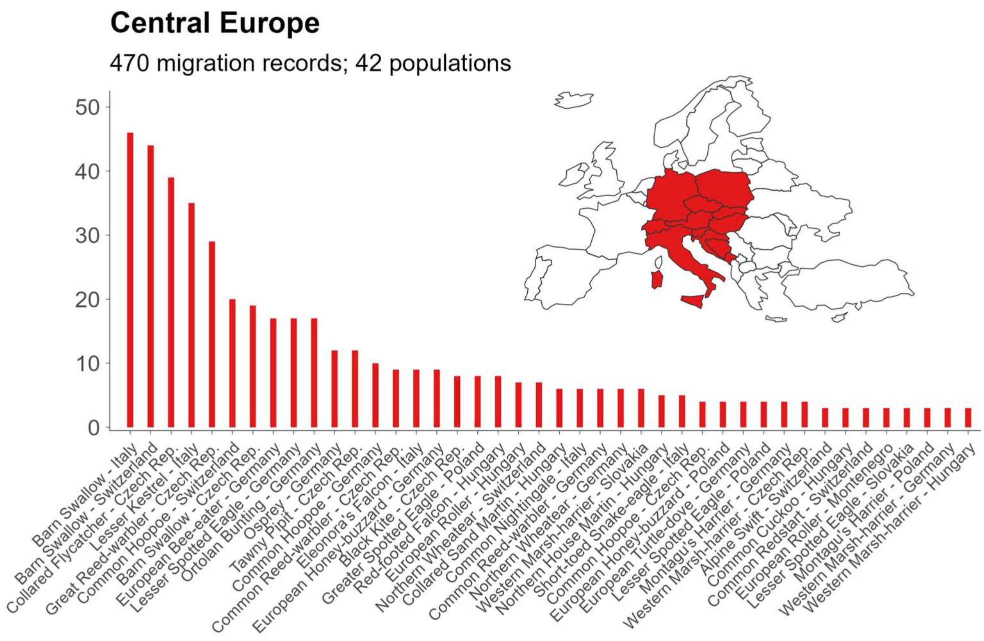

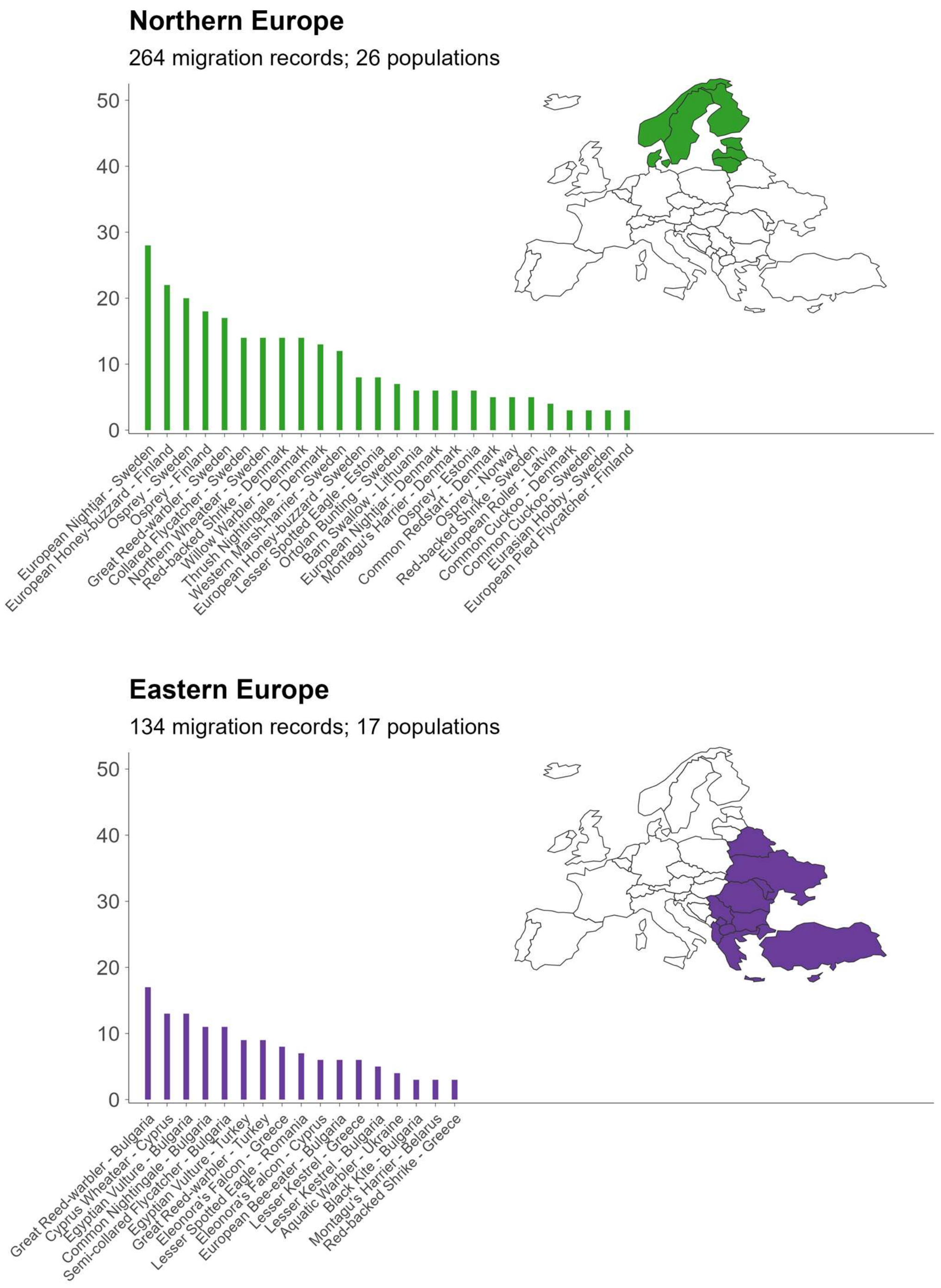

Figure S8. Number of migration records compiled in this study and respective populations organized by region in Europe.

References

Appendix S1

Lisovski, S., Bauer, S., Briedis, M., Davidson, S. C., Dhanjal-Adams, K. L., Hallworth, M. T., Karagicheva, J., Meier, C. M., Merkel, B., Ouwehand, J., Pedersen, L., Rakhimberdiev, E., Roberto-Charron, A., Seavy, N. E., Sumner, M. D., Taylor, C. M., Wotherspoon, S. J., & Bridge, E. S. (2020). Light-level geolocator analyses: A user's guide. *Journal of Animal Ecology*, *89*(1), 221–236. https://doi.org/10.1111/1365-2656.13036

Olson, D. M., Dinerstein, E., Wikramanayake, E. D., Burgess, N. D., Powell, G. V. N., Underwood, E. C., D'amico, J. A., Itoua, I., Strand, H. E., Morrison, J. C., Loucks, C. J., Allnutt, T. F., Ricketts, T. H., Kura, Y., Lamoreux, J. F., Wettengel, W. W., Hedao, P., & Kassem, K. R. (2001). Terrestrial Ecoregions of the World: A New Map of Life on Earth: A new global map of terrestrial ecoregions provides an innovative tool for conserving biodiversity. *BioScience*, *51*(11), 933–938. https://doi.org/10.1641/0006-3568(2001)051[0933:TEOTWA]2.0.CO;2

Appendix S2

BirdLife International (2021). *European Red List of Birds*. Publications Office of the European Union.

BirdLife International and Handbook of the Birds of the World (2018). *Bird species distribution maps of the world. Version 2018.1*. http://datazone.birdlife.org/species/requestdis.

UNEP/CMS. (2008). *Memorandum of Understanding on the Conservation of Migratory Birds of Prey in Africa and Eurasia (Raptors-MOU)*. Convention on the Conservation of Migratory Species of Wild Animals (CMS). https://www.cms.int/raptors/en/page/agreement-text

UNEP/CMS. (2014). *African-Eurasian Migratory Landbirds Action Plan (AEMLAP).* Convention on the Conservation of Migratory Species of Wild Animals (CMS). https://www.cms.int/sites/default/files/document/cop11_Doc_23_1_4_Rev1_Landbirds_AP_E.pdf

Walther, B. A. (2005). *List of Western Palearctic bird species migrating within Africa (last updated March 2005)*. Zoological Museum, University of Copenhagen. http:// http://macroecointern.dk/africamigrants/Specieslist01_10_05.htm

Appendix S3

Blackburn, E., Burgess, M., Freeman, B., Risely, A., Izang, A., Ivande, S., Hewson, C., & Cresswell, W. (2017). Low and annually variable migratory connectivity in a long-distance migrant: Whinchats Saxicola rubetra may show a bet-hedging strategy. *Ibis*, *159*(4), 902–918. https://doi.org/10.1111/ibi.12509

Blackburn, E., Burgess, M., Freeman, B., Risely, A., Izang, A., Ivande, S., Hewson, C., & Cresswell, W. (2019). Spring migration strategies of Whinchat Saxicola rubetra when successfully crossing potential barriers of the Sahara and the Mediterranean Sea. *Ibis*, *161*(1), 131–146. https://doi.org/10.1111/ibi.12610

Briedis, M., Bauer, S., Adamík, P., Alves, J. A., Costa, J. S., Emmenegger, T., Gustafsson, L., Koleček, J., Krist, M., Liechti, F., Lisovski, S., Meier, C. M., Procházka, P., & Hahn, S. (2020). Broad-scale patterns of the Afro-Palaearctic landbird migration. *Global Ecology and Biogeography*, *29*, 722–735. https://doi.org/10.1111/geb.13063

Briedis, M., Bauer, S., Adamık, P., Alves, J. A., Costa, J. S., Emmenegger, T., Gustafsson, L., Kole, J., Liechti, F., Meier, C. M., Prochazka, P., & Hahn, S. (2019). *A full annual perspective on sex-biased migration timing in long-distance migratory birds*. 9.

Brlík, V., Koleček, J., Burgess, M., Hahn, S., Humple, D., Krist, M., Ouwehand, J., Weiser, E. L., Adamík, P., Alves, J. A., Arlt, D., Barišić, S., Becker, D., Belda, E. J., Beran, V., Both, C., Bravo, S. P., Briedis, M., Chutný, B., … Procházka, P. (2020). Weak effects of geolocators on small birds: A meta-analysis controlled for phylogeny and publication bias. *Journal of Animal Ecology*, *89*(1), 207–220. https://doi.org/10.1111/1365-2656.12962

Cresswell, W., Kazeh, N. W., & Patchett, R. (2020). Local human population increase in the nonbreeding areas of long-distance migrant bird species is only weakly associated with their declines, even for synanthropic species. *Diversity and Distributions*, *26*, 340–351. https://doi.org/10.1111/ddi.13006

Finch, T., Butler, S. J., Franco, A. M. A., & Cresswell, W. (2017). Low migratory connectivity is common in long-distance migrant birds. *Journal of Animal Ecology*, *86*(3), 662–673. https://doi.org/10.1111/1365-2656.12635

Guilherme, J. L. (2022). A database of migration records between countries established by African-Eurasian migratory landbirds and raptors [Data set]. Zenodo. https://doi.org/10.5281/zenodo.7044193

Meyburg, B.-U., Ellis, D. H., Meyburg, C., Mendelsohn, J. M., & Scheller, W. (2001). Satellite tracking of two Lesser Spotted Eagles, *Aquila pomarina* , migrating from Namibia. *Ostrich*, *72*(1–2), 35–40. https://doi.org/10.2989/00306520109485281

Thorup, K., Vega, M. L., Snell, K. R. S., Lubkovskaia, R., Willemoes, M., Sjöberg, S., Sokolov, L. V., & Bulyuk, V. (2020). Flying on their own wings: Young and adult cuckoos respond similarly to long-distance displacement during migration. *Scientific Reports*, *10*(1), 7698. https://doi.org/10.1038/s41598-020-64230-x

Appendix S4

Agostini, N., Prommer, M., V'aczi, M., & Panuccio, M. (2019). Time vs risk minimization strategy: Repeated large scale loop migrations of an adult European Honey Buzzard. *Avocetta*, 43. https://doi.org/10.30456/AVO.2019103

Åkesson, S., Atkinson, P. W., Bermejo, A., Puente, J. de la, Ferri, M., Hewson, C. M., Holmgren, J., Kaiser, E., Kearsley, L., Klaassen, R. H. G., Kolunen, H., Matsson, G., Minelli, F., Norevik, G., Pietiäinen, H., Singh, N. J., Spina, F., Viktora, L., & Hedenström, A. (2020). Evolution of chain migration in an aerial insectivorous bird, the common swift *Apus apus*. *Evolution*, 74(10), 2377–2391. https://doi.org/10.1111/evo.14093

Arizaga, J., Willemoes, M., Unamuno, E., Unamuno, J. M., & Thorup, K. (2015). Following year-round movements in Barn Swallows using geolocators: Could breeding pairs remain together during the winter? *Bird Study*, 62(1), 141–145. https://doi.org/10.1080/00063657.2014.998623

Arkumarev, V., Dobrev, D. & Stamenov, A. (2019). First record of Eurasian Griffon Vulture *Gyps fulvus* from the Balkans migrating to South Sudan revealed by GPS tracking. *Scopus* 39(2): 27–35

Bächler, E., Hahn, S., Schaub, M., Arlettaz, R., Jenni, L., Fox, J. W., Afanasyev, V., & Liechti, F. (2010). Year-Round Tracking of Small Trans-Saharan Migrants Using Light-Level Geolocators. *PLOS ONE*, 5(3), e9566. https://doi.org/10.1371/journal.pone.0009566

Bán, M., Moskát, C., Fülöp, A., & Hauber, M. E. (2018). Return migration of Common Cuckoos (*Cuculus canorus*) between breeding grounds in Hungary and wintering grounds in Africa as documented by non-PTT GPS technology. *Journal of Ornithology*, 159(2), 337–344. https://doi.org/10.1007/s10336-017-1508-x

Bermejo, A., Vidal-Mateo, J., & de la Puente, J. (2017). Migración y ecología espacial de la población española de águila calzada (V. Urios, Ed.). SEO/BirdLife. http://www.seo.org/boletin/seguimiento/migracion/02_aguila_calzada/

Bernardy, P. (2016). Die Zugstrategie des Ortolans (*Emberiza hortulana*) - Erkundung der Brut-, Rast- und Überwinterungsgebiete zum Schutz der niedersächsischen Kernpopulation (Report No. 31005/01). Avifaunistische Arbeitsgemeinschaft Lüchow-Dannenberg e.V. https://www.dbu.de/projekt_31005/01_db_2409.html

BirdLife International and Handbook of the Birds of the World (2018). *Bird species distribution maps of the world. Version 2018.1*. http://datazone.birdlife.org/species/requestdis.

Bird Telemetry (2018). Black Kite, Bird Telemetry Team. Retrieved from http://www.birdtelemetry.cz/en/maps/?druh=black-kite [Accessed 28 April 2021]

Bird Telemetry (2020). Marsh Harrier, Bird Telemetry Team. Retrieved from http://www.birdtelemetry.cz/en/maps/?druh=marsh-harrier/ [Accessed 28 April 2021]

Briedis, M., Beran, V., Adamík, P., & Hahn, S. (2020). Integrating light-level geolocation with activity tracking reveals unexpected nocturnal migration patterns of the tawny pipit. *Journal of Avian Biology*, 51(9). https://doi.org/10.1111/jav.02546

Briedis, M., Beran, V., Hahn, S., & Adamík, P. (2016b). Annual cycle and migration strategies of a habitat specialist, the Tawny Pipit *Anthus campestris*, revealed by geolocators. *Journal of Ornithology*, 157(2), 619–626. https://doi.org/10.1007/s10336-015-1313-3

Briedis, M., Hahn, S., Gustafsson, L., Henshaw, I., Träff, J., Král, M., & Adamík, P. (2016b). Breeding latitude leads to different temporal but not spatial organization of the annual cycle in a long-distance migrant. *Journal of Avian Biology*, 47(6), 743–748. https://doi.org/10.1111/jav.01002

Briedis, M., Hahn, S., Krist, M., & Adamík, P. (2018b). Finish with a sprint: Evidence for time-selected last leg of migration in a long-distance migratory songbird. *Ecology and Evolution*, 8(14), 6899–6908. https://doi.org/10.1002/ece3.4206

Briedis, M., Kurlavičius, P., Mackevičienė, R., Vaišvilienė, R., & Hahn, S. (2018a). Loop migration, induced by seasonally different flyway use, in Northern European Barn Swallows. *Journal of Ornithology*, 159(4), 885–891. https://doi.org/10.1007/s10336-018-1560-1

Briedis, M., Träff, J., Hahn, S., Ilieva, M., Král, M., Peev, S., & Adamík, P. (2016c). Year-round spatiotemporal distribution of the enigmatic Semi-collared Flycatcher *Ficedula semitorquata*. *Journal of Ornithology*, 157(3), 895–900. https://doi.org/10.1007/s10336-016-1334-6

BSPB (2013). Imperial Eagle satellite tracking - BSPB Life+ project "Save the Raptors". Bulgarian Society for the Protection of Birds. Retrieved from http://corvus.bspb.org:8001/ [Accessed 27 April 2021]

BTO (2018). Nightjar Tracking Project. BTO - British Trust for Ornithology. Retrieved from https://www.bto.org/our-science/topics/tracking/tracking-studies/nightjars [Accessed 28 April 2021]

Burgess, M. D., Finch, T., Border, J. A., Castello, J., Conway, G., Ketcher, M., Lawrence, M., Orsman, C. J., Mateos, J., Proud, A., Westerberg, S., Wiffen, T., & Henderson, I. G. (2020). Weak migratory connectivity, loop migration and multiple nonbreeding site use in British breeding Whinchats *Saxicola rubetra*. *Ibis*, 162(4), 1292-1302. https://doi.org/10.1111/ibi.12825

Catry, I., Dias, M. P., Catry, T., Afanasyev, V., Fox, J., Franco, A. M. A., & Sutherland, W. J. (2011). Individual variation in migratory movements and winter behaviour of Iberian Lesser Kestrels *Falco naumanni* revealed by geolocators. *Ibis*, 153(1), 154–164. https://doi.org/10.1111/j.1474-919X.2010.01073.x

Eraud, C., Rivière, M., Lormée, H., Fox, J. W., Ducamp, J.-J., & Boutin, J.-M. (2013). Migration Routes and Staging Areas of Trans-Saharan Turtle Doves Appraised from Light-Level Geolocators. *PLOS ONE*, 8(3), e59396. https://doi.org/10.1371/journal.pone.0059396

Evens, R., Conway, G. J., Henderson, I. G., Cresswell, B., Jiguet, F., Moussy, C., Sénécal, D., Witters, N., Beenaerts, N., & Artois, T. (2017). Migratory pathways, stopover zones and wintering destinations of Western European Nightjars *Caprimulgus europaeus*. *Ibis*, 159(3), 680–686. https://doi.org/10.1111/ibi.12469

Finch, T., Saunders, P., Avilés, J. M., Bermejo, A., Catry, I., Puente, J. de la, Emmenegger, T., Mardega, I., Mayet, P., Parejo, D., Račinskis, E., Rodríguez-Ruiz, J., Sackl, P., Schwartz, T., Tiefenbach, M., Valera, F., Hewson, C., Franco, A., & Butler, S. J. (2015). A pan-European, multipopulation assessment of migratory connectivity in a near-threatened migrant bird. *Diversity and Distributions*, 21(9), 1051–1062. https://doi.org/10.1111/ddi.12345

Gersten, A., & Hahn, S. (2016). Timing of migration in Common Redstarts *(Phoenicurus phoenicurus*) in relation to the vegetation phenology at residence sites. *Journal of Ornithology*, 157(4), 1029–1036. https://doi.org/10.1007/s10336-016-1359-x

GKA (2020). Routes bruine zenderkieken 2019/2020. Grauwe Kiekendief Kenniscentrum Akkervogels. Retrieved from https://grauwekiekendief.nl/volg-zenderkieken-bruine/ [Accessed 29 April 2020]

Gschweng, M., Kalko, E. K. V., Querner, U., Fiedler, W., & Berthold, P. (2008). All across Africa: Highly individual migration routes of Eleonora's falcon. *Proceedings of the Royal Society B: Biological Sciences*, 275(1653), 2887–2896. https://doi.org/10.1098/rspb.2008.0575

Guilherme, J. L. (2022). A database of migration records between countries established by African-Eurasian migratory landbirds and raptors [Data set]. Zenodo. https://doi.org/10.5281/zenodo.7044193

Hadjikyriakou, T. G., Kassara, C., de Roland, L.-A. R., Giokas, S., Tsiopelas, N., Evangelidis, A., Thorstrom, R., & Kirschel, A. N. G. (2020). Phenology, variation in habitat use, and daily activity patterns of

Eleonora’s falcon overwintering in Madagascar. *Landscape Ecology*, 35(1), 159–172. https://doi.org/10.1007/s10980-019-00940-6

Hadjikyriakou, T. G., Nwankwo, E. C., Virani, M. Z., & Kirschel, A. N. G. (2020). Habitat availability influences migration speed, refueling patterns and seasonal flyways of a fly-and-forage migrant. *Movement Ecology*, 8(1), 10. https://doi.org/10.1186/s40462-020-0190-4

Hahn, S., Alves, J. A., Bedev, K., Costa, J. S., Emmenegger, T., Schulze, M., Tamm, P., Zehtindjiev, P., & Dhanjal-Adams, K. L. (2020). Range wide migration corridors and non-breeding areas of a northward expanding Afro-Palaearctic migrant, the European Bee-eater *Merops apiaster*. *Ibis*, 162(2), 345-355 https://doi.org/10.1111/ibi.12752

Hahn, S., Briedis, M., Barboutis, C., Schmid, R., Schulze, M., Seifert, N., Szép, T., & Emmenegger, T. (2021). Spatially different annual cycles but similar haemosporidian infections in distant populations of collared sand martins. *BMC Zoology*, 6(1), 6. https://doi.org/10.1186/s40850-021-00071-z

Hahn, S., Emmenegger, T., Lisovski, S., Amrhein, V., Zehtindjiev, P., & Liechti, F. (2014). Variable detours in long-distance migration across ecological barriers and their relation to habitat availability at ground. *Ecology and Evolution*, 4(21), 4150–4160. https://doi.org/10.1002/ece3.1279

Hake, M., Kjellén, N., & Alerstam, T. (2001). Satellite tracking of Swedish Ospreys *Pandion haliaetus*: Autumn migration routes and orientation. *Journal of Avian Biology*, 32(1), 47–56. https://doi.org/10.1034/j.1600-048X.2001.320107.x

Hake, M., Kjellén, N., & Alerstam, T. (2003). Age-dependent migration strategy in honey buzzards *Pernis apivorus* tracked by satellite. *Oikos*, 103(2), 385–396. https://doi.org/10.1034/j.1600-0706.2003.12145.x

Hewson, C. M., Thorup, K., Pearce-Higgins, J. W., & Atkinson, P. W. (2016). Population decline is linked to migration route in the Common Cuckoo. *Nature Communications*, 7, 12296. https://doi.org/10.1038/ncomms12296

Horns, J. J., Buechley, E., Chynoweth, M., Aktay, L., Çoban, E., Kırpık, M. A., Herman, J. M., Şaşmaz, Y., & Şekercioğlu, Ç. H. (2016). Geolocator tracking of Great Reed-Warblers (*Acrocephalus arundinaceus*) identifies key regions for migratory wetland specialists in the Middle East and sub-Saharan East Africa. *The Condor*, 118(4), 835–849. https://doi.org/10.1650/CONDOR-16-63.1

Ibáñez-Álamo, J. D., Rühmann, J., Pérez-Contreras, T., & Soler, M. (2019). Migration behavior and performance of the great spotted cuckoo (*Clamator glandarius*). PLOS ONE, 14(1), e0208436. https://doi.org/10.1371/journal.pone.0208436

Jacobsen, L. B., Jensen, N. O., Willemoes, M., Hansen, L., Desholm, M., Fox, A. D., Tøttrup, A. P., & Thorup, K. (2017). Annual spatiotemporal migration schedules in three larger insectivorous birds:

European nightjar, common swift and common cuckoo. *Animal Biotelemetry*, 5(1), 4. https://doi.org/10.1186/s40317-017-0119-x

Jiguet, F., Arlettaz, R., Belik, V., Bernardy, P., Copete, J.L., Czajkowski, M.A., Dale, S., Dombrovski, V., Elts, J., Ferrand, Y., et al. (2016). Migration strategy of the ortolan bunting final report of the scientific committee. Museum Natl. D'Histoire Nat.

Jiguet, F., Chevallier, D., Baillon, F., Ventroux, J., & Cavallin, P. (2012). Sub-Saharan staging areas of a first-summer Short-toed Snake Eagle *Circaetus gallicus*. *Bird Study*, 59(1), 102–104. https://doi.org/10.1080/00063657.2011.648607

Kassara, C., Fric, J., Gschweng, M., & Sfenthourakis, S. (2012). Complementing the puzzle of Eleonora's Falcon (*Falco eleonorae*) migration: New evidence from an eastern colony in the Aegean Sea. *Journal of Ornithology*, 153(3), 839–848. https://doi.org/10.1007/s10336-011-0802-2

Kassara, C., Gangoso, L., Mellone, U., Piasevoli, G., Hadjikyriakou, T. G., Tsiopelas, N., Giokas, S., López-López, P., Urios, V., Figuerola, J., Silva, R., Bouten, W., Kirschel, A. N. G., Virani, M. Z., Fiedler, W., Berthold, P., & Gschweng, M. (2017). Current and future suitability of wintering grounds for a long-distance migratory raptor. *Scientific Reports*, 7(1), 8798. https://doi.org/10.1038/s41598-017-08753-w

Klaassen, R., Klaassen, H., Berghuis, A., Berghuis, M., Schreven, K., van der Horst, Y., Verkade, H., & Kearsley, L. (2014). Trekroutes en overwinteringsgebieden van Nederlandse Gierzwaluwen ontrafeld met geolocators. *Limosa*, 87, 173–181.

Klvaňa, P., Cepák, J., Munclinger, P., Michálková, R., Tomášek, O., & Albrecht, T. (2018). Around the Mediterranean: An extreme example of loop migration in a long-distance migratory passerine. *Journal of Avian Biology*, 49(2), jav-01595. https://doi.org/10.1111/jav.01595

Koleček, J., Hahn, S., Emmenegger, T., & Procházka, P. (2018). Intra-tropical movements as a beneficial strategy for Palearctic migratory birds. *Royal Society Open Science*, 5(1). https://doi.org/10.1098/rsos.171675

Koleček, J., Procházka, P., El-Arabany, N., Tarka, M., Ilieva, M., Hahn, S., Honza, M., Puente, J. de la, Bermejo, A., Gürsoy, A., Bensch, S., Zehtindjiev, P., Hasselquist, D., & Hansson, B. (2016). Cross-continental migratory connectivity and spatiotemporal migratory patterns in the great reed warbler. *Journal of Avian Biology*, 47(6), 756–767. https://doi.org/10.1111/jav.00929

KOO (2019). Ekologia i ochrona trzmielojada. Komitetu Ochrony Orłów. Retrieved from http://www.koo.org.pl/aktualnosci/220-ekologia-i-ochrona-trzmielojada [Accessed 23 May 2020]

Kotkaklubi (2016). Osprey, Migration map. Kotkaklubi (Eagle Club) and 5D Vision Ltd. Retrieved from http://birdmap.5dvision.ee/EN/ [Accessed 29 April 2021]

Kotkaklubi (2018). Greater Spotted Eagle, Migration map. Kotkaklubi (Eagle Club) and 5D Vision Ltd. Retrieved from http://birdmap.5dvision.ee/EN/ [Accessed 29 April 2021]

Kristensen, M. W., Tøttrup, A. P., & Thorup, K. (2013). Migration of the Common Redstart (*Phoenicurus phoenicurus*): A Eurasian Songbird Wintering in Highly Seasonal Conditions in the West African Sahel. *The Auk*, 130(2), 258–264. https://doi.org/10.1525/auk.2013.13001

Lemke, H. W., Tarka, M., Klaassen, R. H. G., Åkesson, M., Bensch, S., Hasselquist, D., & Hansson, B. (2013). Annual Cycle and Migration Strategies of a Trans-Saharan Migratory Songbird: A Geolocator Study in the Great Reed Warbler. *PLOS ONE*, 8(10), e79209. https://doi.org/10.1371/journal.pone.0079209

Lerche-Jørgensen, M., Willemoes, M., Tøttrup, A. P., Snell, K. R. S., & Thorup, K. (2017). No apparent gain from continuing migration for more than 3000 kilometres: Willow warblers breeding in Denmark winter across the entire northern Savannah as revealed by geolocators. *Movement Ecology*, 5(1), 17. https://doi.org/10.1186/s40462-017-0109-x

Liechti, F., Scandolara, C., Rubolini, D., Ambrosini, R., Korner-Nievergelt, F., Hahn, S., Lardelli, R., Romano, M., Caprioli, M., Romano, A., Sicurella, B., & Saino, N. (2015). Timing of migration and residence areas during the nonbreeding period of barn swallows *Hirundo rustica* in relation to sex and population. *Journal of Avian Biology*, 46(3), 254–265. https://doi.org/10.1111/jav.00485

Liechti, F., Witvliet, W., Weber, R., & Bächler, E. (2013). First evidence of a 200-day non-stop flight in a bird. *Nature Communications*, 4, 2554. https://doi.org/10.1038/ncomms3554

Limiñana, R., Romero, M., Mellone, U., & Urios, V. (2012). Mapping the migratory routes and wintering areas of Lesser Kestrels *Falco naumanni*: New insights from satellite telemetry. *Ibis*, 154(2), 389–399. https://doi.org/10.1111/j.1474-919X.2011.01210.x

Limiñana, R., Soutullo, A., & Urios, V. (2007). Autumn migration of Montagu's harriers Circus pygargus tracked by satellite telemetry. *Journal of Ornithology*, 148(4), 517–523. https://doi.org/10.1007/s10336-007-0182-9

Literák, I., Balla, M., Vyhnal, S., Škrábal, J., Peške, L., Chrašč, P., & Systad, G. (2020). Natal dispersal of black kites from Slovakia. *Biologia*, 75(4), 591–598. https://doi.org/10.2478/s11756-019-00323-x

López-Calderón, C., Magallanes, S., Marzal, A., & Balbontín, J. (2021). The Migration System of Barn Swallows *Hirundo rustica* Breeding in Southwestern Spain and Wintering Across West Africa. *Ardeola*, 68(2). https://doi.org/10.13157/arla.68.2.2021.ra2

Lormee, H., Boutin, J.-M., Pinaud, D., Bidault, H., & Eraud, C. (2016). Turtle Dove *Streptopelia turtur* migration routes and wintering areas revealed using satellite telemetry. *Bird Study*, 63(3), 425–429. https://doi.org/10.1080/00063657.2016.1185086

LUOMUS (2017). Finnish Satellite Ospreys. Finish Natural History Museum. Retrieved from http://www.luomus.fi/en/finnish-satellite-ospreys [Accessed 19 May 2020]

LUOMUS (2019). Pallid harriers. Finish Natural History Museum. Retrieved from http://www.luomus.fi/en/pallid-harriers [Accessed 19 May 2020]

Mackrill, T. R. (2017). Migratory behaviour and ecology of a trans-Saharan migrant raptor, the Osprey *Pandion haliaetus*. University of Leicester.

Mellone, U., López-López, P., Limiñana, R., Piasevoli, G., & Urios, V. (2013). The trans-equatorial loop migration system of Eleonora's falcon: Differences in migration patterns between age classes, regions and seasons. *Journal of Avian Biology*, 44: 417-426. https://doi.org/10.1111/j.1600-048X.2013.00139.x

Mellone, U., Lucia, G., Mallìa, E., & Urios, V. (2016). Individual variation in orientation promotes a 3000-km latitudinal change in wintering grounds in a long-distance migratory raptor. *Ibis*, 158(4), 887–893. https://doi.org/10.1111/ibi.12401

Meyburg, B.-U., & Meyburg, C. (2009). Annual cycle, timing and speed of migration of a pair of Lesser Spotted Eagles (*Aquila pomarina*) – a study by means of satellite telemetry. *Populationsökologie Greifvogel- Und Eulenarten*, 6, 63–85.

Meyburg, B.-U., Meyburg, C., & Barbraud, J.-C. (1998). Migration strategies of an adult Short-toed eagle *Circaetus gallicus* Tracked by satellite. *Alauda*, 66(1), 39–48.

Meyburg, B.-U., Meyburg, C., & Pacteau, C. (1996). Migration automnale d´un Circaète Jean-Le-Blanc *Circaetus gallicus* suivi par satellite. *Alauda*, 64(3): 339-344.

Meyburg, B.-U., Roepke, D., Meyburg, C., & Baß, A. (2011c, September 29 - October 4). Satellitentelemetrische Untersuchungen an adulten deutschen Fischadlern (*Pandion haliaetus*) [Conference poster]. 144. Jahresversammlung der Deutschen Ornithologen-Gesellschaft, Potsdam, Germany.

Meyburg, B.-U., Scheller, W., & Meyburg, C. (2000). Migration and wintering of the Lesser Spotted Eagle *Aquila pomarina*: A Study by Means of Satellite Telemetry. *Global Environmental Resources*, 4(2), 183–193.

Meyburg, B.-U., Gallardo, M., Meyburg, C., & Dimitrova, E. (2004a). Migrations and sojourn in Africa of Egyptian vultures (*Neophron percnopterus*) tracked by satellite. *Journal of Ornithology*, 145(4), 273–280. https://doi.org/10.1007/s10336-004-0037-6

Meyburg, B.-U., Meyburg, C., Bělka, T., Šreibr, O., & Vrana, J. (2004b). Migration, wintering and breeding of a lesser spotted eagle (*Aquila pomarina*) from Slovakia tracked by satellite. *Journal of Ornithology*, 145(1), 1–7. https://doi.org/10.1007/s10336-003-0014-5

Meyburg, B.-U., Graszynski, K., Langgemach, T., Sömmer, P., & Bergmanis, U. (2008). Cainism, nestling management in Germany in 2004-2007 and satellite tracking of juveniles in the Lesser Spotted Eagle (*Aquila pomarina*). *Slovak Raptor Journal*, 2(1), 53–72. https://doi.org/10.2478/v10262-012-0018-2

Meyburg, B.-U., Howey, P. W., Meyburg, C., & Fiuczynski, K. D. (2011b). Two complete migration cycles of an adult Hobby tracked by satellite. *British Birds*, 14.

Meyburg, C., Meyburg, B.-U., Ziesemer, F., & Martens, H. D. (2011a, August 27-30). On the Biology of the Honey Buzzard (*Pernis apivorus*)—Some results revealed by Satellite Telemetry [Conference poster]. 8th Conference of the European Ornithologists' Union, Riga, Latvia.

Milvus Group (2010). Migration Map of the Satellite Tracked Romanian Lesser Spotted Eagles (*Aquila pomarina*). Retrieved from http://www.pomarina.ro/pomarina/index.php [Accessed 24 May 2020]

MME Birdlife Hungary (2009). Red-Footed Falcon, satellitetracking. MME BirdLife Hungary. Retrieved from https://www.satellitetracking.eu/inds/showmap/?check_161=161&check_163=163&check_43=43&check_47=47&check_42=42&check_238=238&check_235=235&check_49=49# [Accessed 27 April 2021]

MME Birdlife Hungary (2014). Marsh Harrier, satellitetracking MME BirdLife Hungary. Retrieved from https://www.satellitetracking.eu/inds/showmap/?check_246=246&check_352=352&check_328=328&check_332=332&check_333=333&check_193=193# [Accessed 19 May 2020]

MME Birdlife Hungary (2015). European Roller, MME BirdLife Hungary. Retrieved from https://www.satellitetracking.eu/inds/showmap/?check_292=292&check_325=325&check_294=294&check_219=219&check_323=323&check_293=293&check_217=217# [Accessed 19 May 2020]

MME Birdlife Hungary (2016). Lesser Spotted Eagles, satellitetracking. MME BirdLife Hungary. Retrieved from https://www.satellitetracking.eu/inds/showmap/?check_233=233&check_234=234 [Accessed 19 May 2020]

MME Birdlife Hungary (2018). Short-Toed Snake-Eagle, satellitetracking. MME BirdLife Hungary. Retrieved from https://www.satellitetracking.eu/inds/showmap/?check_347=347&check_334=334 [Accessed 23 May 2020]

Monti, F., Grémillet, D., Sforzi, A., Dominici, J. M., Bagur, R. T., Navarro, A. M., Fusani, L., Klaassen, R. H. G., Alerstam, T., & Duriez, O. (2018). Migration distance affects stopover use but not travel speed:

Contrasting patterns between long- and short-distance migrating ospreys. *Journal of Avian Biology*, 49(10), e01839. https://doi.org/10.1111/jav.01839

Muñoz, A. R., Marquez, A. L., Chamorro, D., & Real, R. (2016). One complete migration cycle of an adult Griffon Vulture: From southern Spain to Senegambia as revealed by high-resolution GPS tracking technology. African Congress for Conservation Biology. *African Congress for Conservation Biology*.

NABU (2019). Turteltauben auf Reisen.Naturschutzbund Deutschland. Retrieved from https://blogs.nabu.de/zugvoegel/turteltauben-karte/ [Accessed 19 May 2020]

Norevik, G., Åkesson, S., Andersson, A., Bäckman, J., & Hedenström, A. (2019b). The lunar cycle drives migration of a nocturnal bird. *PLOS Biology*, 17(10), e3000456. https://doi.org/10.1371/journal.pbio.3000456

Norevik, G., Boano, G., Hedenström, A., Lardelli, R., Liechti, F., & Åkesson, S. (2019a). Highly mobile insectivorous swifts perform multiple intra-tropical migrations to exploit an asynchronous African phenology. *Oikos*, 128(5), 640–648. https://doi.org/10.1111/oik.05531

ONCFS (2016). La tourtrelle des bois. Office National de la Chasse et de la Faune Sauvage. Retrieved from http://turtledoveresearch.com/fr/ [Accessed 19 May 2020]

Østnes, J. E., Kroglund, R. T., Kleven, O., & Nygård, T. (2019). Migratory patterns of Ospreys (*Pandion haliaetus*) from central Norway. *Ornis Fennica* 96, 101-111.

Ouwehand, J., Ahola, M. P., Ausems, A. N. M. A., Bridge, E. S., Burgess, M., Hahn, S., Hewson, C. M., Klaassen, R. H. G., Laaksonen, T., Lampe, H. M., Velmala, W., & Both, C. (2016). Light-level geolocators reveal migratory connectivity in European populations of pied *flycatchers Ficedula hypoleuca*. *Journal of Avian Biology*, 47(1), 69–83. https://doi.org/10.1111/jav.00721

Ovčiariková, S., Škrábal, J., Matušík, H., Makoň, K., Mráz, J., Arkumarev, V., Dobrev, V., Raab, R., & Literák, I. (2020). Natal dispersal in Black Kites *Milvus migrans migrans* in Europe. *Journal of Ornithology*, 161(4), 935–951. https://doi.org/10.1007/s10336-020-01780-x

Patchett, R., & Cresswell, W. (2020). Regional wind patterns likely shape a seasonal migration detour. *Journal of Avian Biology*, 51(11). https://doi.org/10.1111/jav.02466

Pavón, D., Limiñana, R., Urios, V., Izquierdo, A., Yáñez, B., Ferrer, M., & Vega, A. de la. (2010). Autumn Migration of Juvenile Short-Toed Eagles *Circaetus gallicus* from Southeastern Spain. *Ardea*, 98(1), 113–118. https://doi.org/10.5253/078.098.0114

Pedersen, L., Onrubia, A., Vardanis, Y., Barboutis, C., Waasdorp, S., Helvert, M. van, Geertsma, M., Ekberg, P., Willemoes, M., Strandberg, R., Matsyna, E., Matsyna, A., Klaassen, R. H. G., Alerstam, T., Thorup, K., & Tøttrup, A. P. (2020). Remarkably similar migration patterns between different red-

backed shrike populations suggest that migration rather than breeding area phenology determines the annual cycle. *Journal of Avian Biology*, 51(10). https://doi.org/10.1111/jav.02475

Pedersen, L., Thorup, K., & Tøttrup, A. P. (2019). Annual GPS tracking reveals unexpected wintering area in a long-distance migratory songbird. *Journal of Ornithology*, 160(1), 265–270. https://doi.org/10.1007/s10336-018-1610-8

Phipps, W. L., López-López, P., Buechley, E. R., Oppel, S., Álvarez, E., Arkumarev, V., Bekmansurov, R., Berger-Tal, O., Bermejo, A., Bounas, A., Alanís, I. C., de la Puente, J., Dobrev, V., Duriez, O., Efrat, R., Fréchet, G., García, J., Galán, M., García-Ripollés, C., … Vallverdú, N. (2019). Spatial and Temporal Variability in Migration of a Soaring Raptor Across Three Continents. *Frontiers in Ecology and Evolution*, 7. https://doi.org/10.3389/fevo.2019.00323

Pilard, P., Bourgeois, M., & Sylla, D. (2017). Location by geolocators of wintering areas and phenology of prenuptial and postnuptial migrations of the French population of Lesser Kestrel *Falco naumanni*. *Alauda*, 85(1).

Procházka, P., Brlík, V., Yohannes, E., Meister, B., Auerswald, J., Ilieva, M., & Hahn, S. (2018). Across a migratory divide: Divergent migration directions and nonbreeding grounds of Eurasian reed warblers revealed by geolocators and stable isotopes. *Journal of Avian Biology*, 49(6), jav-012516. https://doi.org/10.1111/jav.01769

Rodríguez, A., Negro, J. J., Bustamante, J., Fox, J. W., & Afanasyev, V. (2009). Geolocators map the wintering grounds of threatened Lesser Kestrels in Africa. *Diversity and Distributions*, 15(6), 1010–1016. https://doi.org/10.1111/j.1472-4642.2009.00600.x

Roy Dennis Wildlife Foundation (2003). Honey buzzard satellite tracking. Retrieved from https://www.roydennis.org/animals/raptors/honey-buzzard/satellite-tracking/ [Accessed 27 April 2021]

Roy Dennis Wildlife Foundation (2004). Marsh Harrier satellite tracking. Retrieved from https://www.roydennis.org/o/wp-content/uploads/2011/11/Chick-21197-2004-2005.pdf [Accessed 27 April 2021]

Roy Dennis Wildlife Foundation (2010). Hobby satellite tracking. Retrieved from https://www.roydennis.org/animals/raptors/hobby/satellite-tracking/ [Accessed 27 April 2021]

RSPB (2008). Tracking ospreys. Royal Society for the Protection of Birds. Retrieved from https://www.rspb.org.uk/our-work/conservation/satellite-tracking-birds/tracking-ospreys/ [Accessed 19 May 2020]

RSPB (2012). Tracking turtle doves. Royal Society for the Protection of Birds. Retrieved from https://www.rspb.org.uk/our-work/conservation/satellite-tracking-birds/tracking-turtle-doves/ [Accessed 19 May 2020]

RSPB (2016). Tracking Montagu's harriers. Royal Society for the Protection of Birds. Retrieved from https://www.rspb.org.uk/our-work/conservation/satellite-tracking-birds/tracking-montagus-harriers/ [Accessed 19 May 2020]

Salewski, V., Flade, M., Lisovski, S., Poluda, A., Iliukha, O., Kiljan, G., Malashevich, U., & Hahn, S. (2019). Identifying migration routes and nonbreeding staging sites of adult males of the globally threatened Aquatic Warbler *Acrocephalus paludicola*. *Bird Conservation International*, 24(9), 503-514. https://doi.org/10.1017/S0959270918000357

Sarà, M., Bondì, S., Bermejo, A., Bourgeois, M., Bouzin, M., Bustamante, J., Puente, J. de la, Evangelidis, A., Frassanito, A., Fulco, E., Giglio, G., Gradev, G., Griggio, M., López-Ricaurte, L., Kordopatis, P., Marin, S., Martínez, J., Mascara, R., Mellone, U., … Rubolini, D. (2019). Broad-front migration leads to strong migratory connectivity in the lesser kestrel (*Falco naumanni*). *Journal of Biogeography*, 46(12), 2663-2677. https://doi.org/10.1111/jbi.13713

Schmaljohann, H., Meier, C., Arlt, D., Bairlein, F., van Oosten, H., Morbey, Y. E., Åkesson, S., Buchmann, M., Chernetsov, N., Desaever, R., Elliott, J., Hellström, M., Liechti, F., López, A., Middleton, J., Ottosson, U., Pärt, T., Spina, F., & Eikenaar, C. (2016). Proximate causes of avian protandry differ between subspecies with contrasting migration challenges. *Behavioral Ecology*, 27(1), 321–331. https://doi.org/10.1093/beheco/arv160

Selstam, G., Sondell, J., & Olsson, P. (2015). Wintering area and migration routes for Ortolan Buntings *Emberiza hortulana* from Sweden determined with light-geologgers. *Ornis Svecica*, 25, 3–14.

SEO BirdLife. (2008). Colebrera Europea, LA MIGRACIÓN DE LAS AVES - SEO/BirdLife. SEO Birdlife - Sociedad Española de Ornitología. http://www.migraciondeaves.org/ [Accessed 19 May 2020]

SEO BirdLife. (2012a). Abejero Europeo, LA MIGRACIÓN DE LAS AVES - SEO/BirdLife. SEO Birdlife - Sociedad Española de Ornitología. http://www.migraciondeaves.org/ [Accessed 19 May 2020]

SEO BirdLife. (2012b). Golondrina Comun, LA MIGRACIÓN DE LAS AVES - SEO/BirdLife. SEO Birdlife - Sociedad Española de Ornitología. http://www.migraciondeaves.org/ [Accessed 19 May 2020]

Softpro Kft. (2016). Hotspots revealed by PTT satellite tracking [Annex for the Mid-term report LIFE11/NAT/HU/000926/]. Conservation of the Red-footed Falcon. http://falcoproject.eu/sites/default/files/mid-term_report_2016/annex_7.2_-_digital_annex_cd_c8.pdf

Stach, R., Jakobsson, S., Kullberg, C., & Fransson, T. (2012). Geolocators reveal three consecutive wintering areas in the thrush nightingale. *Animal Migration*, 1, 1–7. https://doi.org/10.2478/ami-2012-0001

Stancliffe, P. (2011). Tagged! Bird Watching. https://www.bto.org/sites/default/files/u27/downloads/bw-nightingales.pdf

Strandberg, R., Klaassen, R. H. G., Hake, M., Olofsson, P., Thorup, K., & Alerstam, T. (2008). Complex Timing of Marsh Harrier *Circus aeruginosus* Migration Due to Pre- and Post-Migratory Movements. *Ardea*, 96(2), 159–172. https://doi.org/10.5253/078.096.0202

Strandberg, R., Klaassen, R. H. G., Olofsson, P., & Alerstam, T. (2009). Daily Travel Schedules of Adult Eurasian Hobbies *Falco subbuteo* - Variability in Flight Hours and Migration Speed Along the Route. *Ardea*, 97(3), 287–295. https://doi.org/10.5253/078.097.0304

Szép, T., Liechti, F., Nagy, K., Nagy, Z., & Hahn, S. (2017). Discovering the migration and nonbreeding areas of sand martins and house martins breeding in the Pannonian basin (central-eastern Europe). *Journal of Avian Biology*, 48(1), 114–122. https://doi.org/10.1111/jav.01339

Tanferna, A., López-Jiménez, L., Blas, J., Hiraldo, F., & Sergio, F. (2012). Different Location Sampling Frequencies by Satellite Tags Yield Different Estimates of Migration Performance: Pooling Data Requires a Common Protocol. *PLOS ONE*, 7(11), e49659. https://doi.org/10.1371/journal.pone.0049659

Thorup, K., Tøttrup, A. P., Willemoes, M., Klaassen, R. H. G., Strandberg, R., Vega, M. L., Dasari, H. P., Araújo, M. B., Wikelski, M., & Rahbek, C. (2017). Resource tracking within and across continents in long-distance bird migrants. *Science Advances*, 3(1), e1601360. https://doi.org/10.1126/sciadv.1601360

Tøttrup, A. P., Pedersen, L., & Thorup, K. (2018). Autumn migration and wintering site of a wood warbler *Phylloscopus sibilatrix* breeding in Denmark identified using geolocation. *Animal Biotelemetry*, 6(1), 15. https://doi.org/10.1186/s40317-018-0159-x

Tøttrup, A. P., Pedersen, L., Onrubia, A., Klaassen, R. H. G., & Thorup, K. (2017). Migration of red-backed shrikes from the Iberian Peninsula: Optimal or sub-optimal detour? *Journal of Avian Biology*, 48(1), 149–154. https://doi.org/10.1111/jav.01352

Trierweiler, C., Raymond H. G. Klaassen, H., D. R., Klaus-Michael, E., Jan, K., Franz, B., & J., K. B. (2014). Migratory connectivity and population-specific migration routes in a long-distance migratory bird. *Proceedings of the Royal Society B: Biological Sciences*, 281(1778), 20132897. https://doi.org/10.1098/rspb.2013.2897

Väli, Ü., & Sellis, U. (2016). Migration patterns of the Osprey *Pandion haliaetus* on the Eastern European–East African flyway. *Ostrich*, 87(1), 23–28. https://doi.org/10.2989/00306525.2015.1105319

Väli, Ü., Mirski, P., Sellis, U., Dagys, M., & Maciorowski, G. (2018). Genetic determination of migration strategies in large soaring birds: Evidence from hybrid eagles. *Proceedings of the Royal Society B: Biological Sciences*, 285(1884), 20180855. https://doi.org/10.1098/rspb.2018.0855

van Wijk, R. E., Schaub, M., Hahn, S., Juárez-García-Pelayo, N., Schäfer, B., Viktora, L., Martín-Vivaldi, M., Zischewski, M., & Bauer, S. (2018). Diverse migration strategies in hoopoes (*Upupa epops*) lead to weak spatial but strong temporal connectivity. *The Science of Nature*, 105(7), 42. https://doi.org/10.1007/s00114-018-1566-9

Vansteelant, W. M. G., Kekkonen J., & Byholm P. (2017). Wind conditions and geography shape the first outbound migration of juvenile honey buzzards and their distribution across sub-Saharan Africa. *Proceedings of the Royal Society B: Biological Sciences*, 284(1855), 20170387. https://doi.org/10.1098/rspb.2017.0387

Vansteelant, Wouter M. G., Klaassen, R., Strandberg, R., Janssens, K., T´Jollyn, F., Bouten, W., Koks, B. J., & Anselin, A. (2020). Western Marsh Harriers *Circus aeruginosus* from nearby breeding areas migrate along comparable loops, but on contrasting schedules in the West African–Eurasian flyway. *Journal of Ornithology*, 161(4), 953-965. https://doi.org/10.1007/s10336-020-01785-6

VCF (2017). Egyptian vultures with GPS transmitters. Vulture Conservation Foundation. Retrieved from https://www.4vultures.org/our-work/monitoring/egyptian-vulture-online-maps/ [Accessed 04 May 2020]

Vega, M. L., Willemoes, M., Arizaga, J., Onrubia, A., Cuenca, D., Alonso, D., Torralvo, C., Tøttrup, A. P., & Thorup, K. (2019). Migration Strategies of Iberian Breeding White-Rumped *Swifts Apus caffer*, Rufous-Tailed Scrub-Robins Cercotrichas galactotes and Bluethroats *Cyanecula svecica. Ardeola*, 66(1), 51. https://doi.org/10.13157/arla.66.1.2019.ra4

Vega, M. L., Willemoes, M., Thomson, R. L., Tolvanen, J., Rutila, J., Samaš, P., Strandberg, R., Grim, T., Fossøy, F., Stokke, B. G., & Thorup, K. (2016). First-Time Migration in Juvenile Common Cuckoos Documented by Satellite Tracking. *PLOS ONE*, 11(12), e0168940. https://doi.org/10.1371/journal.pone.0168940

Wellbrock, A. H. J., Bauch, C., Rozman, J., & Witte, K. (2017). 'Same procedure as last year?' Repeatedly tracked swifts show individual consistency in migration pattern in successive years. *Journal of Avian Biology*, 48(6), 897–903. https://doi.org/10.1111/jav.01251

Willemoes, M., Strandberg, R., Klaassen, R. H. G., Tottrup, A. P., Vardanis, Y., Howey, P. W., Thorup, K., Wikelski, M., & Alerstam, T. (2014). Narrow-Front Loop Migration in a Population of the Common Cuckoo *Cuculus canorus*, as Revealed by Satellite Telemetry. *PLOS ONE*, 9(1). https://doi.org/10.1371/journal.pone.0083515

WWGBP (2013). Autumn migration of Lesser Spotted Eagles. World Working Group in Birds of Prey. Retrieved from https://satellite-telemetry.jimdo.com/archive-1/ [Accessed 27 April 2021]

Appendix S5

Arizaga, J., Willemoes, M., Unamuno, E., Unamuno, J. M., & Thorup, K. (2015). Following year-round movements in Barn Swallows using geolocators: Could breeding pairs remain together during the winter? *Bird Study*, *62*(1), 141–145. https://doi.org/10.1080/00063657.2014.998623

BirdLife International and Handbook of the Birds of the World (2018). *Bird species distribution maps of the world. Version 2018.1*. http://datazone.birdlife.org/species/requestdis.

Briedis, M., Hahn, S., Gustafsson, L., Henshaw, I., Träff, J., Král, M., & Adamík, P. (2016). Breeding latitude leads to different temporal but not spatial organization of the annual cycle in a long-distance migrant. *Journal of Avian Biology*, *47*(6), 743–748. https://doi.org/10.1111/jav.01002

Briedis, M., Krist, M., Král, M., Voigt, C. C., & Adamík, P. (2018). Linking events throughout the annual cycle in a migratory bird—Nonbreeding period buffers accumulation of carry-over effects. *Behavioral Ecology and Sociobiology*, *72*(6), 93. https://doi.org/10.1007/s00265-018-2509-3

Briedis, M., Kurlavičius, P., Mackevičienė, R., Vaišvilienė, R., & Hahn, S. (2018). Loop migration, induced by seasonally different flyway use, in Northern European Barn Swallows. *Journal of Ornithology*, *159*(4), 885–891. https://doi.org/10.1007/s10336-018-1560-1

BTO (2018). Nightjar Tracking Project. BTO - British Trust for Ornithology. Retrieved from https://www.bto.org/our-science/topics/tracking/tracking-studies/nightjars [Accessed 28 April 2021]

Calenge, C. (2006). The package adehabitat for the R software: Tool for the analysis of space and habitat use by animals. *Ecological Modelling*, *197*, 1035.

Cramp, S. (1985). Handbook of the Birds of Europe, the Middle East, and North Africa: The Birds of the Western Palearctic Vol. IV. 620-637. Oxford University Press: Oxford.

Cramp, S. (1988). Handbook of the Birds of Europe, the Middle East, and North Africa: The Birds of the Western Palearctic Vol. IV. 620-637. Oxford University Press: Oxford.

Dhanjal-Adams, K. L., Bauer, S., Emmenegger, T., Hahn, S., Lisovski, S., & Liechti, F. (2018). Spatiotemporal Group Dynamics in a Long-Distance Migratory Bird. *Current Biology*, *28*(17), 2824-2830.e3. https://doi.org/10.1016/j.cub.2018.06.054

Evens, R., Conway, G. J., Henderson, I. G., Cresswell, B., Jiguet, F., Moussy, C., Sénécal, D., Witters, N., Beenaerts, N., & Artois, T. (2017). Migratory pathways, stopover zones and wintering destinations of Western European Nightjars *Caprimulgus europaeus*. *Ibis*, *159*(3), 680–686. https://doi.org/10.1111/ibi.12469

Hahn, S., Alves, J. A., Bedev, K., Costa, J. S., Emmenegger, T., Schulze, M., Tamm, P., Zehtindjiev, P., & Dhanjal-Adams, K. L. (2020). Range-wide migration corridors and nonbreeding areas of a northward expanding Afro-Palaearctic migrant, the European Bee-eater *Merops apiaster*. Ibis, 162(2), 345–355. https://doi.org/10.1111/ibi.12752

Jacobsen, L. B., Jensen, N. O., Willemoes, M., Hansen, L., Desholm, M., Fox, A. D., Tøttrup, A. P., & Thorup, K. (2017). Annual spatiotemporal migration schedules in three larger insectivorous birds: European nightjar, common swift and common cuckoo. *Animal Biotelemetry*, *5*(1), 4. https://doi.org/10.1186/s40317-017-0119-x

Klvaňa, P., Cepák, J., Munclinger, P., Michálková, R., Tomášek, O., & Albrecht, T. (2018). Around the Mediterranean: An extreme example of loop migration in a long-distance migratory passerine. *Journal of Avian Biology*, *49*(2), jav-01595. https://doi.org/10.1111/jav.01595

Liechti, F., Scandolara, C., Rubolini, D., Ambrosini, R., Korner-Nievergelt, F., Hahn, S., Lardelli, R., Romano, M., Caprioli, M., Romano, A., Sicurella, B., & Saino, N. (2015). Timing of migration and residence areas during the nonbreeding period of barn swallows *Hirundo rustica* in relation to sex and population. *Journal of Avian Biology*, *46*(3), 254–265. https://doi.org/10.1111/jav.00485

López-Calderón, C. (2019). *Linking different worlds: Migration ecology in two species of hirundines* [Universidad de Sevilla]. https://idus.us.es/handle/11441/86506

Norevik, G., Åkesson, S., Andersson, A., Bäckman, J., & Hedenström, A. (2019). The lunar cycle drives migration of a nocturnal bird. *PLOS Biology*, *17*(10), e3000456. https://doi.org/10.1371/journal.pbio.3000456

Norevik, G., Åkesson, S., & Hedenström, A. (2017). Migration strategies and annual space-use in an Afro-Palaearctic aerial insectivore – the European nightjar *Caprimulgus europaeus*. *Journal of Avian Biology*, *48*(5), 738–747. https://doi.org/10.1111/jav.01071

Pebesma, E. (2018). Simple Features for R: Standardized Support for Spatial Vector Data. *The R Journal*, *10*(1), 439. https://doi.org/10.32614/RJ-2018-009

R Core Team. (2021). *R: A Language and Environment for Statistical Computing*. R Foundation for Statistical Computing. https://www.R-project.org/

SEO/BirdLife. (2012). Golondrina Comun, LA MIGRACIÓN DE LAS AVES - SEO/BirdLife. SEO Birdlife - Sociedad Española de Ornitología. http://www.migraciondeaves.org/ [Accessed 19 May 2020]

Appendix S7

BirdLife International & HBW. (2018). Bird species distribution maps of the world. Version 2018.1. http://datazone.birdlife.org/species/requestdis.

Morrick, Z. N., Lilleyman, A., Fuller, R. A., Bush, R., Coleman, J. T., Garnett, S. T., Gerasimov, Y. N., Jessop, R., Ma, Z., Maglio, G., Minton, C. D. T., Syroechkovskiy, E., & Woodworth, B. K. (2021). Differential population trends align with migratory connectivity in an endangered shorebird. Conservation Science and Practice. https://doi.org/10.1111/csp2.594

van Wijk, R. E., Schaub, M., Hahn, S., Juárez-García-Pelayo, N., Schäfer, B., Viktora, L., Martín-Vivaldi, M., Zischewski, M., & Bauer, S. (2018). Diverse migration strategies in hoopoes (*Upupa epops*) lead to weak spatial but strong temporal connectivity. The Science of Nature, 105(7), 42. https://doi.org/10.1007/s00114-018-1566-9
