# Supplementary material for: Connectivity between countries established by landbirds and raptors migrating along the African–Eurasian flyway
Source: Conserv Biol. 2022 Dec 15;37(1):e14002. doi: 10.1111/cobi.14002 (PMC10107209; doi:10.1111/cobi.14002)

Connectivity between countries established by landbirds and raptors migrating along the African-Eurasian flyway

Guilherme et al. (2022). Conservation Biology. DOI: 10.1111/cobi.14002

Appendix S10.

Mapping country-level connectivity for all countries in Europe and sub-Saharan Africa.

The following 65 figures synthesize country-level connectivity, as reveled by the migratory links obtained from the reviewed studies, for each country in Europe and sub-Saharan Africa, shown separately for landbirds and raptors. For each country, we present the list of all migratory links by decreasing order of strength, indicating in each case the species creating the link and the country it connects to. The maps represent how the migratory links connect the countries in Europe to countries in sub-Saharan Africa.

A migratory link represents the connection between two countries established by birds from a population as they migrate from a European breeding country to a sub-Saharan African nonbreeding country. The strength of a migratory link represents the proportion of individuals in a population that spend the nonbreeding season in a given country in sub-Saharan Africa, and was estimated from the distribution of migration records (observed and interpolated) among the migratory links in each population (details in Appendices S4, S7 & S9).

For analysis, we merged the smaller sub-Saharan countries (those with latitudinal and/or longitudinal extents <200 km) to their neighboring countries: Gambia to Senegal; Togo to Benin; Equatorial Guinea to Gabon; Burundi to Rwanda; Djibouti to Ethiopia; Swaziland to South Africa; and Angola's enclave Cabinda to the Republic of Congo (details in Appendix S1).

List of Figures

Europe

Figure S10.1: Portugal 4
Figure S10.2: Spain 5
Figure S10.3: France 6
Figure S10.4: United Kingdom 7
Figure S10.5: Belgium 8

Figure S10.6: Netherlands 9
Figure S10.7: Italy 10
Figure S10.8: Switzerland 11
Figure S10.9: Germany 12
Figure S10.10: Czech Republic 13
Figure S10.11: Poland 14
Figure S10.12: Slovakia 15
Figure S10.13: Hungary 16
Figure S10.14: Montenegro 17
Figure S10.15: Denmark 18
Figure S10.16: Norway 19
Figure S10.17: Sweden 20
Figure S10.18: Finland 21
Figure S10.19: Estonia 22
Figure S10.20: Latvia 23
Figure S10.21: Lithuania 24
Figure S10.22: Belarus 25
Figure S10.23: Ukraine 26
Figure S10.24: Romania 27
Figure S10.25: Bulgaria 28
Figure S10.26: Greece 29
Figure S10.27: Cyprus 30
Figure S10.28: Turkey 31

Sub-Saharan Africa

Figure S10.29: Mauritania 32
Figure S10.30: Senegal & Gambia 33
Figure S10.31: Guinea-Bissau 34
Figure S10.32: Guinea 35
Figure S10.33: Sierra Leone 36
Figure S10.34: Liberia 37
Figure S10.35: Mali 38
Figure S10.36: Côte d’Ivoire 39
Figure S10.37: Burkina Faso 40
Figure S10.38: Ghana 41
Figure S10.39: Benin & Togo 42
Figure S10.40: Nigeria 43
Figure S10.41: Niger 44

Figure S10.42: Cameroon 45
Figure S10.43: Chad........ 46
Figure S10.44: Central African Republic........ 47
Figure S10.45: Gabon & Equatorial Guinea 48
Figure S10.46: Congo & Cabinda (Angola)........ 49
Figure S10.47: Democratic Republic of Congo 50
Figure S10.48: Angola 51
Figure S10.49: Namibia 52
Figure S10.50: South Africa & Swaziland........ 53
Figure S10.51: Lesotho 54
Figure S10.52: Mozambique........ 55
Figure S10.53: Botswana 56
Figure S10.54: Zimbabwe........ 57
Figure S10.55: Zambia........ 58
Figure S10.56: Malawi........ 59
Figure S10.57: Tanzania 60
Figure S10.58: Rwanda & Burundi........ 61
Figure S10.59: Uganda........ 62
Figure S10.60: Kenya........ 63
Figure S10.61: Somalia........ 64
Figure S10.62: Ethiopia & Djibouti 65
Figure S10.63: South Sudan........ 66
Figure S10.64: Sudan........ 67
Figure S10.65: Madagascar........ 68

Figure S10.1: Portugal

Mapping country-level connectivity for Portugal, separately for (a) landbirds and (b) raptors. For each group, we present the list of all migratory links by decreasing order of strength (with respective number of tracked individuals in parenthesis), indicating in each case the species creating the link and the country it connects to. Maps represent how the migratory links (observed: solid lines; inferred: dotted lines) connect the countries in Europe (in green) to countries in sub-Saharan Africa (in blue).

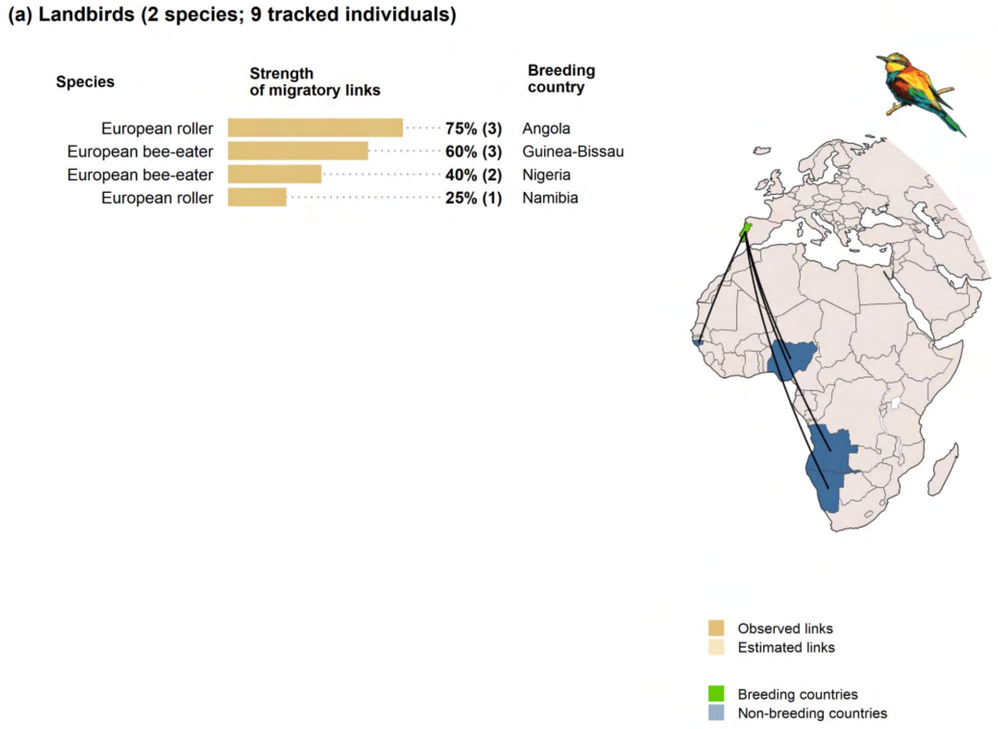

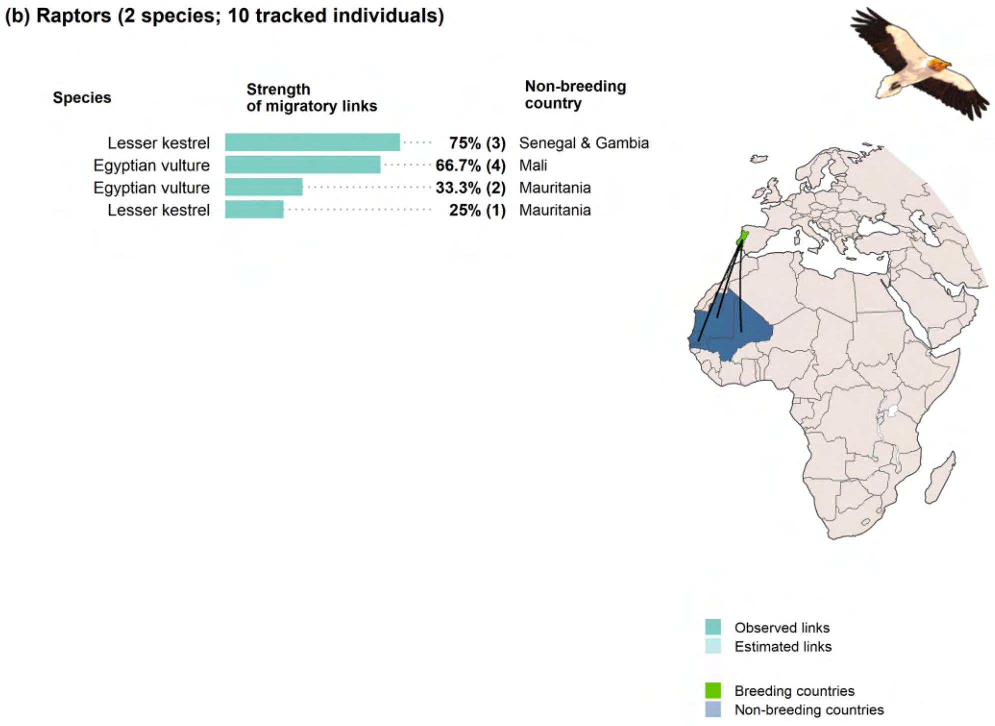

Figure S10.2: Spain

Mapping country-level connectivity for Spain, separately for (a) landbirds and (b) raptors. For each group, we present the list of all migratory links by decreasing order of strength (with respective number of tracked individuals in parenthesis), indicating in each case the species creating the link and the country it connects to. Maps represent how the migratory links (observed: solid lines; inferred: dotted lines) connect the countries in Europe (in green) to countries in sub-Saharan Africa (in blue).

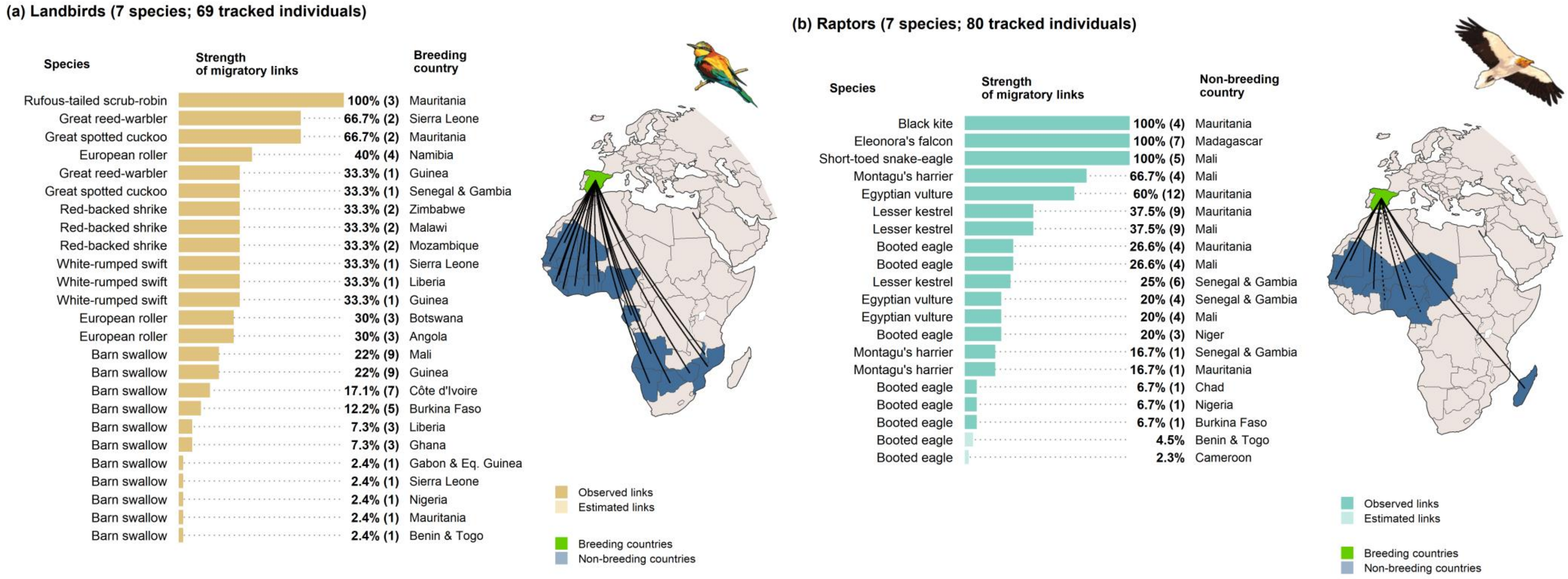

Figure S10.3: France

Mapping country-level connectivity for France, separately for (a) landbirds and (b) raptors. For each group, we present the list of all migratory links by decreasing order of strength (with respective number of tracked individuals in parenthesis), indicating in each case the species creating the link and the country it connects to. Maps represent how the migratory links (observed: solid lines; inferred: dotted lines) connect the countries in Europe (in green) to countries in sub-Saharan Africa (in blue).

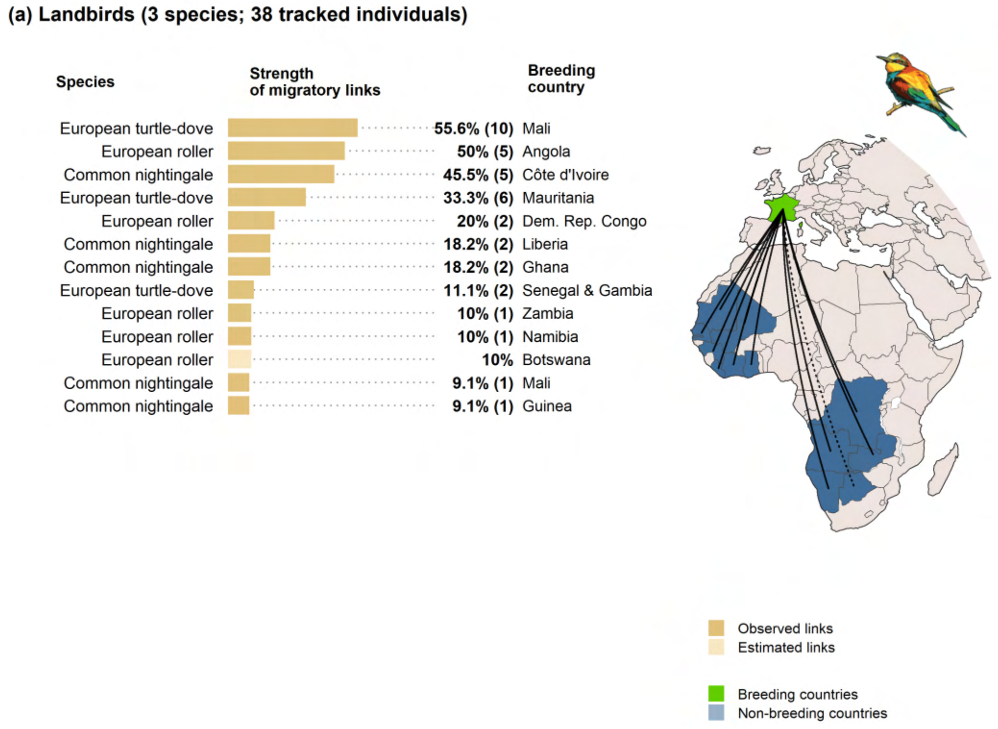

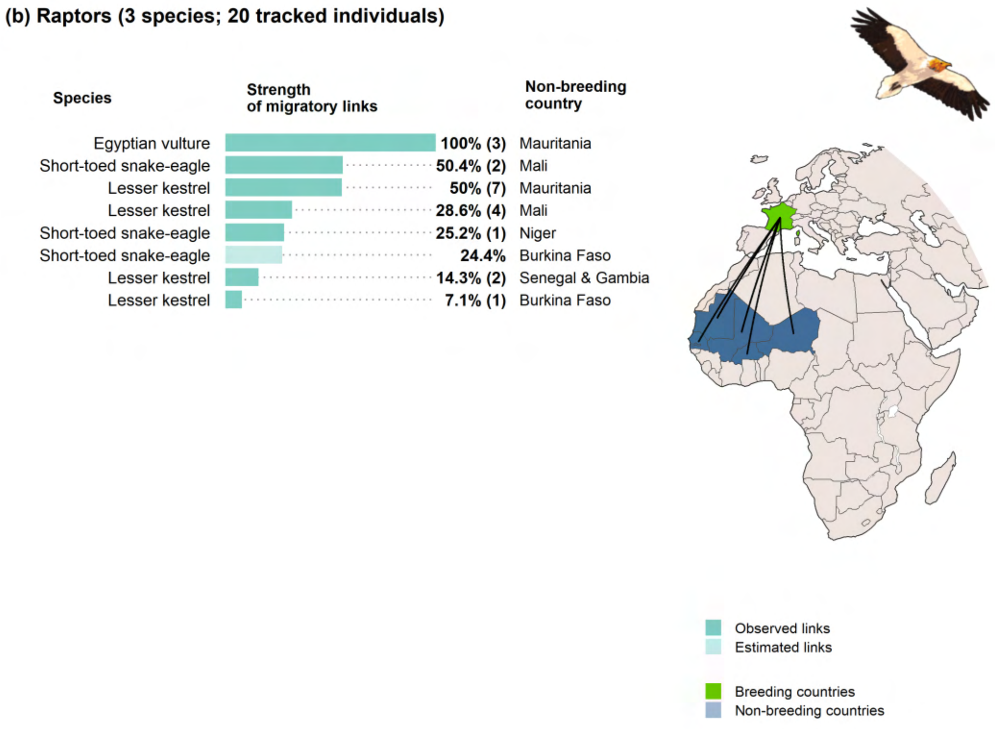

Figure S10.4: United Kingdom

Mapping country-level connectivity for United Kingdom, separately for (a) landbirds and (b) raptors. For each group, we present the list of all migratory links by decreasing order of strength (with respective number of tracked individuals in parenthesis), indicating in each case the species creating the link and the country it connects to. Maps represent how the migratory links (observed: solid lines; inferred: dotted lines) connect the countries in Europe (in green) to countries in sub-Saharan Africa (in blue).

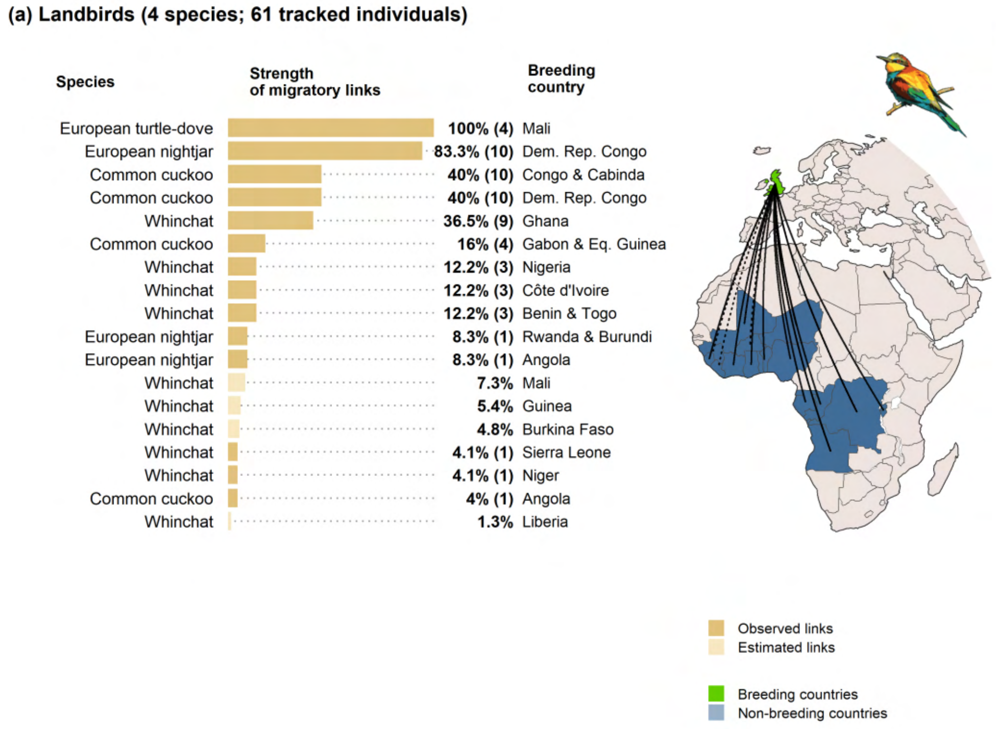

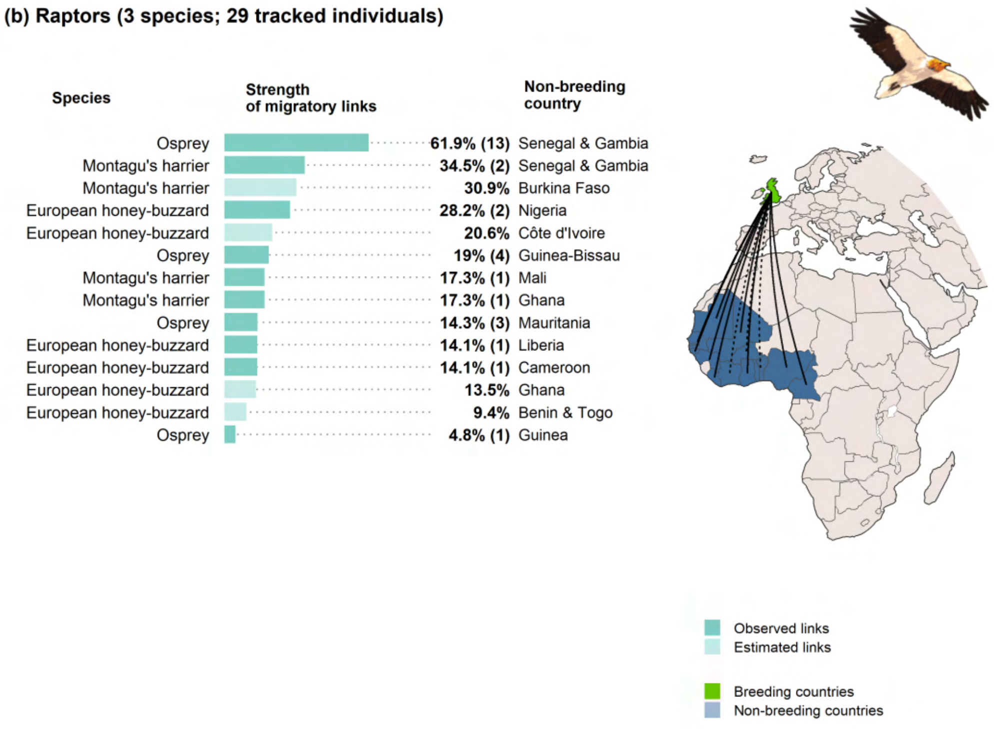

Figure S10.5: Belgium

Mapping country-level connectivity for Belgium, separately for (a) landbirds and (b) raptors. For each group, we present the list of all migratory links by decreasing order of strength (with respective number of tracked individuals in parenthesis), indicating in each case the species creating the link and the country it connects to. Maps represent how the migratory links (observed: solid lines; inferred: dotted lines) connect the countries in Europe (in green) to countries in sub-Saharan Africa (in blue).

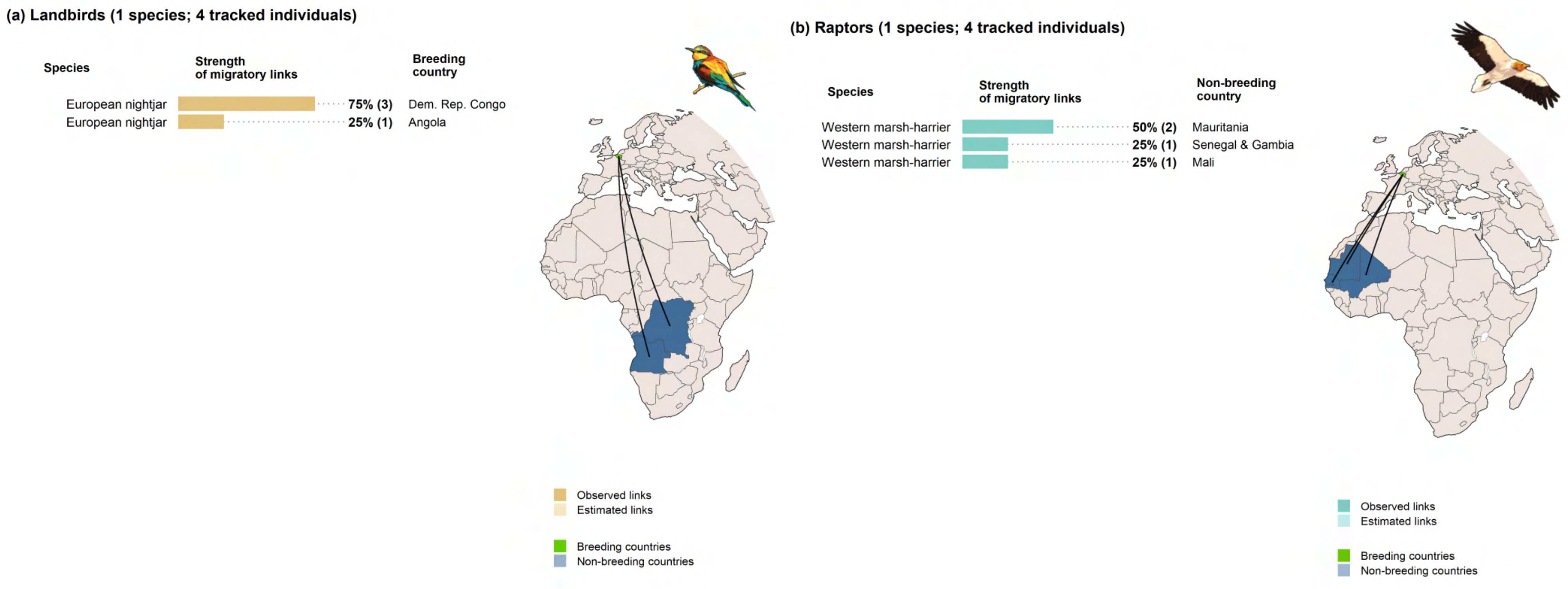

Figure S10.6: Netherlands

Mapping country-level connectivity for the Netherlands, separately for (a) landbirds and (b) raptors. For each group, we present the list of all migratory links by decreasing order of strength (with respective number of tracked individuals in parenthesis), indicating in each case the species creating the link and the country it connects to. Maps represent how the migratory links (observed: solid lines; inferred: dotted lines) connect the countries in Europe (in green) to countries in sub-Saharan Africa (in blue).

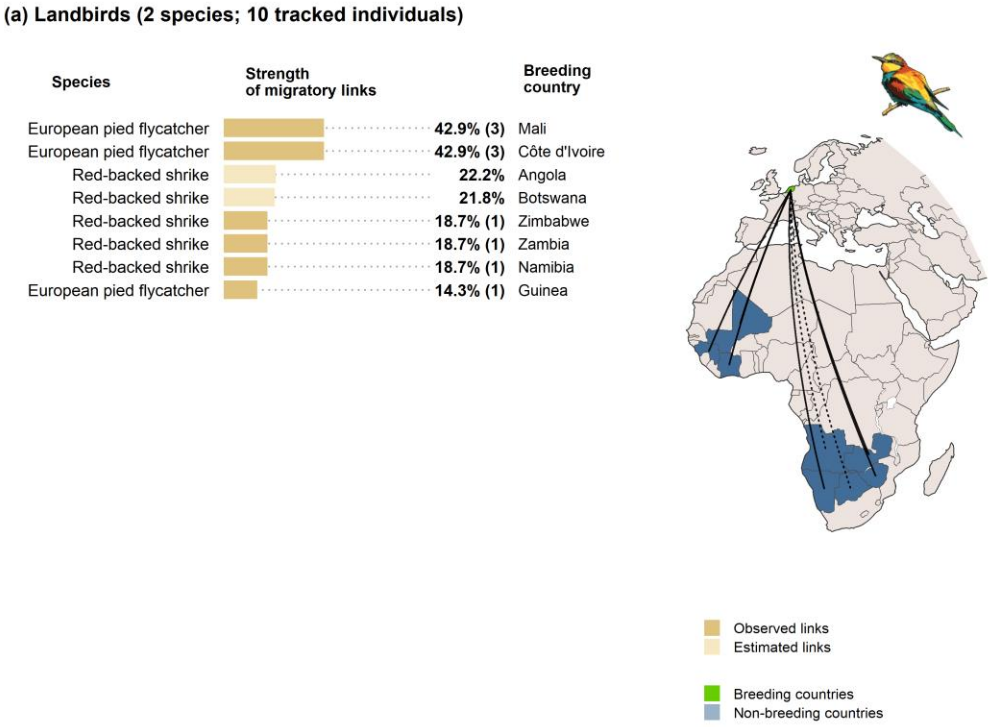

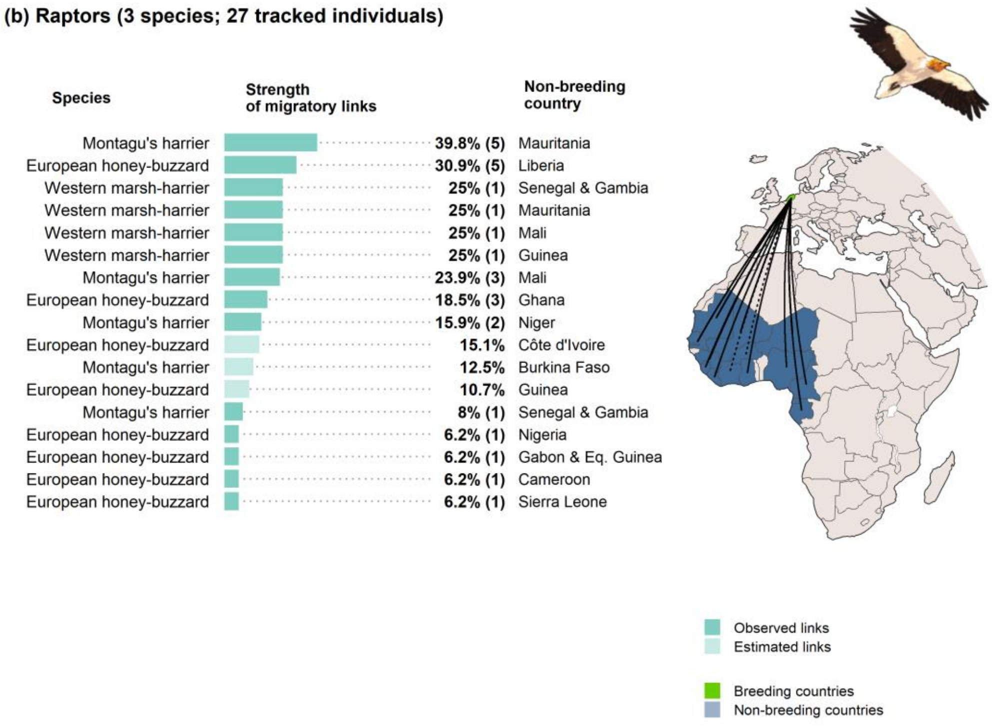

Figure S10.7: Italy

Mapping country-level connectivity for Italy, separately for (a) landbirds and (b) raptors. For each group, we present the list of all migratory links by decreasing order of strength (with respective number of tracked individuals in parenthesis), indicating in each case the species creating the link and the country it connects to. Maps represent how the migratory links (observed: solid lines; inferred: dotted lines) connect the countries in Europe (in green) to countries in sub-Saharan Africa (in blue).

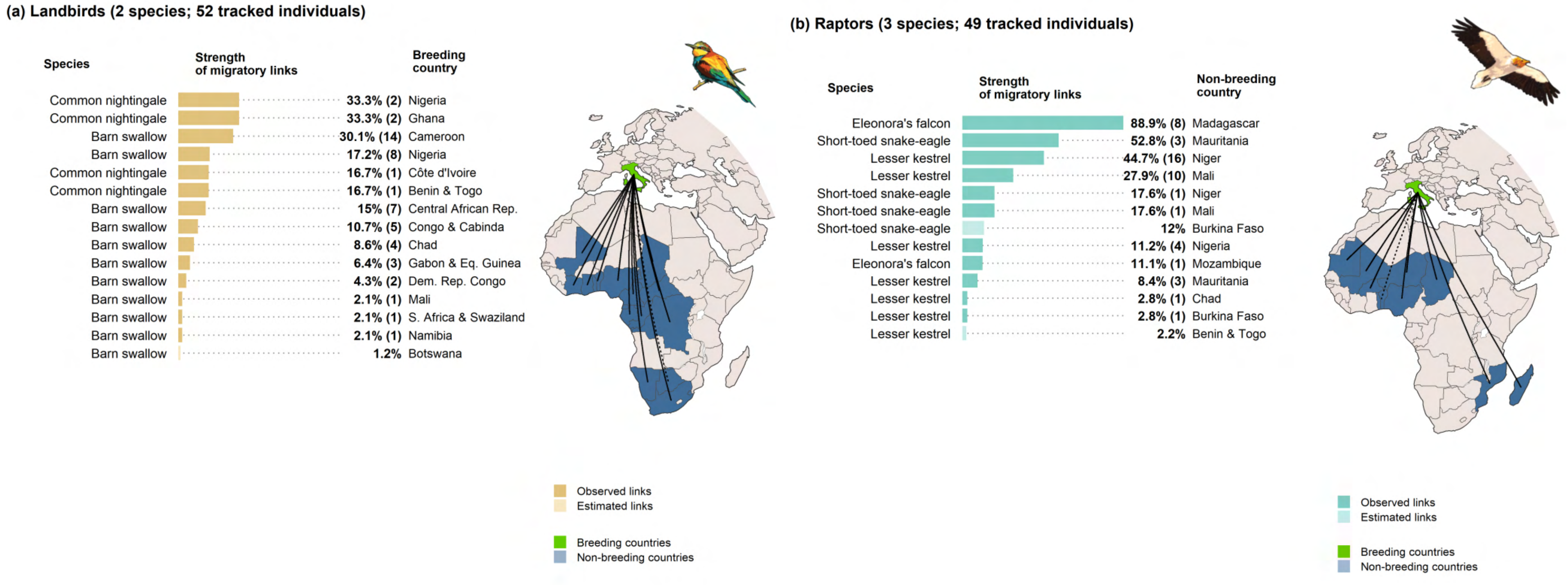

Figure S10.8: Switzerland

Mapping country-level connectivity for Switzerland, separately for (a) landbirds and (b) raptors. For each group, we present the list of all migratory links by decreasing order of strength (with respective number of tracked individuals in parenthesis), indicating in each case the species creating the link and the country it connects to. Maps represent how the migratory links (observed: solid lines; inferred: dotted lines) connect the countries in Europe (in green) to countries in sub-Saharan Africa (in blue).

(a) Landbirds (5 species; 77 tracked individuals)

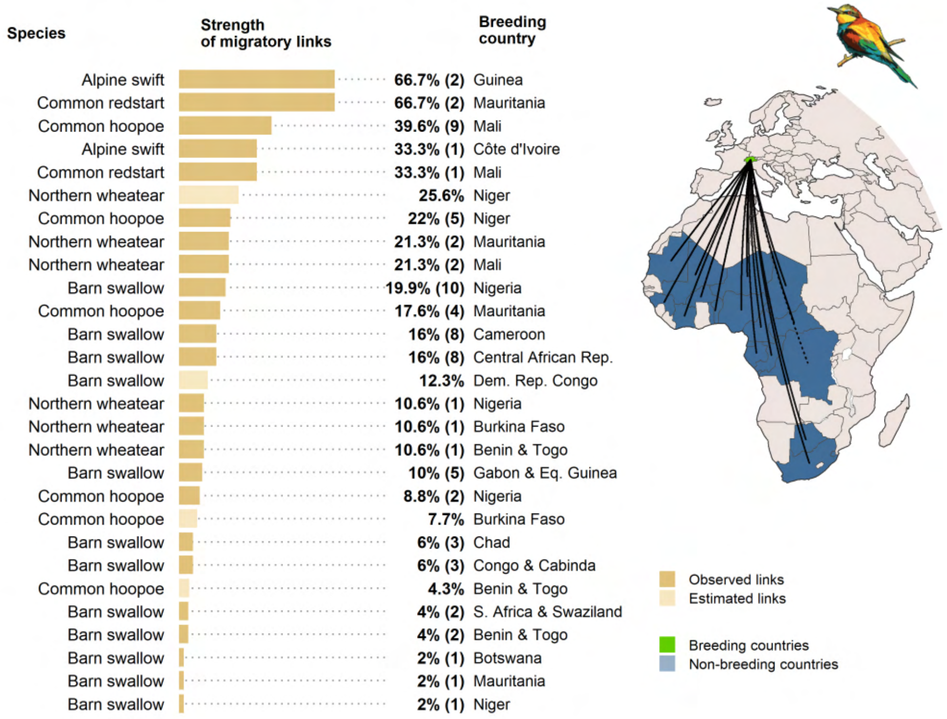

(b) Raptors (0 species tracked)

Figure S10.9: Germany

Mapping country-level connectivity for Germany, separately for (a) landbirds and (b) raptors. For each group, we present the list of all migratory links by decreasing order of strength (with respective number of tracked individuals in parenthesis), indicating in each case the species creating the link and the country it connects to. Maps represent how the migratory links (observed: solid lines; inferred: dotted lines) connect the countries in Europe (in green) to countries in sub-Saharan Africa (in blue).

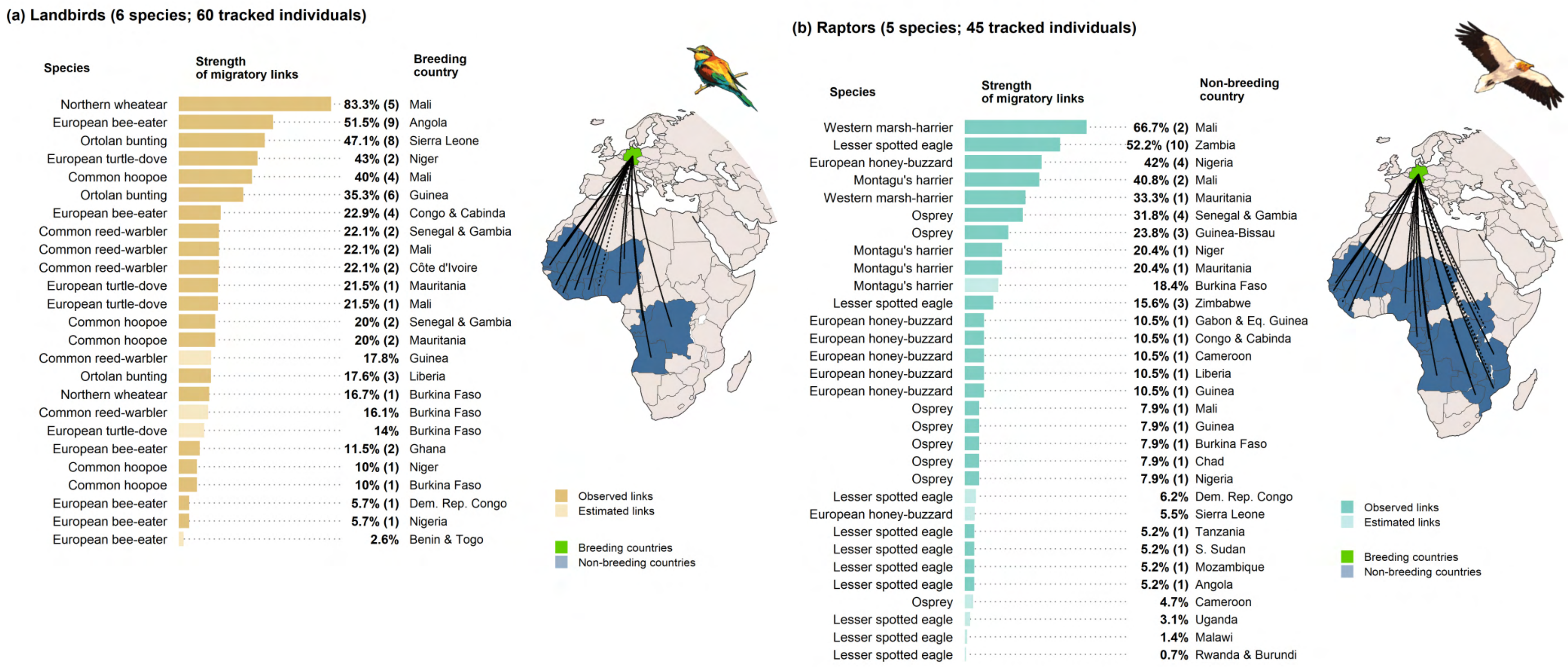

Figure S10.10: Czech Republic

Mapping country-level connectivity for Czech Republic, separately for (a) landbirds and (b) raptors. For each group, we present the list of all migratory links by decreasing order of strength (with respective number of tracked individuals in parenthesis), indicating in each case the species creating the link and the country it connects to. Maps represent how the migratory links (observed: solid lines; inferred: dotted lines) connect the countries in Europe (in green) to countries in sub-Saharan Africa (in blue).

(a) Landbirds (6 species; 112 tracked individuals)

Species	Strength of migratory links	Breeding country
Common hoopoe	50% (2)	Mauritania
Common hoopoe	50% (2)	Mali
Tawny pipit	50% (6)	Mauritania
Barn swallow	47% (10)	Dem. Rep. Congo
Tawny pipit	41.7% (5)	Mali
Great reed-warbler	38.6% (12)	Nigeria
Collared flycatcher	30.8% (12)	Zambia
Collared flycatcher	28.2% (11)	Dem. Rep. Congo
Common reed-warbler	21.5% (3)	Mali
Collared flycatcher	17.9% (7)	Botswana
Great reed-warbler	16.1% (5)	Côte d'Ivoire
Collared flycatcher	15.4% (6)	Angola
Common reed-warbler	14.4% (2)	Sierra Leone
Great reed-warbler	12.9% (4)	Cameroon
Common reed-warbler	10.6%	Côte d'Ivoire
Great reed-warbler	9.6% (3)	Ghana
Barn swallow	9.4% (2)	S. Africa & Swaziland
Barn swallow	9.4% (2)	Botswana
Common reed-warbler	9%	Burkina Faso
Tawny pipit	8.3% (1)	Senegal & Gambia
Common reed-warbler	8%	Guinea
Common reed-warbler	7.8%	Ghana
Common reed-warbler	7.2% (1)	Chad
Common reed-warbler	7.2% (1)	Cameroon
Common reed-warbler	7.2% (1)	Niger
Common reed-warbler	7.2% (1)	Liberia
Great reed-warbler	6.4% (2)	Benin & Togo
Barn swallow	6.1%	Namibia
Collared flycatcher	5.1% (2)	Namibia
Barn swallow	4.7% (1)	Zambia
Barn swallow	4.7% (1)	S. Sudan
Barn swallow	4.7% (1)	Congo & Cabinda
Barn swallow	4.7% (1)	Central African Rep.
Barn swallow	4.7% (1)	Angola
Great reed-warbler	4.1%	Central African Rep.
Barn swallow	3.6%	Zimbabwe
Great reed-warbler	3.2% (1)	Gabon & Eq. Guinea
Great reed-warbler	3.2% (1)	Dem. Rep. Congo
Great reed-warbler	3.2% (1)	Burkina Faso
Great reed-warbler	2.7%	Congo & Cabinda
Collared flycatcher	2.6% (1)	Zimbabwe
Barn swallow	1%	Rwanda & Burundi

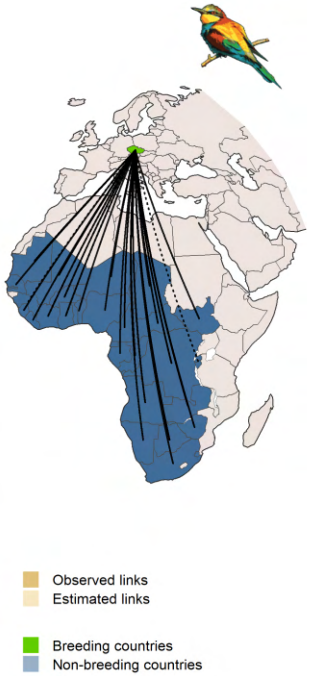

(b) Raptors (2 species; 12 tracked individuals)

Species	Strength of migratory links	Non-breeding country
Western marsh-harrier	100% (4)	Mali
Black kite	50% (4)	Senegal & Gambia
Black kite	25% (2)	Ghana
Black kite	12.5% (1)	Nigeria
Black kite	12.5% (1)	Uganda

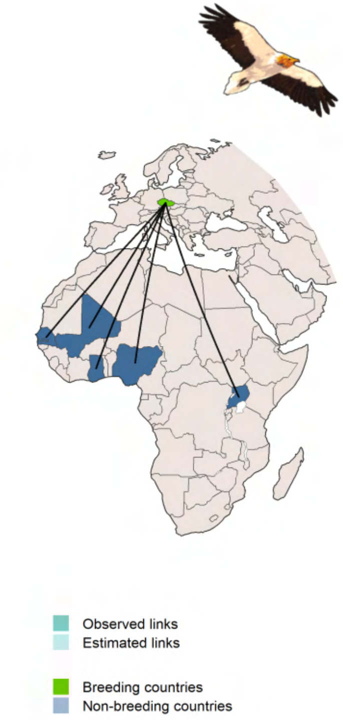

Figure S10.11: Poland

Mapping country-level connectivity for Poland, separately for (a) landbirds and (b) raptors. For each group, we present the list of all migratory links by decreasing order of strength (with respective number of tracked individuals in parenthesis), indicating in each case the species creating the link and the country it connects to. Maps represent how the migratory links (observed: solid lines; inferred: dotted lines) connect the countries in Europe (in green) to countries in sub-Saharan Africa (in blue).

(a) Landbirds (0 species tracked)

(b) Raptors (4 species; 19 tracked individuals)

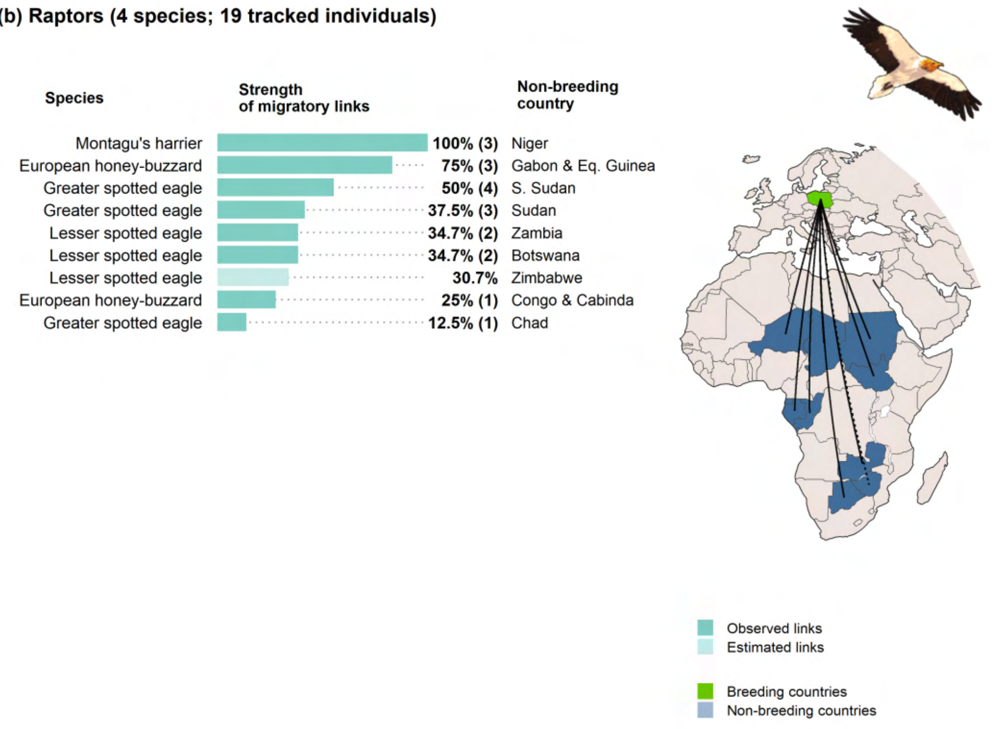

Figure S10.12: Slovakia

Mapping country-level connectivity for Slovakia, separately for (a) landbirds and (b) raptors. For each group, we present the list of all migratory links by decreasing order of strength (with respective number of tracked individuals in parenthesis), indicating in each case the species creating the link and the country it connects to. Maps represent how the migratory links (observed: solid lines; inferred: dotted lines) connect the countries in Europe (in green) to countries in sub-Saharan Africa (in blue).

(a) Landbirds (0 species tracked)

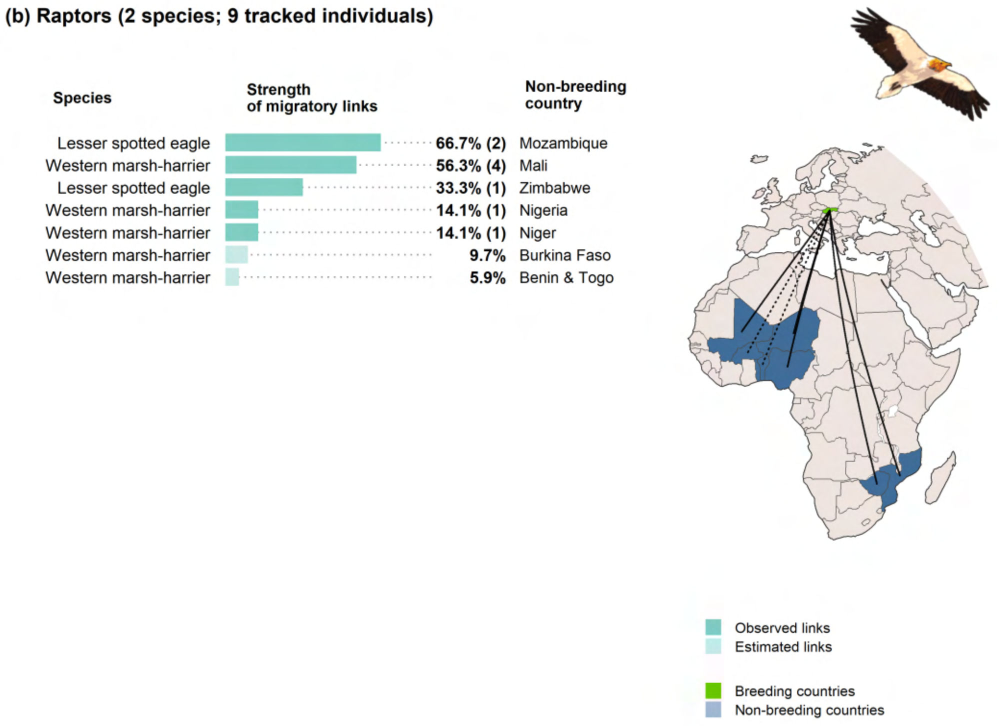

Figure S10.13: Hungary

Mapping country-level connectivity for Hungary, separately for (a) landbirds and (b) raptors. For each group, we present the list of all migratory links by decreasing order of strength (with respective number of tracked individuals in parenthesis), indicating in each case the species creating the link and the country it connects to. Maps represent how the migratory links (observed: solid lines; inferred: dotted lines) connect the countries in Europe (in green) to countries in sub-Saharan Africa (in blue).

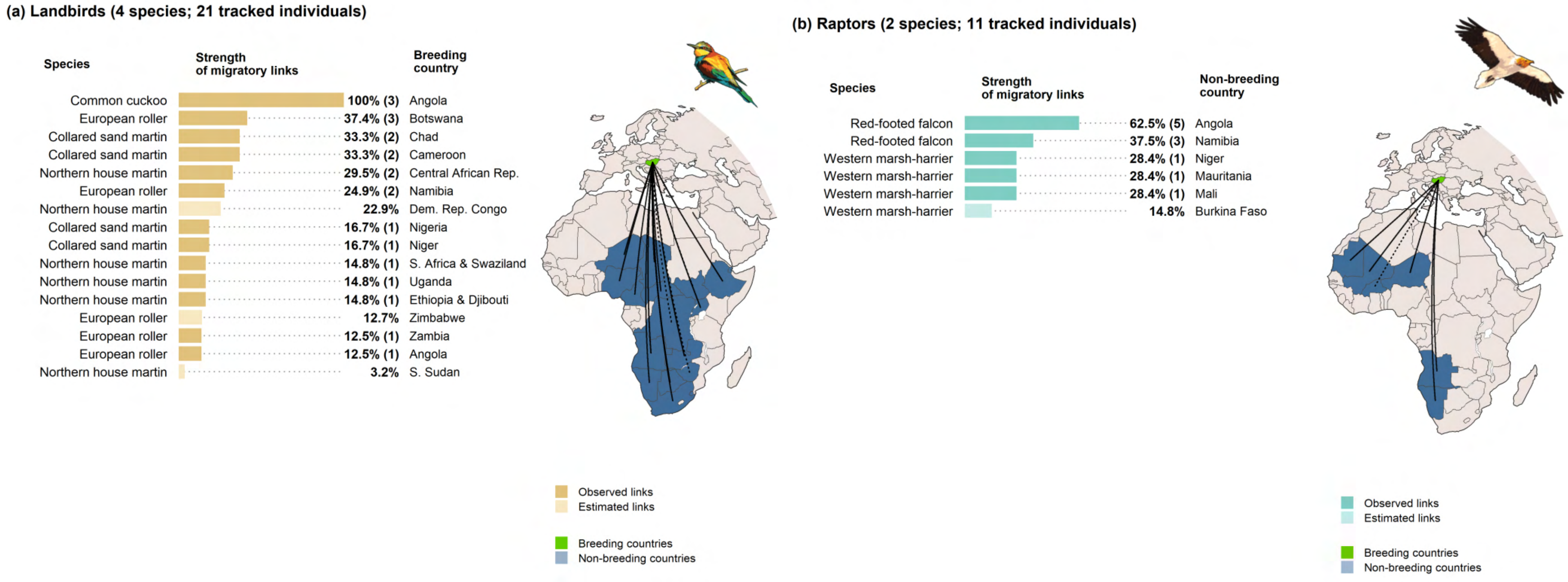

Figure S10.14: Montenegro

Mapping country-level connectivity for Montenegro, separately for (a) landbirds and (b) raptors. For each group, we present the list of all migratory links by decreasing order of strength (with respective number of tracked individuals in parenthesis), indicating in each case the species creating the link and the country it connects to. Maps represent how the migratory links (observed: solid lines; inferred: dotted lines) connect the countries in Europe (in green) to countries in sub-Saharan Africa (in blue).

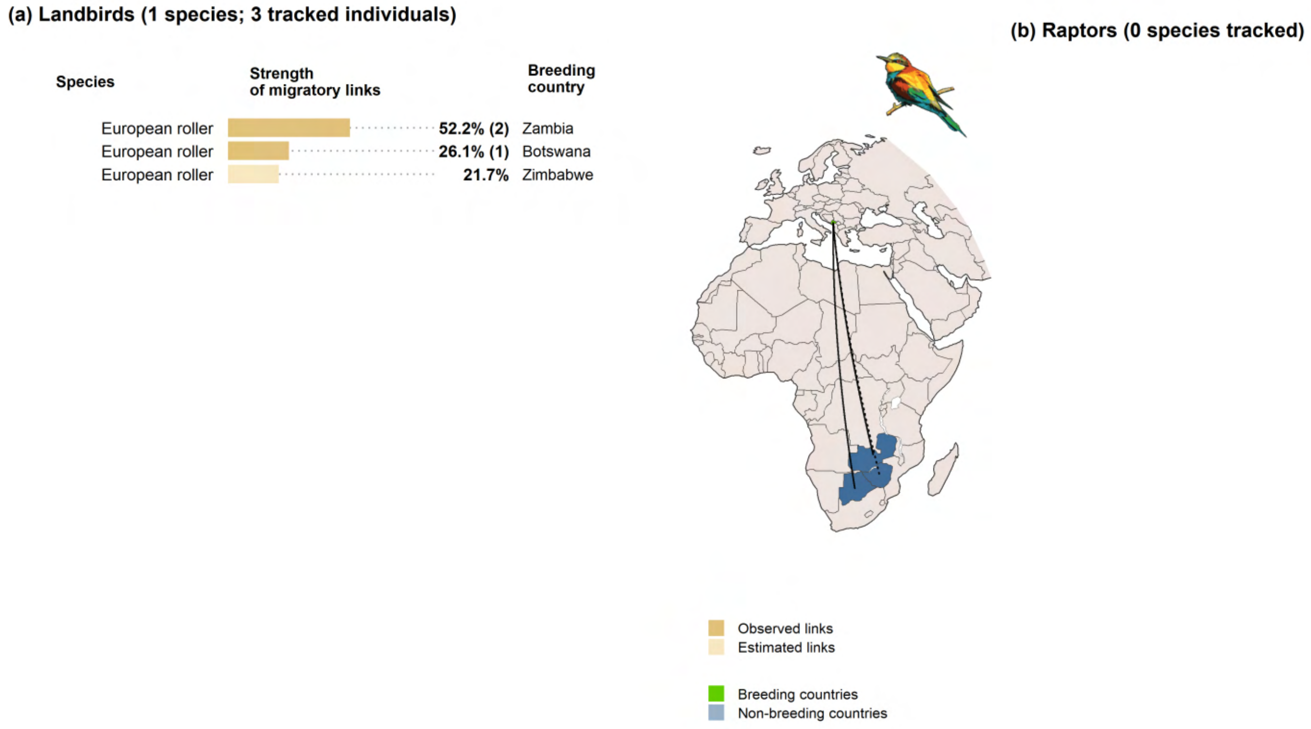

Figure S10.15: Denmark

Mapping country-level connectivity for Denmark, separately for (a) landbirds and (b) raptors. For each group, we present the list of all migratory links by decreasing order of strength (with respective number of tracked individuals in parenthesis), indicating in each case the species creating the link and the country it connects to. Maps represent how the migratory links (observed: solid lines; inferred: dotted lines) connect the countries in Europe (in green) to countries in sub-Saharan Africa (in blue).

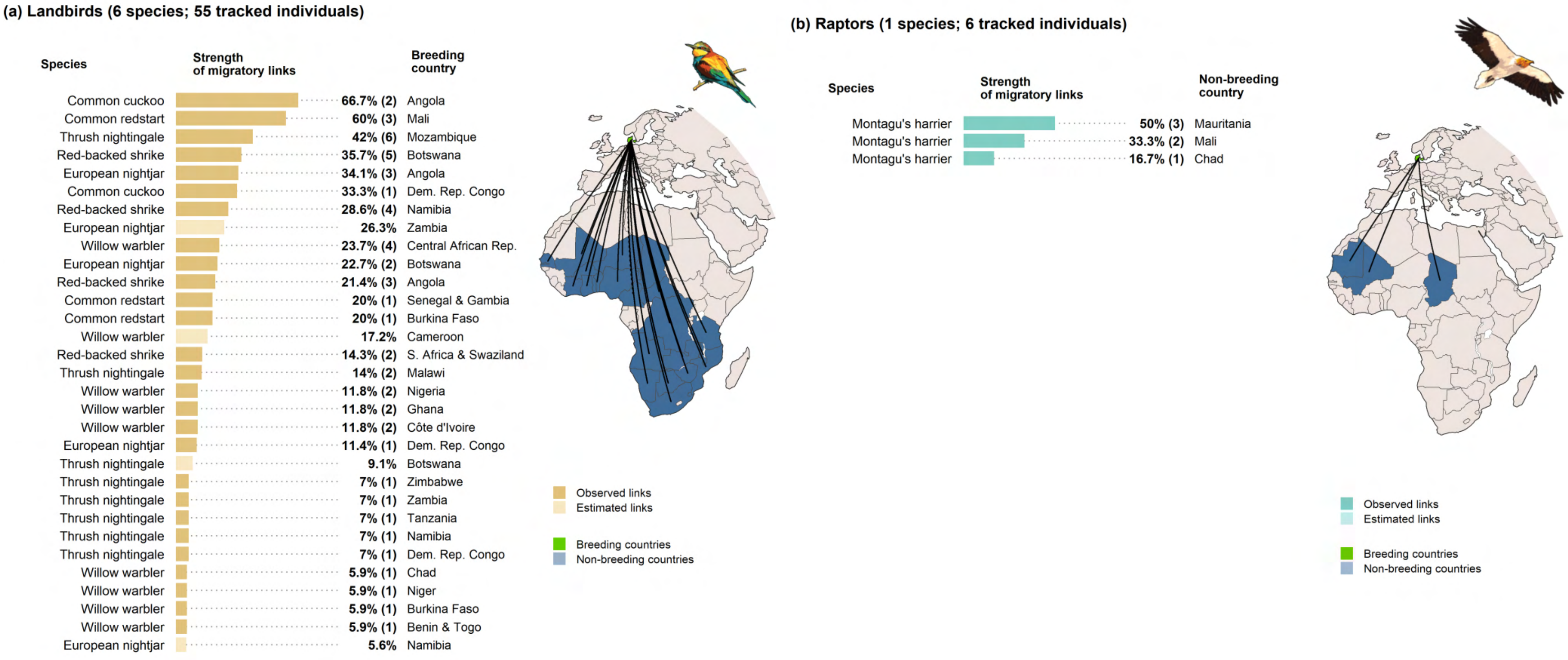

Figure S10.16: Norway

Mapping country-level connectivity for Norway, separately for (a) landbirds and (b) raptors. For each group, we present the list of all migratory links by decreasing order of strength (with respective number of tracked individuals in parenthesis), indicating in each case the species creating the link and the country it connects to. Maps represent how the migratory links (observed: solid lines; inferred: dotted lines) connect the countries in Europe (in green) to countries in sub-Saharan Africa (in blue).

(a) Landbirds (0 species tracked)

(b) Raptors (1 species; 5 tracked individuals)

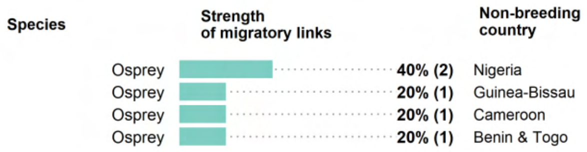

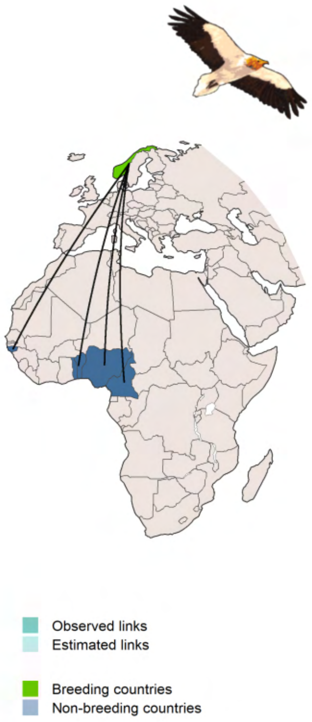

Figure S10.17: Sweden

Mapping country-level connectivity for Sweden, separately for (a) landbirds and (b) raptors. For each group, we present the list of all migratory links by decreasing order of strength (with respective number of tracked individuals in parenthesis), indicating in each case the species creating the link and the country it connects to. Maps represent how the migratory links (observed: solid lines; inferred: dotted lines) connect the countries in Europe (in green) to countries in sub-Saharan Africa (in blue).

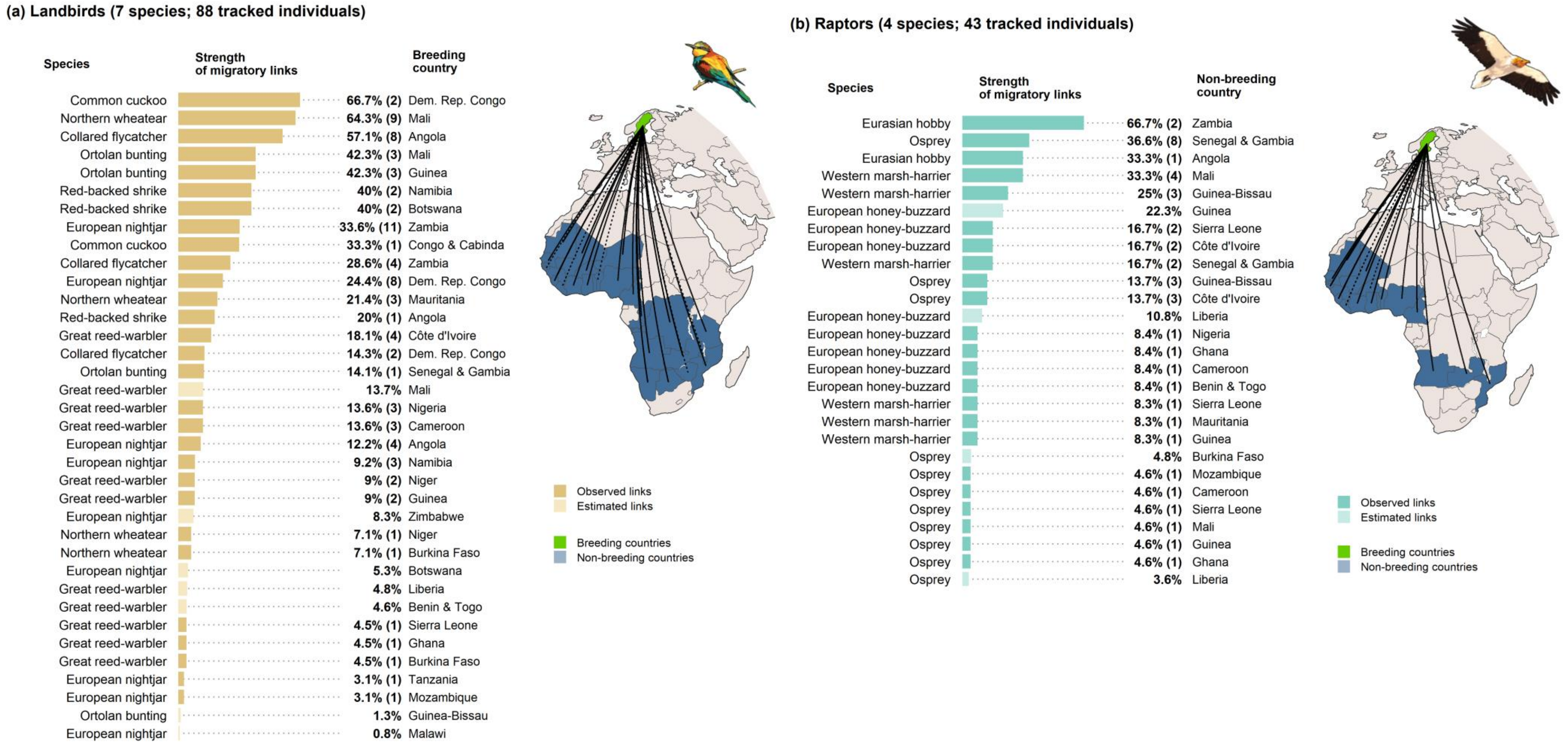

Figure S10.18: Finland

Mapping country-level connectivity for Finland, separately for (a) landbirds and (b) raptors. For each group, we present the list of all migratory links by decreasing order of strength (with respective number of tracked individuals in parenthesis), indicating in each case the species creating the link and the country it connects to. Maps represent how the migratory links (observed: solid lines; inferred: dotted lines) connect the countries in Europe (in green) to countries in sub-Saharan Africa (in blue).

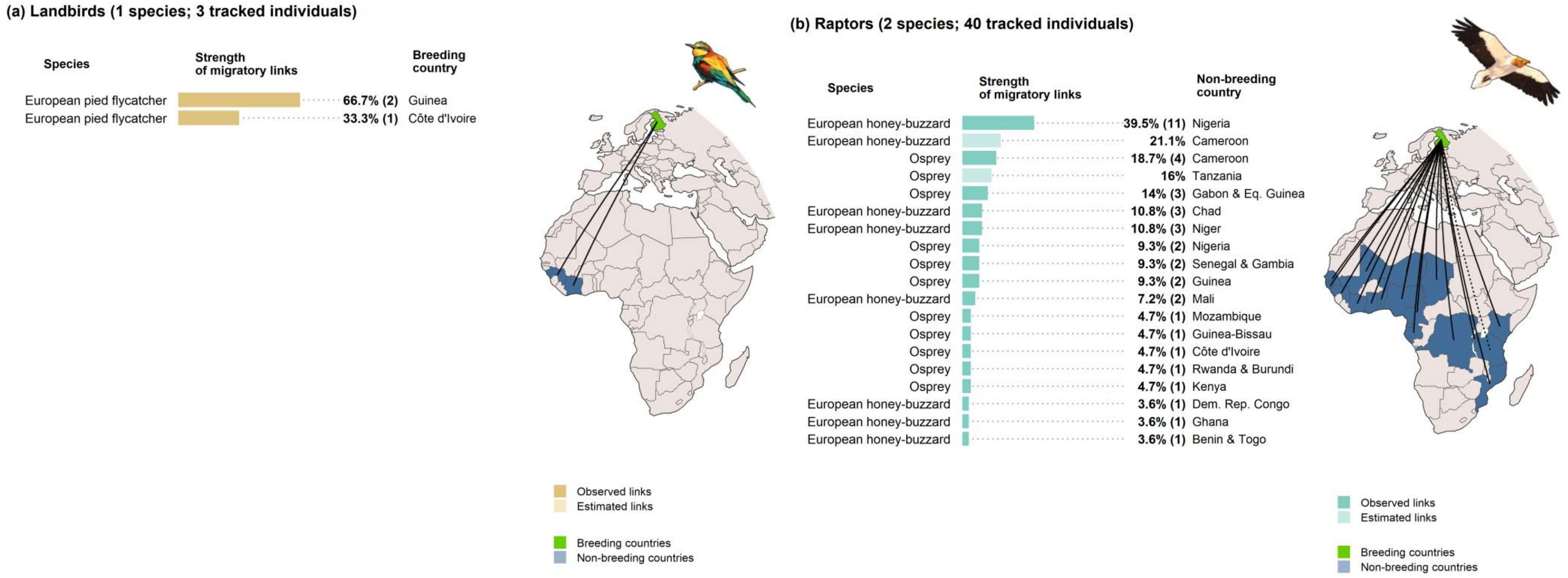

Figure S10.19: Estonia

Mapping country-level connectivity for Estonia, separately for (a) landbirds and (b) raptors. For each group, we present the list of all migratory links by decreasing order of strength (with respective number of tracked individuals in parenthesis), indicating in each case the species creating the link and the country it connects to. Maps represent how the migratory links (observed: solid lines; inferred: dotted lines) connect the countries in Europe (in green) to countries in sub-Saharan Africa (in blue).

(a) Landbirds (0 species tracked)

(b) Raptors (2 species; 14 tracked individuals)

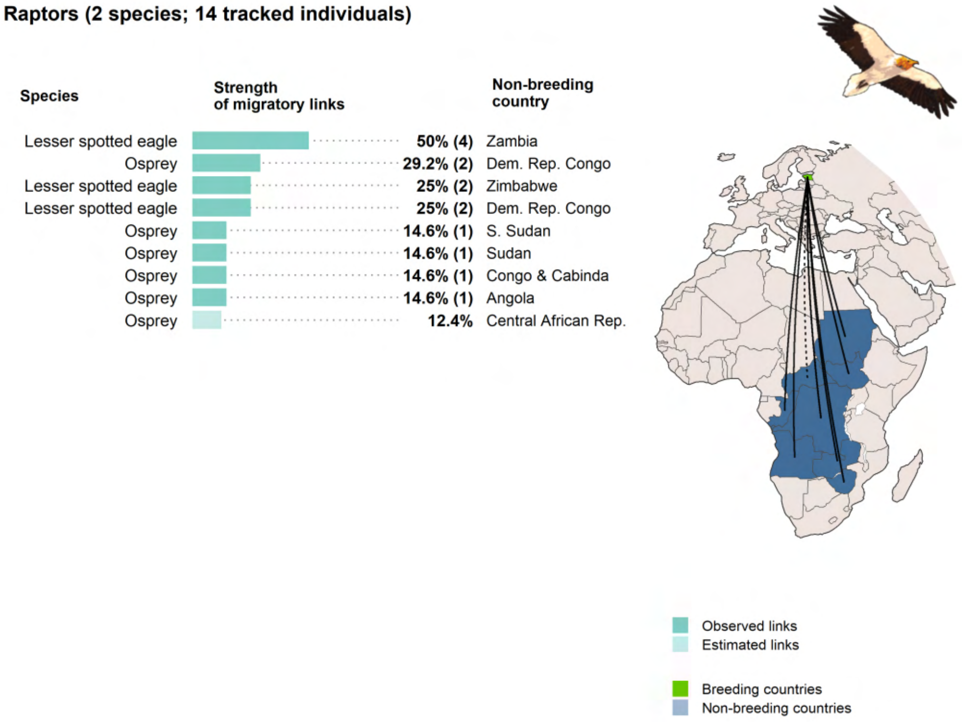

Figure S10.20: Latvia

Mapping country-level connectivity for Latvia, separately for (a) landbirds and (b) raptors. For each group, we present the list of all migratory links by decreasing order of strength (with respective number of tracked individuals in parenthesis), indicating in each case the species creating the link and the country it connects to. Maps represent how the migratory links (observed: solid lines; inferred: dotted lines) connect the countries in Europe (in green) to countries in sub-Saharan Africa (in blue).

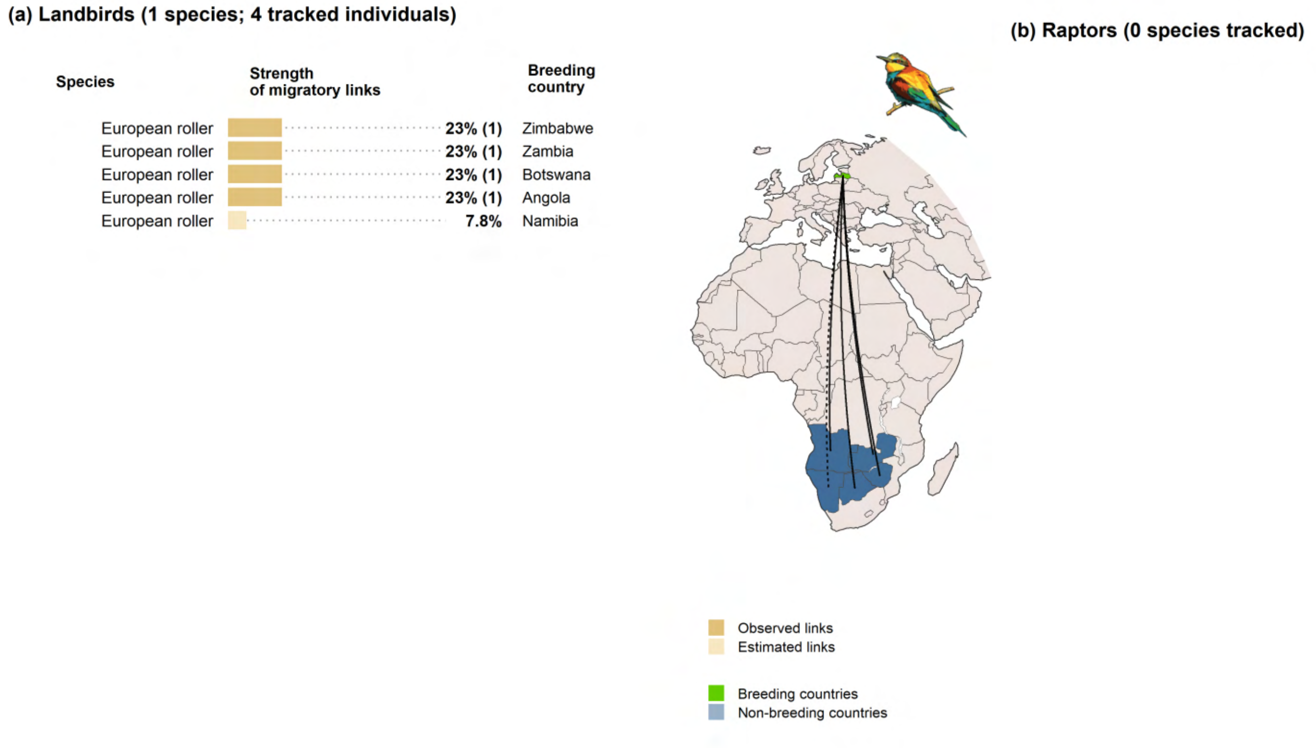

Figure S10.21: Lithuania

Mapping country-level connectivity for Lithuania, separately for (a) landbirds and (b) raptors. For each group, we present the list of all migratory links by decreasing order of strength (with respective number of tracked individuals in parenthesis), indicating in each case the species creating the link and the country it connects to. Maps represent how the migratory links (observed: solid lines; inferred: dotted lines) connect the countries in Europe (in green) to countries in sub-Saharan Africa (in blue).

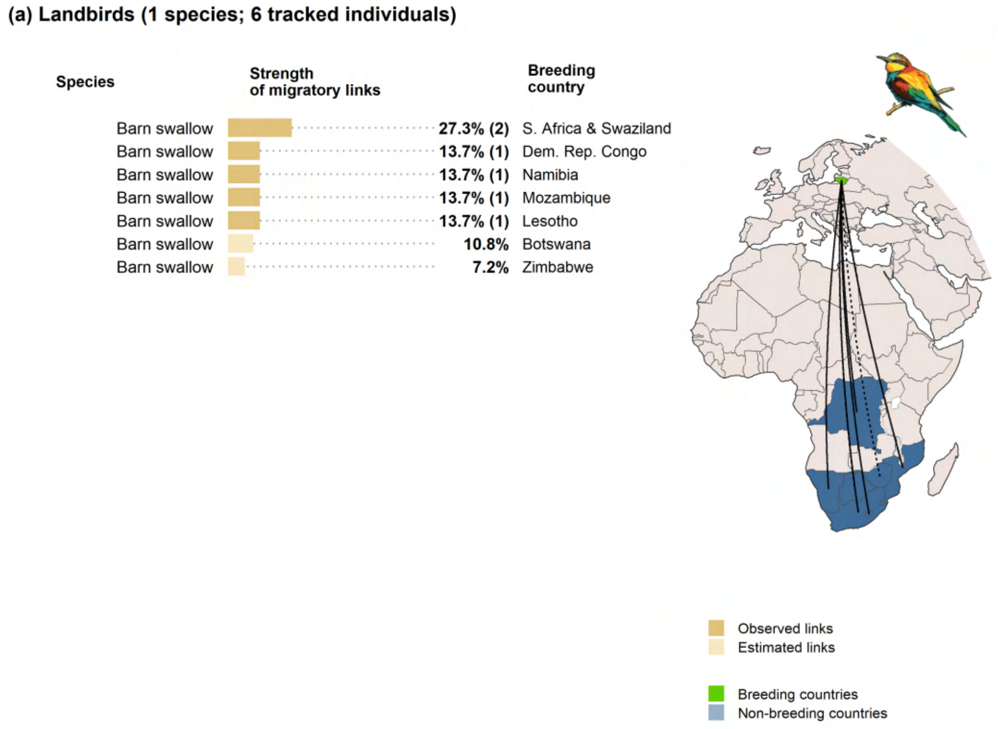

(b) Raptors (0 species tracked)

Figure S10.22: Belarus

Mapping country-level connectivity for Belarus, separately for (a) landbirds and (b) raptors. For each group, we present the list of all migratory links by decreasing order of strength (with respective number of tracked individuals in parenthesis), indicating in each case the species creating the link and the country it connects to. Maps represent how the migratory links (observed: solid lines; inferred: dotted lines) connect the countries in Europe (in green) to countries in sub-Saharan Africa (in blue).

(a) Landbirds (0 species tracked)

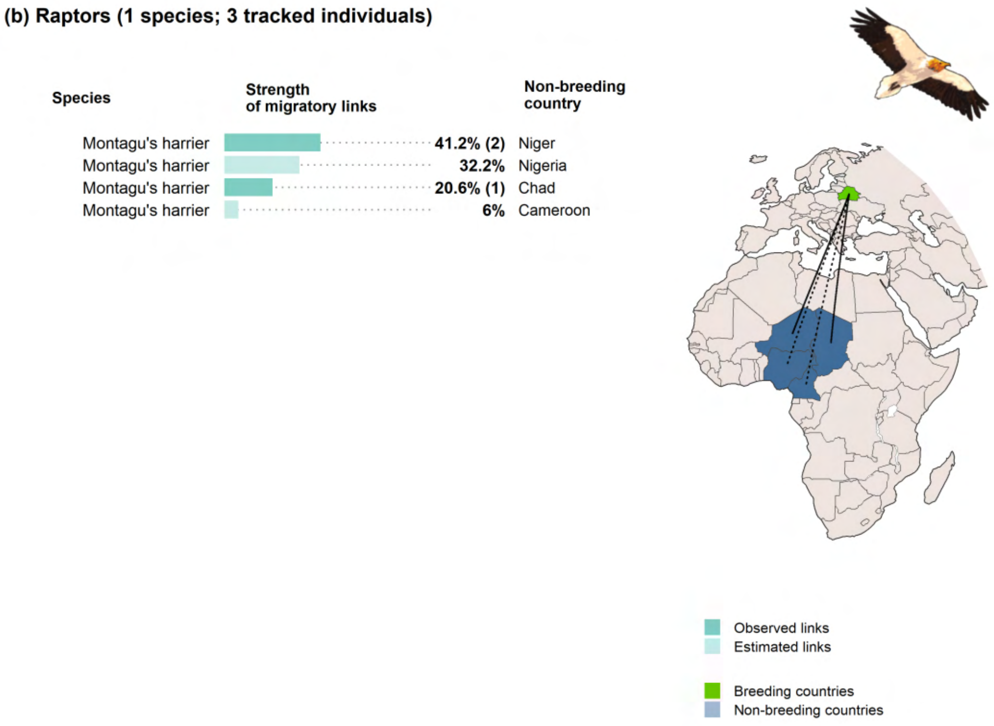

Figure S10.23: Ukraine

Mapping country-level connectivity for Ukraine, separately for (a) landbirds and (b) raptors. For each group, we present the list of all migratory links by decreasing order of strength (with respective number of tracked individuals in parenthesis), indicating in each case the species creating the link and the country it connects to. Maps represent how the migratory links (observed: solid lines; inferred: dotted lines) connect the countries in Europe (in green) to countries in sub-Saharan Africa (in blue).

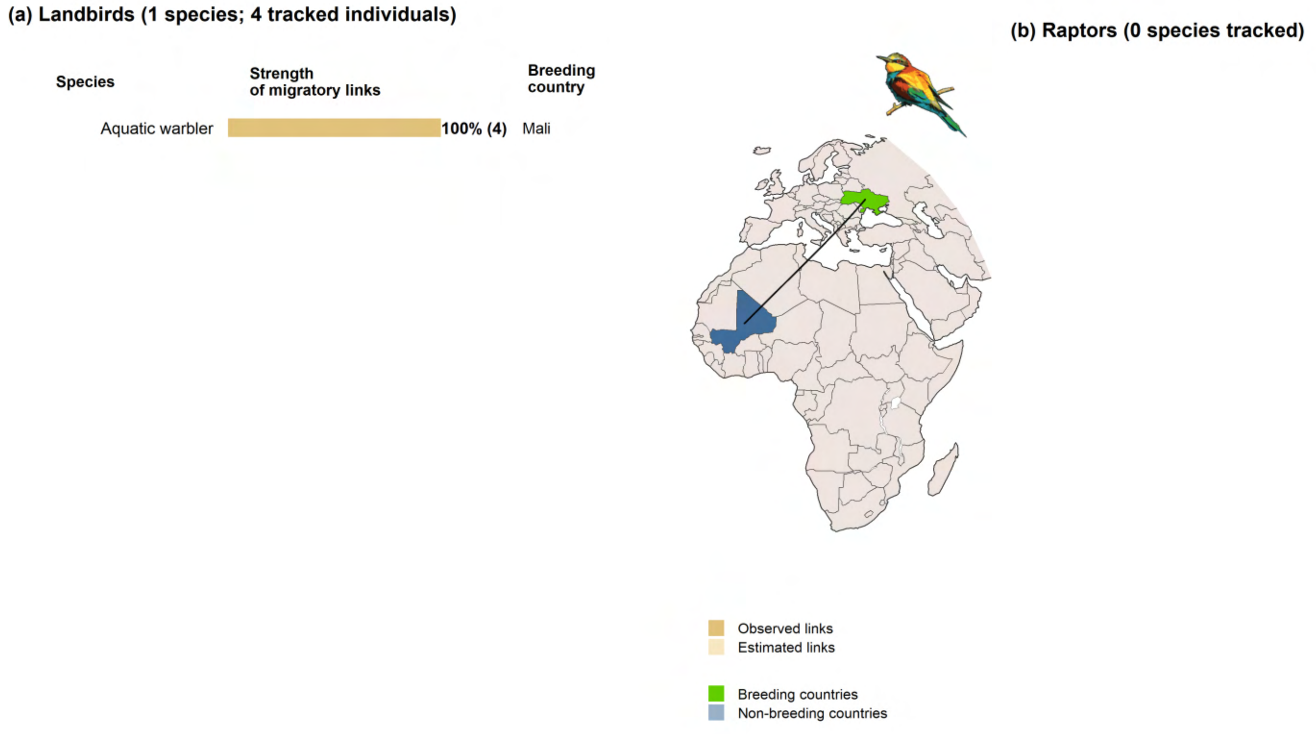

Figure S10.24: Romania

Mapping country-level connectivity for Romania, separately for (a) landbirds and (b) raptors. For each group, we present the list of all migratory links by decreasing order of strength (with respective number of tracked individuals in parenthesis), indicating in each case the species creating the link and the country it connects to. Maps represent how the migratory links (observed: solid lines; inferred: dotted lines) connect the countries in Europe (in green) to countries in sub-Saharan Africa (in blue).

(a) Landbirds (0 species tracked)

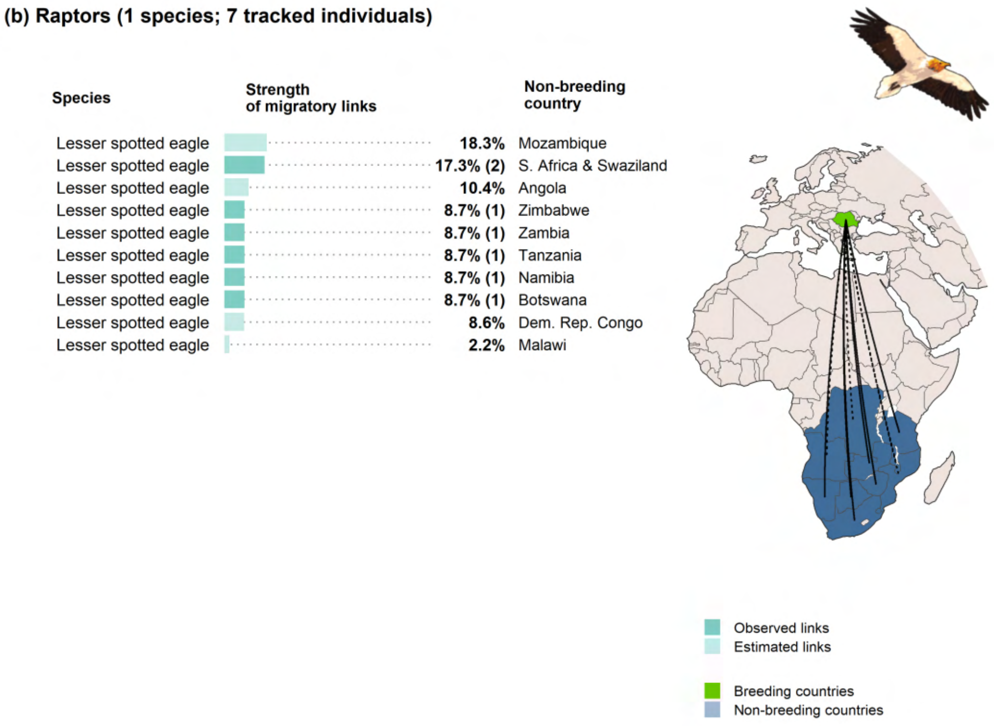

Figure S10.25: Bulgaria

Mapping country-level connectivity for Bulgaria, separately for (a) landbirds and (b) raptors. For each group, we present the list of all migratory links by decreasing order of strength (with respective number of tracked individuals in parenthesis), indicating in each case the species creating the link and the country it connects to. Maps represent how the migratory links (observed: solid lines; inferred: dotted lines) connect the countries in Europe (in green) to countries in sub-Saharan Africa (in blue).

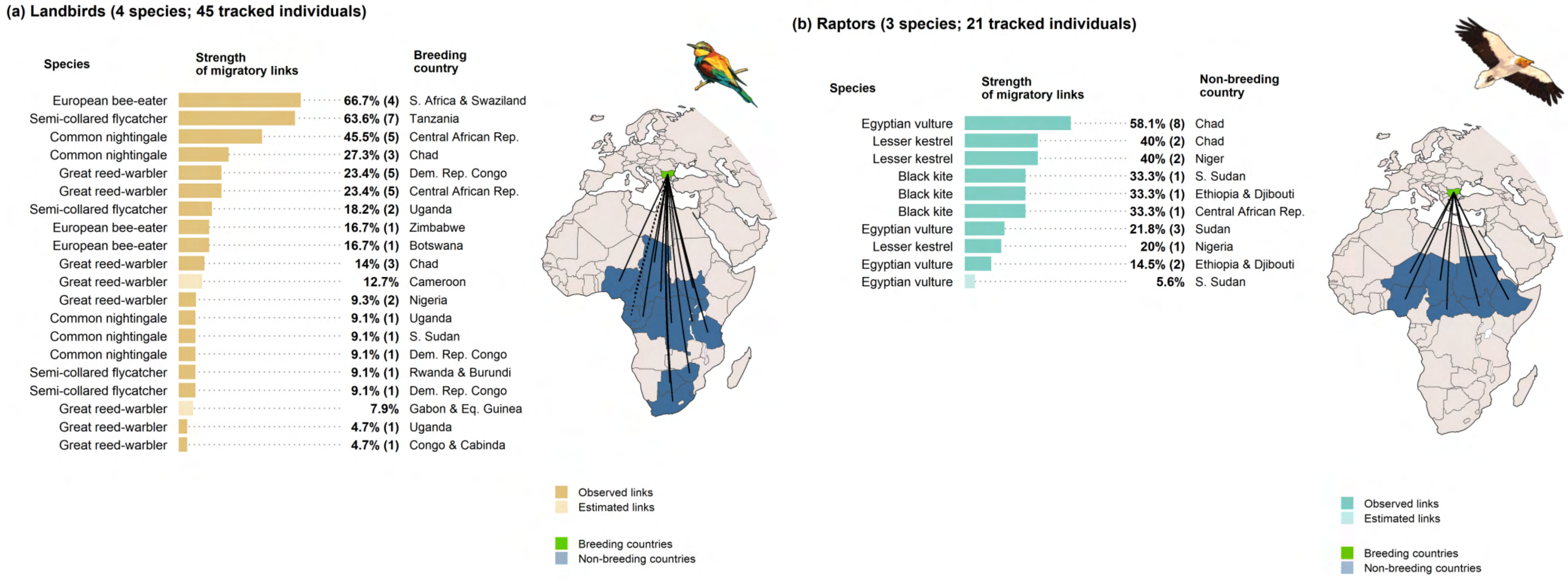

Figure S10.26: Greece

Mapping country-level connectivity for Greece, separately for (a) landbirds and (b) raptors. For each group, we present the list of all migratory links by decreasing order of strength (with respective number of tracked individuals in parenthesis), indicating in each case the species creating the link and the country it connects to. Maps represent how the migratory links (observed: solid lines; inferred: dotted lines) connect the countries in Europe (in green) to countries in sub-Saharan Africa (in blue).

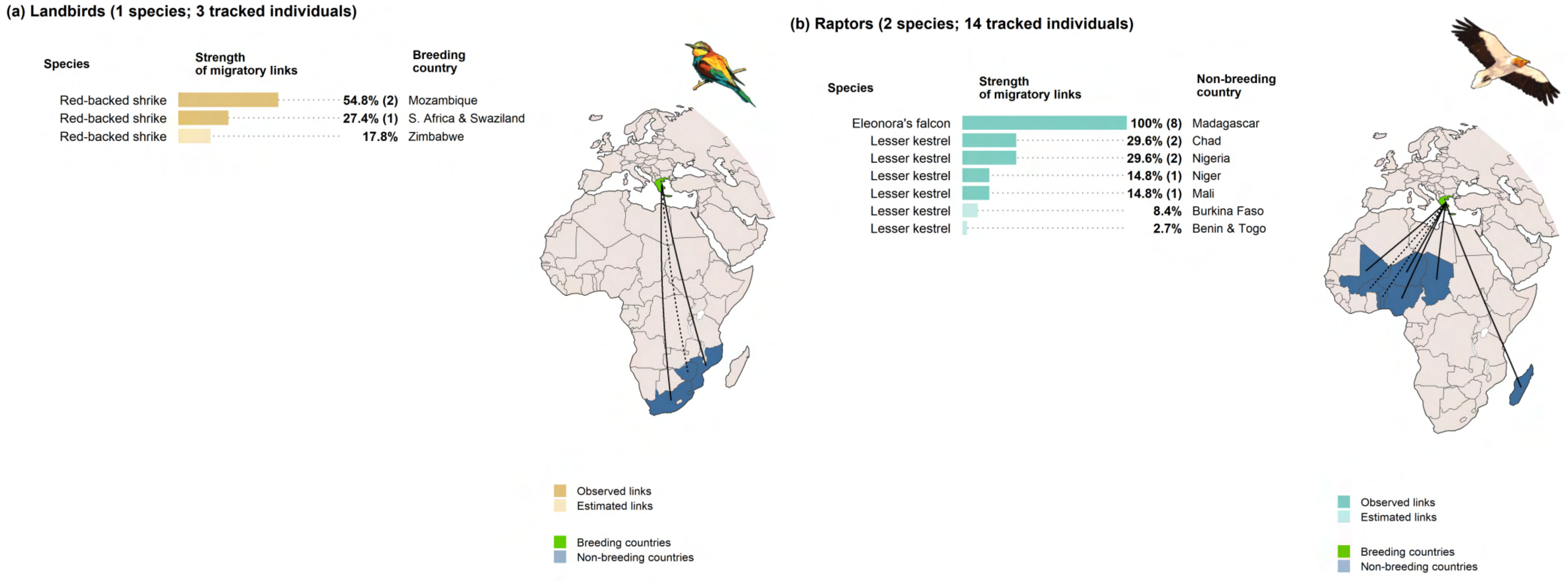

Figure S10.27: Cyprus

Mapping country-level connectivity for Cyprus, separately for (a) landbirds and (b) raptors. For each group, we present the list of all migratory links by decreasing order of strength (with respective number of tracked individuals in parenthesis), indicating in each case the species creating the link and the country it connects to. Maps represent how the migratory links (observed: solid lines; inferred: dotted lines) connect the countries in Europe (in green) to countries in sub-Saharan Africa (in blue).

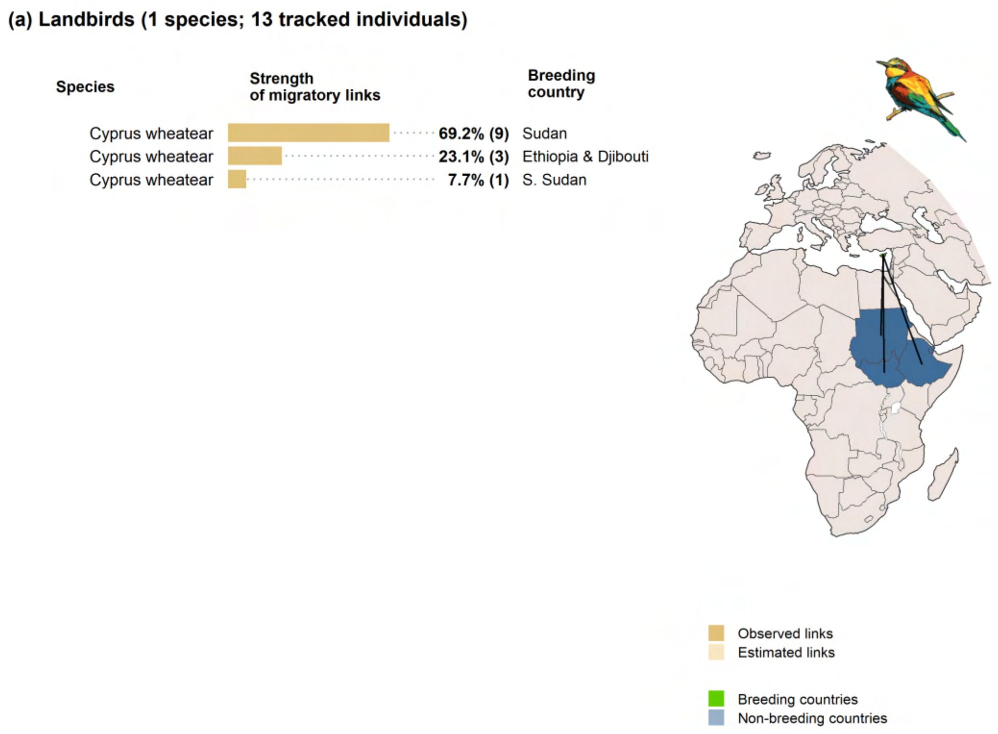

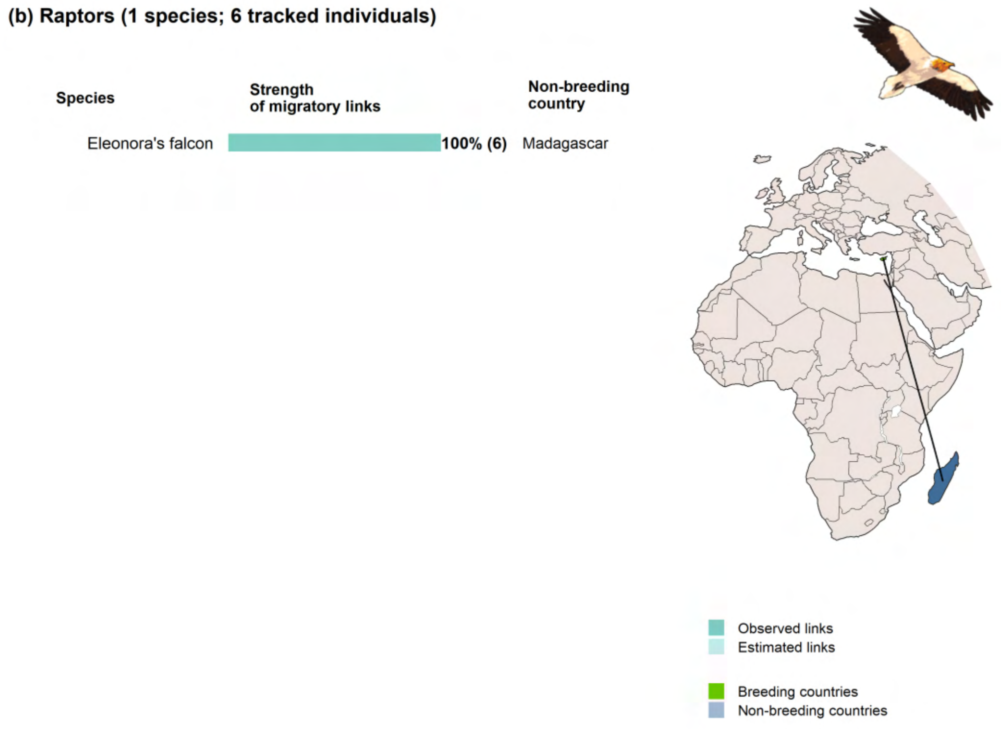

Figure S10.28: Turkey

Mapping country-level connectivity for Turkey, separately for (a) landbirds and (b) raptors. For each group, we present the list of all migratory links by decreasing order of strength (with respective number of tracked individuals in parenthesis), indicating in each case the species creating the link and the country it connects to. Maps represent how the migratory links (observed: solid lines; inferred: dotted lines) connect the countries in Europe (in green) to countries in sub-Saharan Africa (in blue).

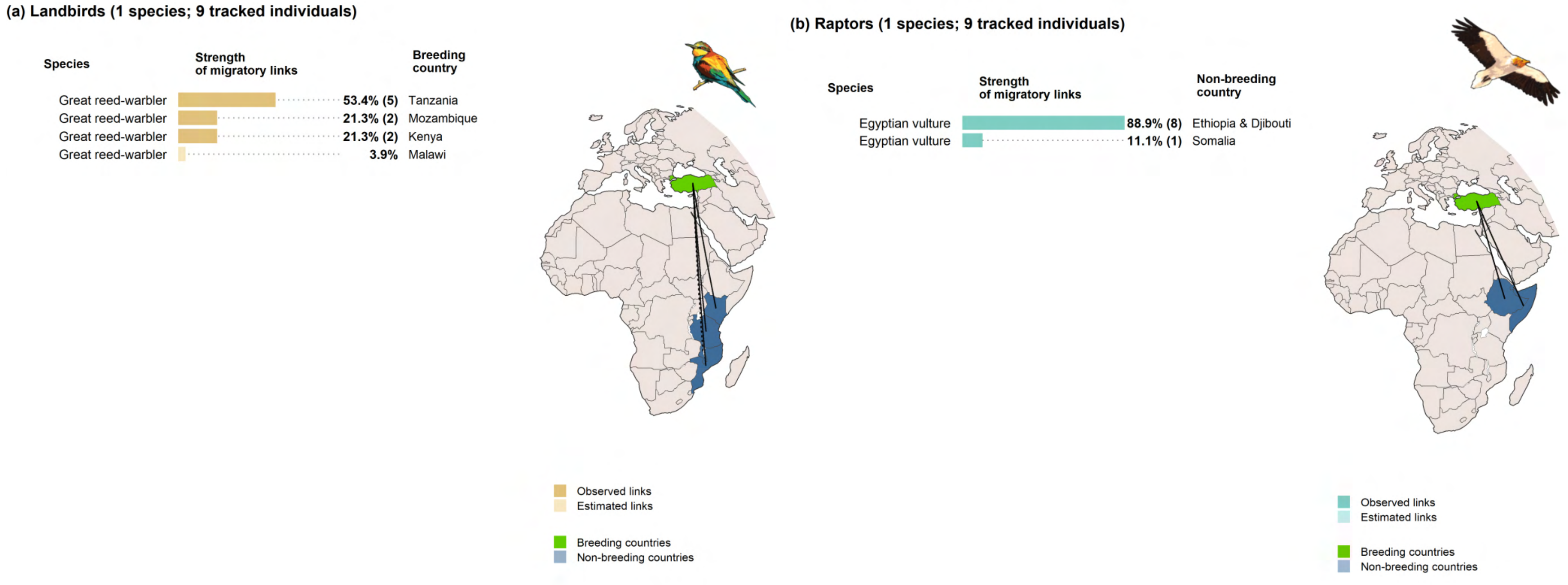

Figure S10.29: Mauritania

Mapping country-level connectivity for Mauritania, separately for (a) landbirds and (b) raptors. For each group, we present the list of all migratory links by decreasing order of strength (with respective number of tracked individuals in parenthesis), indicating in each case the species creating the link and the country it connects to. Maps represent how the migratory links (observed: solid lines; inferred: dotted lines) connect the countries in Europe (in green) to countries in sub-Saharan Africa (in blue).

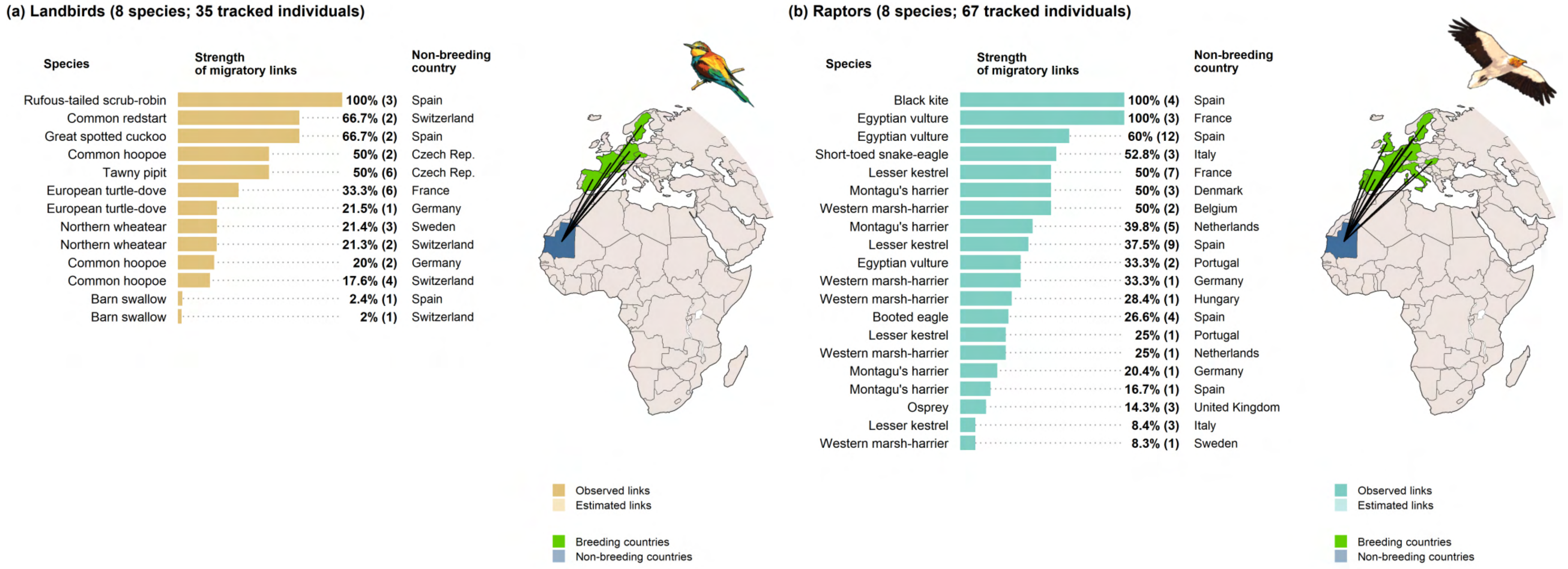

Figure S10.30: Senegal & Gambia

Mapping country-level connectivity for Senegal & Gambia, separately for (a) landbirds and (b) raptors. For each group, we present the list of all migratory links by decreasing order of strength (with respective number of tracked individuals in parenthesis), indicating in each case the species creating the link and the country it connects to. Maps represent how the migratory links (observed: solid lines; inferred: dotted lines) connect the countries in Europe (in green) to countries in sub-Saharan Africa (in blue).

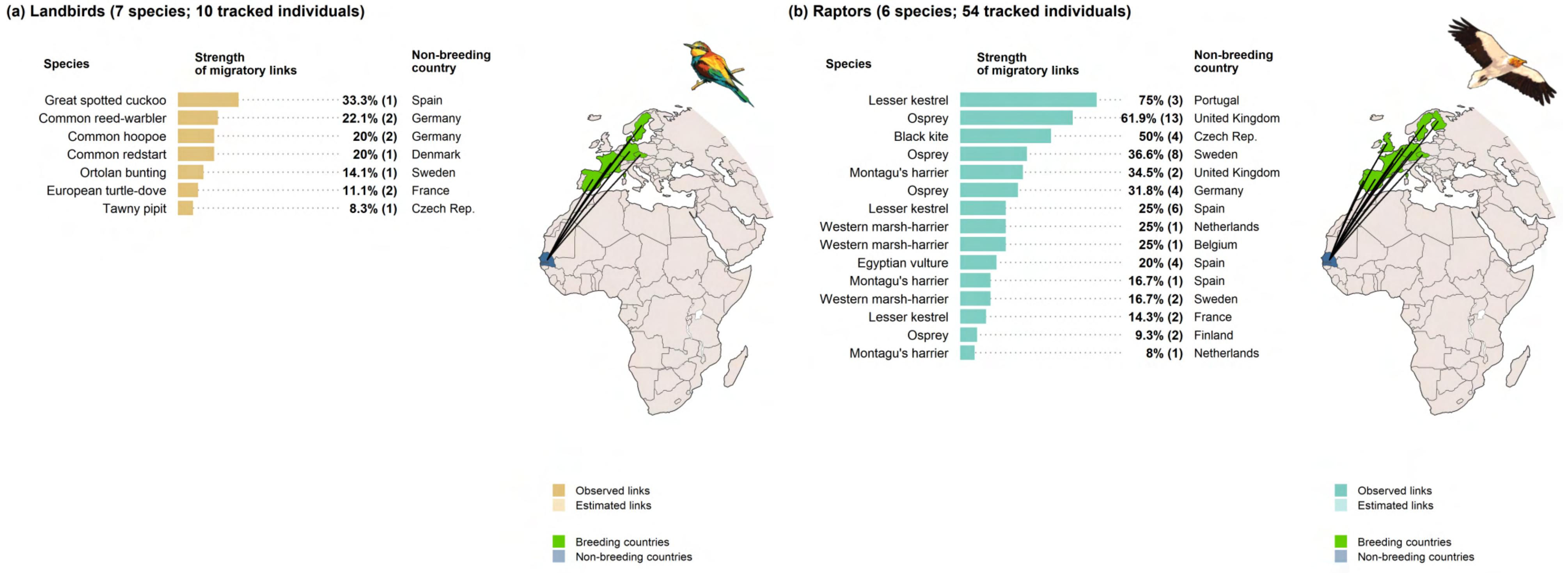

Figure S10.31: Guinea-Bissau

Mapping country-level connectivity for Guinea-Bissau, separately for (a) landbirds and (b) raptors. For each group, we present the list of all migratory links by decreasing order of strength (with respective number of tracked individuals in parenthesis), indicating in each case the species creating the link and the country it connects to. Maps represent how the migratory links (observed: solid lines; inferred: dotted lines) connect the countries in Europe (in green) to countries in sub-Saharan Africa (in blue).

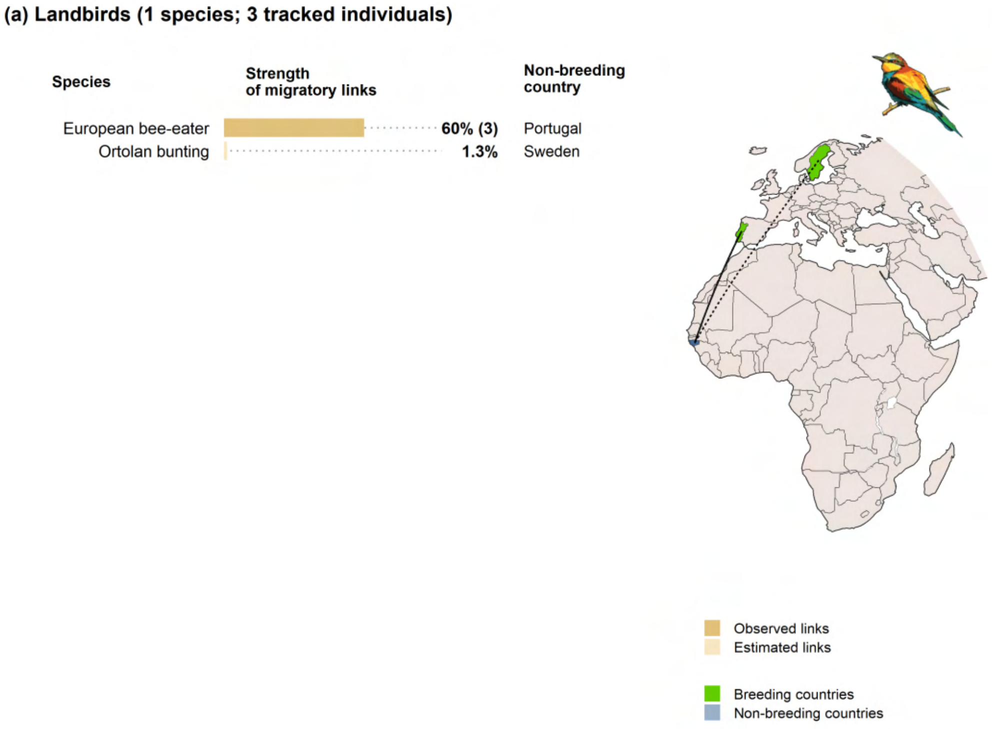

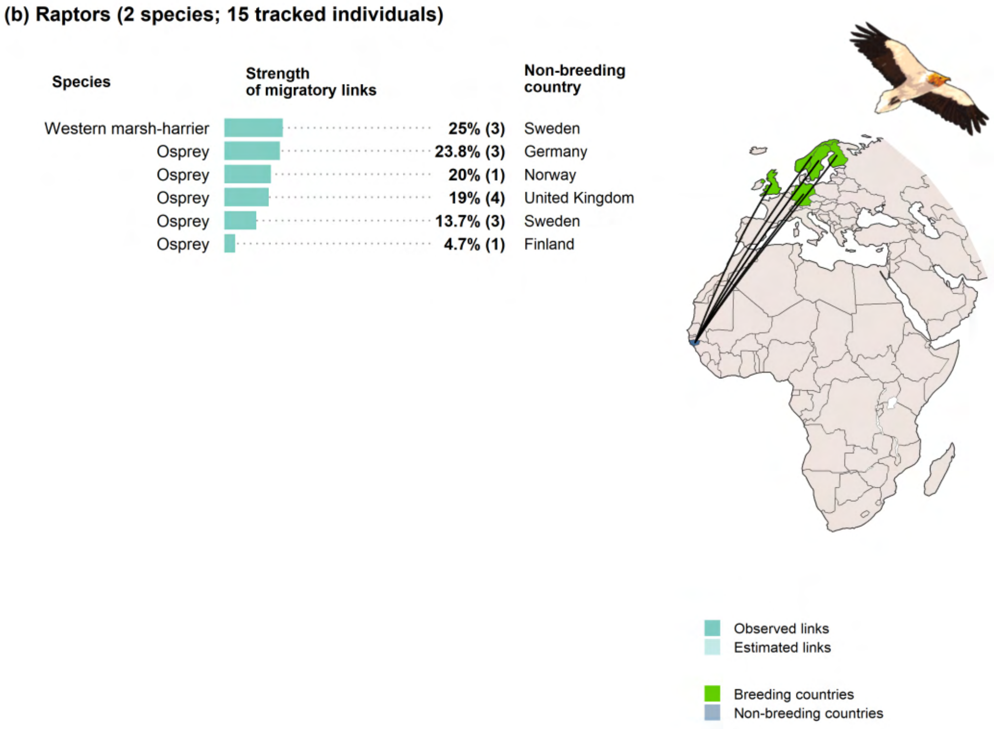

Figure S10.32: Guinea

Mapping country-level connectivity for Guinea, separately for (a) landbirds and (b) raptors. For each group, we present the list of all migratory links by decreasing order of strength (with respective number of tracked individuals in parenthesis), indicating in each case the species creating the link and the country it connects to. Maps represent how the migratory links (observed: solid lines; inferred: dotted lines) connect the countries in Europe (in green) to countries in sub-Saharan Africa (in blue).

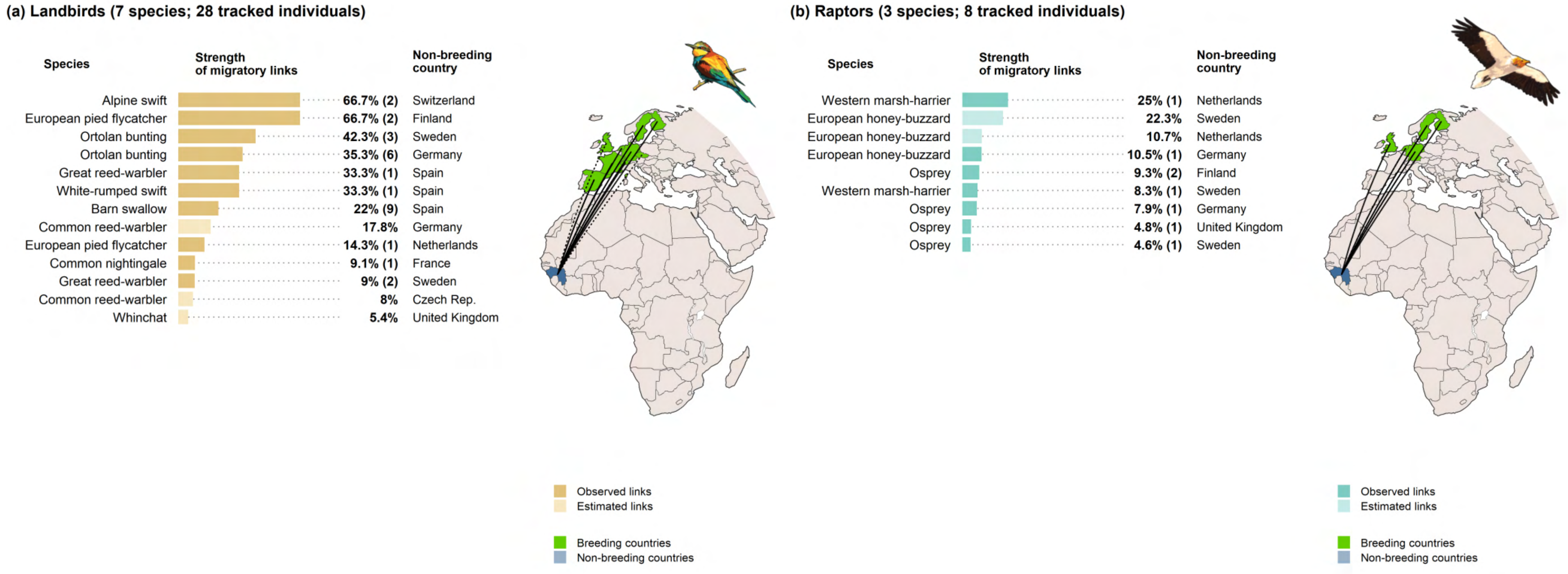

Figure S10.33: Sierra Leone

Mapping country-level connectivity for Sierra Leone, separately for (a) landbirds and (b) raptors. For each group, we present the list of all migratory links by decreasing order of strength (with respective number of tracked individuals in parenthesis), indicating in each case the species creating the link and the country it connects to. Maps represent how the migratory links (observed: solid lines; inferred: dotted lines) connect the countries in Europe (in green) to countries in sub-Saharan Africa (in blue).

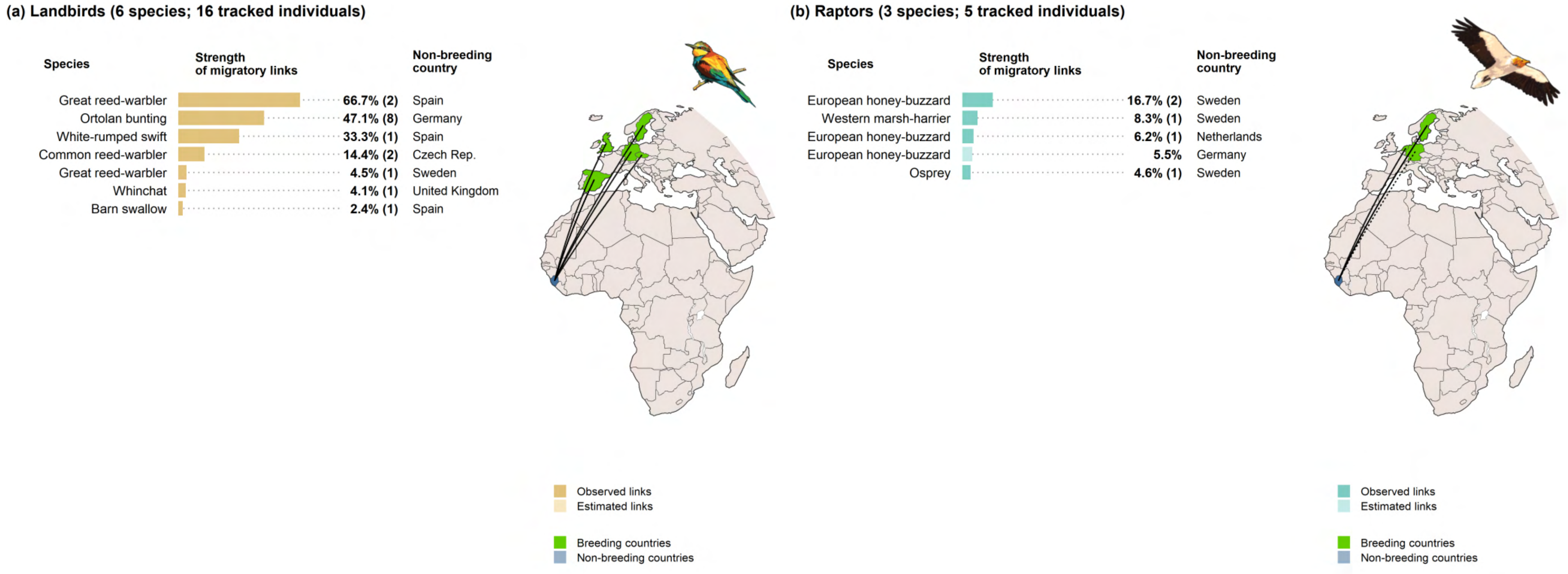

Figure S10.34: Liberia

Mapping country-level connectivity for Liberia, separately for (a) landbirds and (b) raptors. For each group, we present the list of all migratory links by decreasing order of strength (with respective number of tracked individuals in parenthesis), indicating in each case the species creating the link and the country it connects to. Maps represent how the migratory links (observed: solid lines; inferred: dotted lines) connect the countries in Europe (in green) to countries in sub-Saharan Africa (in blue).

(a) Landbirds (5 species; 10 tracked individuals)

Species	Strength of migratory links	Non-breeding country
White-rumped swift	33.3% (1)	Spain
Common nightingale	18.2% (2)	France
Ortolan bunting	17.6% (3)	Germany
Barn swallow	7.3% (3)	Spain
Common reed-warbler	7.2% (1)	Czech Rep.
Great reed-warbler	4.8%	Sweden
Whinchat	1.3%	United Kingdom

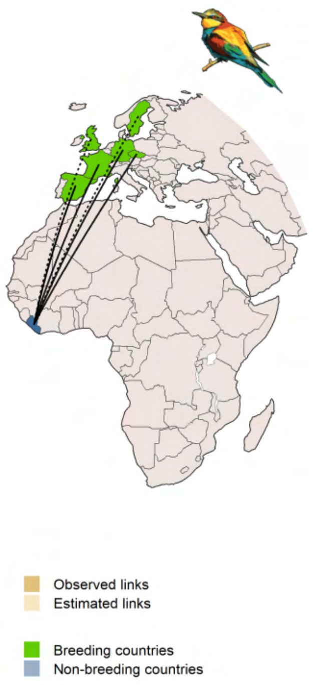

(b) Raptors (1 species; 7 tracked individuals)

Species	Strength of migratory links	Non-breeding country
European honey-buzzard	30.9% (5)	Netherlands
European honey-buzzard	14.1% (1)	United Kingdom
European honey-buzzard	10.8%	Sweden
European honey-buzzard	10.5% (1)	Germany
Osprey	3.6%	Sweden

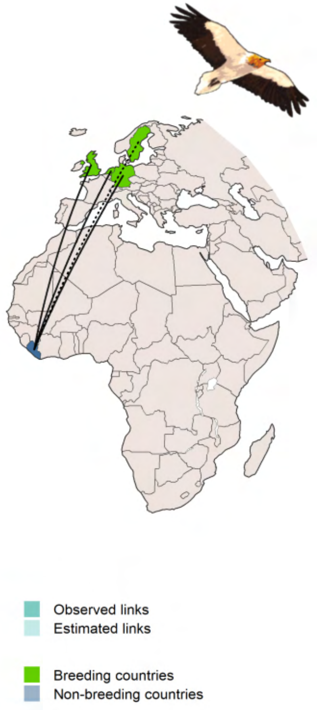

Figure S10.35: Mali

Mapping country-level connectivity for Mali, separately for (a) landbirds and (b) raptors. For each group, we present the list of all migratory links by decreasing order of strength (with respective number of tracked individuals in parenthesis), indicating in each case the species creating the link and the country it connects to. Maps represent how the migratory links (observed: solid lines; inferred: dotted lines) connect the countries in Europe (in green) to countries in sub-Saharan Africa (in blue).

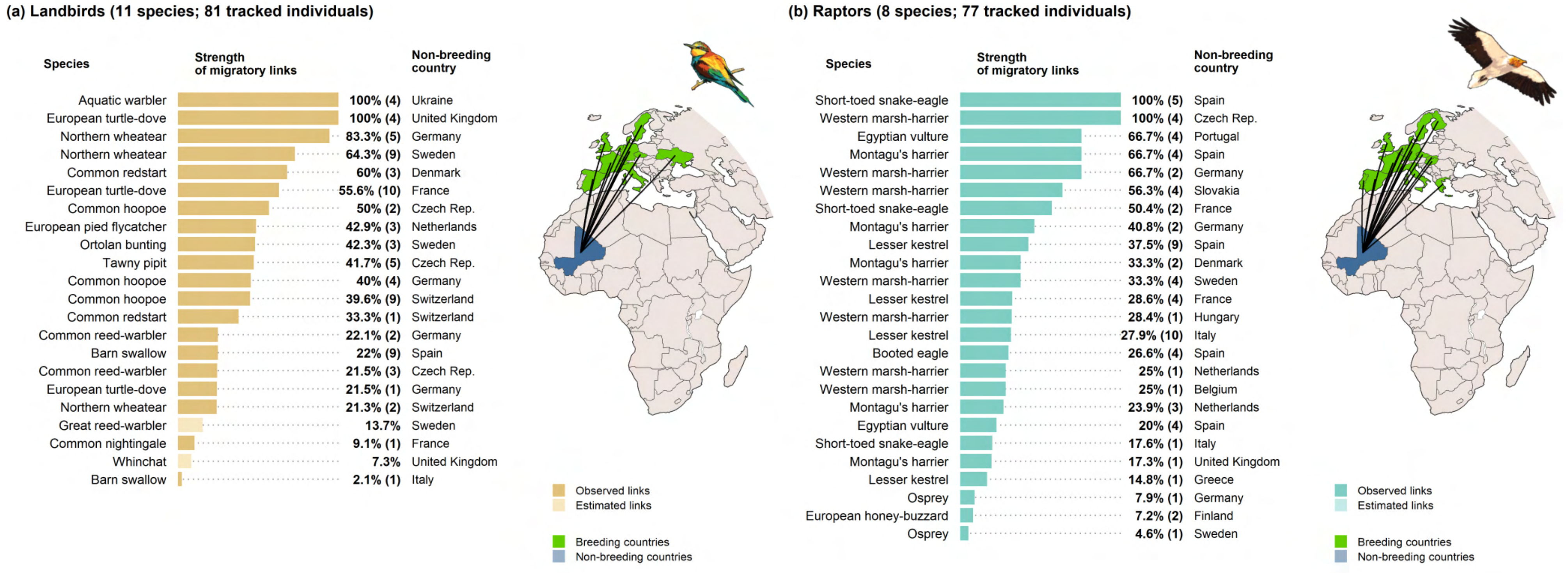

Figure S10.36: Côte d'Ivoire

Mapping country-level connectivity for Côte d'Ivoire, separately for (a) landbirds and (b) raptors. For each group, we present the list of all migratory links by decreasing order of strength (with respective number of tracked individuals in parenthesis), indicating in each case the species creating the link and the country it connects to. Maps represent how the migratory links (observed: solid lines; inferred: dotted lines) connect the countries in Europe (in green) to countries in sub-Saharan Africa (in blue).

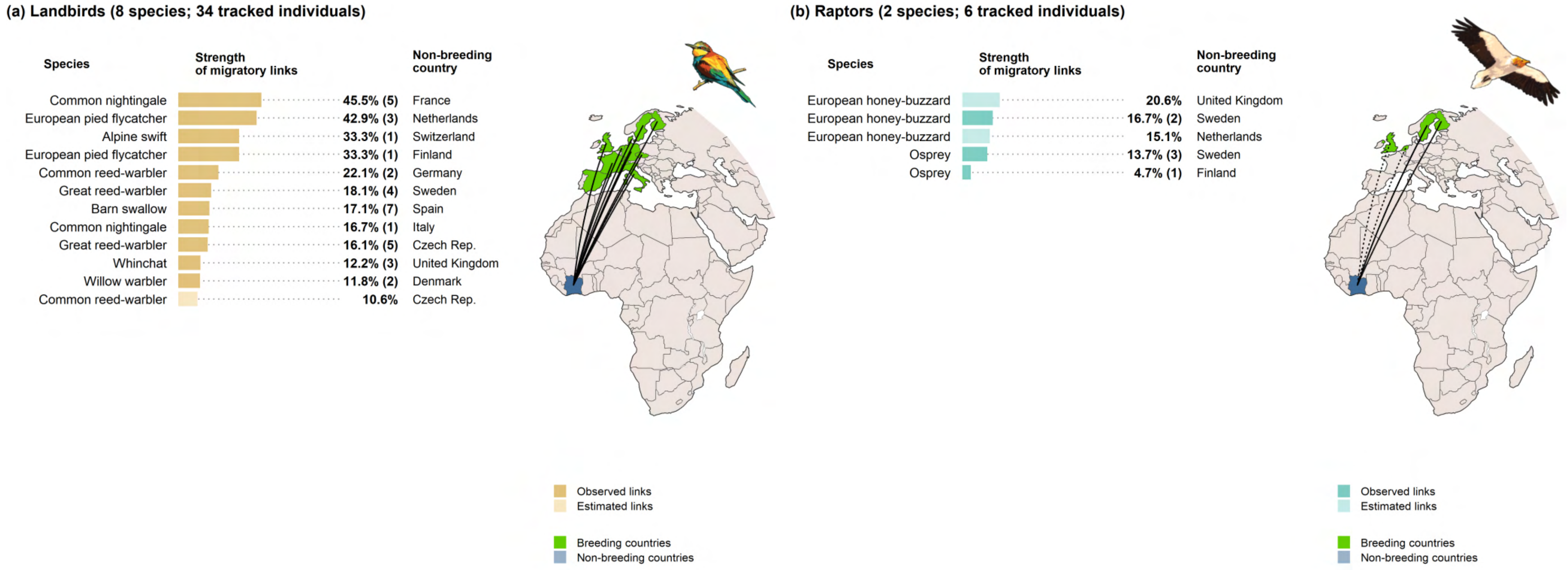

Figure S10.37: Burkina Faso

Mapping country-level connectivity for Burkina Faso, separately for (a) landbirds and (b) raptors. For each group, we present the list of all migratory links by decreasing order of strength (with respective number of tracked individuals in parenthesis), indicating in each case the species creating the link and the country it connects to. Maps represent how the migratory links (observed: solid lines; inferred: dotted lines) connect the countries in Europe (in green) to countries in sub-Saharan Africa (in blue).

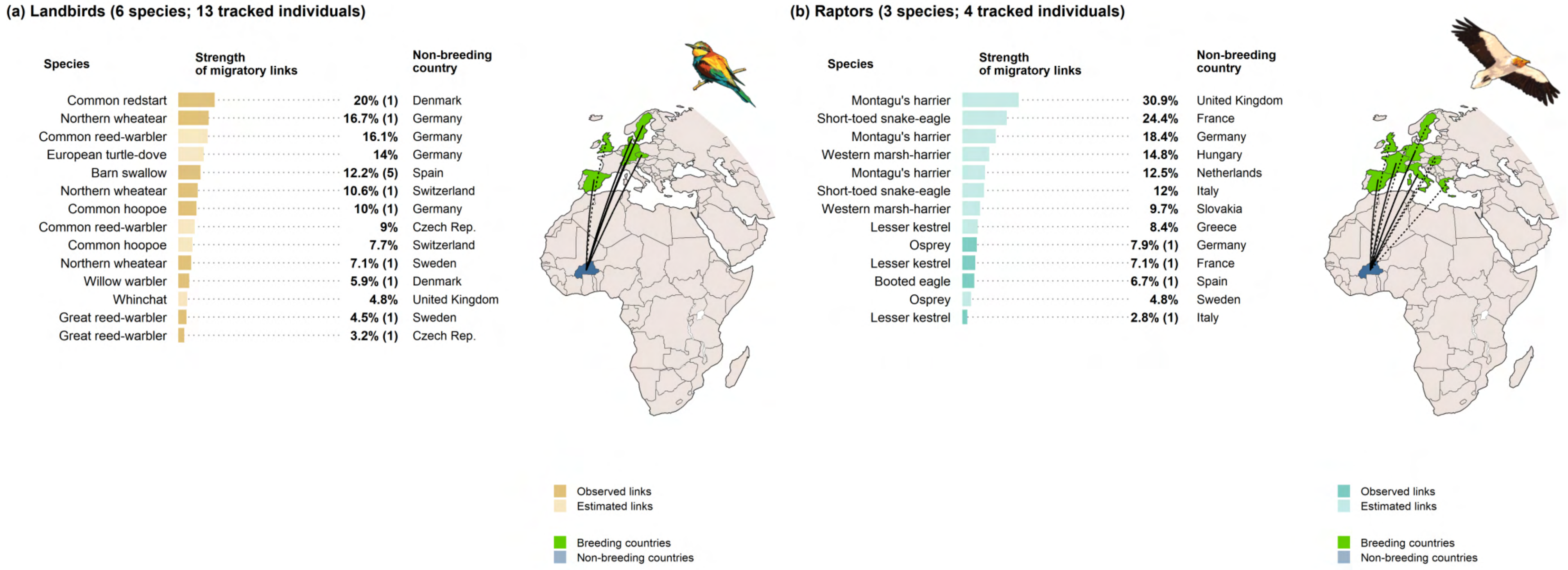

Figure S10.38: Ghana

Mapping country-level connectivity for Ghana, separately for (a) landbirds and (b) raptors. For each group, we present the list of all migratory links by decreasing order of strength (with respective number of tracked individuals in parenthesis), indicating in each case the species creating the link and the country it connects to. Maps represent how the migratory links (observed: solid lines; inferred: dotted lines) connect the countries in Europe (in green) to countries in sub-Saharan Africa (in blue).

(a) Landbirds (6 species; 24 tracked individuals)

Species	Strength of migratory links	Non-breeding country
Whinchat	36.5% (9)	United Kingdom
Common nightingale	33.3% (2)	Italy
Common nightingale	18.2% (2)	France
Willow warbler	11.8% (2)	Denmark
European bee-eater	11.5% (2)	Germany
Great reed-warbler	9.6% (3)	Czech Rep.
Common reed-warbler	7.8%	Czech Rep.
Barn swallow	7.3% (3)	Spain
Great reed-warbler	4.5% (1)	Sweden

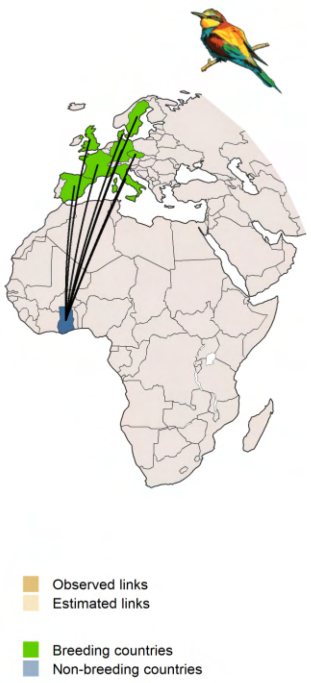

(b) Raptors (4 species; 9 tracked individuals)

Species	Strength of migratory links	Non-breeding country
Black kite	25% (2)	Czech Rep.
European honey-buzzard	18.5% (3)	Netherlands
Montagu's harrier	17.3% (1)	United Kingdom
European honey-buzzard	13.5%	United Kingdom
European honey-buzzard	8.4% (1)	Sweden
Osprey	4.6% (1)	Sweden
European honey-buzzard	3.6% (1)	Finland

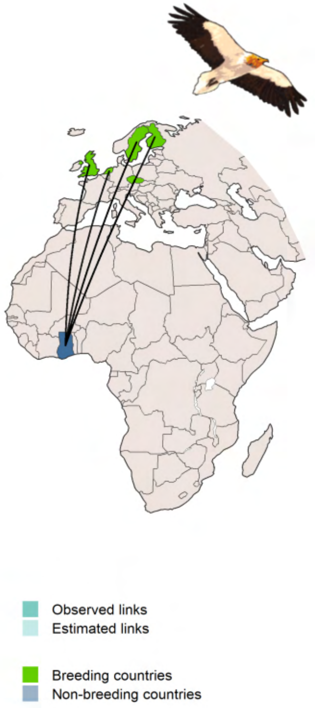

Figure S10.39: Benin & Togo

Mapping country-level connectivity for Benin & Togo, separately for (a) landbirds and (b) raptors. For each group, we present the list of all migratory links by decreasing order of strength (with respective number of tracked individuals in parenthesis), indicating in each case the species creating the link and the country it connects to. Maps represent how the migratory links (observed: solid lines; inferred: dotted lines) connect the countries in Europe (in green) to countries in sub-Saharan Africa (in blue).

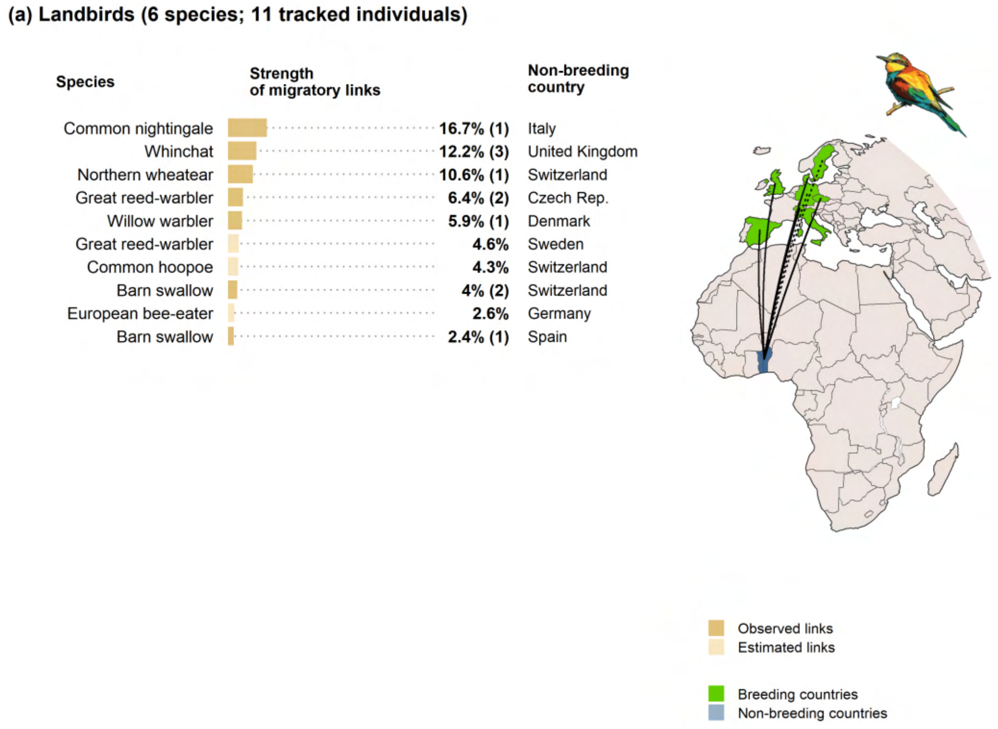

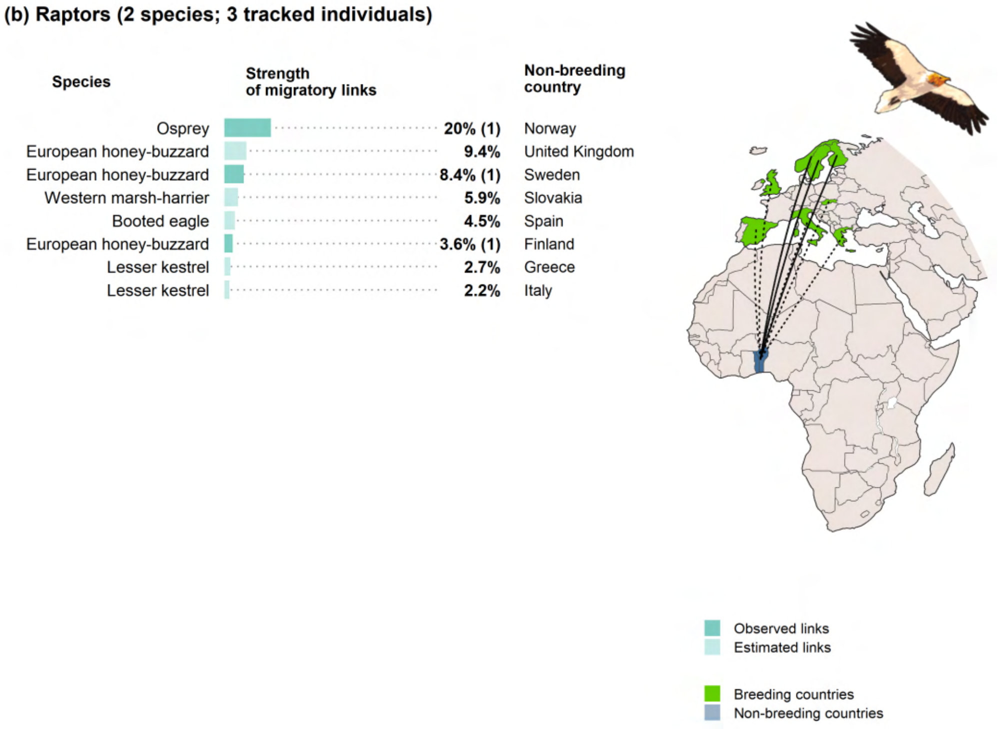

Figure S10.40: Nigeria

Mapping country-level connectivity for Nigeria, separately for (a) landbirds and (b) raptors. For each group, we present the list of all migratory links by decreasing order of strength (with respective number of tracked individuals in parenthesis), indicating in each case the species creating the link and the country it connects to. Maps represent how the migratory links (observed: solid lines; inferred: dotted lines) connect the countries in Europe (in green) to countries in sub-Saharan Africa (in blue).

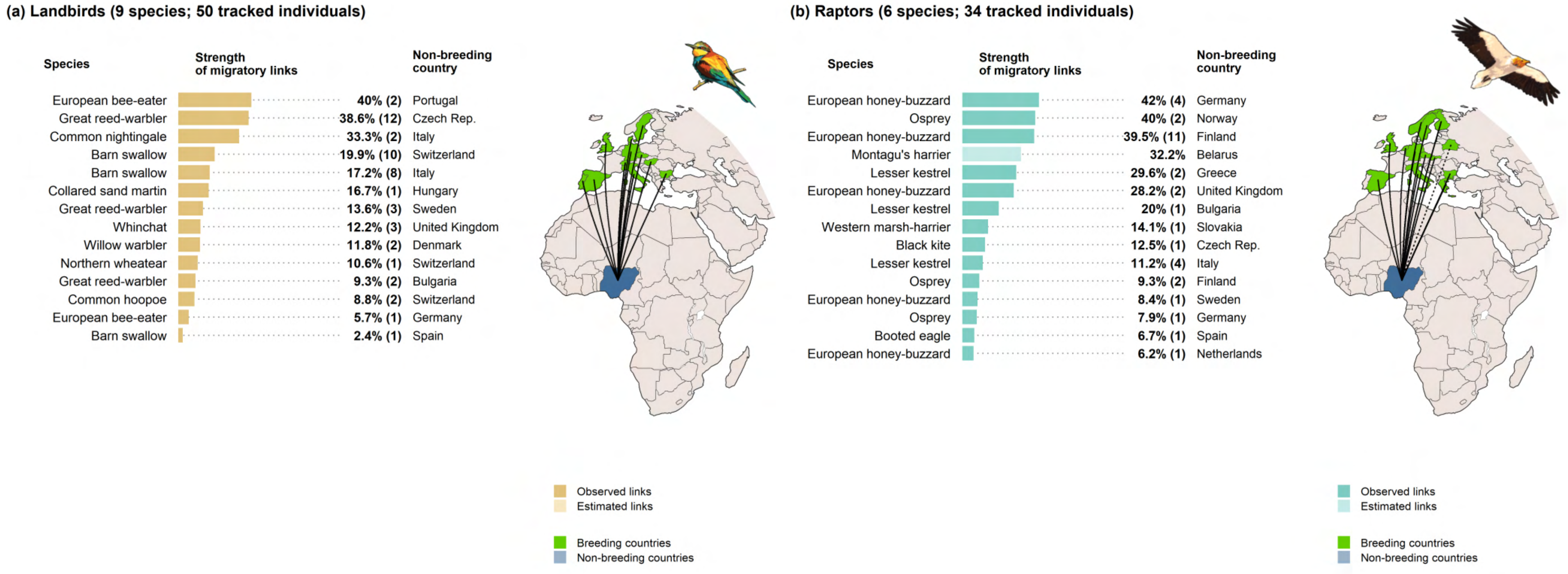

Figure S10.41: Niger

Mapping country-level connectivity for Niger, separately for (a) landbirds and (b) raptors. For each group, we present the list of all migratory links by decreasing order of strength (with respective number of tracked individuals in parenthesis), indicating in each case the species creating the link and the country it connects to. Maps represent how the migratory links (observed: solid lines; inferred: dotted lines) connect the countries in Europe (in green) to countries in sub-Saharan Africa (in blue).

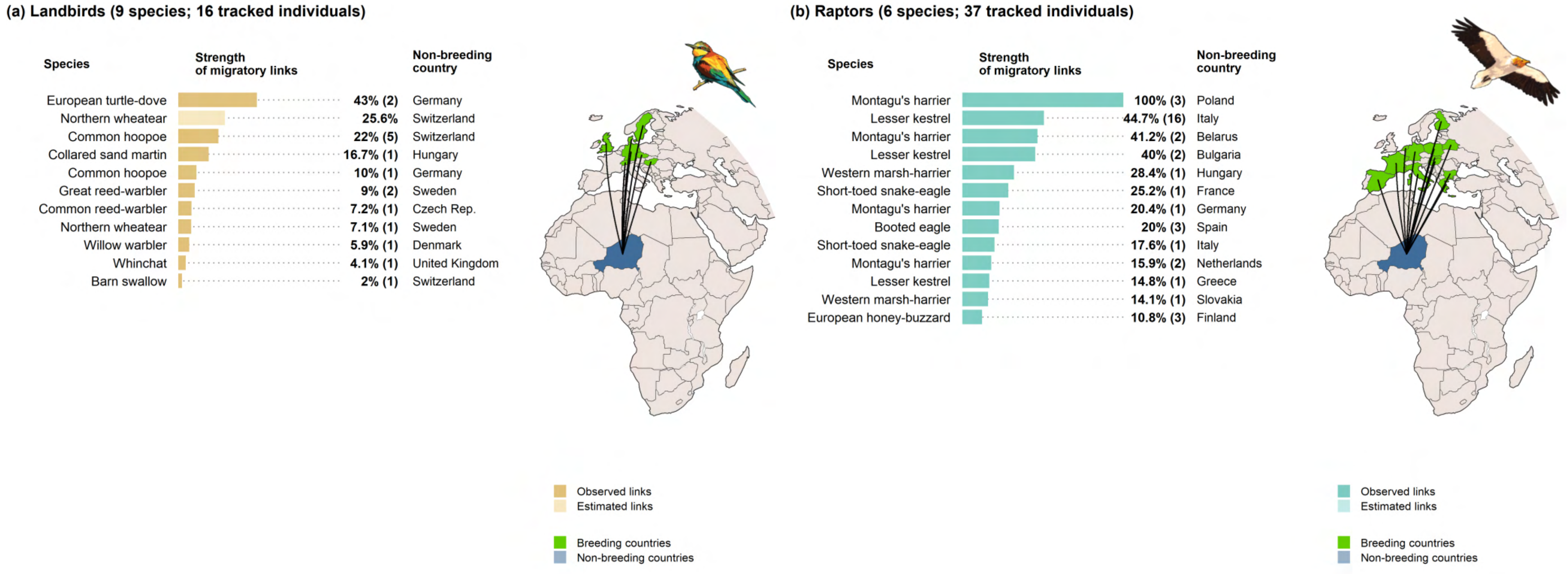

Figure S10.42: Cameroon

Mapping country-level connectivity for Cameroon, separately for (a) landbirds and (b) raptors. For each group, we present the list of all migratory links by decreasing order of strength (with respective number of tracked individuals in parenthesis), indicating in each case the species creating the link and the country it connects to. Maps represent how the migratory links (observed: solid lines; inferred: dotted lines) connect the countries in Europe (in green) to countries in sub-Saharan Africa (in blue).

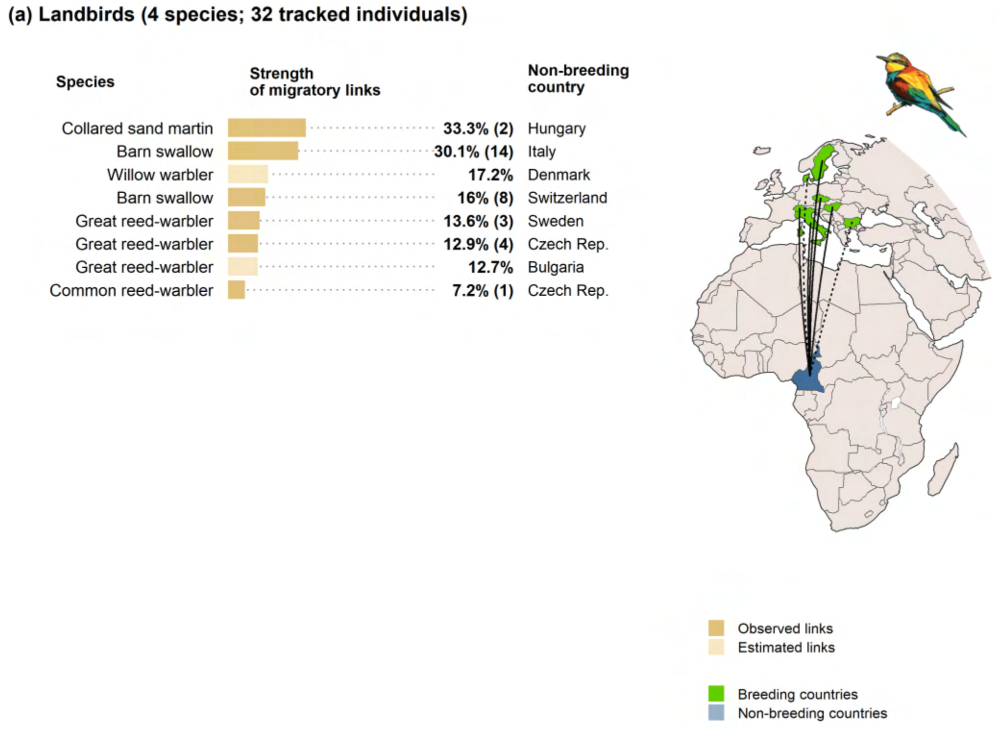

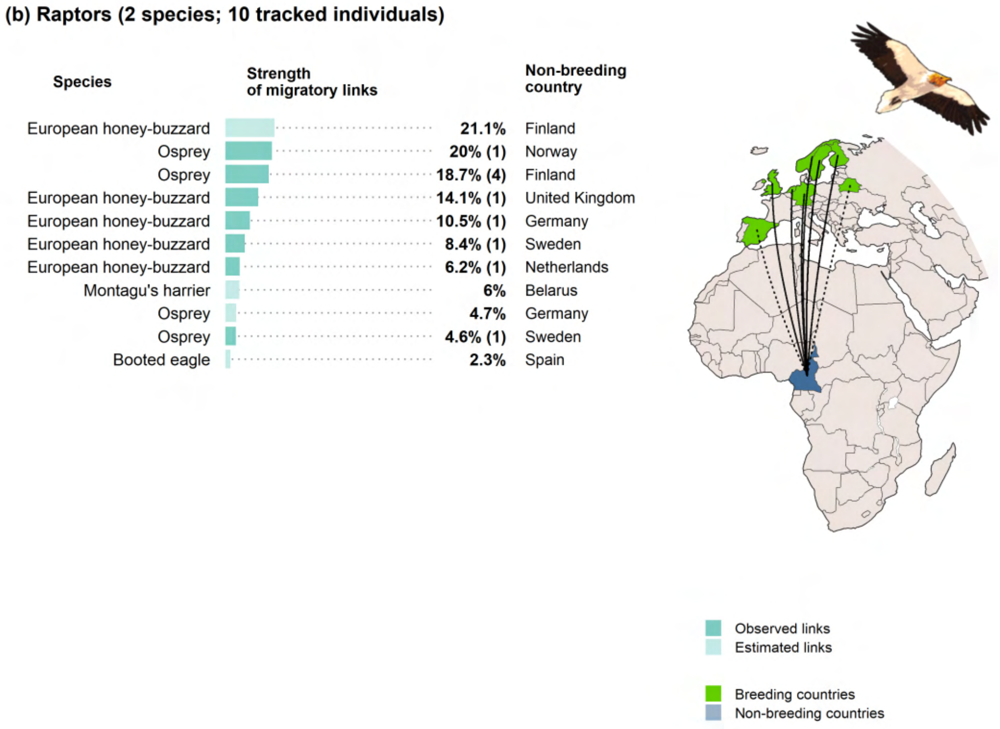

Figure S10.43: Chad

Mapping country-level connectivity for Chad, separately for (a) landbirds and (b) raptors. For each group, we present the list of all migratory links by decreasing order of strength (with respective number of tracked individuals in parenthesis), indicating in each case the species creating the link and the country it connects to. Maps represent how the migratory links (observed: solid lines; inferred: dotted lines) connect the countries in Europe (in green) to countries in sub-Saharan Africa (in blue).

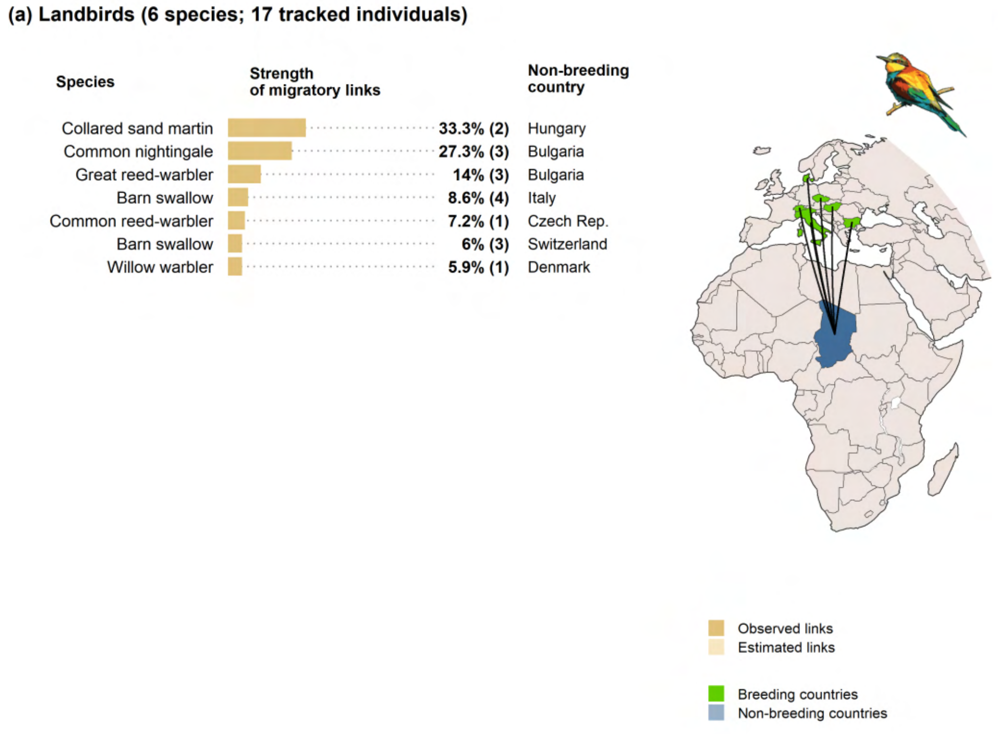

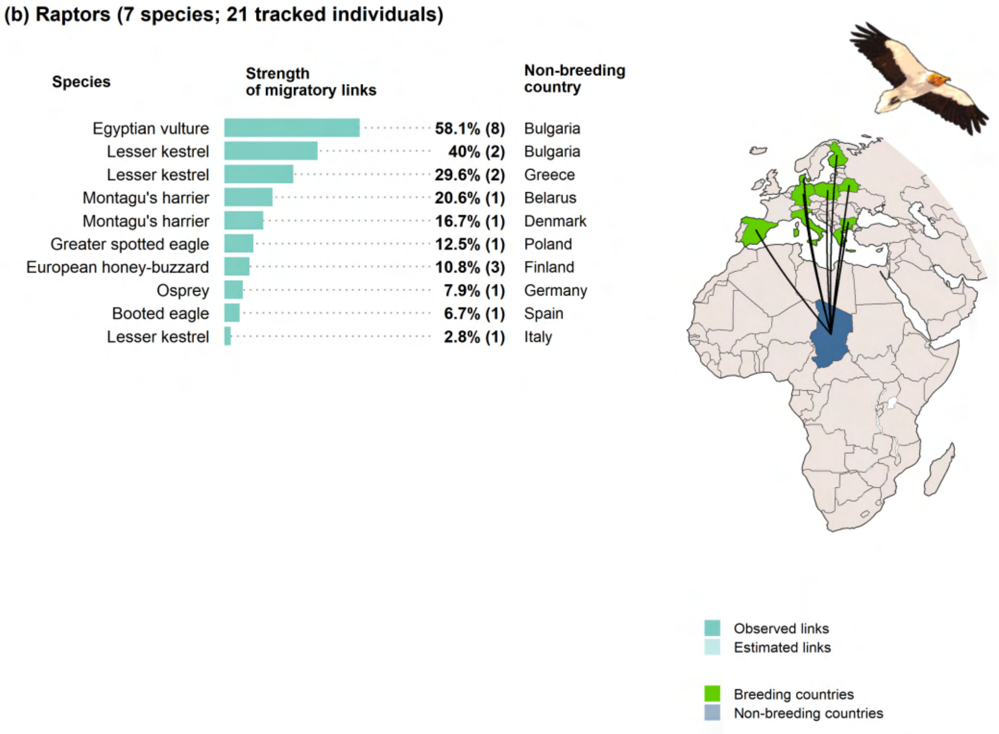

Figure S10.44: Central African Republic

Mapping country-level connectivity for Central African Republic, separately for (a) landbirds and (b) raptors. For each group, we present the list of all migratory links by decreasing order of strength (with respective number of tracked individuals in parenthesis), indicating in each case the species creating the link and the country it connects to. Maps represent how the migratory links (observed: solid lines; inferred: dotted lines) connect the countries in Europe (in green) to countries in sub-Saharan Africa (in blue).

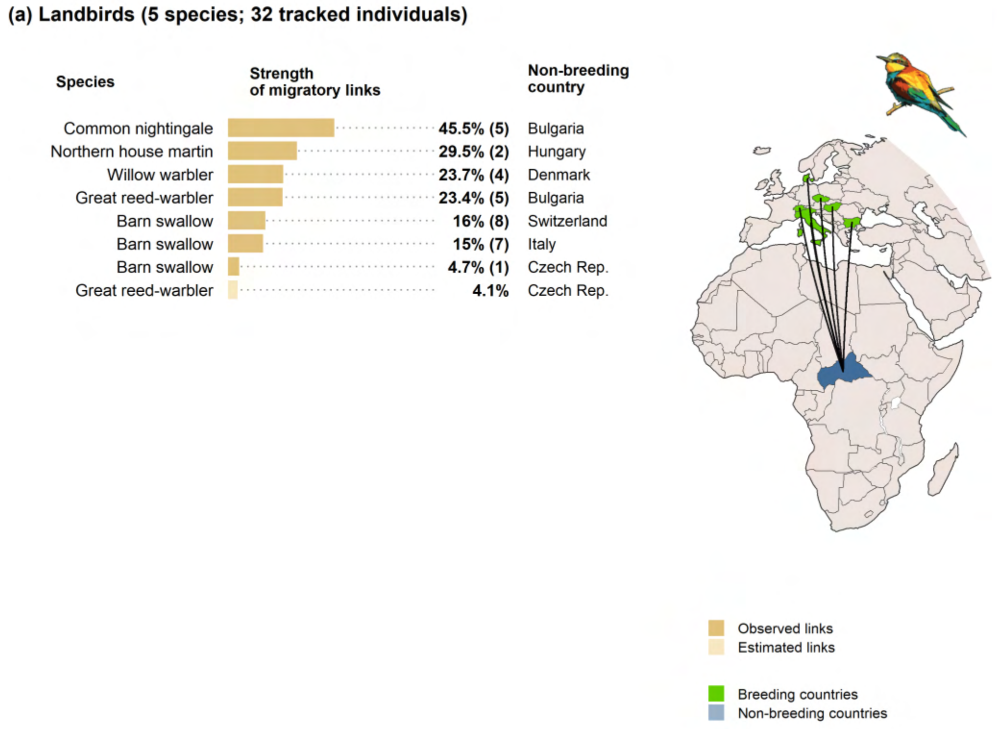

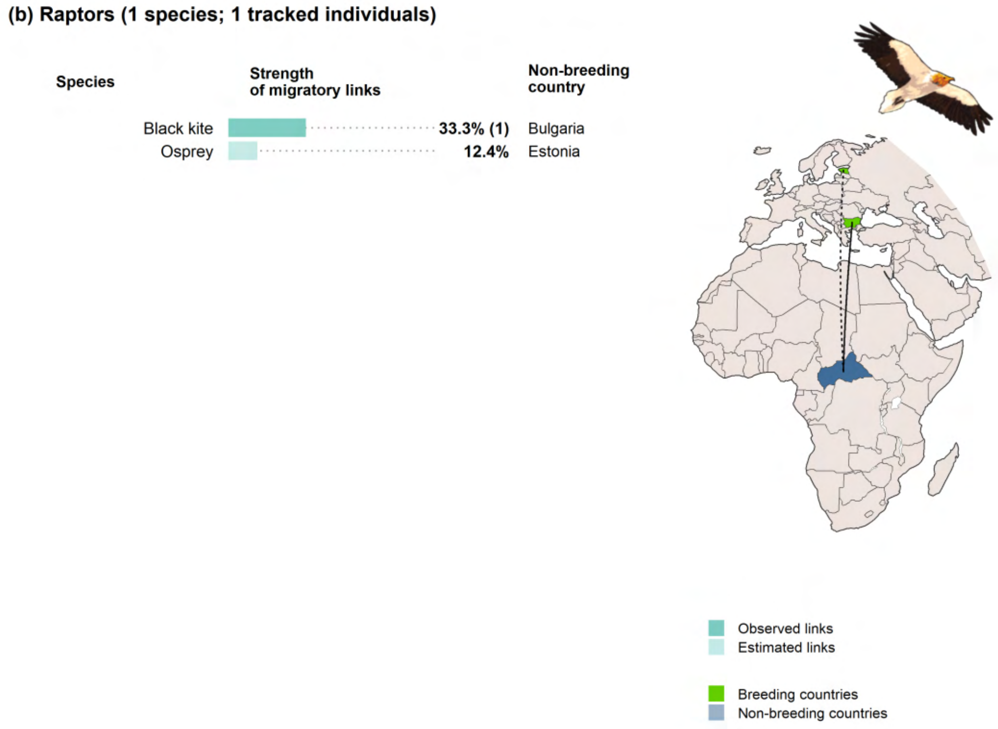

Figure S10.45: Gabon & Equatorial Guinea

Mapping country-level connectivity for Gabon & Equatorial Guinea, separately for (a) landbirds and (b) raptors. For each group, we present the list of all migratory links by decreasing order of strength (with respective number of tracked individuals in parenthesis), indicating in each case the species creating the link and the country it connects to. Maps represent how the migratory links (observed: solid lines; inferred: dotted lines) connect the countries in Europe (in green) to countries in sub-Saharan Africa (in blue).

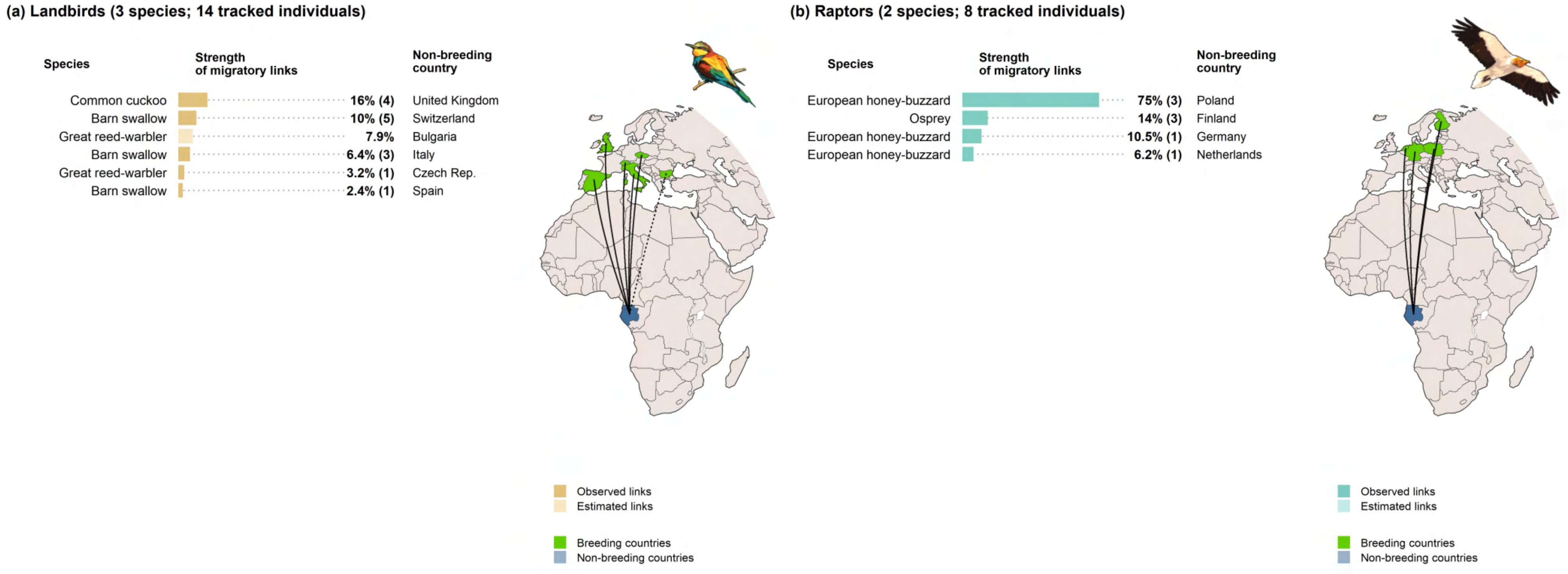

Figure S10.46: Congo & Cabinda (Angola)

Mapping country-level connectivity for Congo & Cabinda (Angola), separately for (a) landbirds and (b) raptors. For each group, we present the list of all migratory links by decreasing order of strength (with respective number of tracked individuals in parenthesis), indicating in each case the species creating the link and the country it connects to. Maps represent how the migratory links (observed: solid lines; inferred: dotted lines) connect the countries in Europe (in green) to countries in sub-Saharan Africa (in blue).

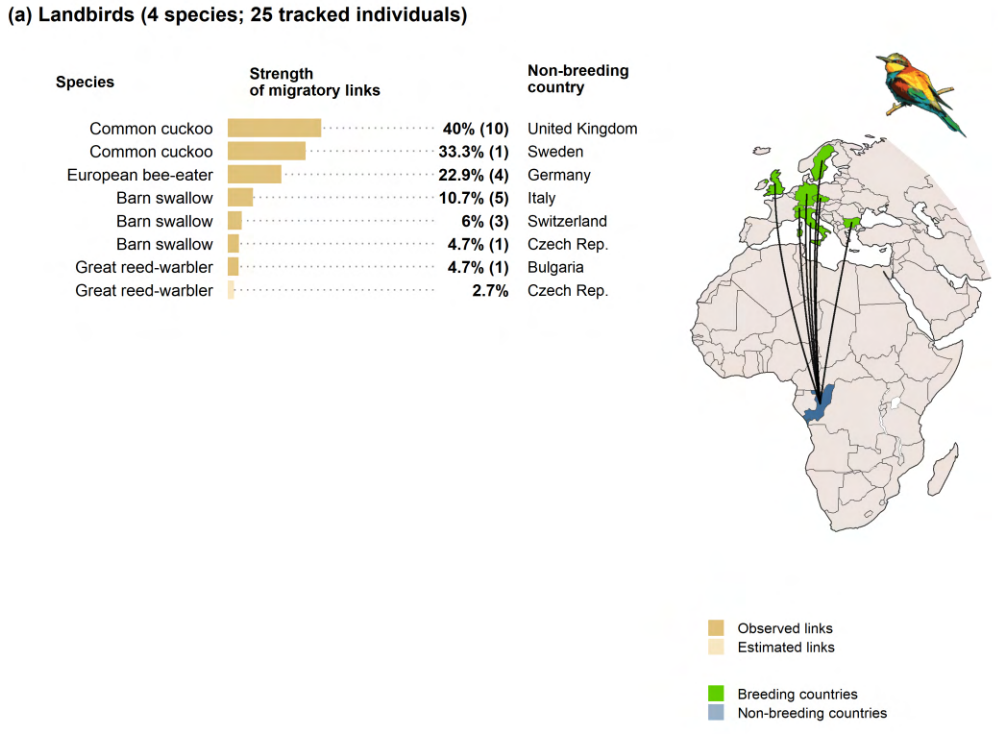

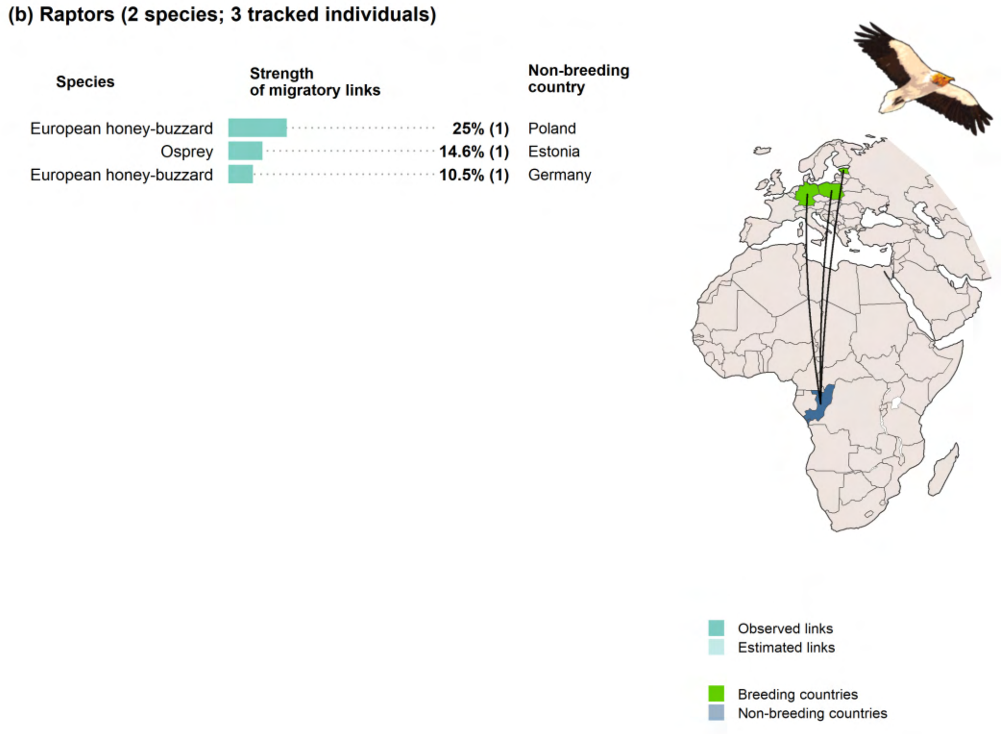

Figure S10.47: Democratic Republic of Congo

Mapping country-level connectivity for Democratic Republic of Congo, separately for (a) landbirds and (b) raptors. For each group, we present the list of all migratory links by decreasing order of strength (with respective number of tracked individuals in parenthesis), indicating in each case the species creating the link and the country it connects to. Maps represent how the migratory links (observed: solid lines; inferred: dotted lines) connect the countries in Europe (in green) to countries in sub-Saharan Africa (in blue).

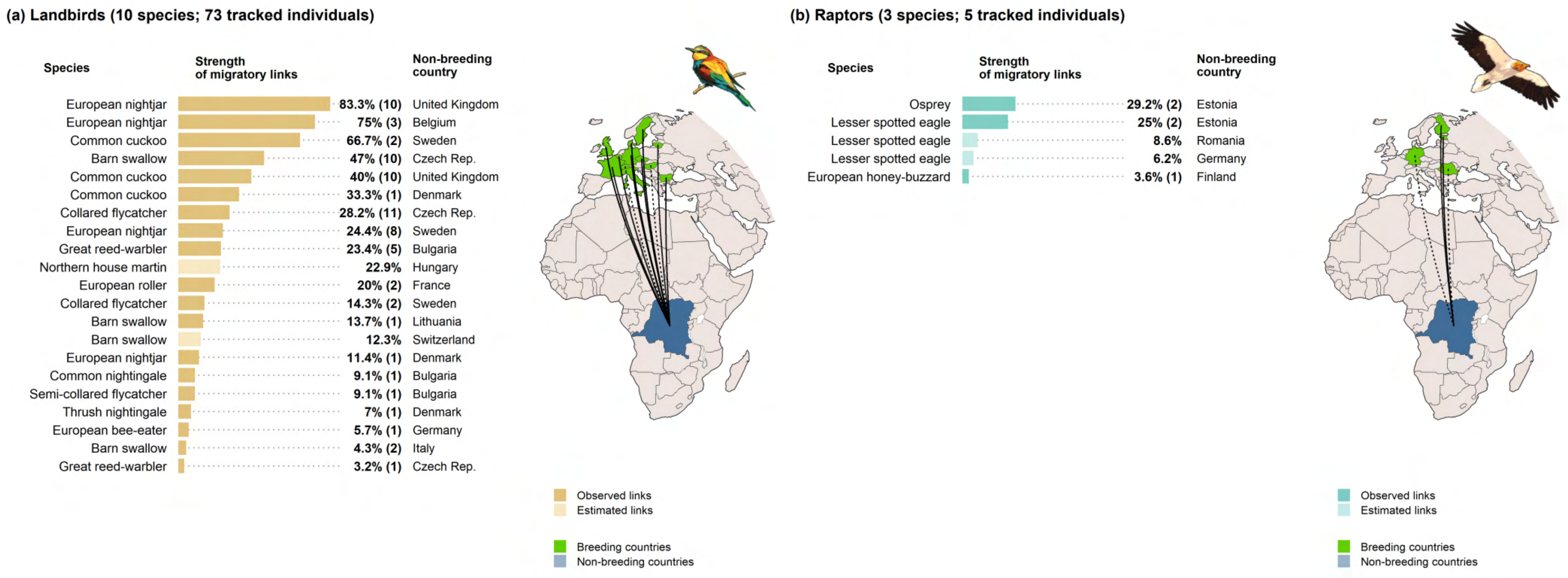

Figure S10.48: Angola

Mapping country-level connectivity for Angola, separately for (a) landbirds and (b) raptors. For each group, we present the list of all migratory links by decreasing order of strength (with respective number of tracked individuals in parenthesis), indicating in each case the species creating the link and the country it connects to. Maps represent how the migratory links (observed: solid lines; inferred: dotted lines) connect the countries in Europe (in green) to countries in sub-Saharan Africa (in blue).

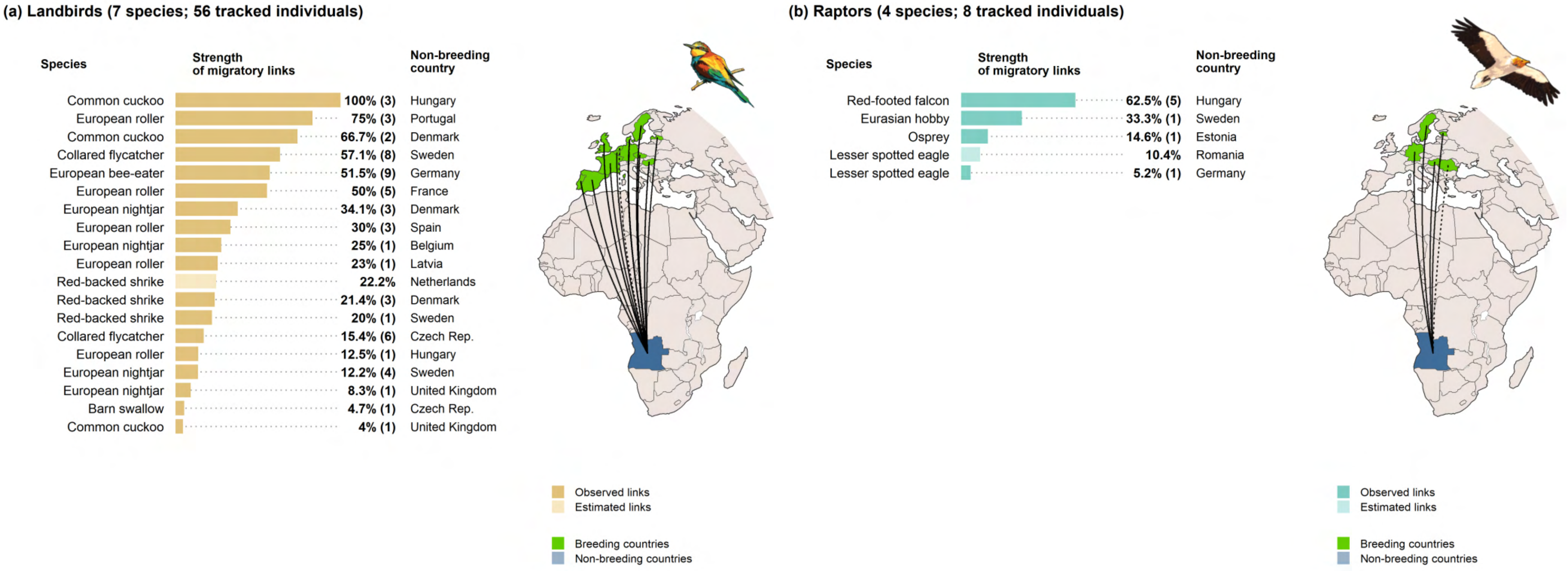

Figure S10.49: Namibia

Mapping country-level connectivity for Namibia, separately for (a) landbirds and (b) raptors. For each group, we present the list of all migratory links by decreasing order of strength (with respective number of tracked individuals in parenthesis), indicating in each case the species creating the link and the country it connects to. Maps represent how the migratory links (observed: solid lines; inferred: dotted lines) connect the countries in Europe (in green) to countries in sub-Saharan Africa (in blue).

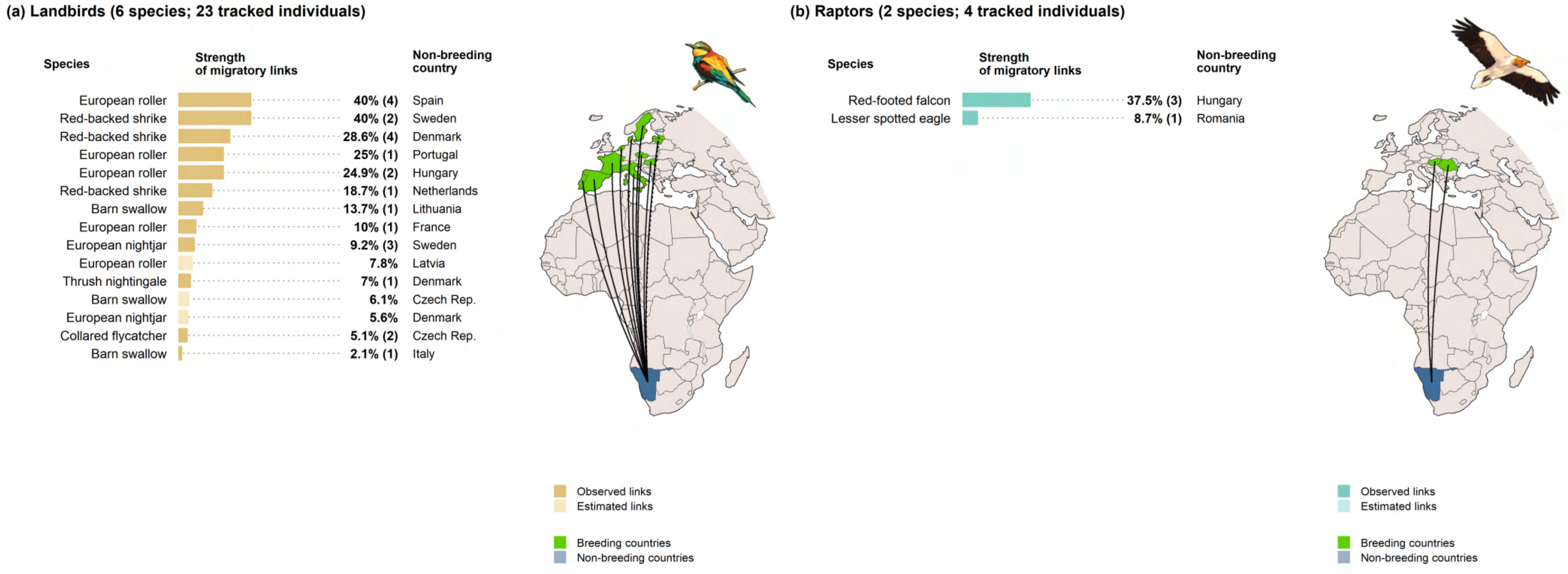

Figure S10.50: South Africa & Swaziland

Mapping country-level connectivity for South Africa & Swaziland, separately for (a) landbirds and (b) raptors. For each group, we present the list of all migratory links by decreasing order of strength (with respective number of tracked individuals in parenthesis), indicating in each case the species creating the link and the country it connects to. Maps represent how the migratory links (observed: solid lines; inferred: dotted lines) connect the countries in Europe (in green) to countries in sub-Saharan Africa (in blue).

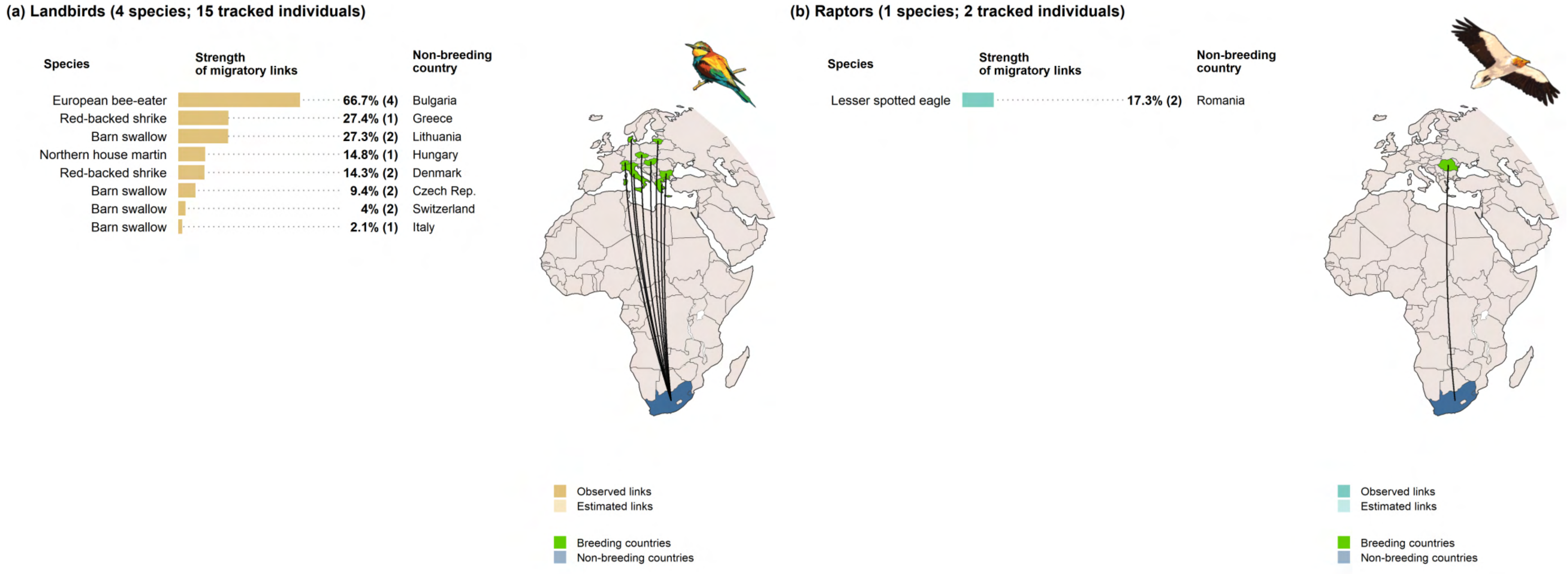

Figure S10.51: Lesotho

Mapping country-level connectivity for Lesotho, separately for (a) landbirds and (b) raptors. For each group, we present the list of all migratory links by decreasing order of strength (with respective number of tracked individuals in parenthesis), indicating in each case the species creating the link and the country it connects to. Maps represent how the migratory links (observed: solid lines; inferred: dotted lines) connect the countries in Europe (in green) to countries in sub-Saharan Africa (in blue).

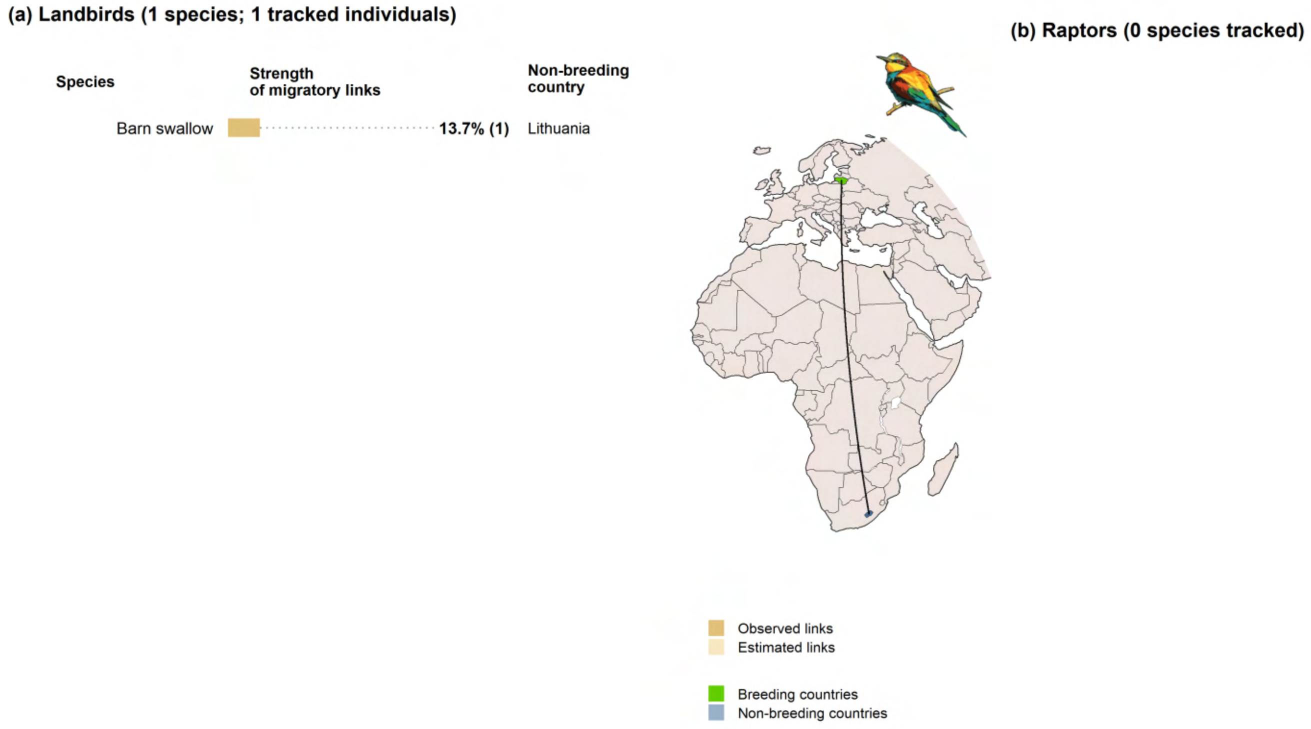

Figure S10.52: Mozambique

Mapping country-level connectivity for Mozambique, separately for (a) landbirds and (b) raptors. For each group, we present the list of all migratory links by decreasing order of strength (with respective number of tracked individuals in parenthesis), indicating in each case the species creating the link and the country it connects to. Maps represent how the migratory links (observed: solid lines; inferred: dotted lines) connect the countries in Europe (in green) to countries in sub-Saharan Africa (in blue).

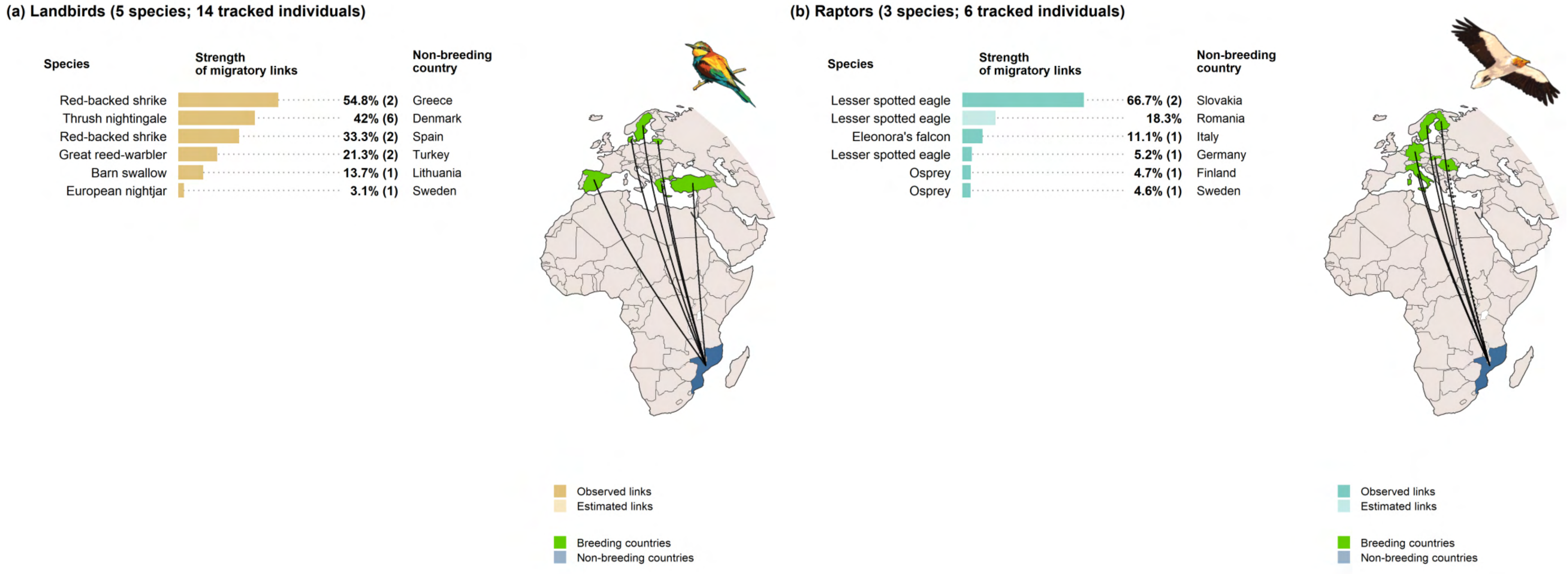

Figure S10.53: Botswana

Mapping country-level connectivity for Botswana, separately for (a) landbirds and (b) raptors. For each group, we present the list of all migratory links by decreasing order of strength (with respective number of tracked individuals in parenthesis), indicating in each case the species creating the link and the country it connects to. Maps represent how the migratory links (observed: solid lines; inferred: dotted lines) connect the countries in Europe (in green) to countries in sub-Saharan Africa (in blue).

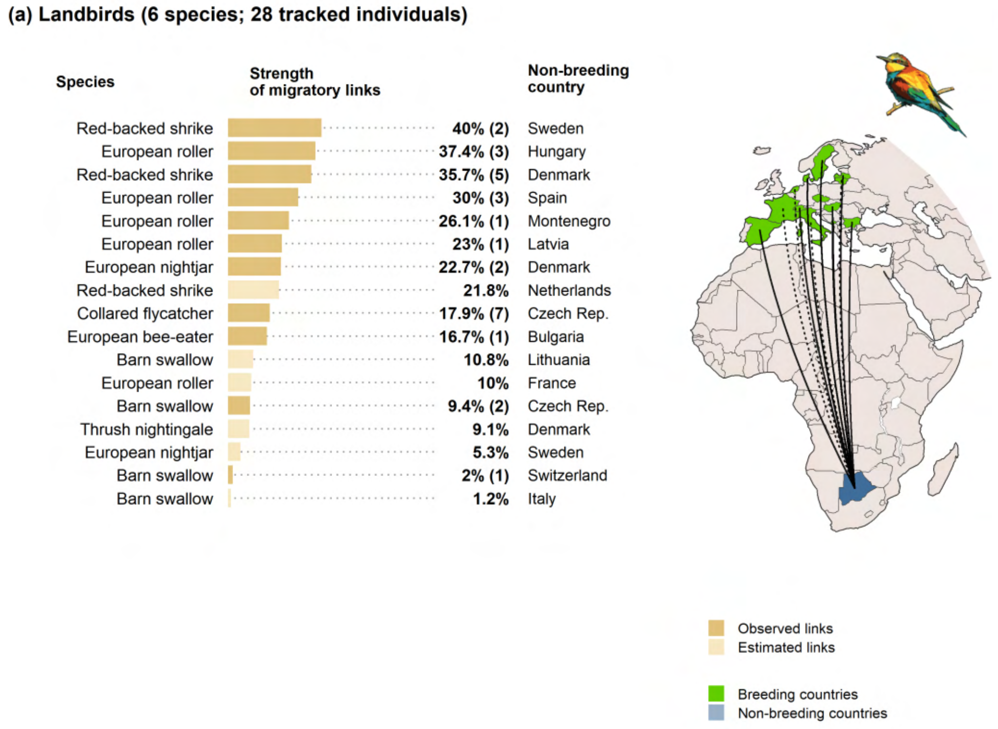

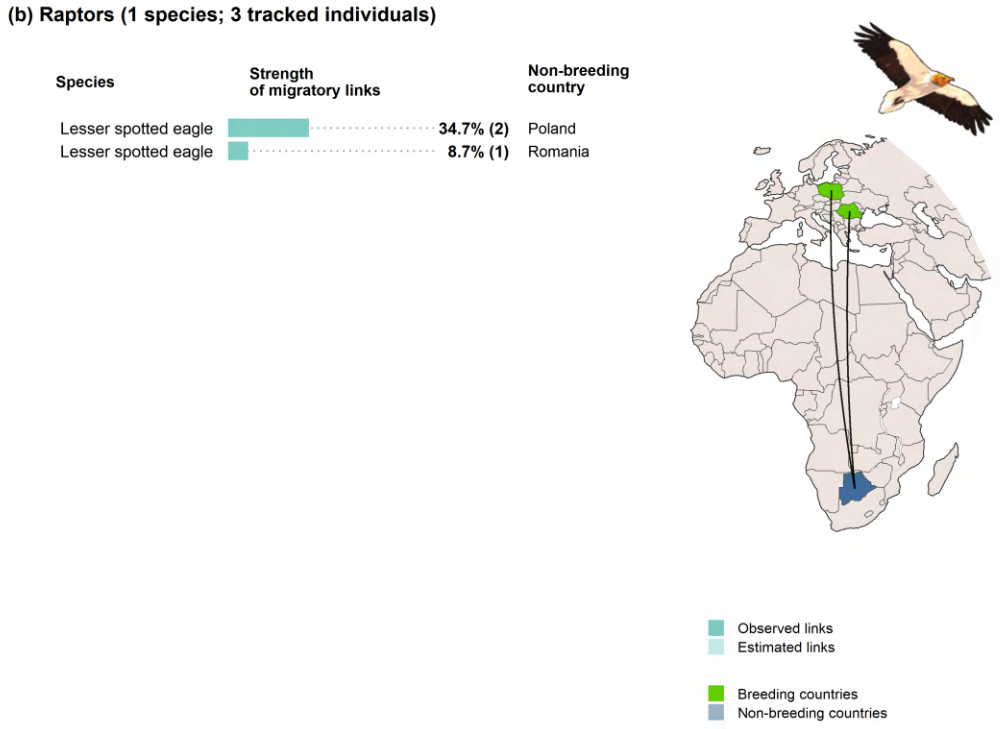

Figure S10.54: Zimbabwe

Mapping country-level connectivity for Zimbabwe, separately for (a) landbirds and (b) raptors. For each group, we present the list of all migratory links by decreasing order of strength (with respective number of tracked individuals in parenthesis), indicating in each case the species creating the link and the country it connects to. Maps represent how the migratory links (observed: solid lines; inferred: dotted lines) connect the countries in Europe (in green) to countries in sub-Saharan Africa (in blue).

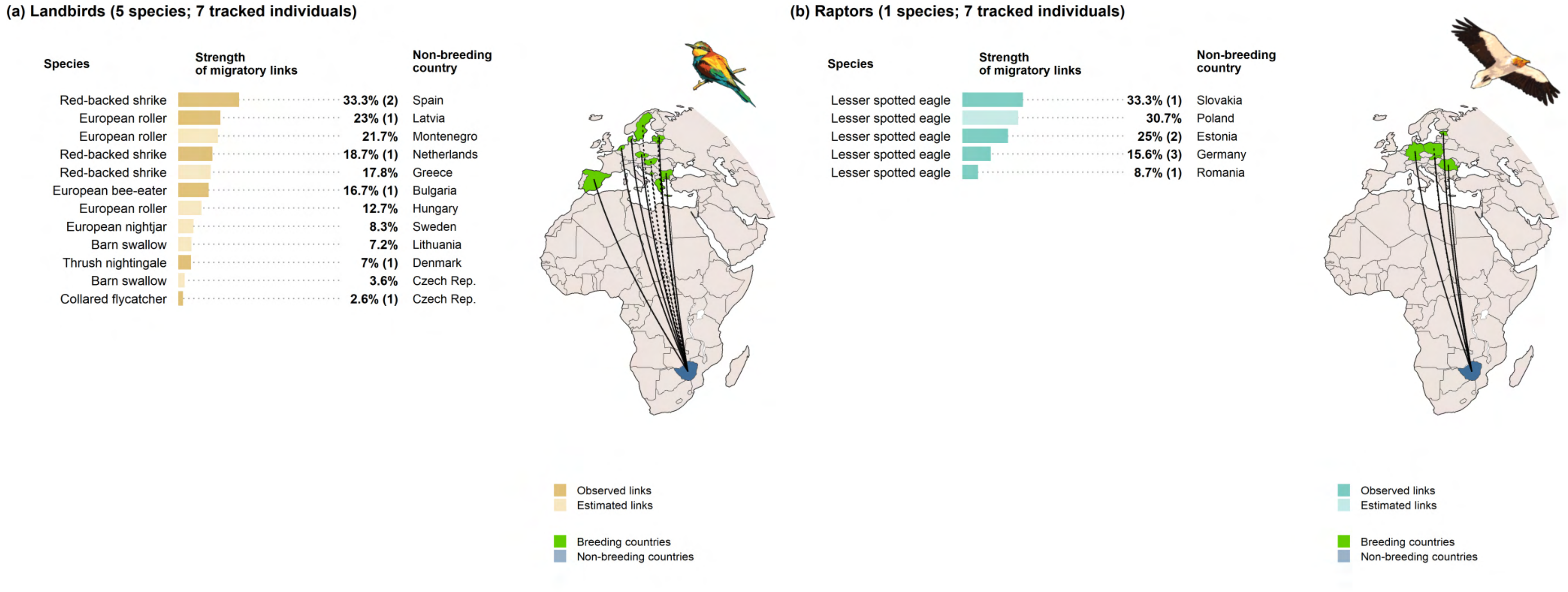

Figure S10.55: Zambia

Mapping country-level connectivity for Zambia, separately for (a) landbirds and (b) raptors. For each group, we present the list of all migratory links by decreasing order of strength (with respective number of tracked individuals in parenthesis), indicating in each case the species creating the link and the country it connects to. Maps represent how the migratory links (observed: solid lines; inferred: dotted lines) connect the countries in Europe (in green) to countries in sub-Saharan Africa (in blue).

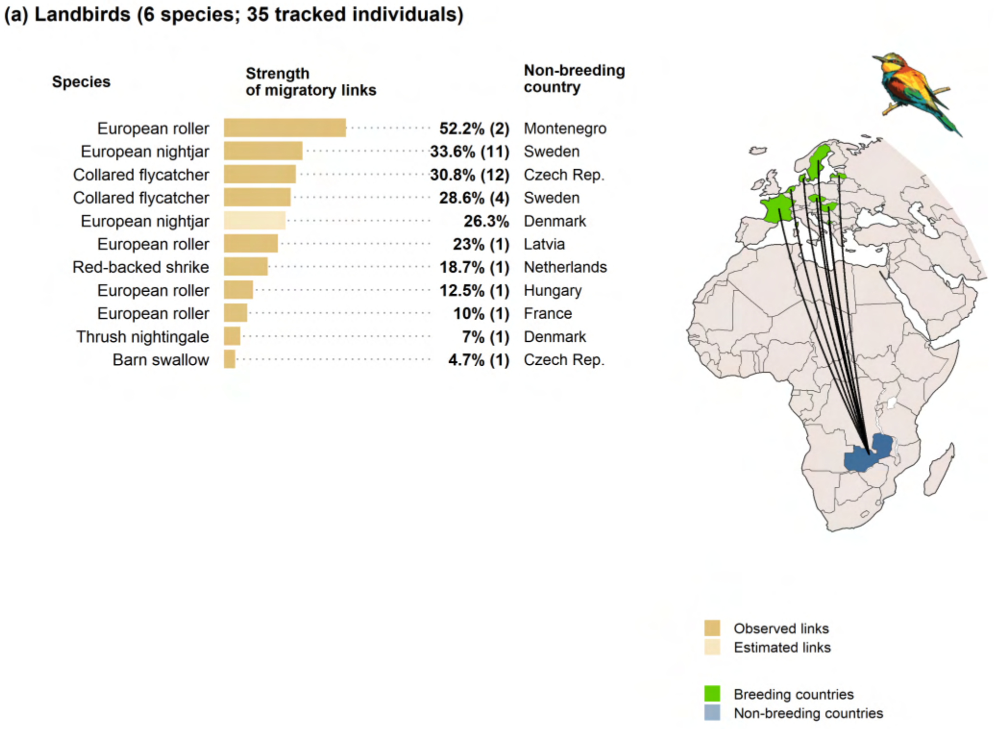

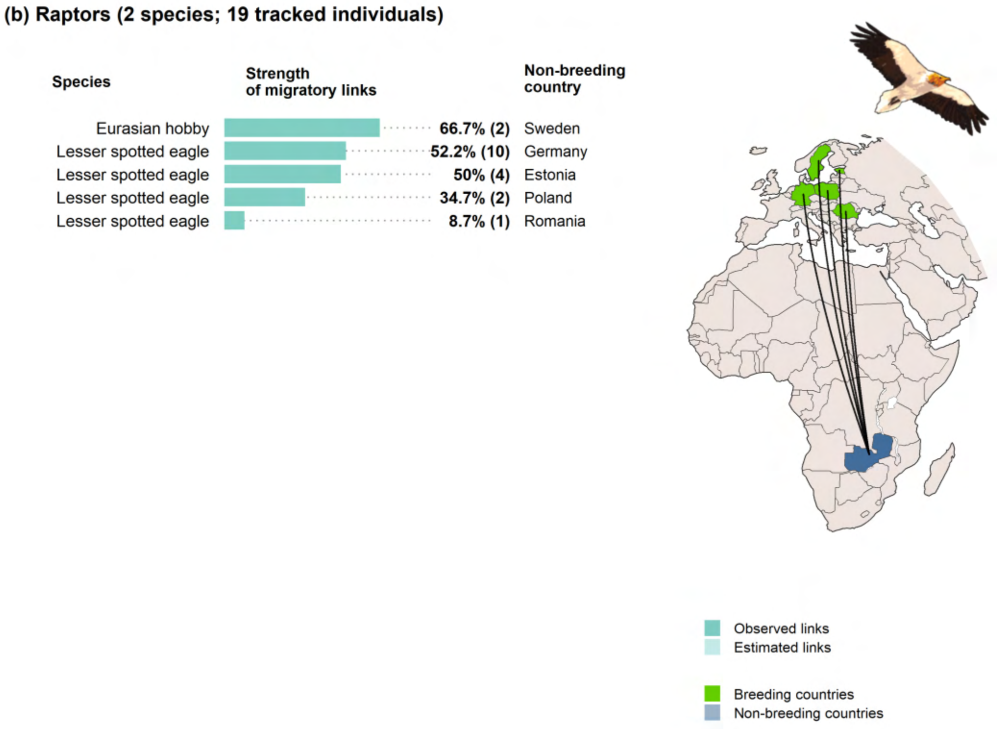

Figure S10.56: Malawi

Mapping country-level connectivity for Malawi, separately for (a) landbirds and (b) raptors. For each group, we present the list of all migratory links by decreasing order of strength (with respective number of tracked individuals in parenthesis), indicating in each case the species creating the link and the country it connects to. Maps represent how the migratory links (observed: solid lines; inferred: dotted lines) connect the countries in Europe (in green) to countries in sub-Saharan Africa (in blue).

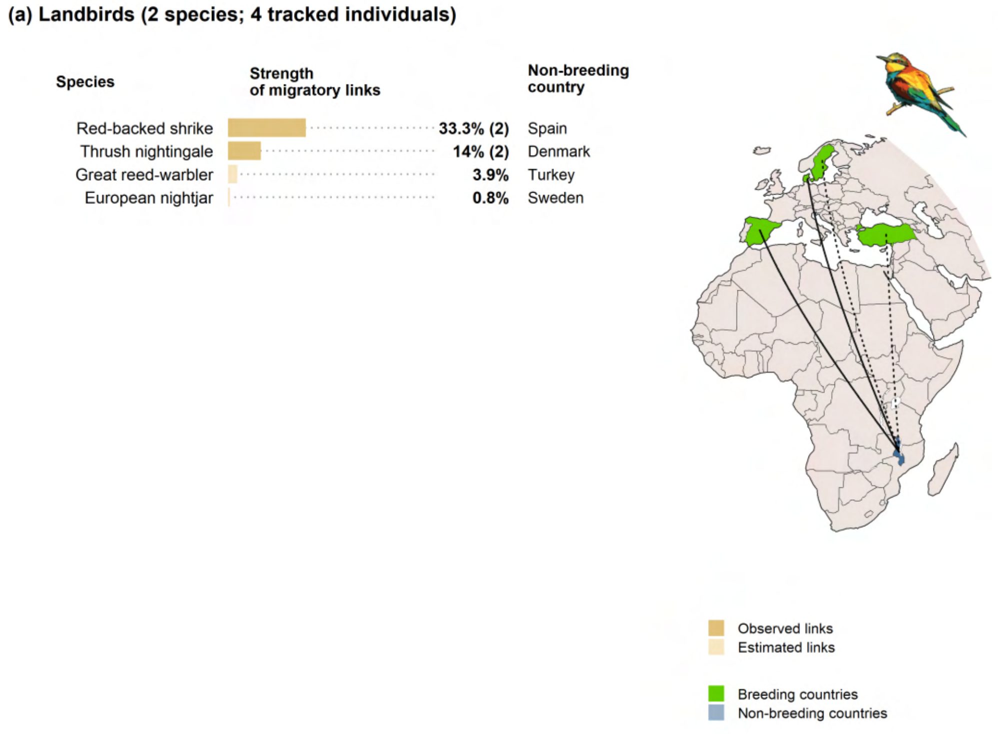

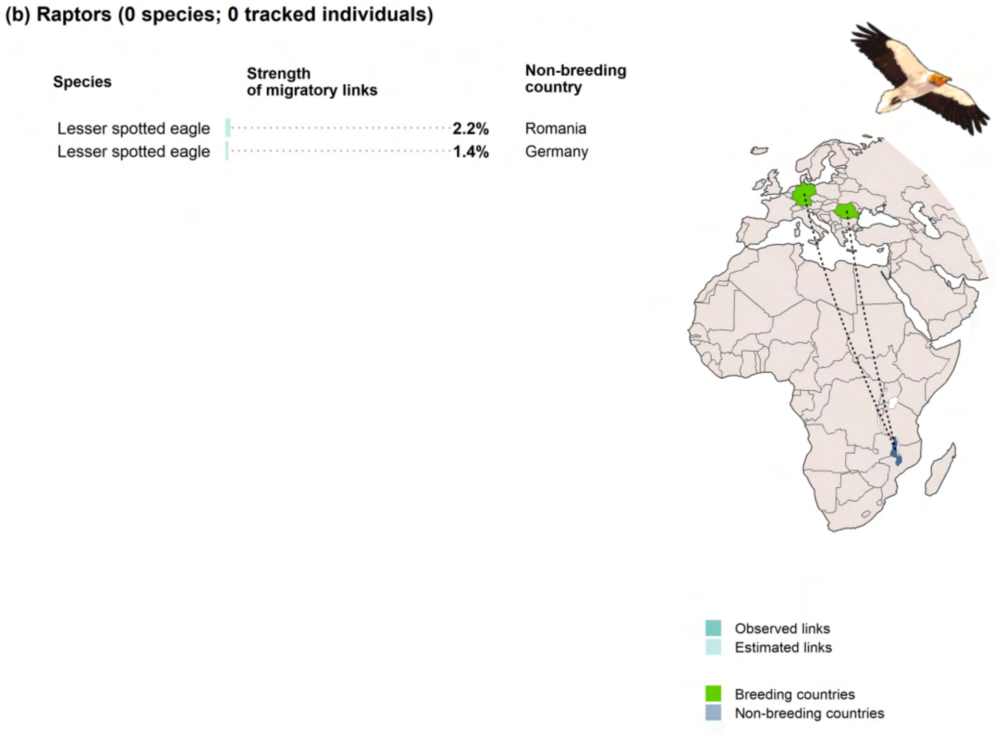

Figure S10.57: Tanzania

Mapping country-level connectivity for Tanzania, separately for (a) landbirds and (b) raptors. For each group, we present the list of all migratory links by decreasing order of strength (with respective number of tracked individuals in parenthesis), indicating in each case the species creating the link and the country it connects to. Maps represent how the migratory links (observed: solid lines; inferred: dotted lines) connect the countries in Europe (in green) to countries in sub-Saharan Africa (in blue).

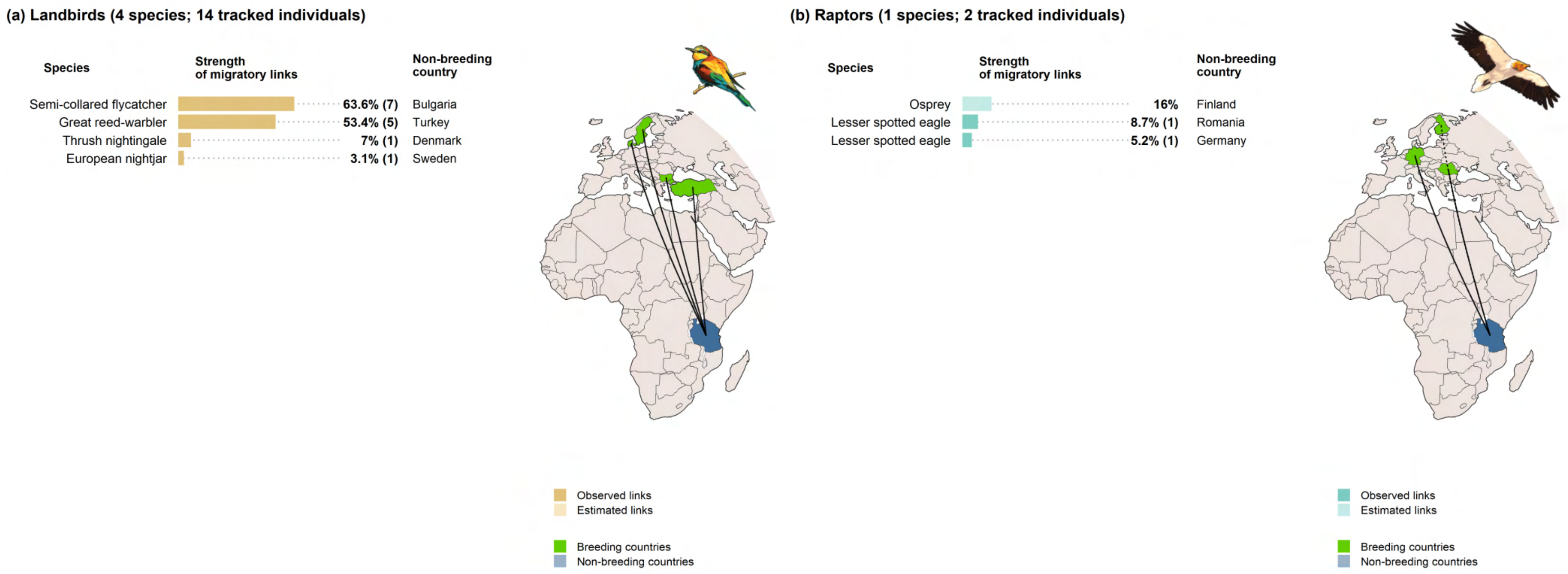

Figure S10.58: Rwanda & Burundi

Mapping country-level connectivity for Rwanda & Burundi, separately for (a) landbirds and (b) raptors. For each group, we present the list of all migratory links by decreasing order of strength (with respective number of tracked individuals in parenthesis), indicating in each case the species creating the link and the country it connects to. Maps represent how the migratory links (observed: solid lines; inferred: dotted lines) connect the countries in Europe (in green) to countries in sub-Saharan Africa (in blue).

(a) Landbirds (2 species; 2 tracked individuals)

Species	Strength of migratory links	Non-breeding country
Semi-collared flycatcher	9.1% (1)	Bulgaria
European nightjar	8.3% (1)	United Kingdom
Barn swallow	1%	Czech Rep.

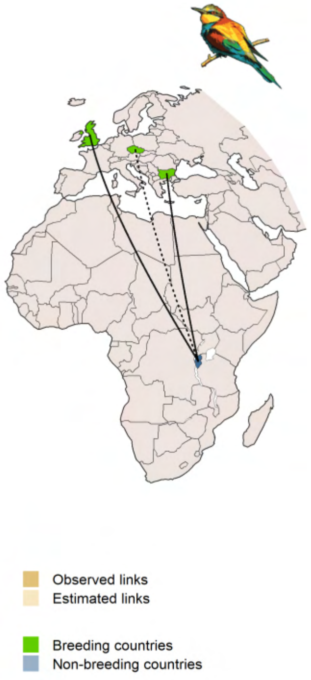

(b) Raptors (1 species; 1 tracked individuals)

Species	Strength of migratory links	Non-breeding country
Osprey	4.7% (1)	Finland
Lesser spotted eagle	0.7%	Germany

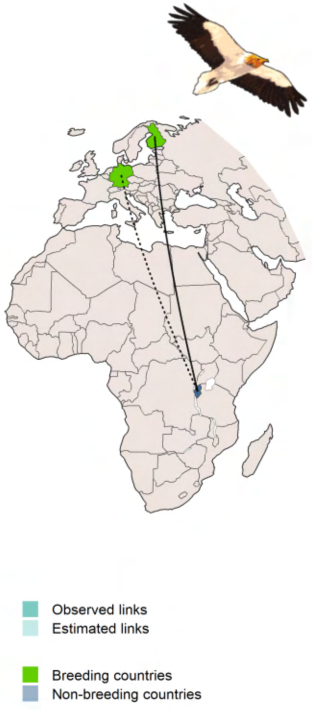

Figure S10.59: Uganda

Mapping country-level connectivity for Uganda, separately for (a) landbirds and (b) raptors. For each group, we present the list of all migratory links by decreasing order of strength (with respective number of tracked individuals in parenthesis), indicating in each case the species creating the link and the country it connects to. Maps represent how the migratory links (observed: solid lines; inferred: dotted lines) connect the countries in Europe (in green) to countries in sub-Saharan Africa (in blue).

(a) Landbirds (4 species; 5 tracked individuals)

Species	Strength of migratory links	Non-breeding country
Semi-collared flycatcher	18.2% (2)	Bulgaria
Northern house martin	14.8% (1)	Hungary
Common nightingale	9.1% (1)	Bulgaria
Great reed-warbler	4.7% (1)	Bulgaria

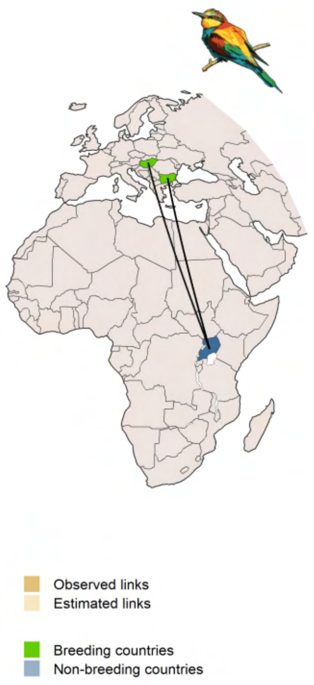

(b) Raptors (1 species; 1 tracked individuals)

Species	Strength of migratory links	Non-breeding country
Black kite	12.5% (1)	Czech Rep.
Lesser spotted eagle	3.1%	Germany

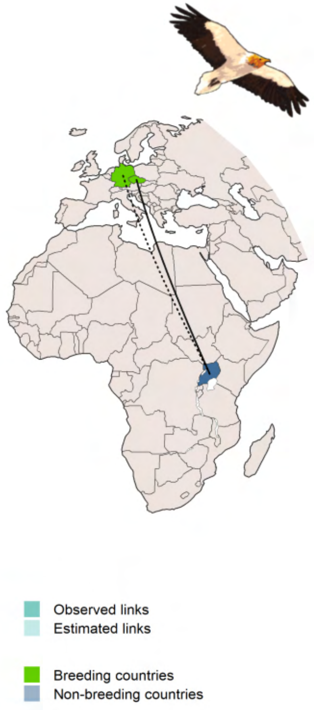

Figure S10.60: Kenya

Mapping country-level connectivity for Kenya, separately for (a) landbirds and (b) raptors. For each group, we present the list of all migratory links by decreasing order of strength (with respective number of tracked individuals in parenthesis), indicating in each case the species creating the link and the country it connects to. Maps represent how the migratory links (observed: solid lines; inferred: dotted lines) connect the countries in Europe (in green) to countries in sub-Saharan Africa (in blue).

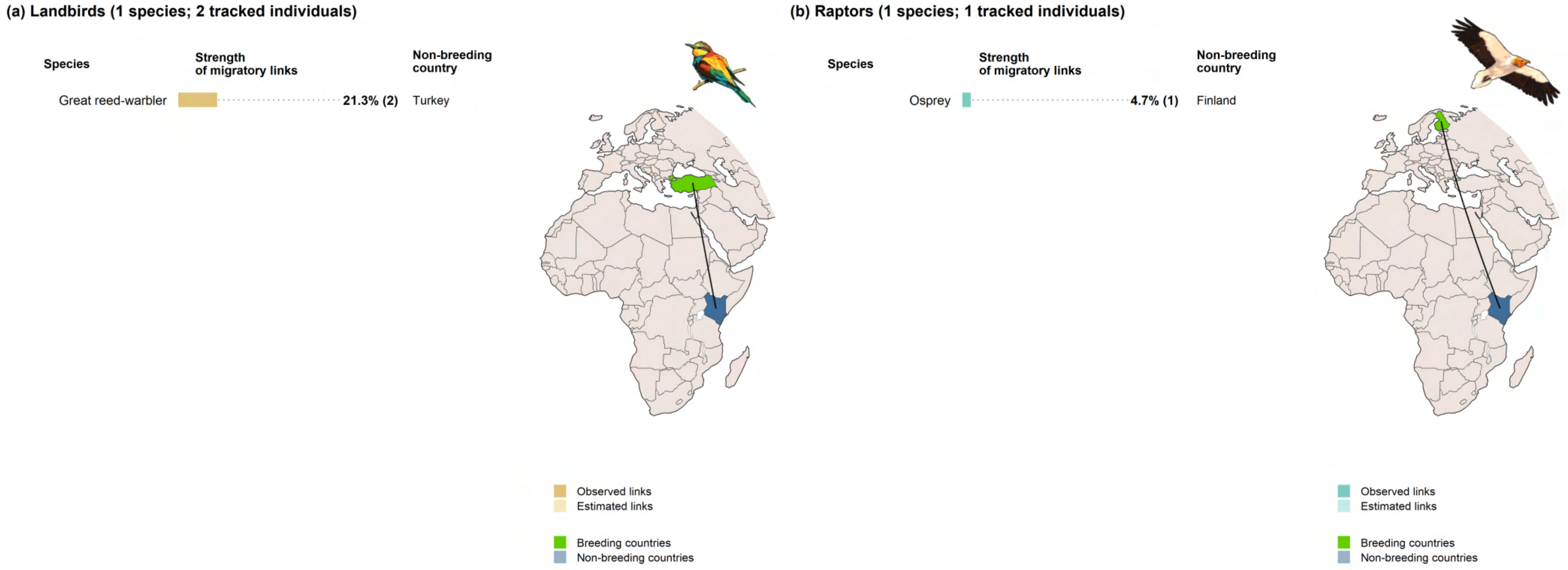

Figure S10.61: Somalia

Mapping country-level connectivity for Somalia, separately for (a) landbirds and (b) raptors. For each group, we present the list of all migratory links by decreasing order of strength (with respective number of tracked individuals in parenthesis), indicating in each case the species creating the link and the country it connects to. Maps represent how the migratory links (observed: solid lines; inferred: dotted lines) connect the countries in Europe (in green) to countries in sub-Saharan Africa (in blue).

(a) Landbirds (0 species tracked)

(b) Raptors (1 species; 1 tracked individuals)

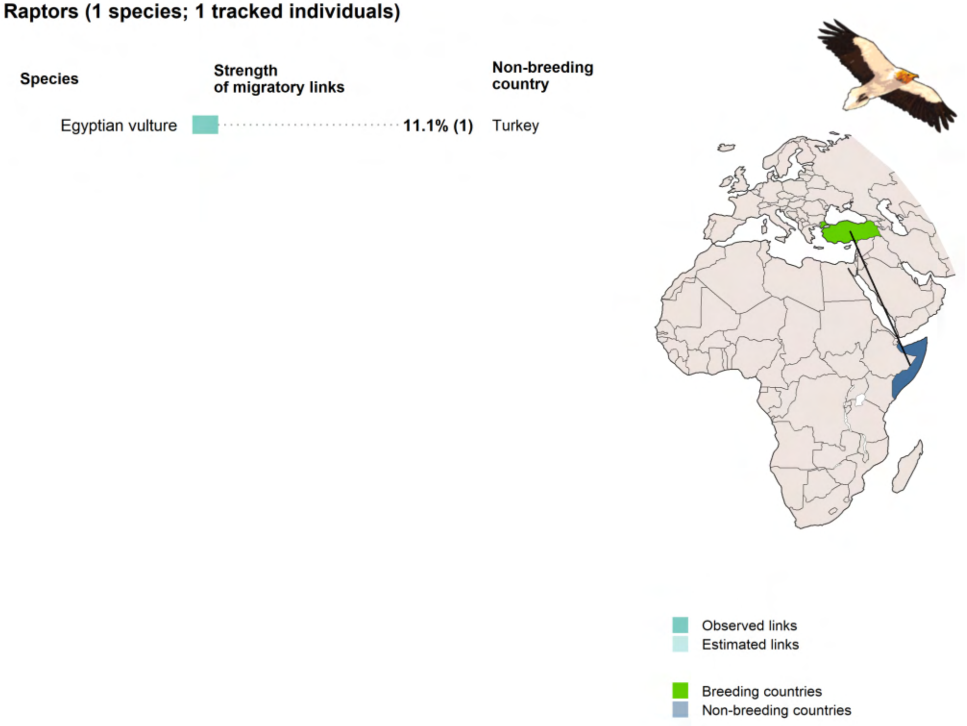

Figure S10.62: Ethiopia & Djibouti

Mapping country-level connectivity for Ethiopia & Djibouti, separately for (a) landbirds and (b) raptors. For each group, we present the list of all migratory links by decreasing order of strength (with respective number of tracked individuals in parenthesis), indicating in each case the species creating the link and the country it connects to. Maps represent how the migratory links (observed: solid lines; inferred: dotted lines) connect the countries in Europe (in green) to countries in sub-Saharan Africa (in blue).

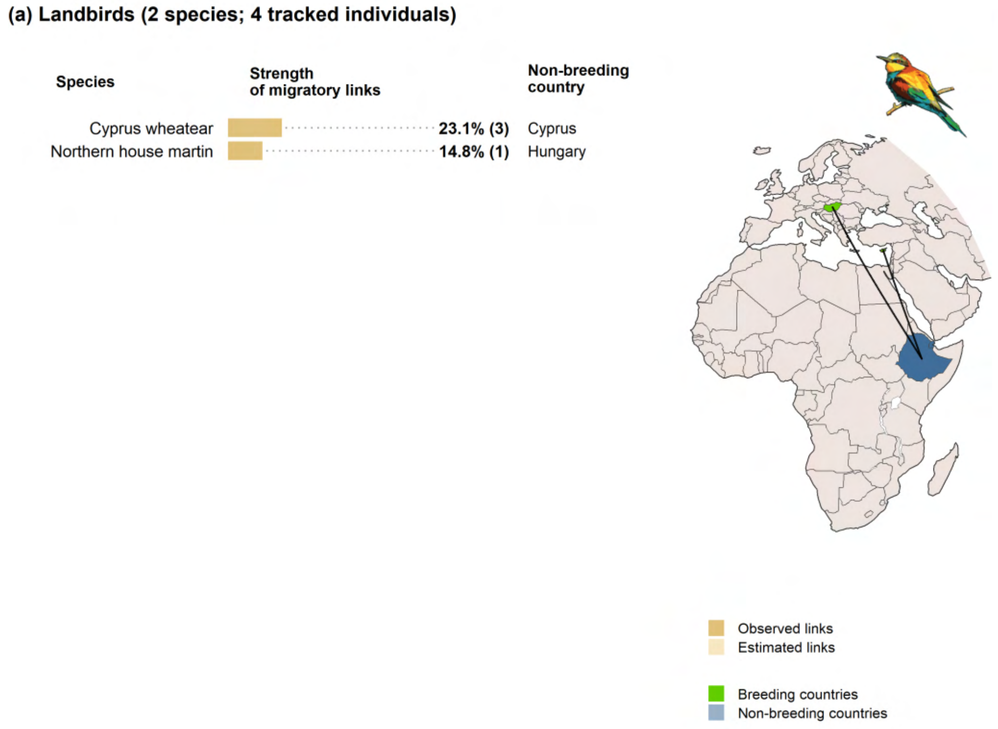

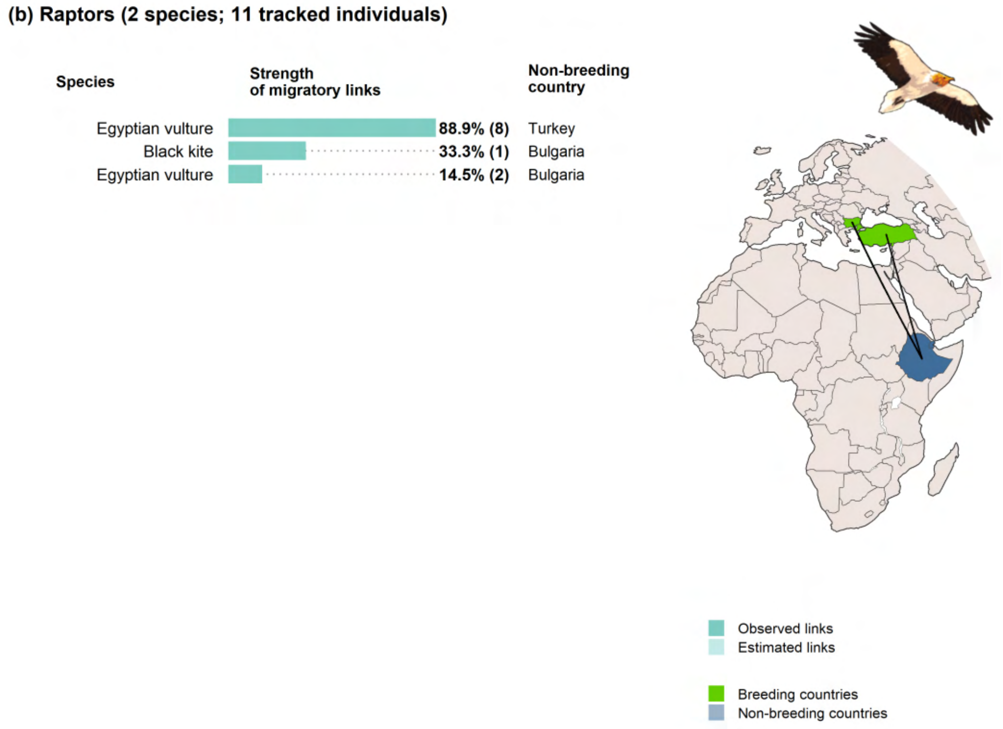

Figure S10.63: South Sudan

Mapping country-level connectivity for South Sudan, separately for (a) landbirds and (b) raptors. For each group, we present the list of all migratory links by decreasing order of strength (with respective number of tracked individuals in parenthesis), indicating in each case the species creating the link and the country it connects to. Maps represent how the migratory links (observed: solid lines; inferred: dotted lines) connect the countries in Europe (in green) to countries in sub-Saharan Africa (in blue).

(a) Landbirds (3 species; 3 tracked individuals)

Species	Strength of migratory links	Non-breeding country
Common nightingale	9.1% (1)	Bulgaria
Cyprus wheatear	7.7% (1)	Cyprus
Barn swallow	4.7% (1)	Czech Rep.
Northern house martin	3.2%	Hungary

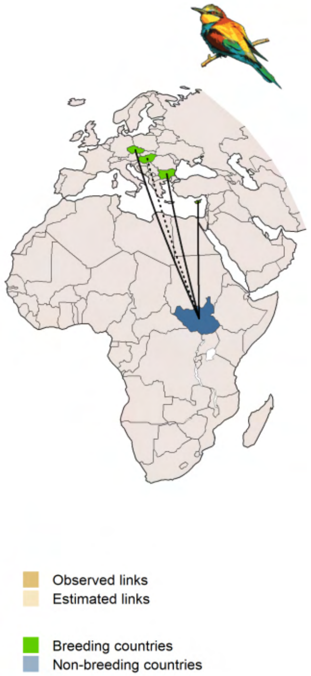

(b) Raptors (4 species; 7 tracked individuals)

Species	Strength of migratory links	Non-breeding country
Greater spotted eagle	50% (4)	Poland
Black kite	33.3% (1)	Bulgaria
Osprey	14.6% (1)	Estonia
Egyptian vulture	5.6%	Bulgaria
Lesser spotted eagle	5.2% (1)	Germany

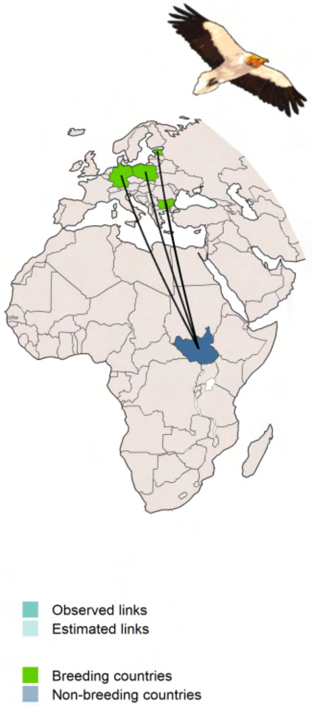

Figure S10.64: Sudan

Mapping country-level connectivity for Sudan, separately for (a) landbirds and (b) raptors. For each group, we present the list of all migratory links by decreasing order of strength (with respective number of tracked individuals in parenthesis), indicating in each case the species creating the link and the country it connects to. Maps represent how the migratory links (observed: solid lines; inferred: dotted lines) connect the countries in Europe (in green) to countries in sub-Saharan Africa (in blue).

(a) Landbirds (1 species; 9 tracked individuals)

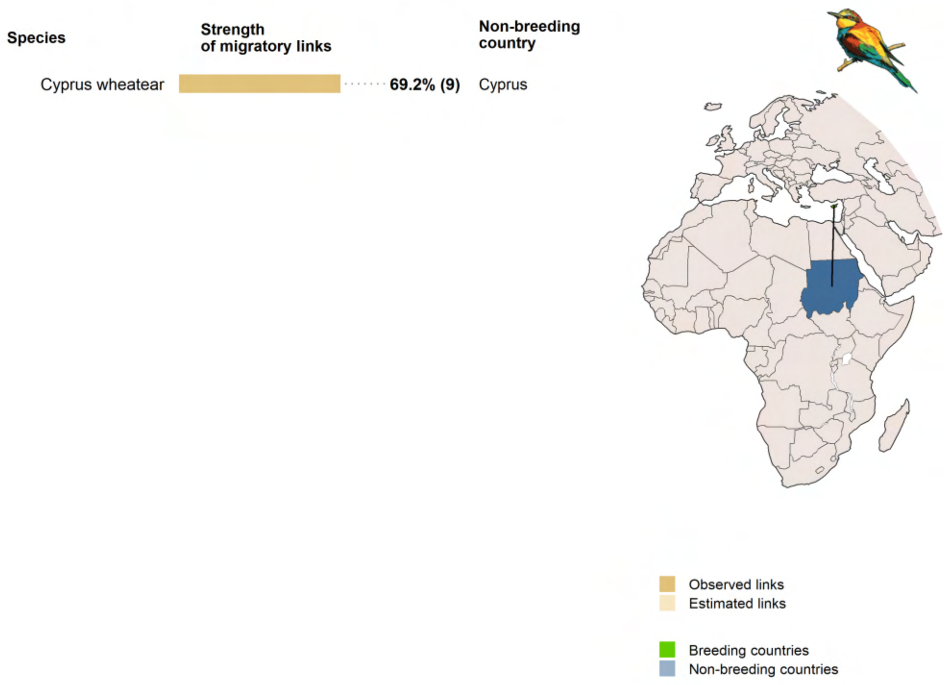

(b) Raptors (3 species; 7 tracked individuals)

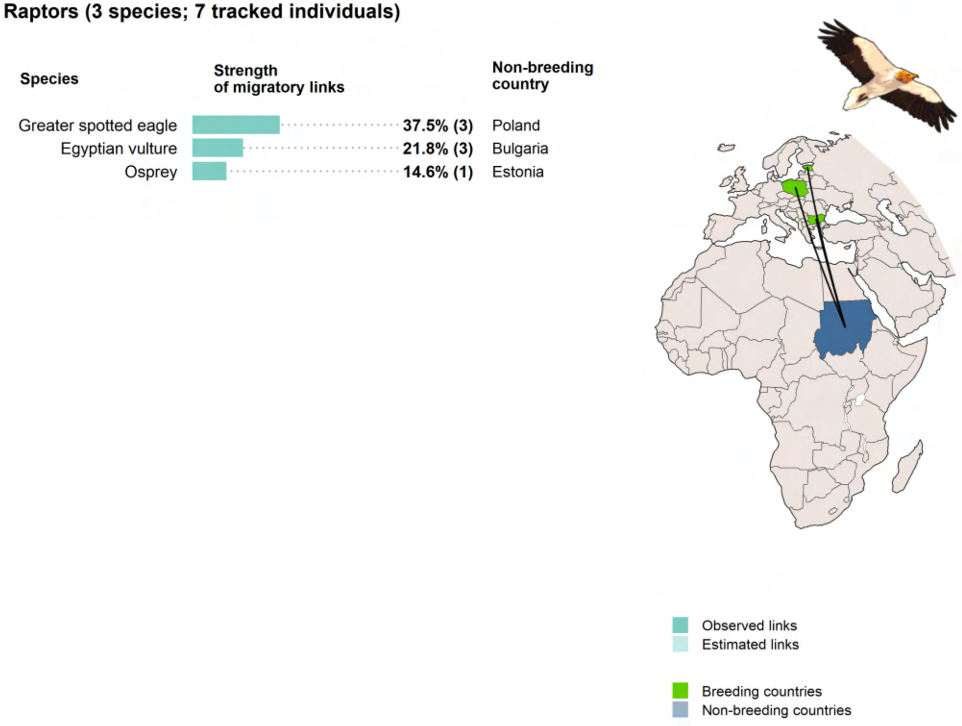

Figure S10.65: Madagascar

Mapping country-level connectivity for Madagascar, separately for (a) landbirds and (b) raptors. For each group, we present the list of all migratory links by decreasing order of strength (with respective number of tracked individuals in parenthesis), indicating in each case the species creating the link and the country it connects to. Maps represent how the migratory links (observed: solid lines; inferred: dotted lines) connect the countries in Europe (in green) to countries in sub-Saharan Africa (in blue).

(a) Landbirds (0 species tracked)

(b) Raptors (1 species; 29 tracked individuals)

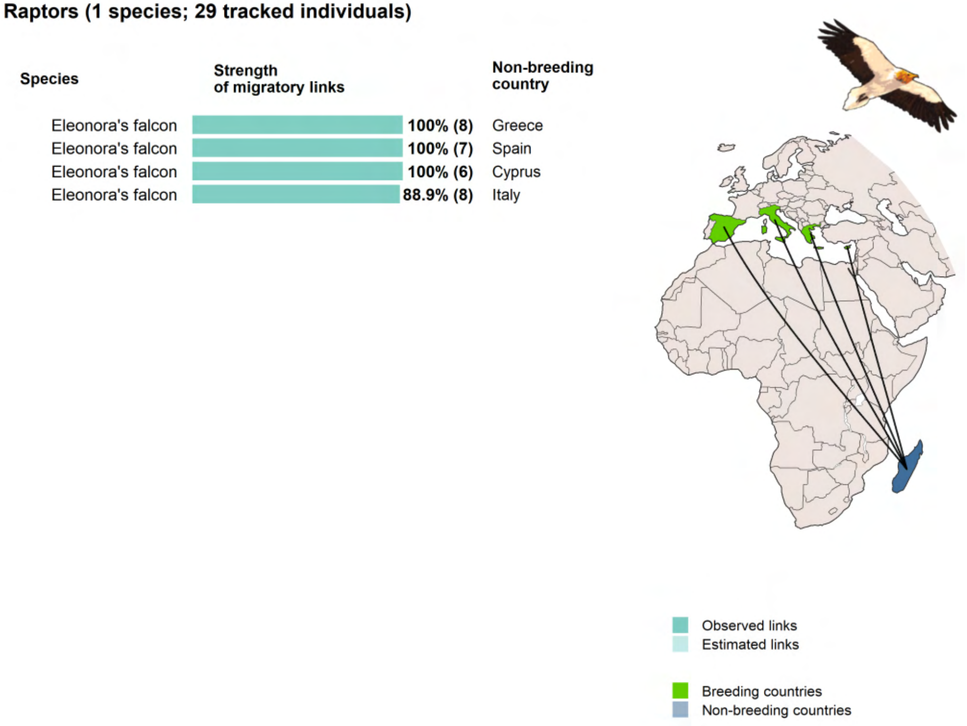

Supplement: Supplementary file 4 — Appendix S10. Mapping country‐level connectivity for all countries in Europe and sub‐Saharan Africa. [file COBI-37-0-s005.pdf]
