# Supplementary material for: Connectivity between countries established by landbirds and raptors migrating along the African–Eurasian flyway
Source: Conserv Biol. 2022 Dec 15;37(1):e14002. doi: 10.1111/cobi.14002 (PMC10107209; doi:10.1111/cobi.14002)

Connectivity between countries established by landbirds and raptors migrating along the African-Eurasian flyway

Guilherme et al. (2022). Conservation Biology. DOI: 10.1111/cobi.14002

Appendix S11.

Mapping species-level connectivity for species of long-distance migratory landbirds and raptors.

The following 43 figures synthesize species-level connectivity as reveled by the migratory links (observed and inferred), for each species of long-distance migratory landbirds and raptors. For each species, we present connectivity between countries, with each map corresponding to the set of birds that breed in a given European country (i.e., one map for each population tracked). We also present the importance of each country in sub-Saharan Africa as nonbreeding grounds for the European population of each species in our dataset.

A migratory link represents the connection between two countries established by birds from a population as they migrate from a European breeding country to a sub-Saharan African non-breeding country. The strength of a migratory link represents the proportion of individuals in a population that spend the nonbreeding season in a given country in sub-Saharan Africa, and was estimated from the distribution of migration records (observed and interpolated) among the migratory links in each population (details in Appendices S4, S7 & S9).

For analysis, we merged the smaller sub-Saharan countries (those with latitudinal and/or longitudinal extents <200 km) to their neighboring countries: Gambia to Senegal; Togo to Benin; Equatorial Guinea to Gabon; Burundi to Rwanda; Djibouti to Ethiopia; Swaziland to South Africa; and Angola's enclave Cabinda to the Republic of Congo (details in Appendix S1).

List of Figures

Landbirds

Figure S11.1: European turtle-dove (*Streptopelia turtur*) 4

Figure S11.2: European nightjar (*Caprimulgus europaeus*) 5

Figure S11.3: Alpine swift (*Tachymarptis melba*) 6

Figure S11.4: White-rumped swift (*Apus caffer*) 7

Figure S11.5: Great spotted cuckoo (*Clamator glandarius*) 8

Figure S11.6: Common cuckoo (*Cuculus canorus*) 9

Figure S11.7: Common hoopoe (*Upupa epops*) 10

Figure S11.8: European bee-eater (*Merops apiaster*) 11

Figure S11.9: European roller (*Coracias garrulus*) 12

Figure S11.10: Red-backed shrike (*Lanius collurio*) 13

Figure S11.11: Aquatic warbler (*Acrocephalus paludicola*) 14

Figure S11.12: Common reed-warbler (*Acrocephalus scirpaceus*) 15

Figure S11.13: Great reed-warbler (*Acrocephalus arundinaceus*) 16

Figure S11.14: Northern house martin (*Delichon urbicum*) 17

Figure S11.15: Barn swallow (*Hirundo rustica*) 18

Figure S11.16: Collared sand martin (*Riparia riparia*) 19

Figure S11.17: Willow warbler (*Phylloscopus trochilus*) 20

Figure S11.18: Rufous-tailed scrub-robin (*Cercotrichas galactotes*) 21

Figure S11.19: Thrush nightingale (*Luscinia luscinia*) 22

Figure S11.20: Common nightingale (*Luscinia megarhynchos*) 23

Figure S11.21: Semi-collared flycatcher (*Ficedula semitorquata*) 24

Figure S11.22: European pied flycatcher (*Ficedula hypoleuca*) 25

Figure S11.23: Collared flycatcher (*Ficedula albicollis*) 26

Figure S11.24: Common redstart (*Phoenicurus phoenicurus*) 27

Figure S11.25: Whinchat (*Saxicola rubetra*) 28

Figure S11.26: Northern wheatear (*Oenanthe oenanthe*) 29

Figure S11.27: Cyprus wheatear (*Oenanthe cypriaca*) 30

Figure S11.28: Tawny pipit (*Anthus campestris*) 31

Figure S11.29: Ortolan bunting (*Emberiza hortulana*) 32

Raptors

Figure S11.30: Osprey (*Pandion haliaetus*) 33

Figure S11.31: European honey-buzzard (*Pernis apivorus*) 34

Figure S11.32: Egyptian vulture (*Neophron percnopterus*) 35

Figure S11.33: Short-toed snake-eagle (*Circaetus gallicus*) 36

Figure S11.34: Lesser spotted eagle (*Clanga pomarina*) 37

Figure S11.35: Greater spotted eagle (*Clanga clanga*) 38

Figure S11.36: Booted eagle (*Hieraaetus pennatus*) 39

Figure S11.37: Western marsh-harrier (*Circus aeruginosus*) 40

Figure S11.38: Montagu’s harrier (*Circus pygargus*) 42

Figure S11.39: Black kite (*Milvus migrans*) 44

Figure S11.40: Lesser kestrel (*Falco naumanni*) 45

Figure S11.41: Red-footed falcon (*Falco vespertinus*) 46

Figure S11.42: Eleonora’s falcon (*Falco eleonorae*) 47

Figure S11.43: Eurasian hobby (*Falco subbuteo*) 48

Figure S11.1: European turtle-dove (*Streptopelia turtur*)

Mapping species-level connectivity for the European turtle-dove. (a) Importance of each country in sub-Saharan Africa as nonbreeding grounds for the European population, as revealed by the migration links (observed: solid line, inferred: dotted line) weighted by the percentage of the European population estimated to establish the link; countries in sub-Saharan Africa are colored according to the percentage of the total European population they host during the nonbreeding season. (b - d) Connectivity for each population in our dataset; lines represent the migratory links (observed and inferred), with their respective strength indicated alongside the map (asterisks indicate inferred links).

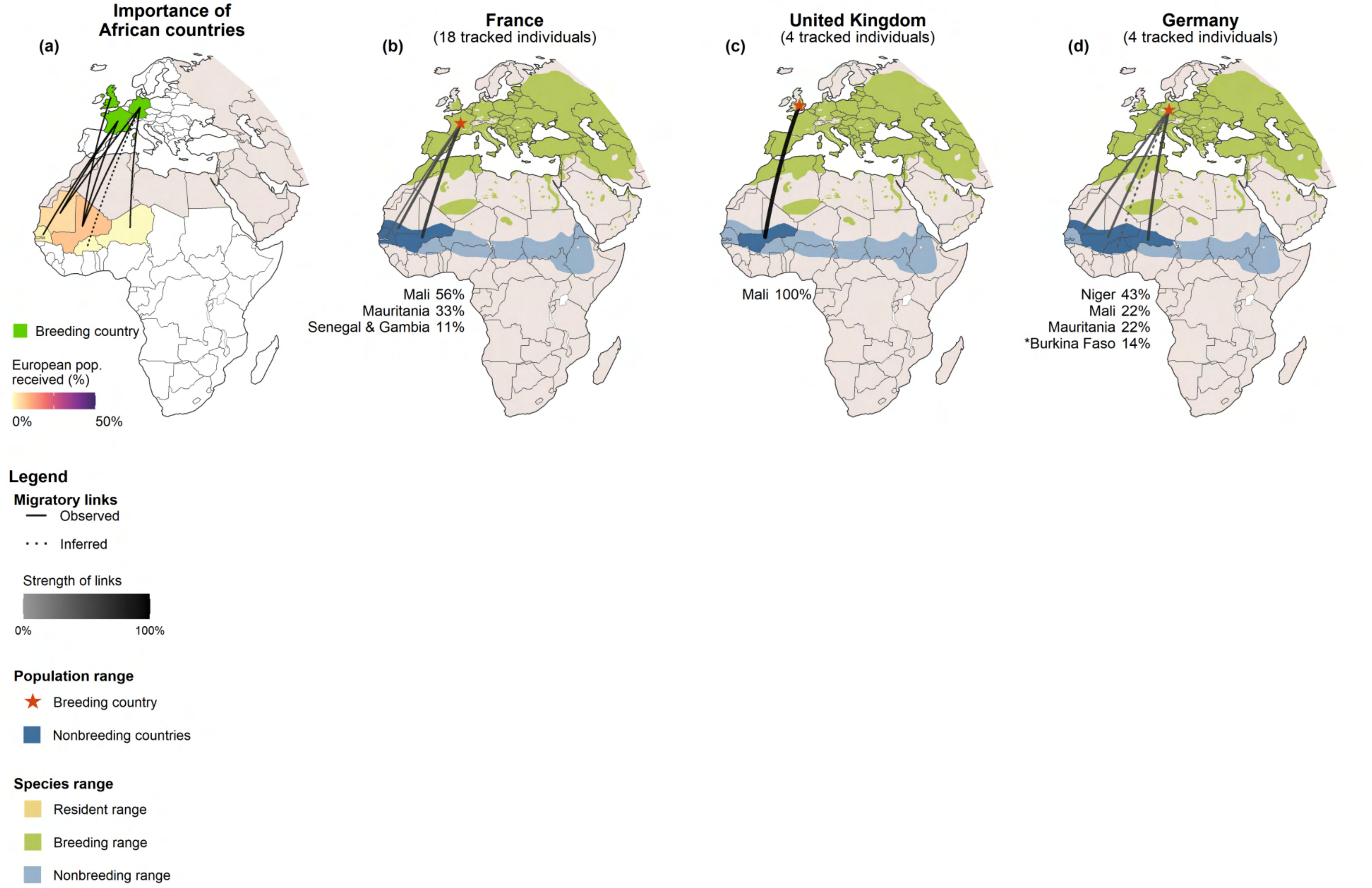

Figure S11.2: European nightjar (*Caprimulgus europaeus*)

Mapping species-level connectivity for the European nightjar. (a) Importance of each country in sub-Saharan Africa as nonbreeding grounds for the European population, as revealed by the migration links (observed: solid line, inferred: dotted line) weighted by the percentage of the European population estimated to establish the link; countries in sub-Saharan Africa are colored according to the percentage of the total European population they host during the nonbreeding season. (b - e) Connectivity for each population in our dataset; lines represent the migratory links (observed and inferred), with their respective strength indicated alongside the map (asterisks indicate inferred links).

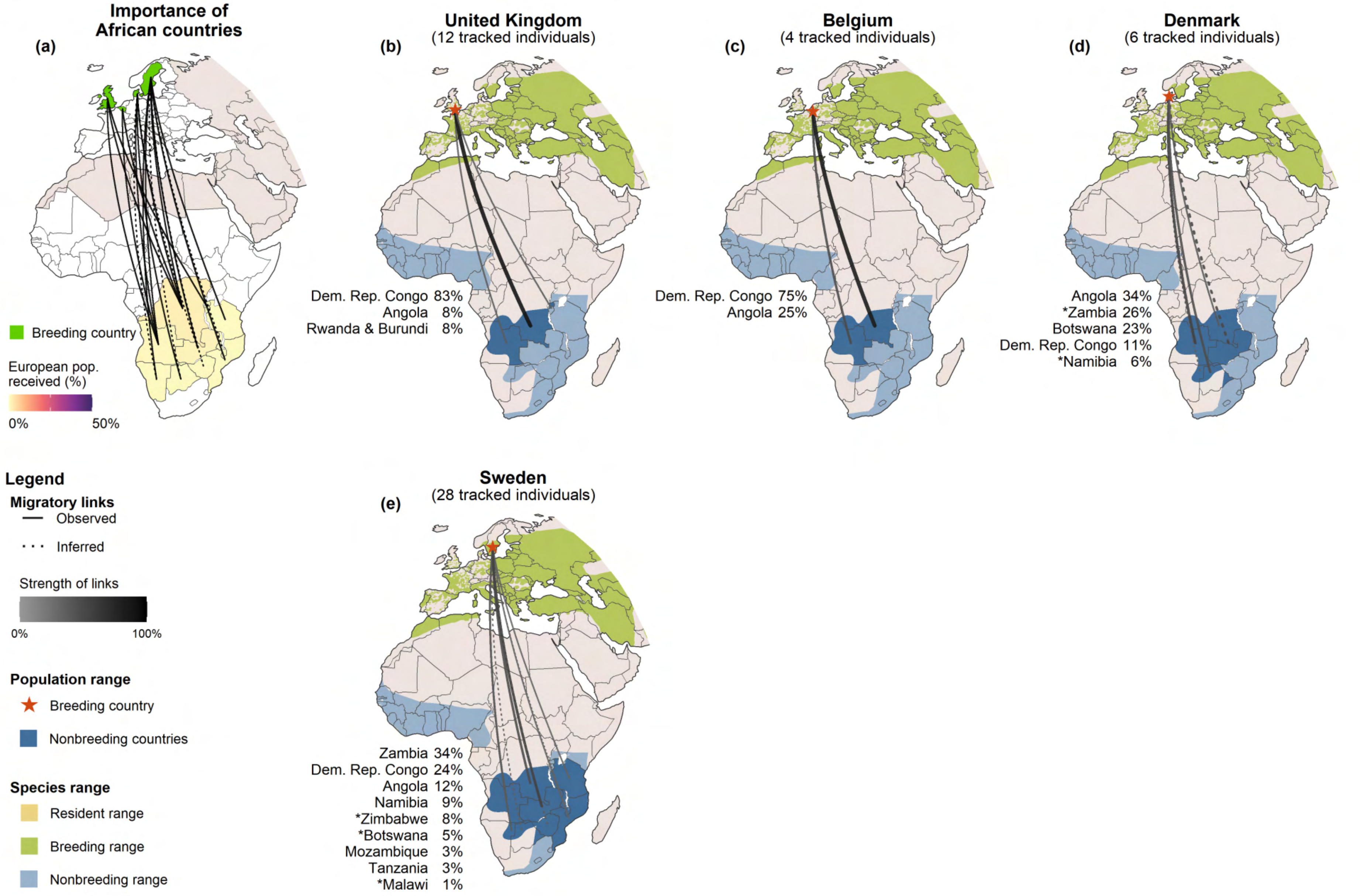

Figure S11.3: Alpine swift (*Tachymarptis melba*)

Mapping species-level connectivity for the Alpine swift. (a) Importance of each country in sub-Saharan Africa as nonbreeding grounds for the European population, as revealed by the migration links (observed: solid line, inferred: dotted line) weighted by the percentage of the European population estimated to establish the link; countries in sub-Saharan Africa are colored according to the percentage of the total European population they host during the nonbreeding season. (b - b) Connectivity for each population in our dataset; lines represent the migratory links (observed and inferred), with their respective strength indicated alongside the map (asterisks indicate inferred links).

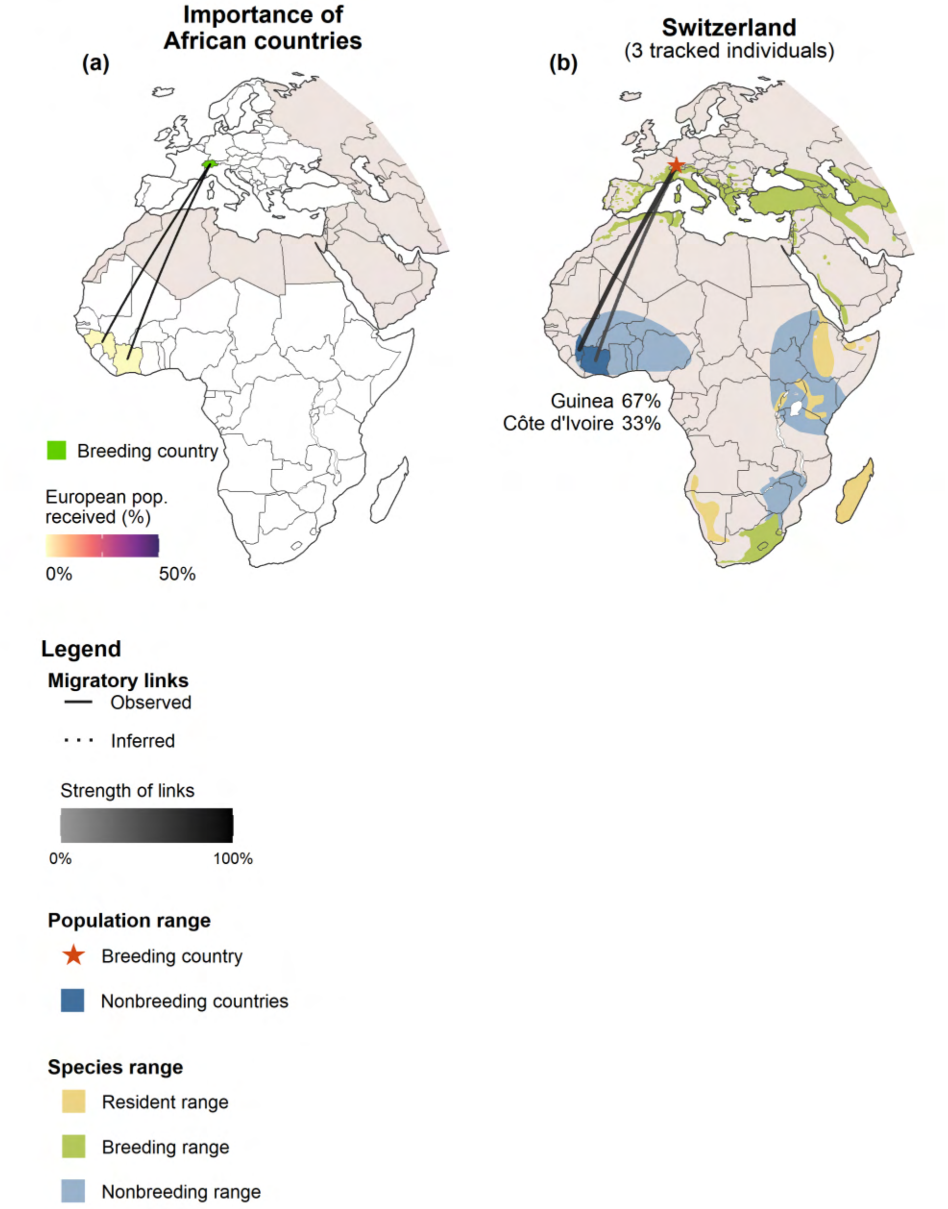

Figure S11.4: White-rumped swift (*Apus caffer*)

Mapping species-level connectivity for the white-rumped swift. (a) Importance of each country in sub-Saharan Africa as nonbreeding grounds for the European population, as revealed by the migration links (observed: solid line, inferred: dotted line) weighted by the percentage of the European population estimated to establish the link; countries in sub-Saharan Africa are colored according to the percentage of the total European population they host during the nonbreeding season. (b - b) Connectivity for each population in our dataset; lines represent the migratory links (observed and inferred), with their respective strength indicated alongside the map (asterisks indicate inferred links).

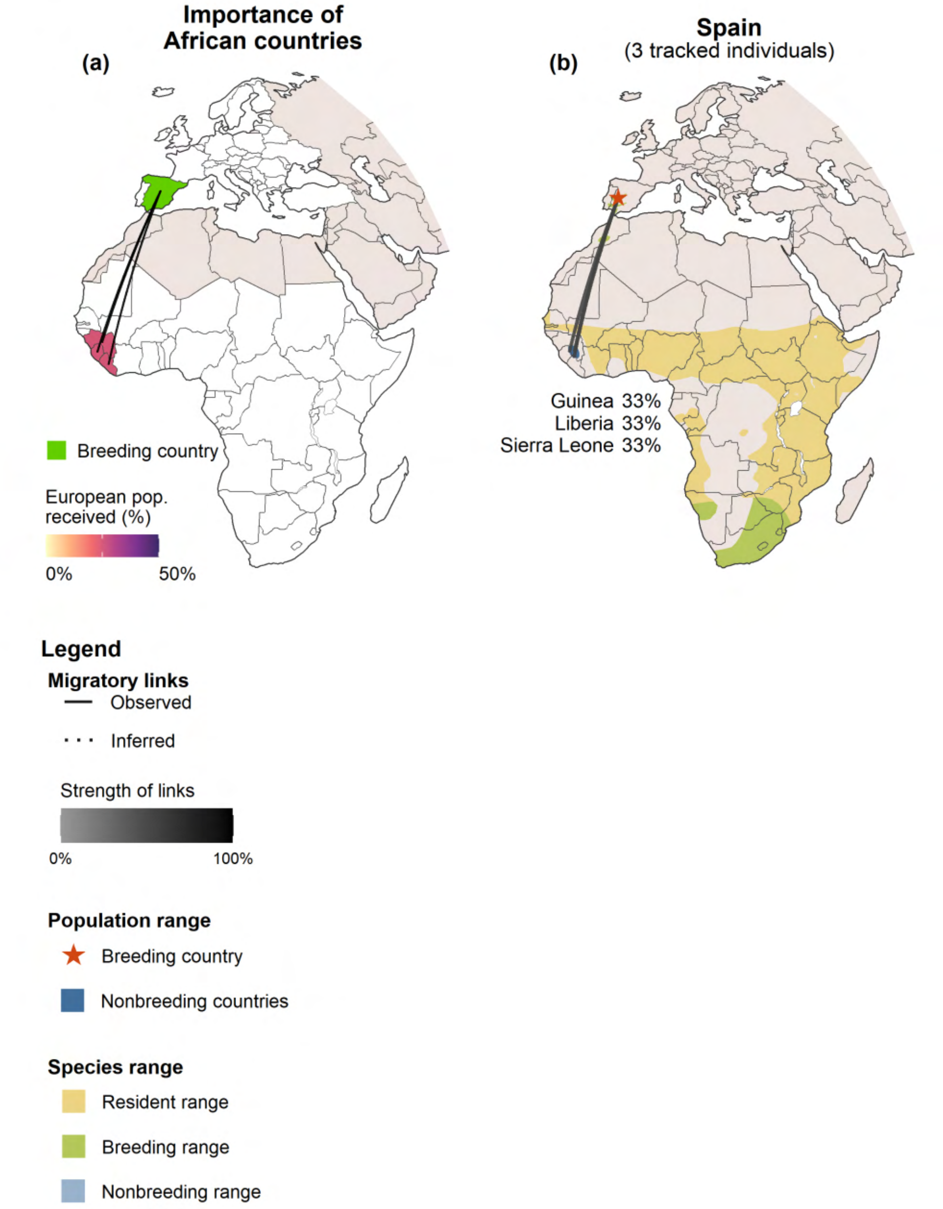

Figure S11.5: Great spotted cuckoo (*Clamator glandarius*)

Mapping species-level connectivity for the great spotted cuckoo. (a) Importance of each country in sub-Saharan Africa as nonbreeding grounds for the European population, as revealed by the migration links (observed: solid line, inferred: dotted line) weighted by the percentage of the European population estimated to establish the link; countries in sub-Saharan Africa are colored according to the percentage of the total European population they host during the nonbreeding season. (b - b) Connectivity for each population in our dataset; lines represent the migratory links (observed and inferred), with their respective strength indicated alongside the map (asterisks indicate inferred links).

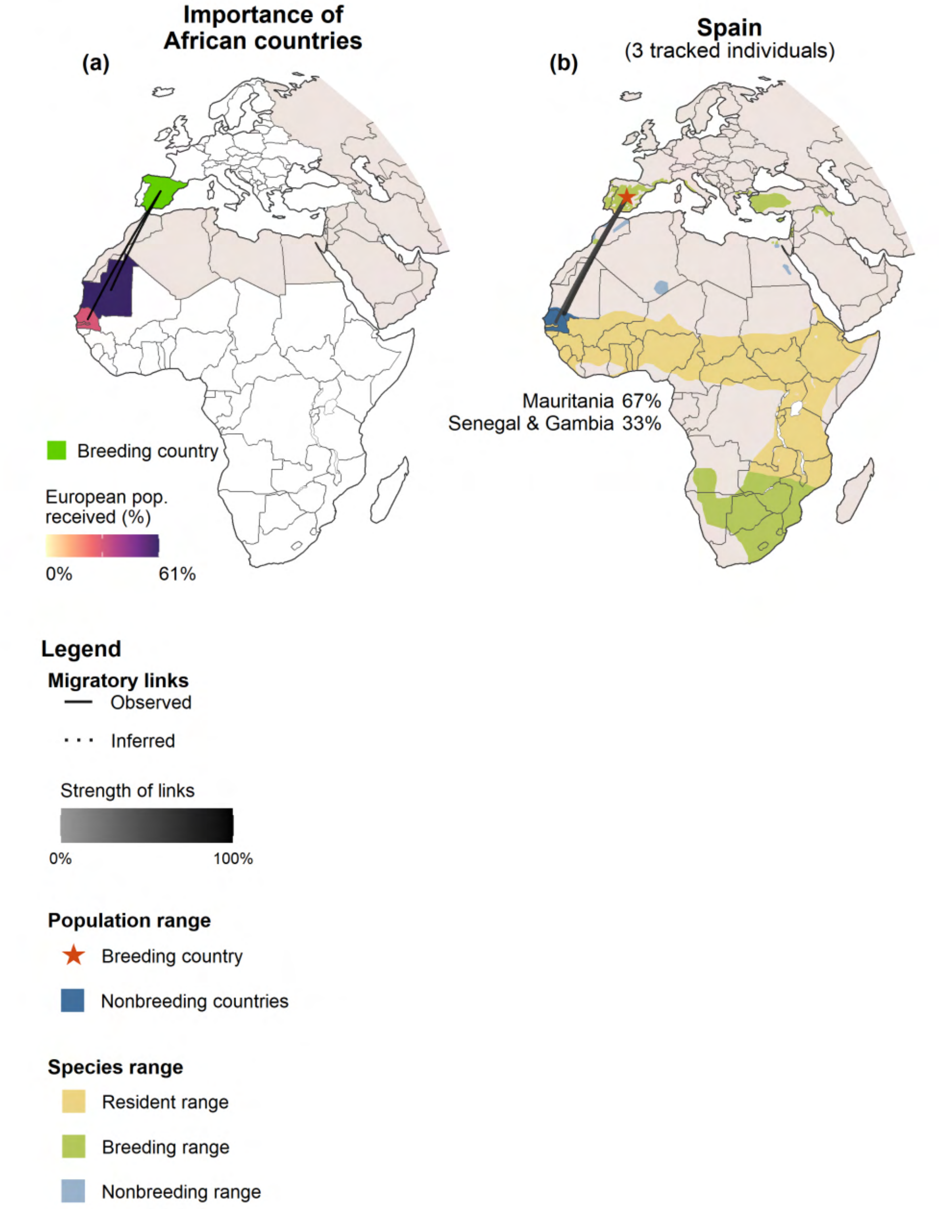

Figure S11.6: Common cuckoo (*Cuculus canorus*)

Mapping species-level connectivity for the common cuckoo. (a) Importance of each country in sub-Saharan Africa as nonbreeding grounds for the European population, as revealed by the migration links (observed: solid line, inferred: dotted line) weighted by the percentage of the European population estimated to establish the link; countries in sub-Saharan Africa are colored according to the percentage of the total European population they host during the nonbreeding season. (b - e) Connectivity for each population in our dataset; lines represent the migratory links (observed and inferred), with their respective strength indicated alongside the map (asterisks indicate inferred links).

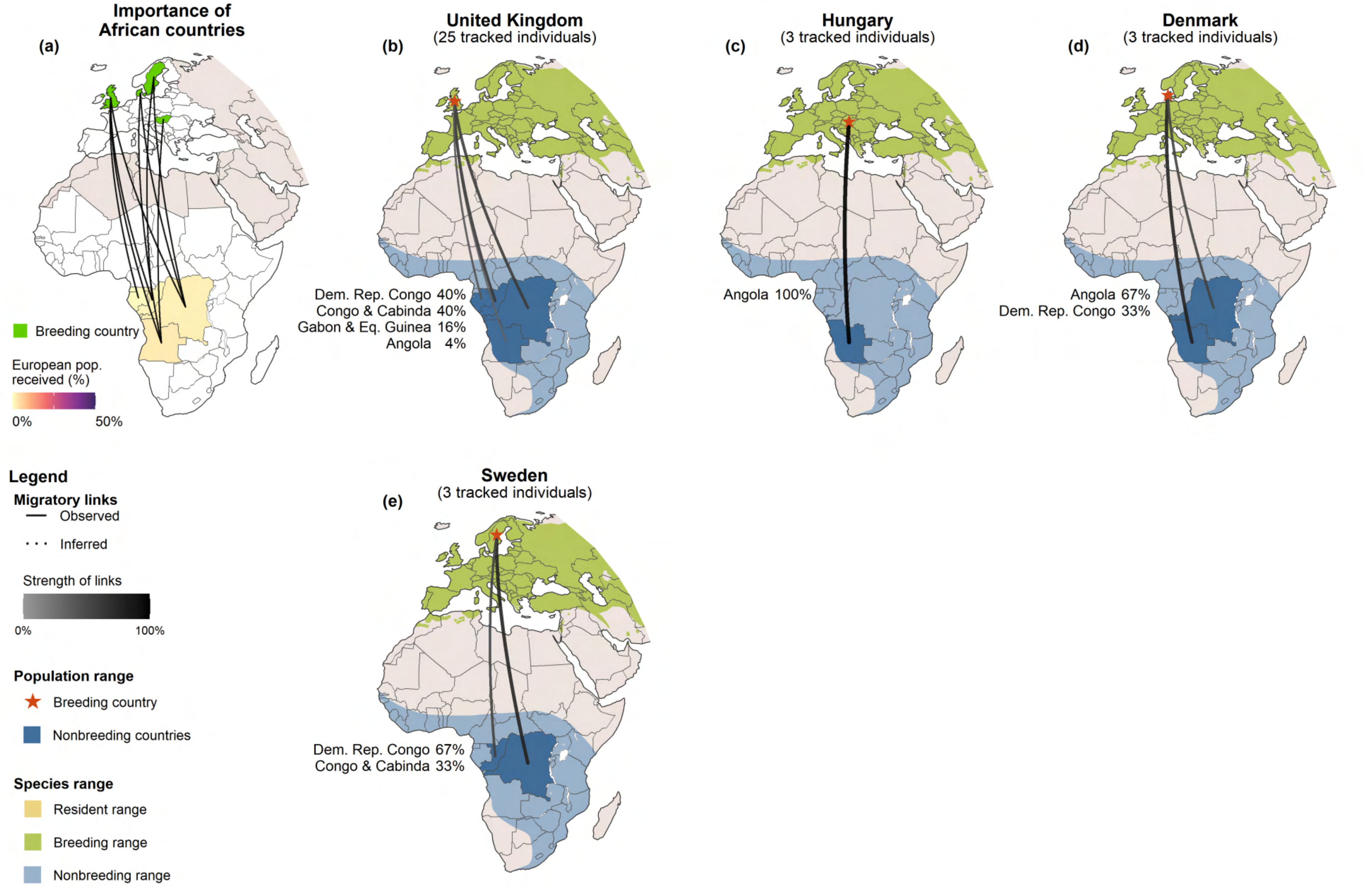

Figure S11.7: Common hoopoe (*Upupa epops*)

Mapping species-level connectivity for the common hoopoe. (a) Importance of each country in sub-Saharan Africa as nonbreeding grounds for the European population, as revealed by the migration links (observed: solid line, inferred: dotted line) weighted by the percentage of the European population estimated to establish the link; countries in sub-Saharan Africa are colored according to the percentage of the total European population they host during the nonbreeding season. (b - d) Connectivity for each population in our dataset; lines represent the migratory links (observed and inferred), with their respective strength indicated alongside the map (asterisks indicate inferred links).

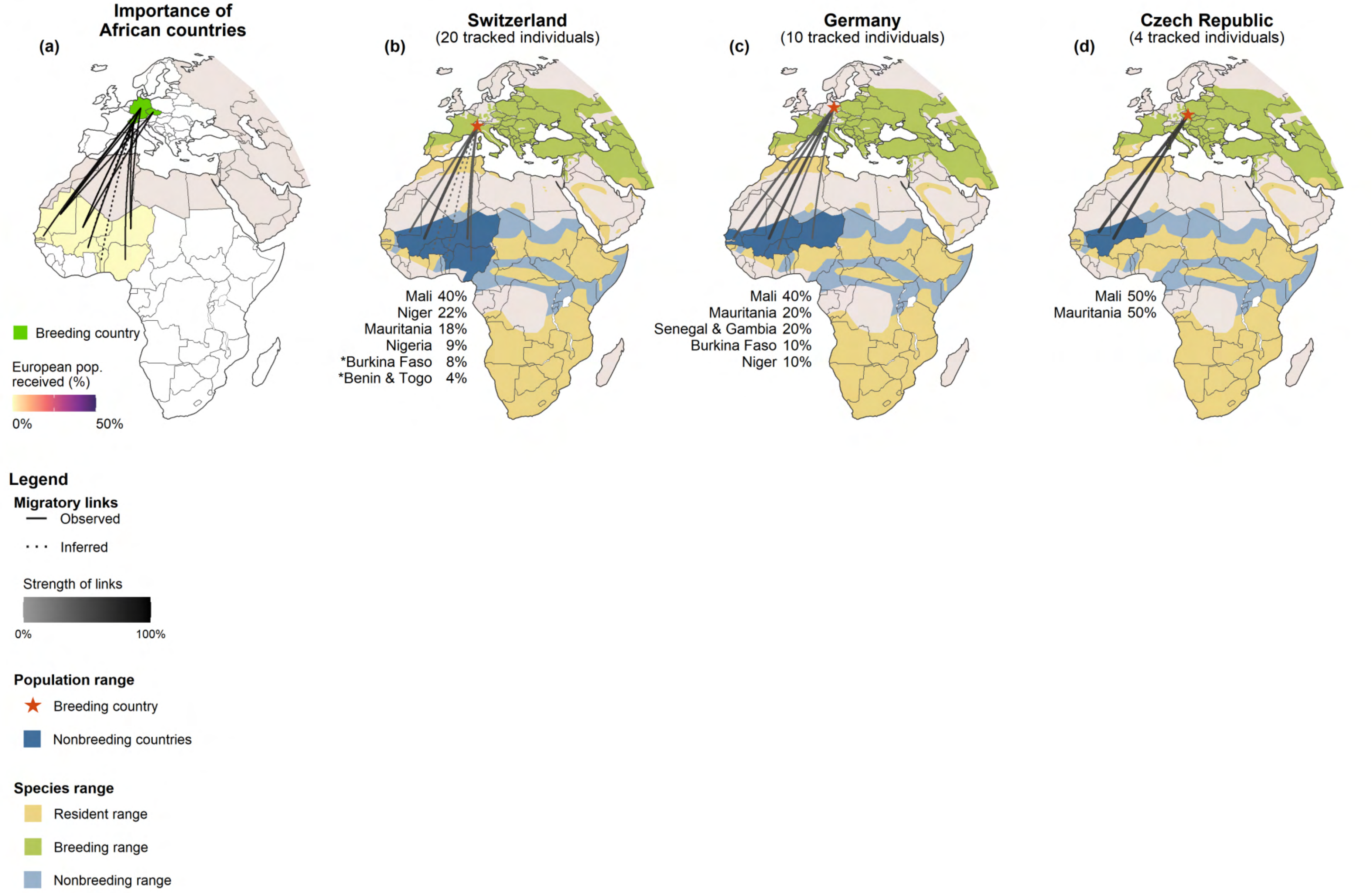

Figure S11.8: European bee-eater (*Merops apiaster*)

Mapping species-level connectivity for the European bee-eater. (a) Importance of each country in sub-Saharan Africa as nonbreeding grounds for the European population, as revealed by the migration links (observed: solid line, inferred: dotted line) weighted by the percentage of the European population estimated to establish the link; countries in sub-Saharan Africa are colored according to the percentage of the total European population they host during the nonbreeding season. (b - d) Connectivity for each population in our dataset; lines represent the migratory links (observed and inferred), with their respective strength indicated alongside the map (asterisks indicate inferred links).

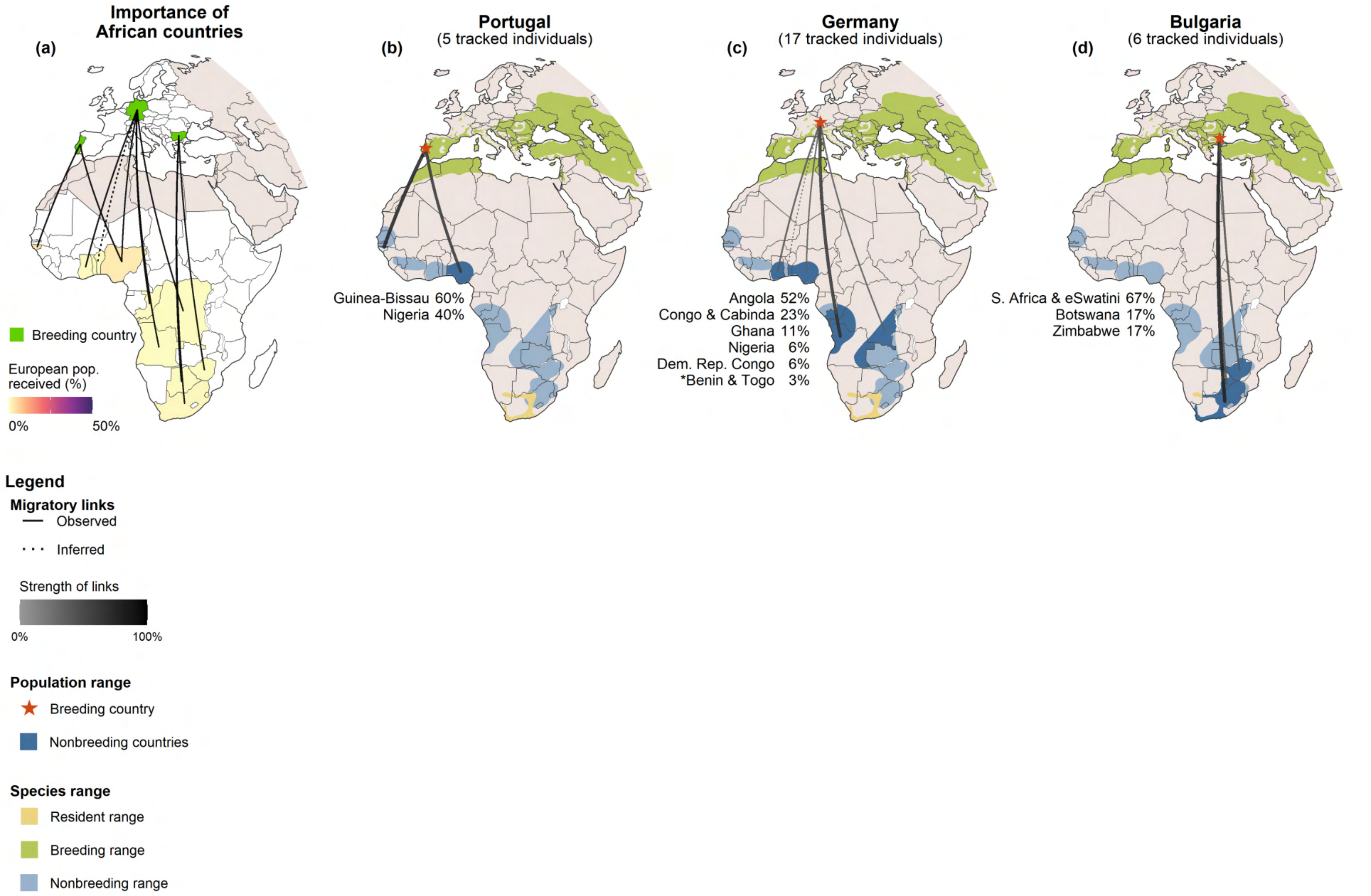

Figure S11.9: European roller (*Coracias garrulus*)

Mapping species-level connectivity for the European roller. (a) Importance of each country in sub-Saharan Africa as nonbreeding grounds for the European population, as revealed by the migration links (observed: solid line, inferred: dotted line) weighted by the percentage of the European population estimated to establish the link; countries in sub-Saharan Africa are colored according to the percentage of the total European population they host during the nonbreeding season. (b - g) Connectivity for each population in our dataset; lines represent the migratory links (observed and inferred), with their respective strength indicated alongside the map (asterisks indicate inferred links).

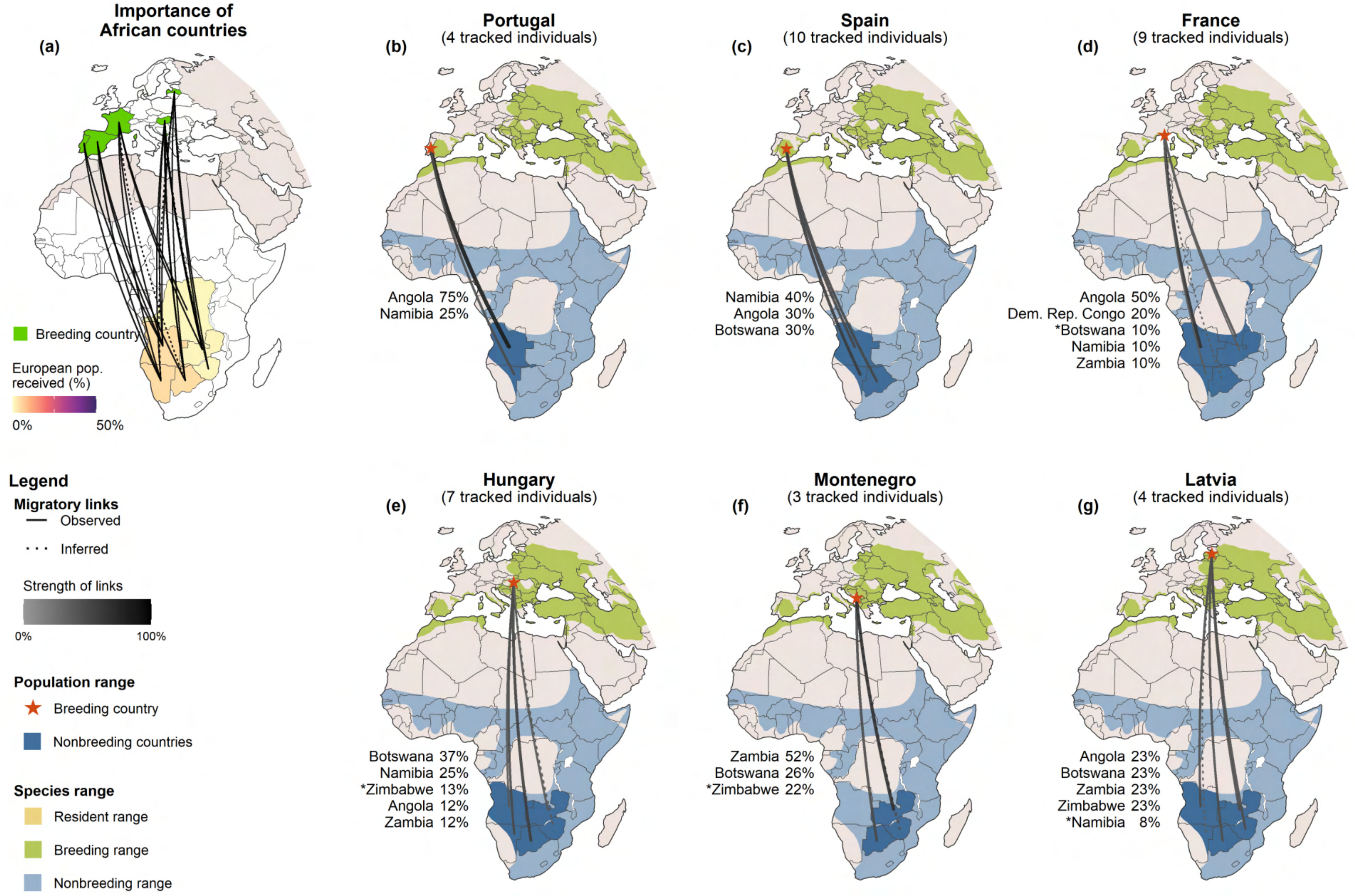

Figure S11.10: Red-backed shrike (*Lanius collurio*)

Mapping species-level connectivity for the red-backed shrike. (a) Importance of each country in sub-Saharan Africa as nonbreeding grounds for the European population, as revealed by the migration links (observed: solid line, inferred: dotted line) weighted by the percentage of the European population estimated to establish the link; countries in sub-Saharan Africa are colored according to the percentage of the total European population they host during the nonbreeding season. (b - f) Connectivity for each population in our dataset; lines represent the migratory links (observed and inferred), with their respective strength indicated alongside the map (asterisks indicate inferred links).

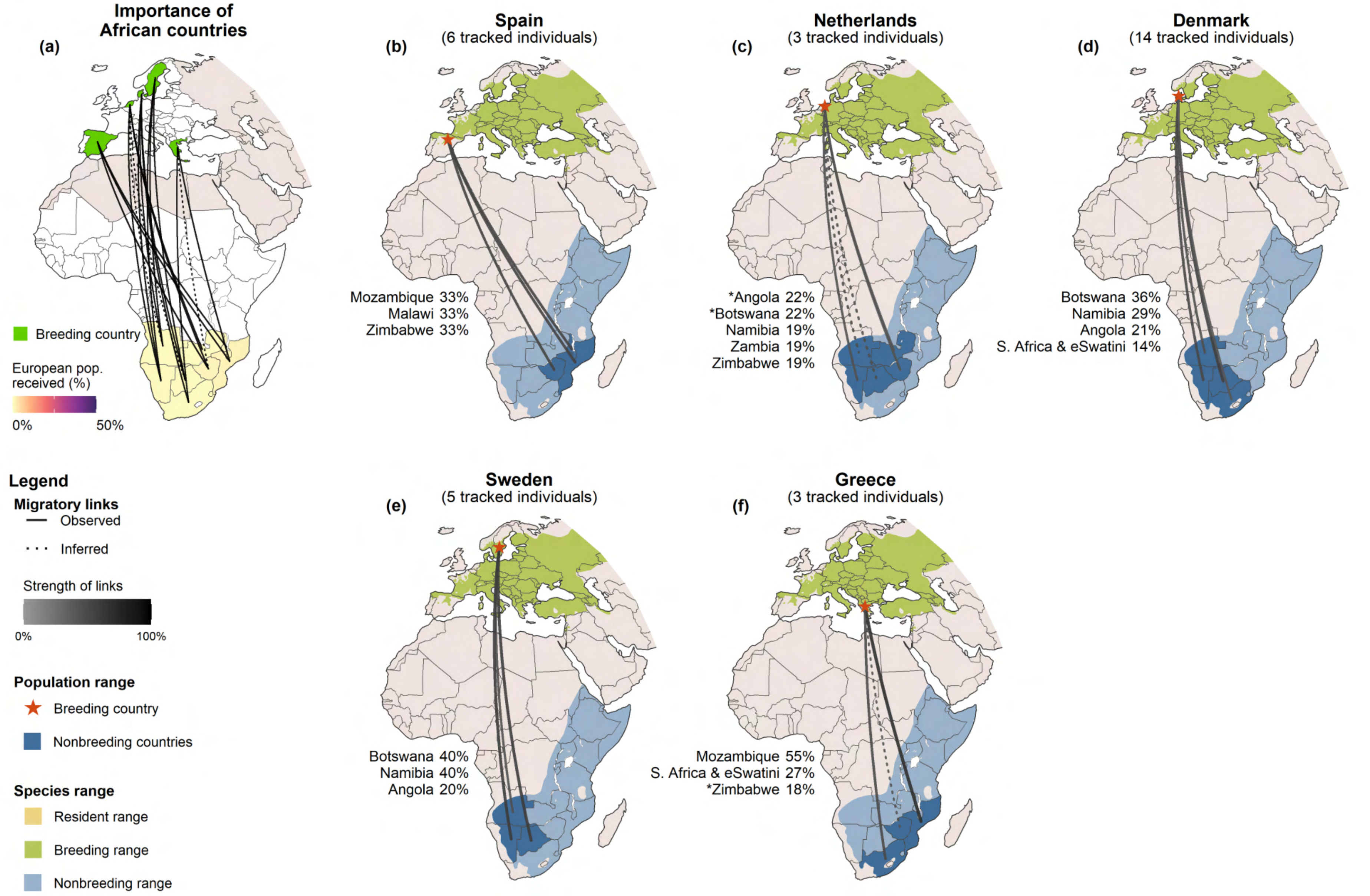

Figure S11.11: Aquatic warbler (*Acrocephalus paludicola*)

Mapping species-level connectivity for the aquatic warbler. (a) Importance of each country in sub-Saharan Africa as nonbreeding grounds for the European population, as revealed by the migration links (observed: solid line, inferred: dotted line) weighted by the percentage of the European population estimated to establish the link; countries in sub-Saharan Africa are colored according to the percentage of the total European population they host during the nonbreeding season. (b - b) Connectivity for each population in our dataset; lines represent the migratory links (observed and inferred), with their respective strength indicated alongside the map (asterisks indicate inferred links).

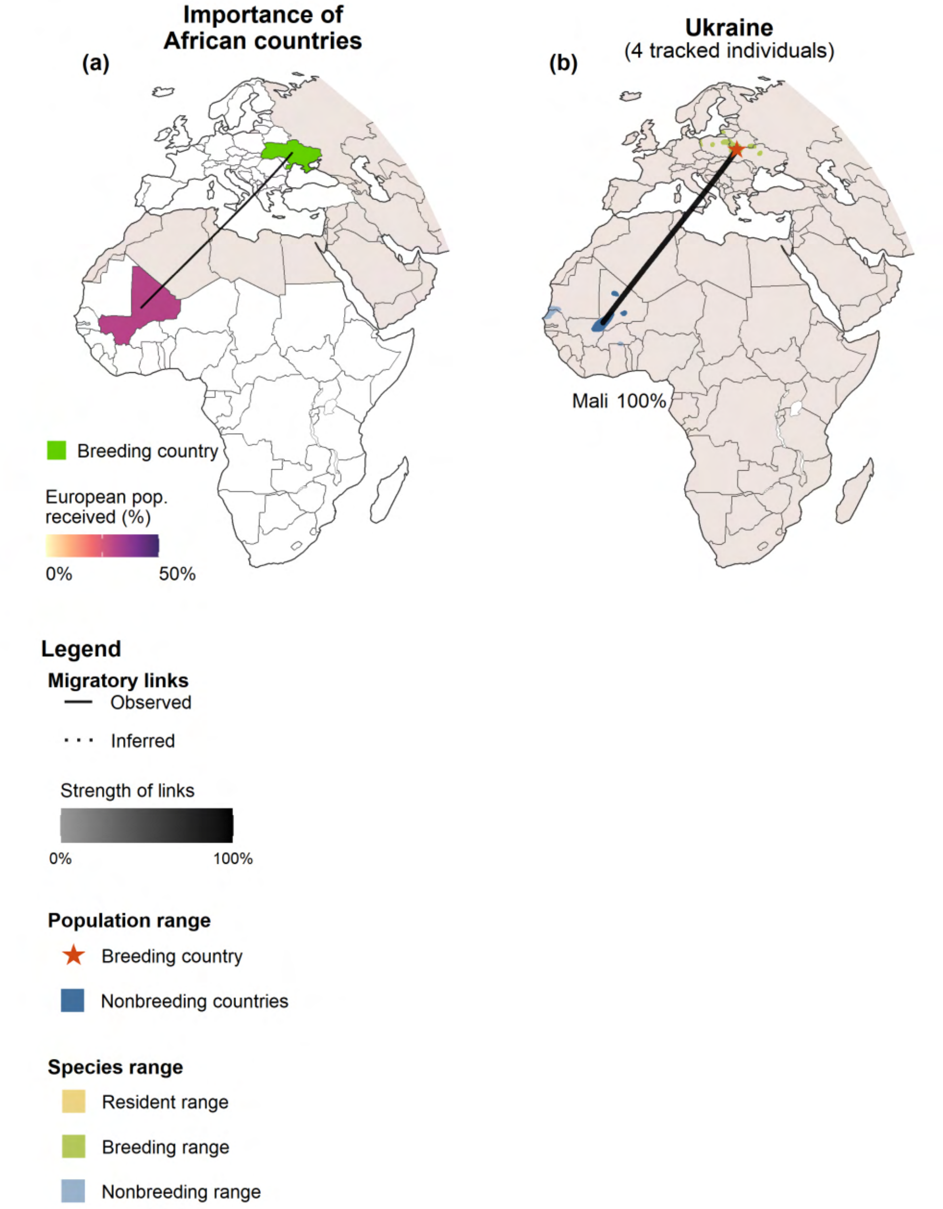

Figure S11.12: Common reed-warbler (*Acrocephalus scirpaceus*)

Mapping species-level connectivity for the common reed-warbler. (a) Importance of each country in sub-Saharan Africa as nonbreeding grounds for the European population, as revealed by the migration links (observed: solid line, inferred: dotted line) weighted by the percentage of the European population estimated to establish the link; countries in sub-Saharan Africa are colored according to the percentage of the total European population they host during the nonbreeding season. (b - c) Connectivity for each population in our dataset; lines represent the migratory links (observed and inferred), with their respective strength indicated alongside the map (asterisks indicate inferred links).

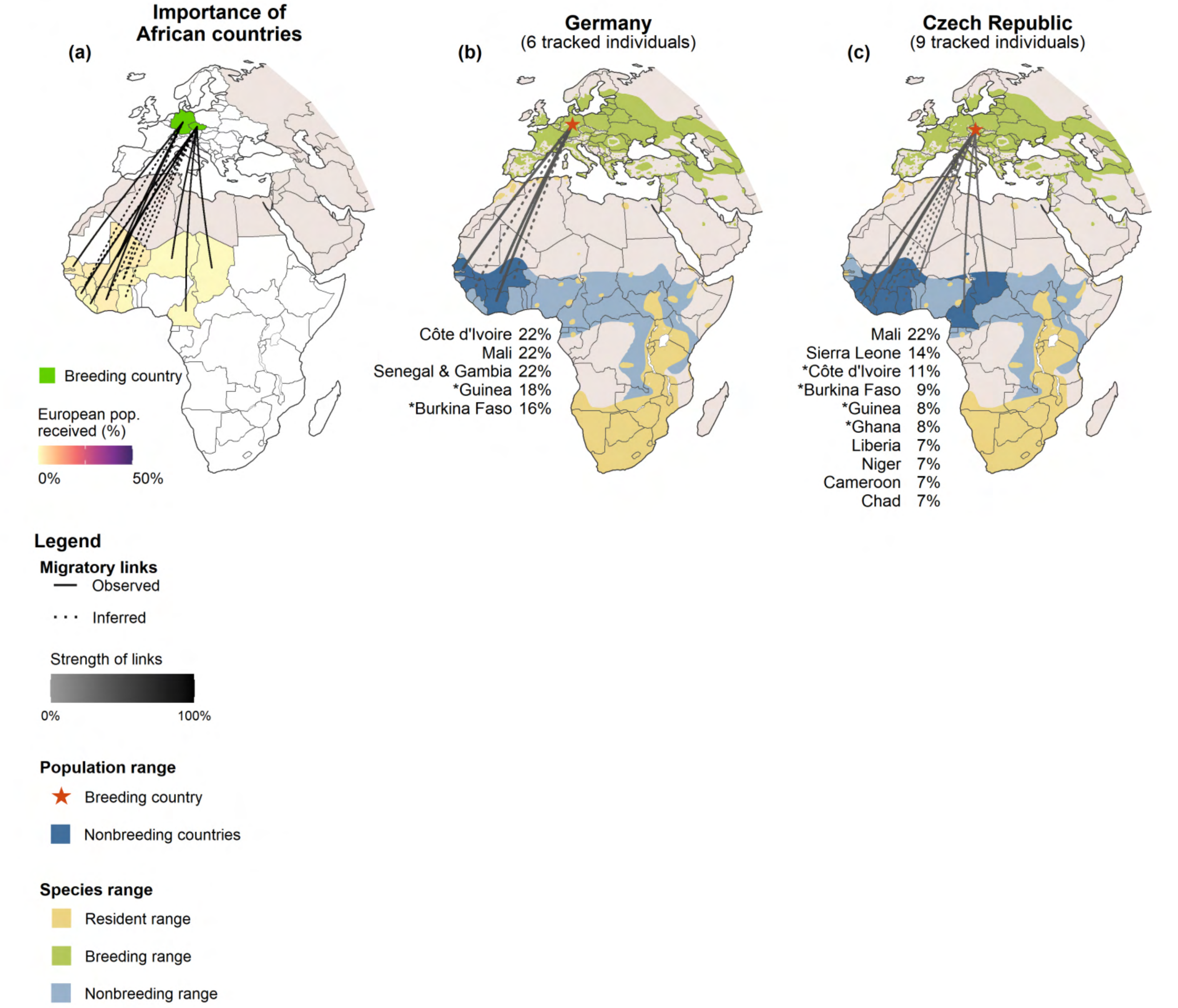

Figure S11.13: Great reed-warbler (*Acrocephalus arundinaceus*)

Mapping species-level connectivity for the great reed-warbler. (a) Importance of each country in sub-Saharan Africa as nonbreeding grounds for the European population, as revealed by the migration links (observed: solid line, inferred: dotted line) weighted by the percentage of the European population estimated to establish the link; countries in sub-Saharan Africa are colored according to the percentage of the total European population they host during the nonbreeding season. (b - f) Connectivity for each population in our dataset; lines represent the migratory links (observed and inferred), with their respective strength indicated alongside the map (asterisks indicate inferred links).

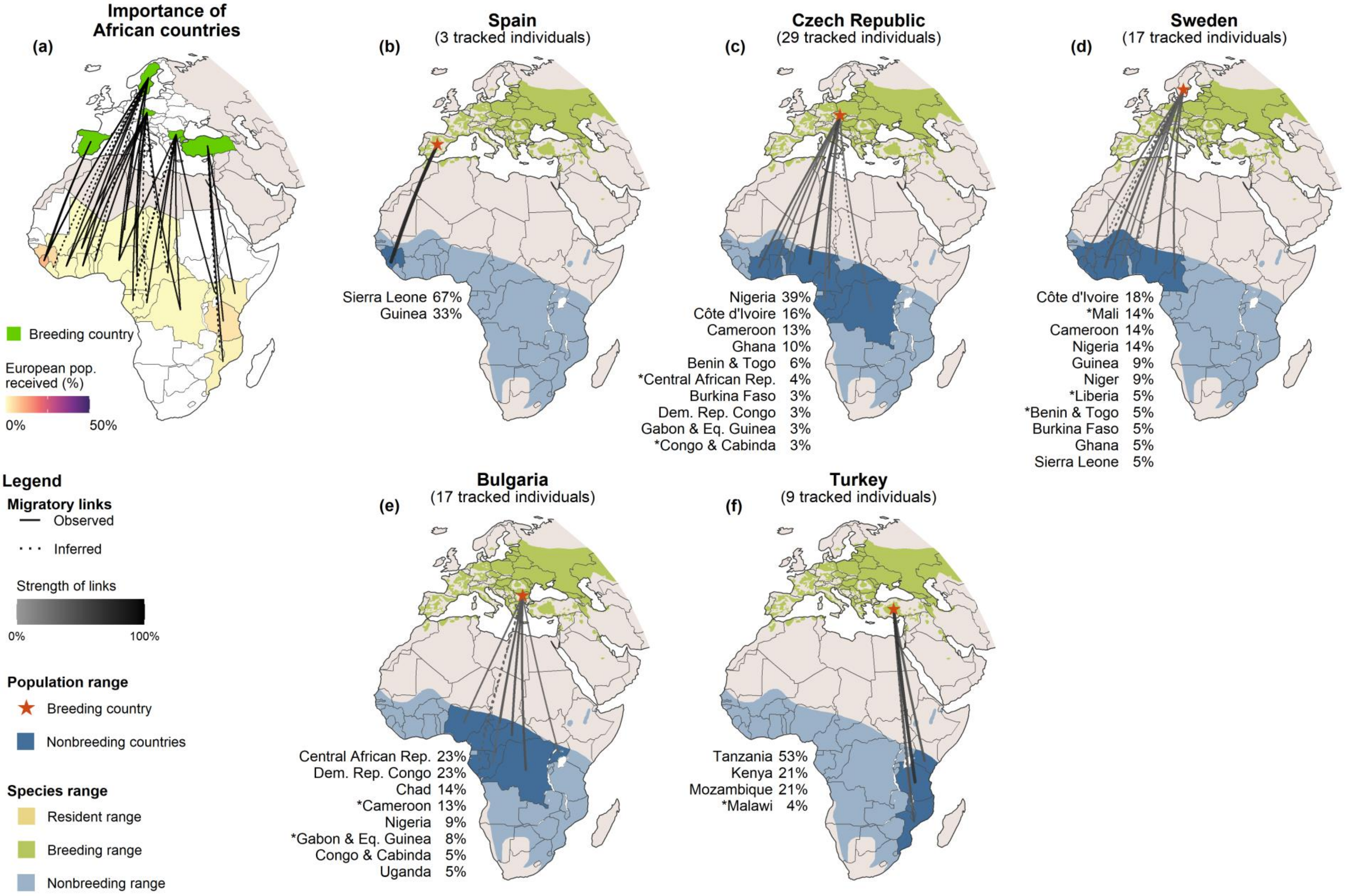

Figure S11.14: Northern house martin (*Delichon urbicum*)

Mapping species-level connectivity for the Northern house martin. (a) Importance of each country in sub-Saharan Africa as nonbreeding grounds for the European population, as revealed by the migration links (observed: solid line, inferred: dotted line) weighted by the percentage of the European population estimated to establish the link; countries in sub-Saharan Africa are colored according to the percentage of the total European population they host during the nonbreeding season. (b - b) Connectivity for each population in our dataset; lines represent the migratory links (observed and inferred), with their respective strength indicated alongside the map (asterisks indicate inferred links).

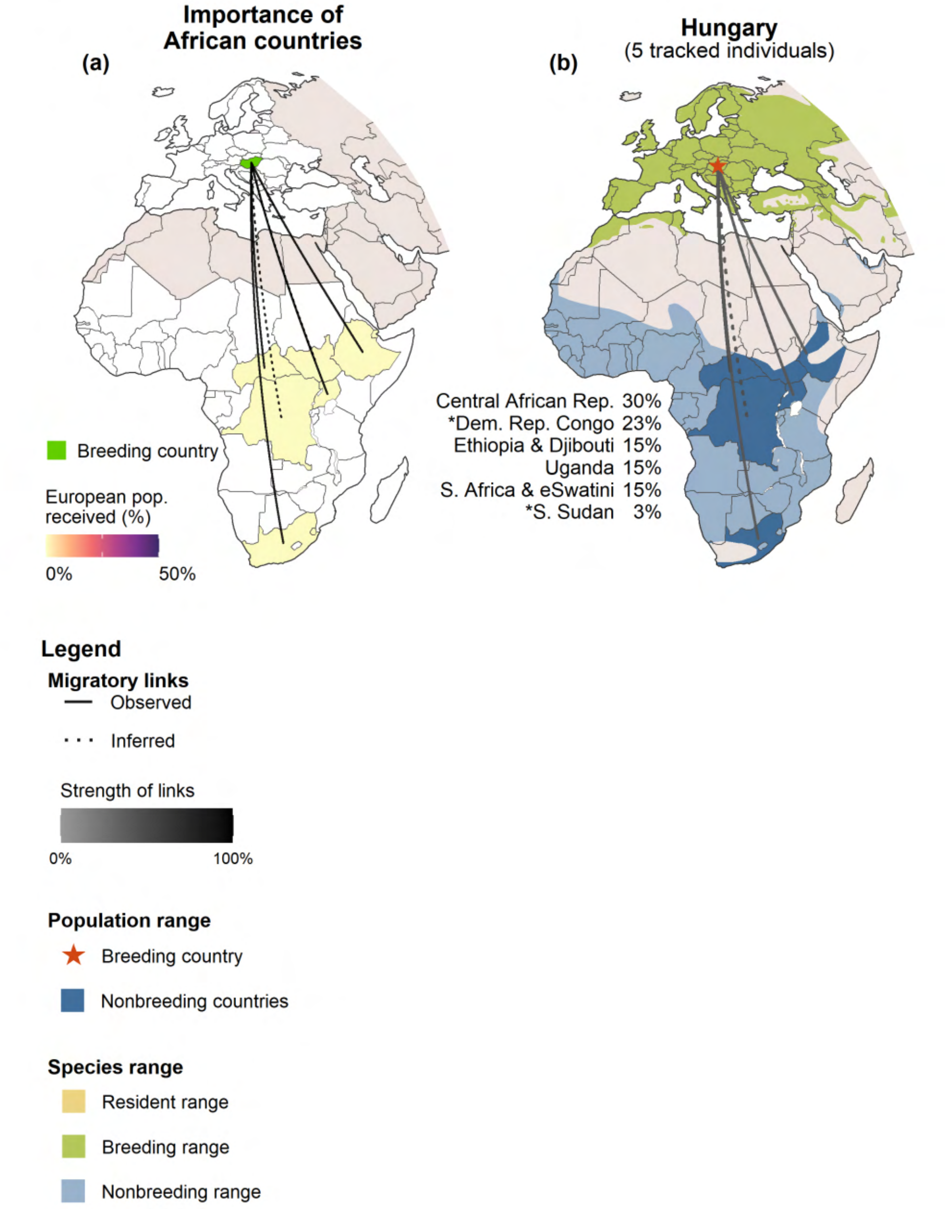

Figure S11.15: Barn swallow (*Hirundo rustica*)

Mapping species-level connectivity for the barn swallow. (a) Importance of each country in sub-Saharan Africa as nonbreeding grounds for the European population, as revealed by the migration links (observed: solid line, inferred: dotted line) weighted by the percentage of the European population estimated to establish the link; countries in sub-Saharan Africa are colored according to the percentage of the total European population they host during the nonbreeding season. (b - f) Connectivity for each population in our dataset; lines represent the migratory links (observed and inferred), with their respective strength indicated alongside the map (asterisks indicate inferred links).

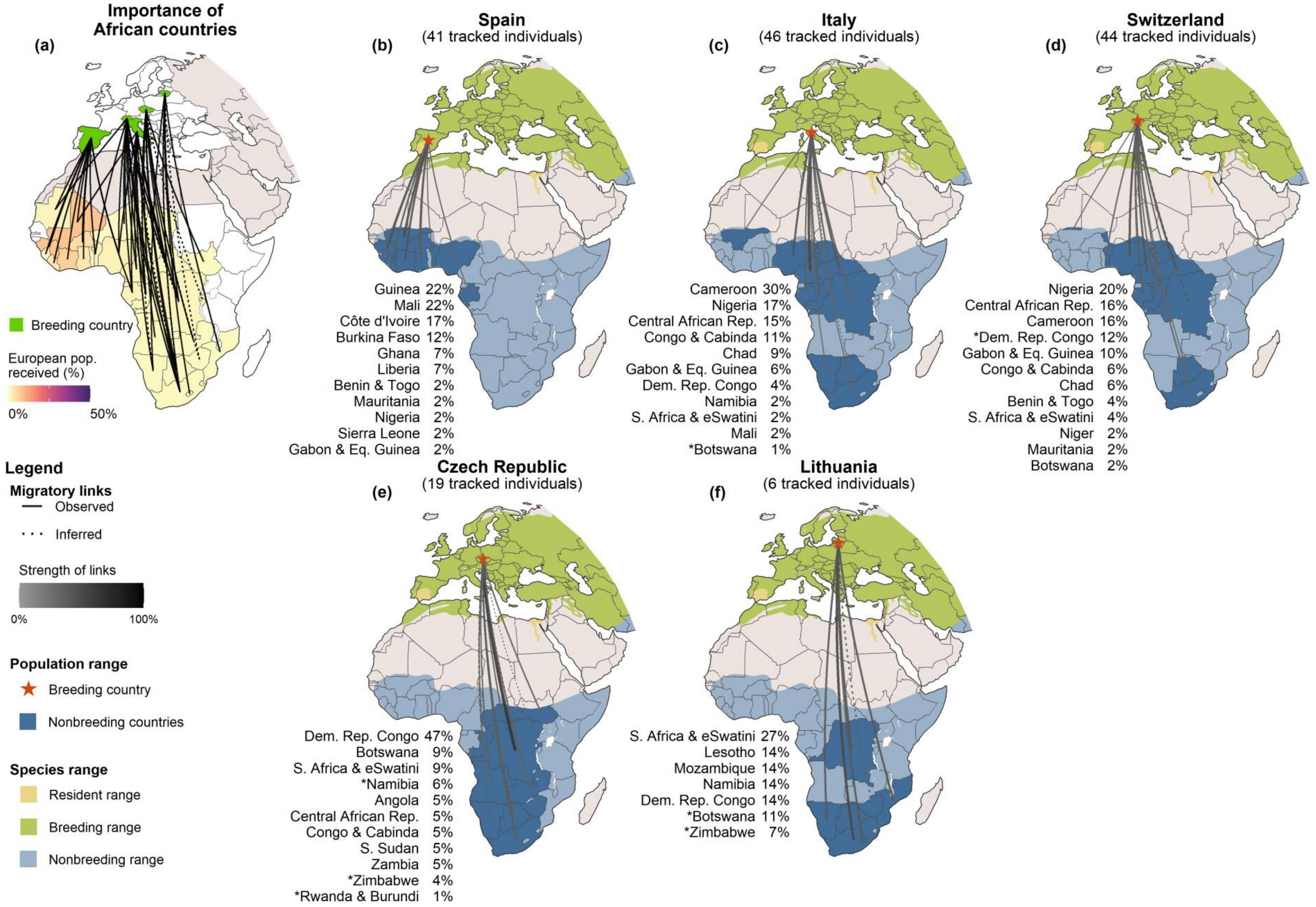

Figure S11.16: Collared sand martin (*Riparia riparia*)

Mapping species-level connectivity for the collared sand martin. (a) Importance of each country in sub-Saharan Africa as nonbreeding grounds for the European population, as revealed by the migration links (observed: solid line, inferred: dotted line) weighted by the percentage of the European population estimated to establish the link; countries in sub-Saharan Africa are colored according to the percentage of the total European population they host during the nonbreeding season. (b - b) Connectivity for each population in our dataset; lines represent the migratory links (observed and inferred), with their respective strength indicated alongside the map (asterisks indicate inferred links).

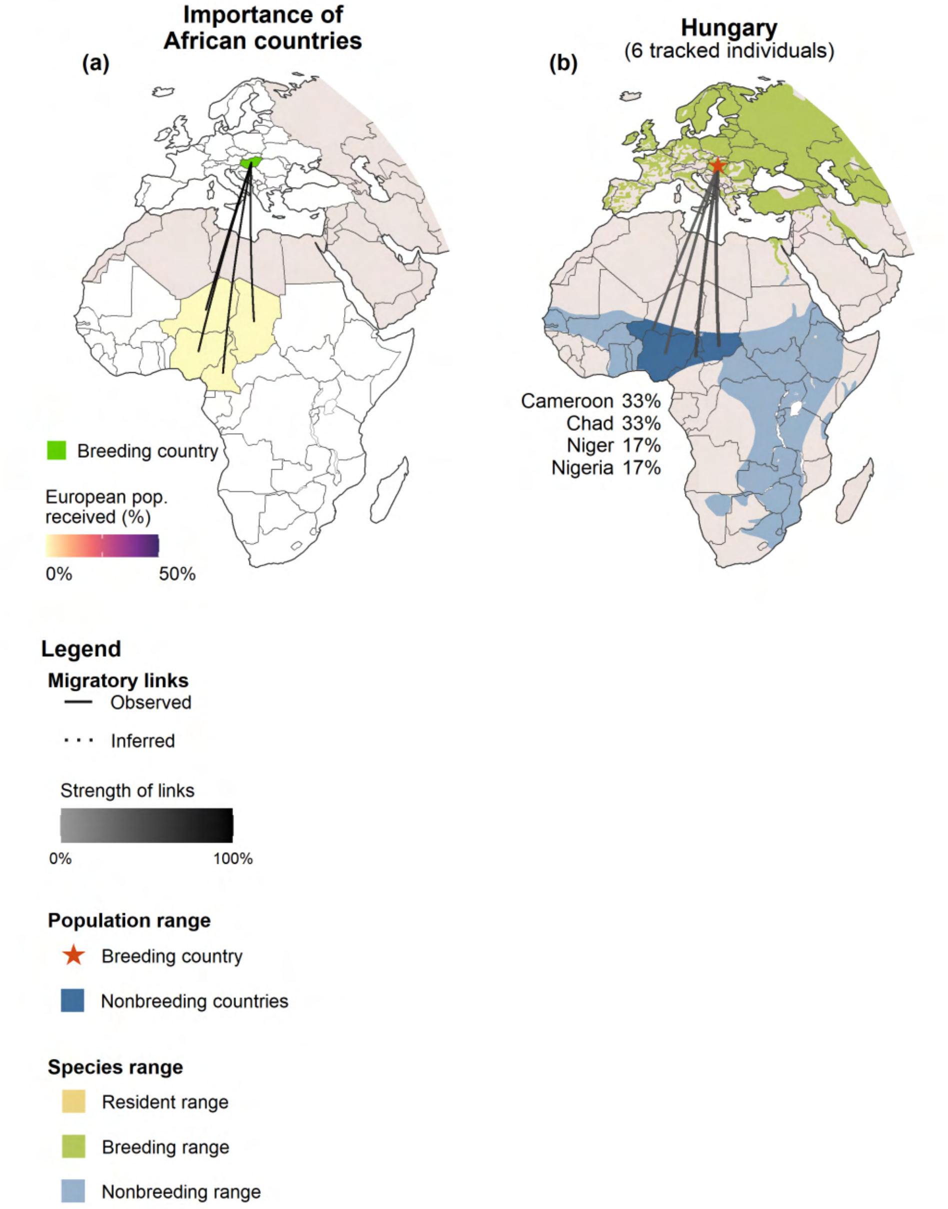

Figure S11.17: Willow warbler (*Phylloscopus trochilus*)

Mapping species-level connectivity for the willow warbler. (a) Importance of each country in sub-Saharan Africa as nonbreeding grounds for the European population, as revealed by the migration links (observed: solid line, inferred: dotted line) weighted by the percentage of the European population estimated to establish the link; countries in sub-Saharan Africa are colored according to the percentage of the total European population they host during the nonbreeding season. (b - b) Connectivity for each population in our dataset; lines represent the migratory links (observed and inferred), with their respective strength indicated alongside the map (asterisks indicate inferred links).

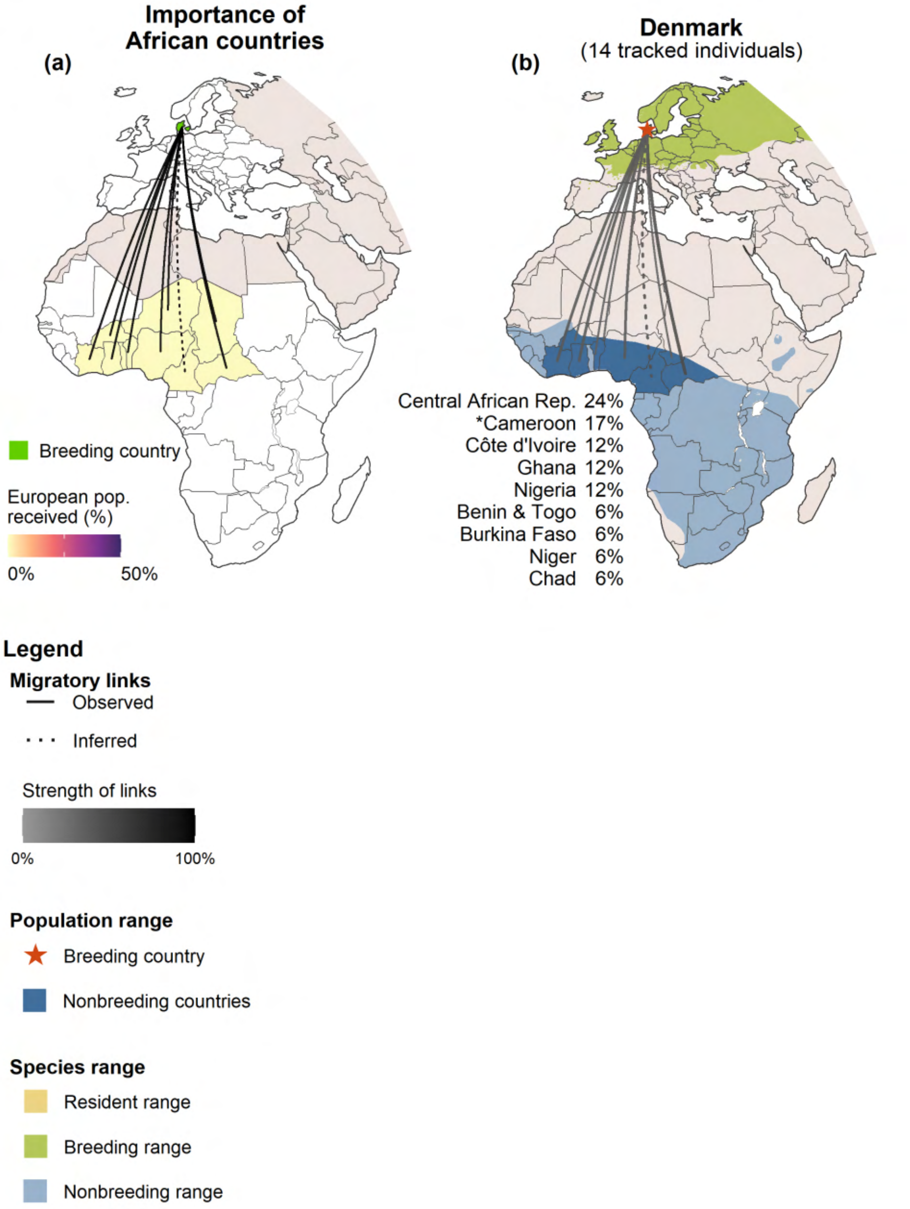

Figure S11.18: Rufous-tailed scrub-robin (*Cercotrichas galactotes*)

Mapping species-level connectivity for the rufous-tailed scrub-robin. (a) Importance of each country in sub-Saharan Africa as nonbreeding grounds for the European population, as revealed by the migration links (observed: solid line, inferred: dotted line) weighted by the percentage of the European population estimated to establish the link; countries in sub-Saharan Africa are colored according to the percentage of the total European population they host during the nonbreeding season. (b - b) Connectivity for each population in our dataset; lines represent the migratory links (observed and inferred), with their respective strength indicated alongside the map (asterisks indicate inferred links).

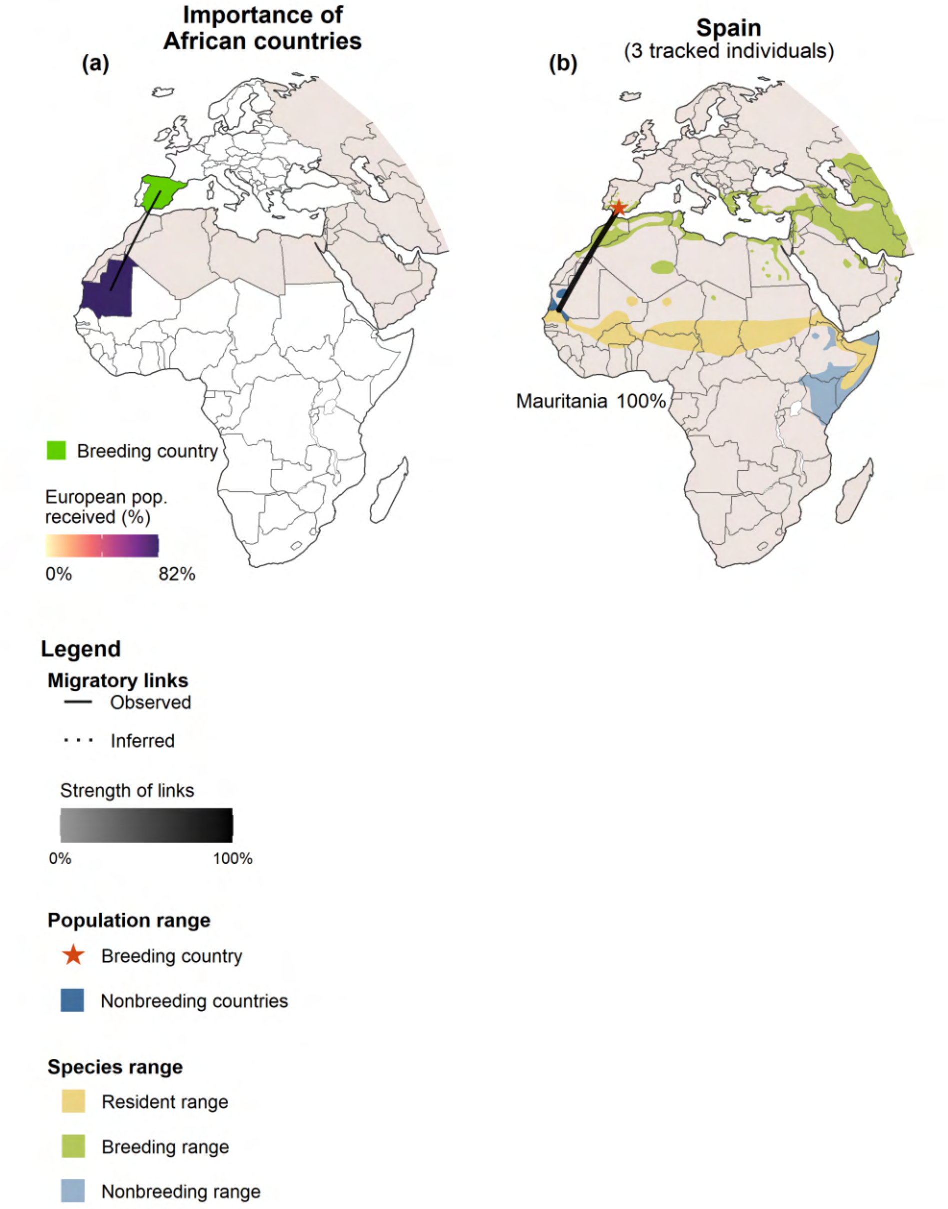

Figure S11.19: Thrush nightingale (*Luscinia luscinia*)

Mapping species-level connectivity for the thrush nightingale. (a) Importance of each country in sub-Saharan Africa as nonbreeding grounds for the European population, as revealed by the migration links (observed: solid line, inferred: dotted line) weighted by the percentage of the European population estimated to establish the link; countries in sub-Saharan Africa are colored according to the percentage of the total European population they host during the nonbreeding season. (b - b) Connectivity for each population in our dataset; lines represent the migratory links (observed and inferred), with their respective strength indicated alongside the map (asterisks indicate inferred links).

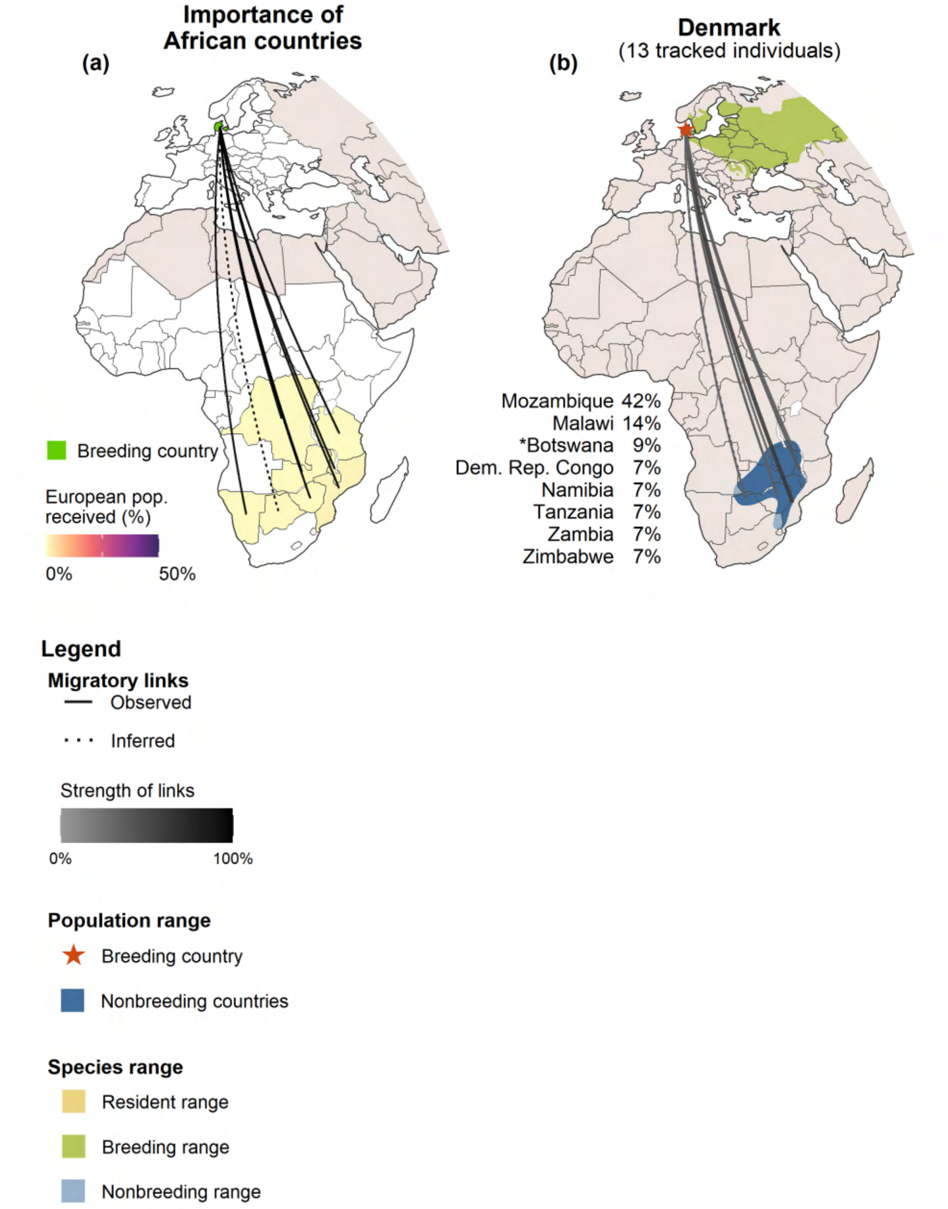

Figure S11.20: Common nightingale (*Luscinia megarhynchos*)

Mapping species-level connectivity for the common nightingale. (a) Importance of each country in sub-Saharan Africa as nonbreeding grounds for the European population, as revealed by the migration links (observed: solid line, inferred: dotted line) weighted by the percentage of the European population estimated to establish the link; countries in sub-Saharan Africa are colored according to the percentage of the total European population they host during the nonbreeding season. (b - d) Connectivity for each population in our dataset; lines represent the migratory links (observed and inferred), with their respective strength indicated alongside the map (asterisks indicate inferred links).

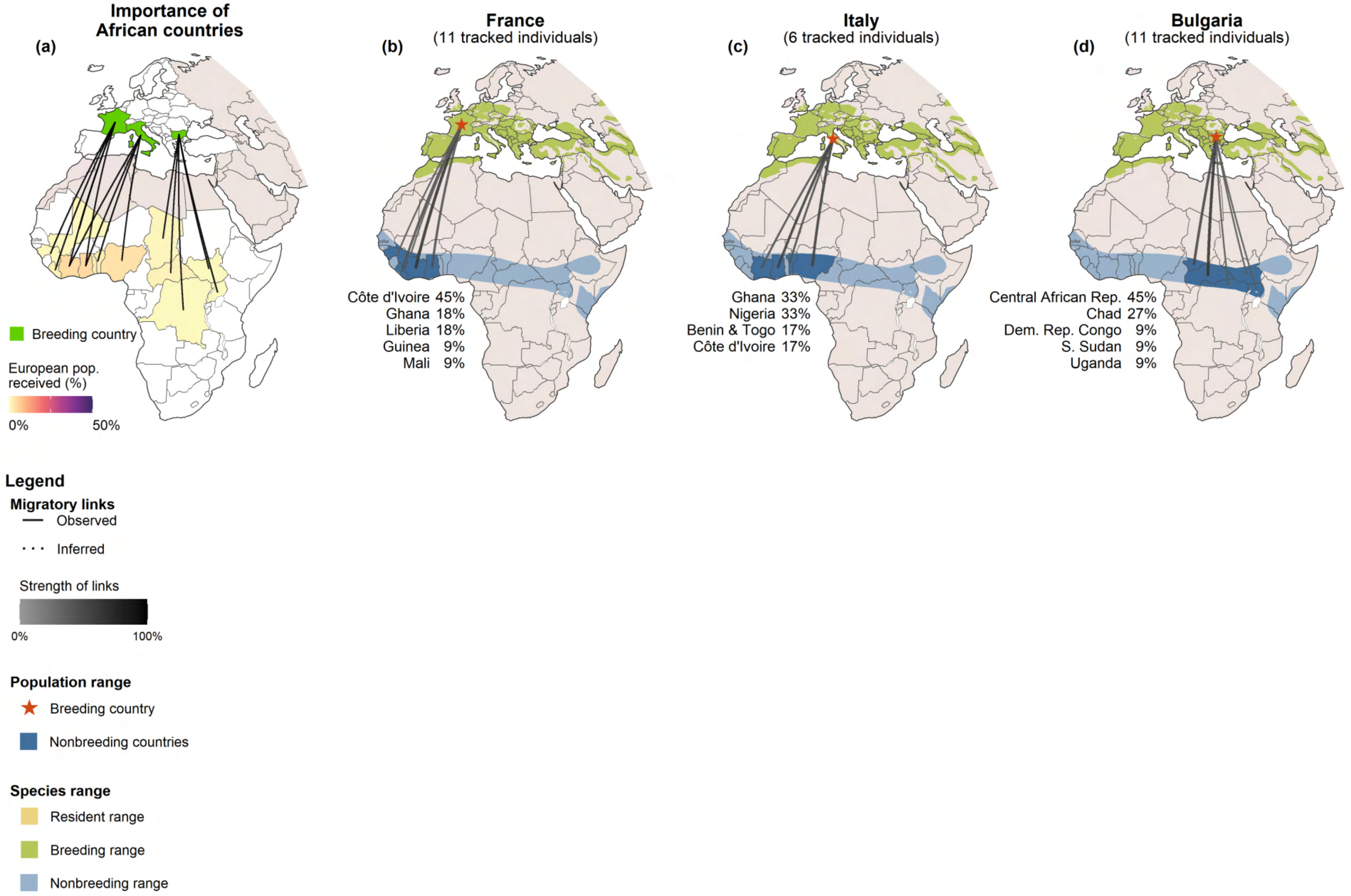

Figure S11.21: Semi-collared flycatcher (*Ficedula semitorquata*)

Mapping species-level connectivity for the semi-collared flycatcher. (a) Importance of each country in sub-Saharan Africa as nonbreeding grounds for the European population, as revealed by the migration links (observed: solid line, inferred: dotted line) weighted by the percentage of the European population estimated to establish the link; countries in sub-Saharan Africa are colored according to the percentage of the total European population they host during the nonbreeding season. (b - b) Connectivity for each population in our dataset; lines represent the migratory links (observed and inferred), with their respective strength indicated alongside the map (asterisks indicate inferred links).

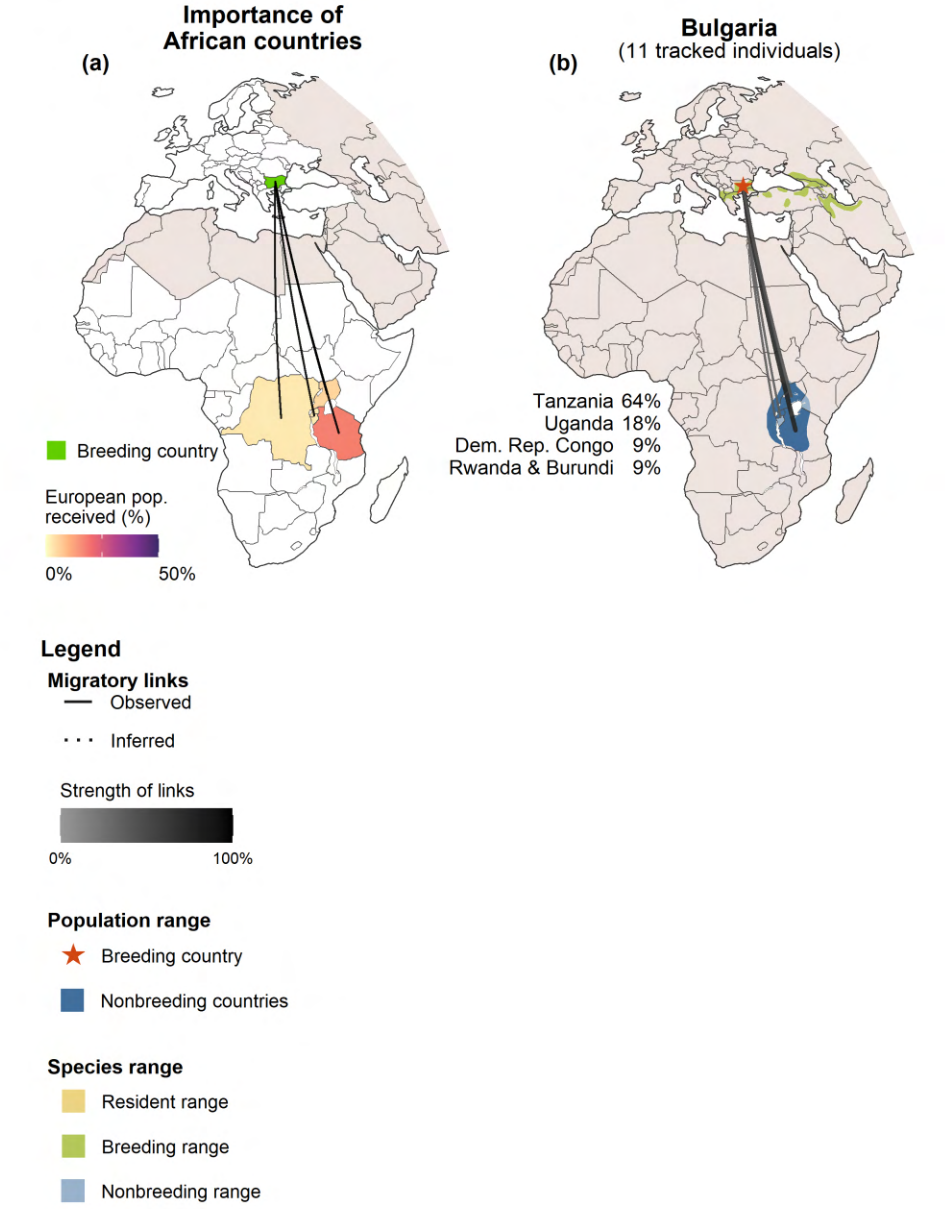

Figure S11.22: European pied flycatcher (*Ficedula hypoleuca*)

Mapping species-level connectivity for the European pied flycatcher. (a) Importance of each country in sub-Saharan Africa as nonbreeding grounds for the European population, as revealed by the migration links (observed: solid line, inferred: dotted line) weighted by the percentage of the European population estimated to establish the link; countries in sub-Saharan Africa are colored according to the percentage of the total European population they host during the nonbreeding season. (b - c) Connectivity for each population in our dataset; lines represent the migratory links (observed and inferred), with their respective strength indicated alongside the map (asterisks indicate inferred links).

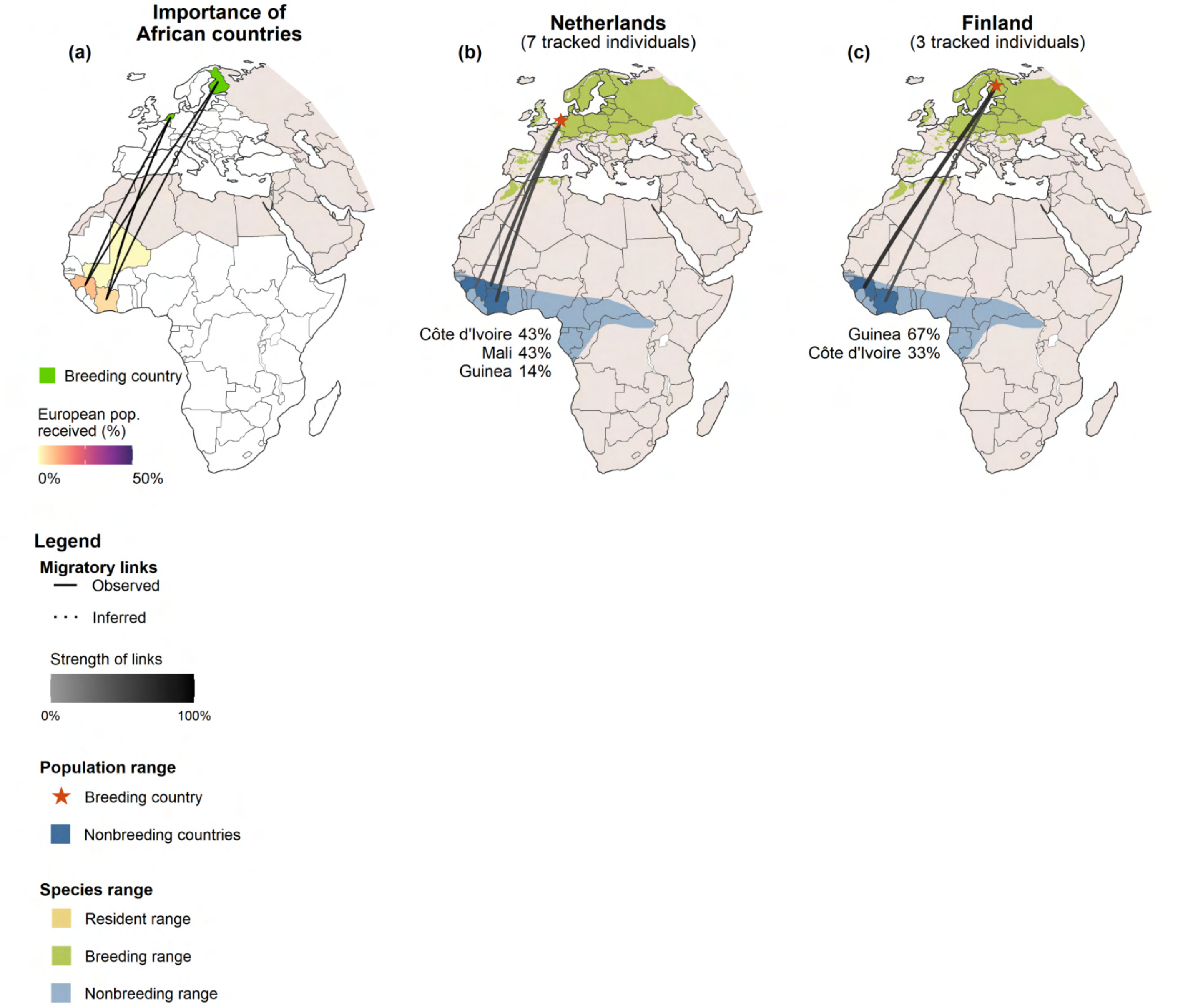

Figure S11.23: Collared flycatcher (*Ficedula albicollis*)

Mapping species-level connectivity for the collared flycatcher. (a) Importance of each country in sub-Saharan Africa as nonbreeding grounds for the European population, as revealed by the migration links (observed: solid line, inferred: dotted line) weighted by the percentage of the European population estimated to establish the link; countries in sub-Saharan Africa are colored according to the percentage of the total European population they host during the nonbreeding season. (b - c) Connectivity for each population in our dataset; lines represent the migratory links (observed and inferred), with their respective strength indicated alongside the map (asterisks indicate inferred links).

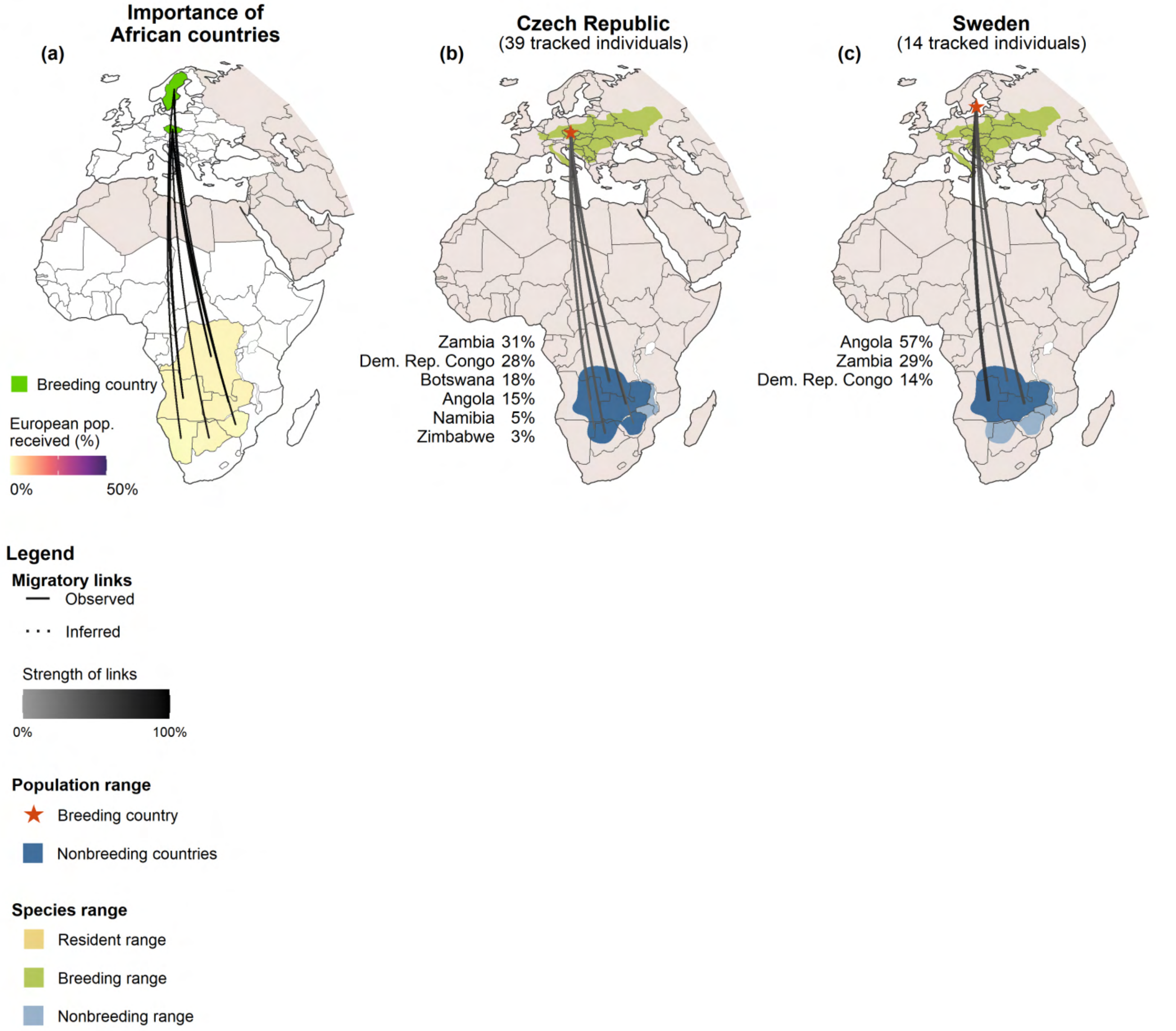

Figure S11.24: Common redstart (*Phoenicurus phoenicurus*)

Mapping species-level connectivity for the common redstart. (a) Importance of each country in sub-Saharan Africa as nonbreeding grounds for the European population, as revealed by the migration links (observed: solid line, inferred: dotted line) weighted by the percentage of the European population estimated to establish the link; countries in sub-Saharan Africa are colored according to the percentage of the total European population they host during the nonbreeding season. (b - c) Connectivity for each population in our dataset; lines represent the migratory links (observed and inferred), with their respective strength indicated alongside the map (asterisks indicate inferred links).

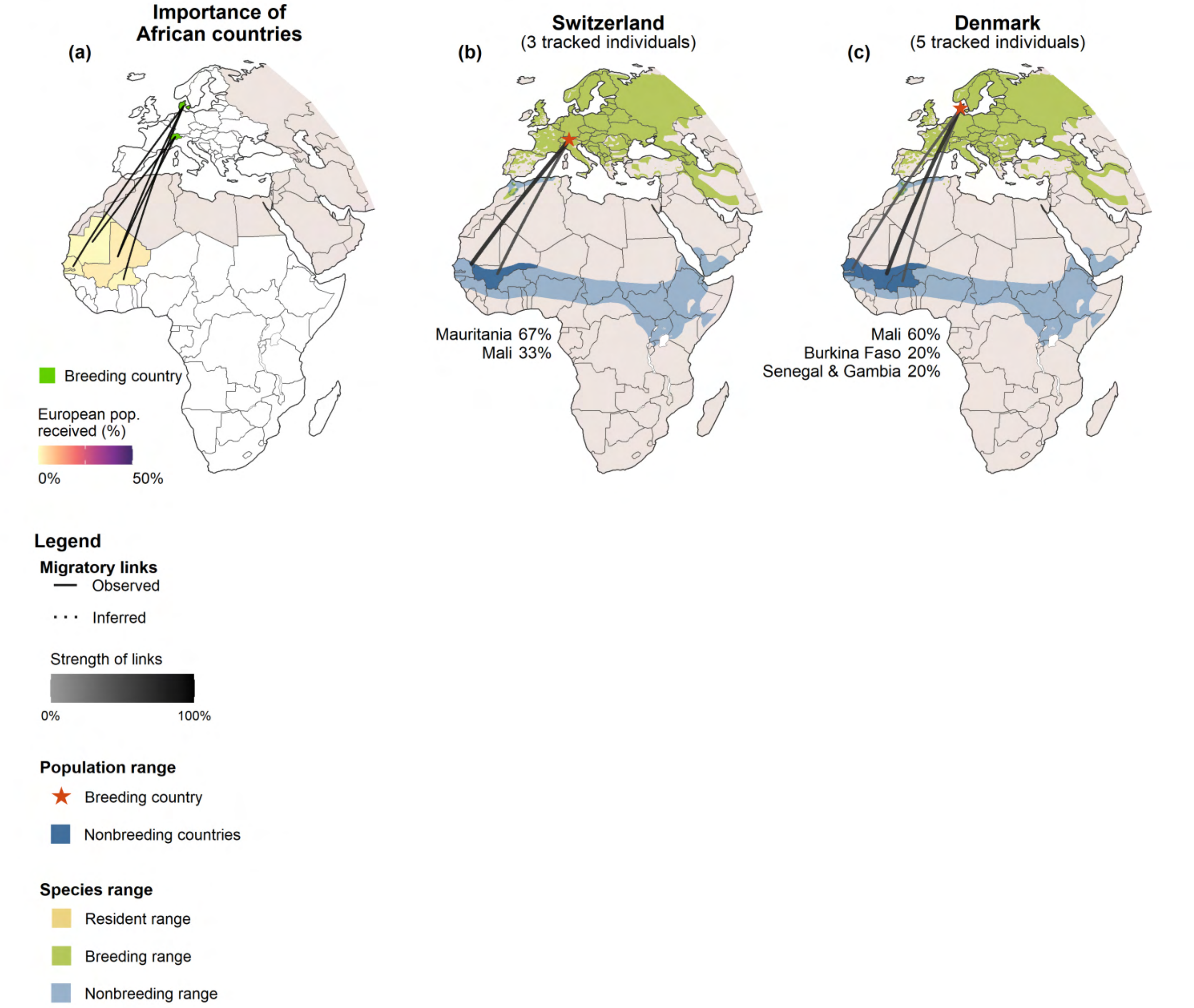

Figure S11.25: Whinchat (*Saxicola rubetra*)

Mapping species-level connectivity for the whinchat. (a) Importance of each country in sub-Saharan Africa as nonbreeding grounds for the European population, as revealed by the migration links (observed: solid line, inferred: dotted line) weighted by the percentage of the European population estimated to establish the link; countries in sub-Saharan Africa are colored according to the percentage of the total European population they host during the nonbreeding season. (b - b) Connectivity for each population in our dataset; lines represent the migratory links (observed and inferred), with their respective strength indicated alongside the map (asterisks indicate inferred links).

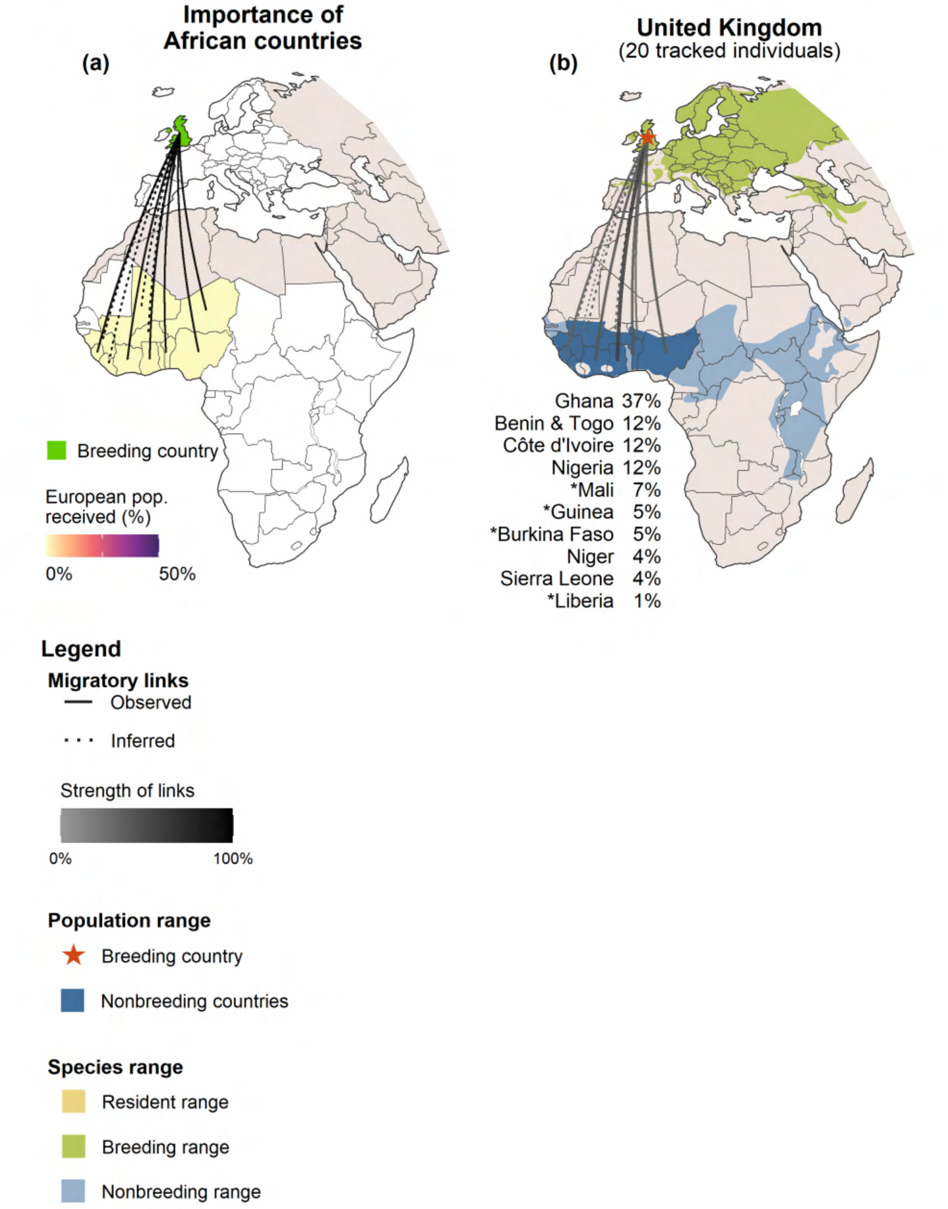

Figure S11.26: Northern wheatear (*Oenanthe oenanthe*)

Mapping species-level connectivity for the Northern wheatear. (a) Importance of each country in sub-Saharan Africa as nonbreeding grounds for the European population, as revealed by the migration links (observed: solid line, inferred: dotted line) weighted by the percentage of the European population estimated to establish the link; countries in sub-Saharan Africa are colored according to the percentage of the total European population they host during the nonbreeding season. (b - d) Connectivity for each population in our dataset; lines represent the migratory links (observed and inferred), with their respective strength indicated alongside the map (asterisks indicate inferred links).

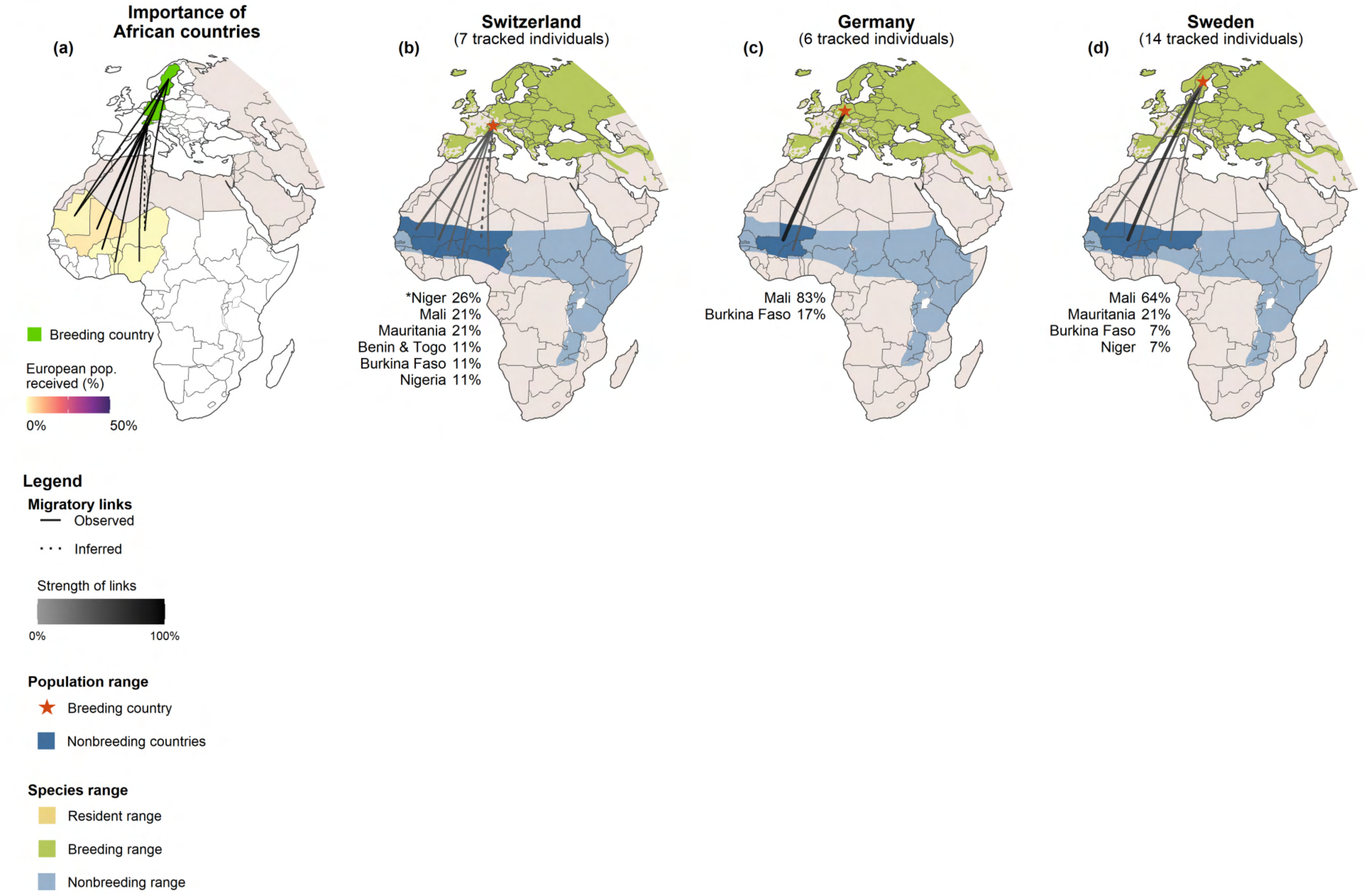

Figure S11.27: Cyprus wheatear (*Oenanthe cypriaca*)

Mapping species-level connectivity for the Cyprus wheatear. (a) Importance of each country in sub-Saharan Africa as nonbreeding grounds for the European population, as revealed by the migration links (observed: solid line, inferred: dotted line) weighted by the percentage of the European population estimated to establish the link; countries in sub-Saharan Africa are colored according to the percentage of the total European population they host during the nonbreeding season. (b - b) Connectivity for each population in our dataset; lines represent the migratory links (observed and inferred), with their respective strength indicated alongside the map (asterisks indicate inferred links).

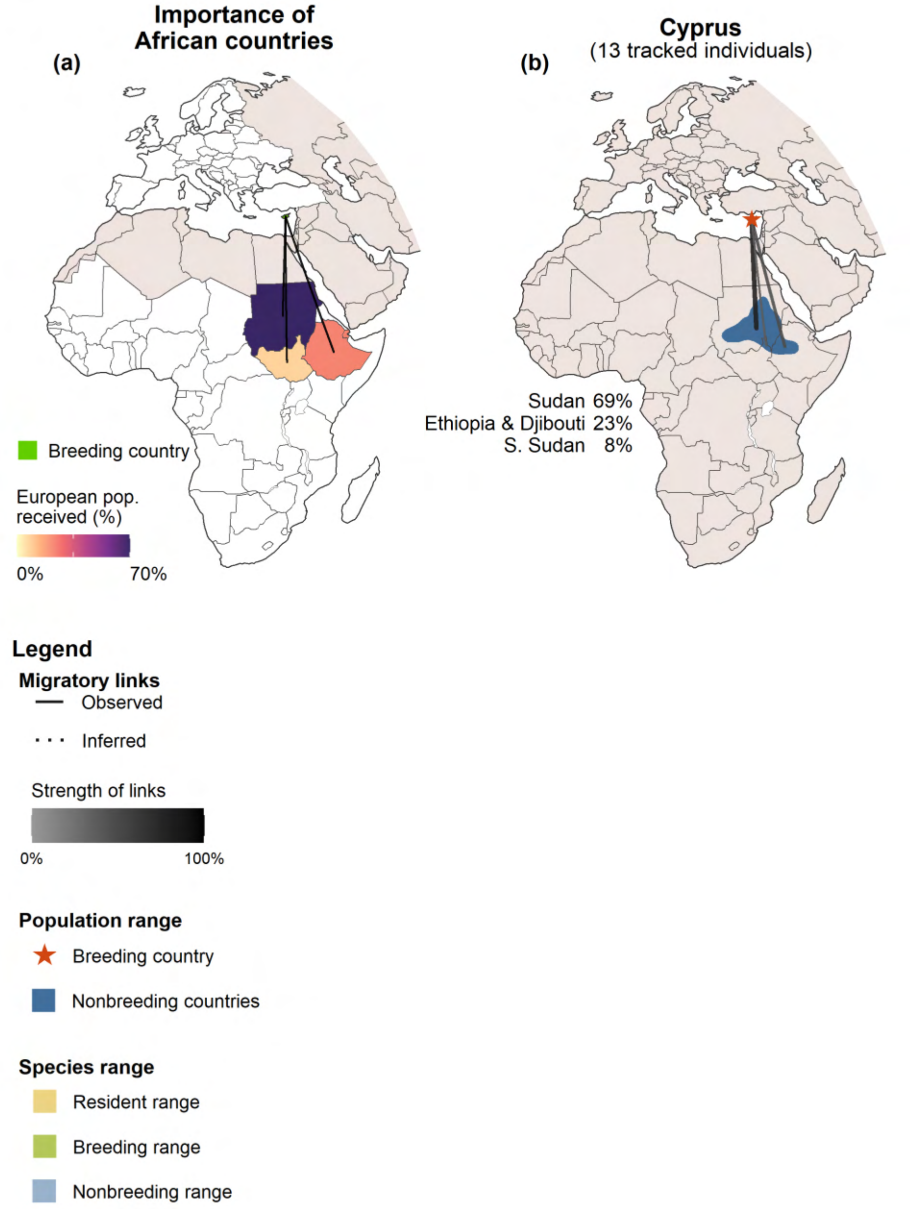

Figure S11.28: Tawny pipit (*Anthus campestris*)

Mapping species-level connectivity for the tawny pipit. (a) Importance of each country in sub-Saharan Africa as nonbreeding grounds for the European population, as revealed by the migration links (observed: solid line, inferred: dotted line) weighted by the percentage of the European population estimated to establish the link; countries in sub-Saharan Africa are colored according to the percentage of the total European population they host during the nonbreeding season. (b - b) Connectivity for each population in our dataset; lines represent the migratory links (observed and inferred), with their respective strength indicated alongside the map (asterisks indicate inferred links).

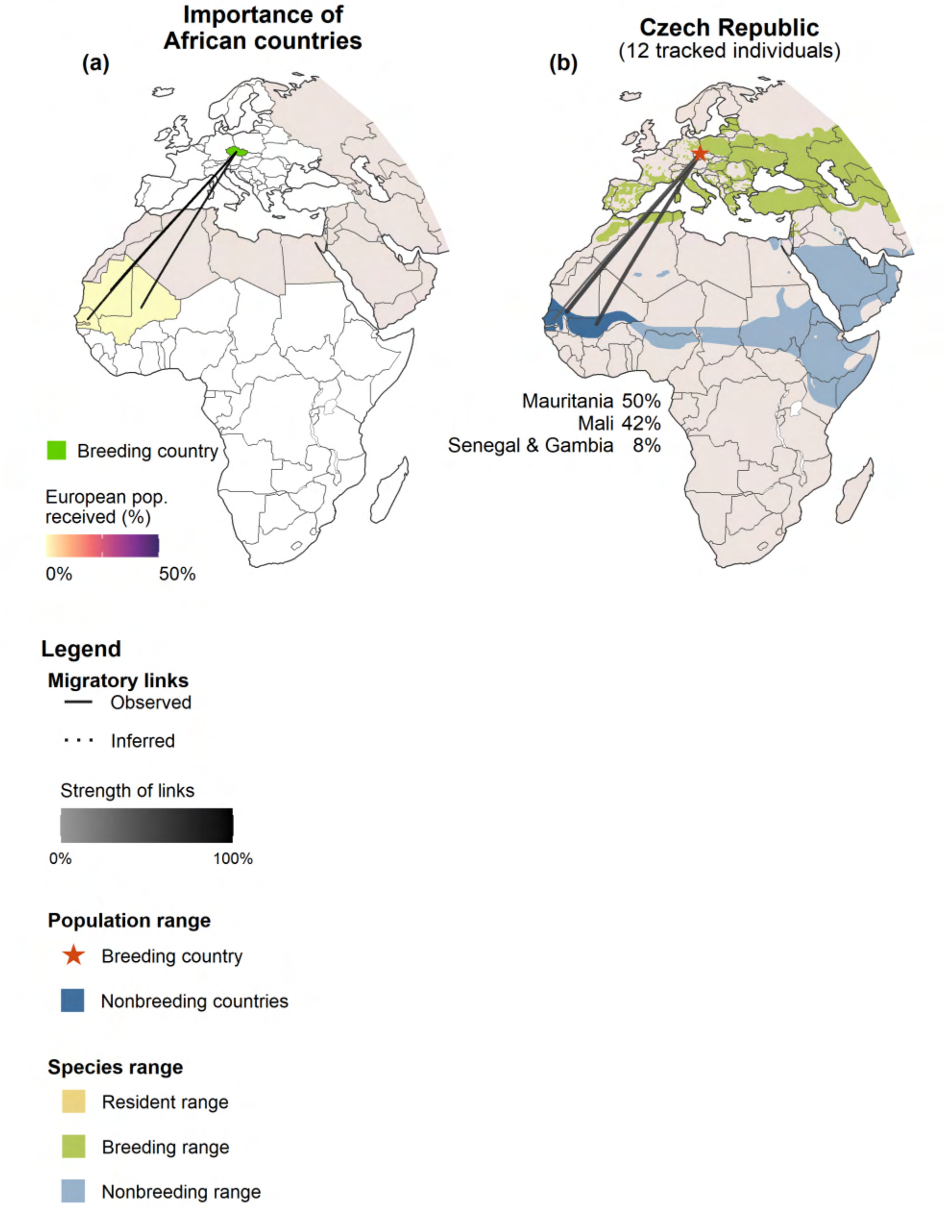

Figure S11.29: Ortolan bunting (*Emberiza hortulana*)

Mapping species-level connectivity for the ortolan bunting. (a) Importance of each country in sub-Saharan Africa as nonbreeding grounds for the European population, as revealed by the migration links (observed: solid line, inferred: dotted line) weighted by the percentage of the European population estimated to establish the link; countries in sub-Saharan Africa are colored according to the percentage of the total European population they host during the nonbreeding season. (b - c) Connectivity for each population in our dataset; lines represent the migratory links (observed and inferred), with their respective strength indicated alongside the map (asterisks indicate inferred links).

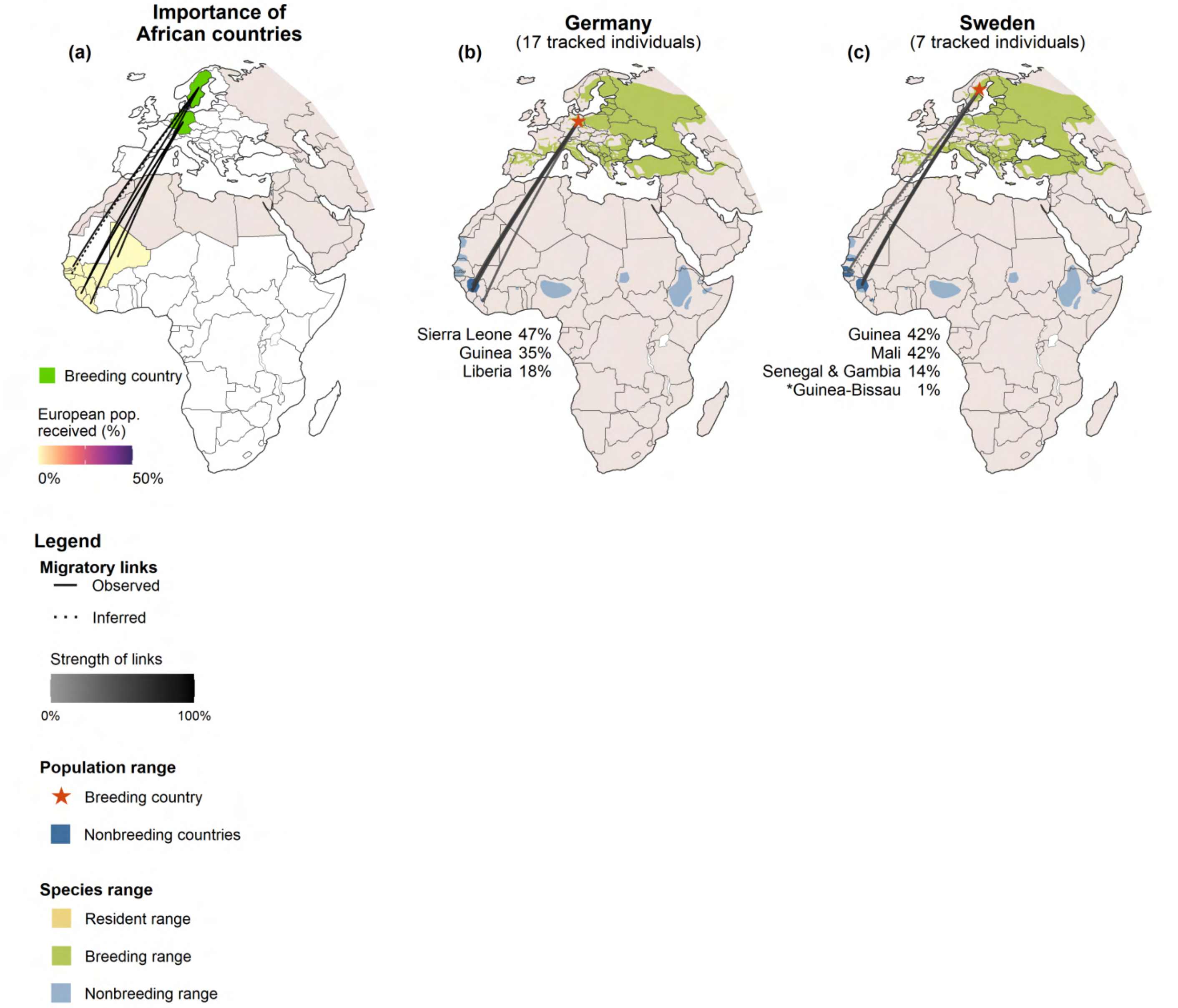

Figure S11.30: Osprey (*Pandion haliaetus*)

Mapping species-level connectivity for the osprey. (a) Importance of each country in sub-Saharan Africa as nonbreeding grounds for the European population, as revealed by the migration links (observed: solid line, inferred: dotted line) weighted by the percentage of the European population estimated to establish the link; countries in sub-Saharan Africa are colored according to the percentage of the total European population they host during the nonbreeding season. (b - g) Connectivity for each population in our dataset; lines represent the migratory links (observed and inferred), with their respective strength indicated alongside the map (asterisks indicate inferred links).

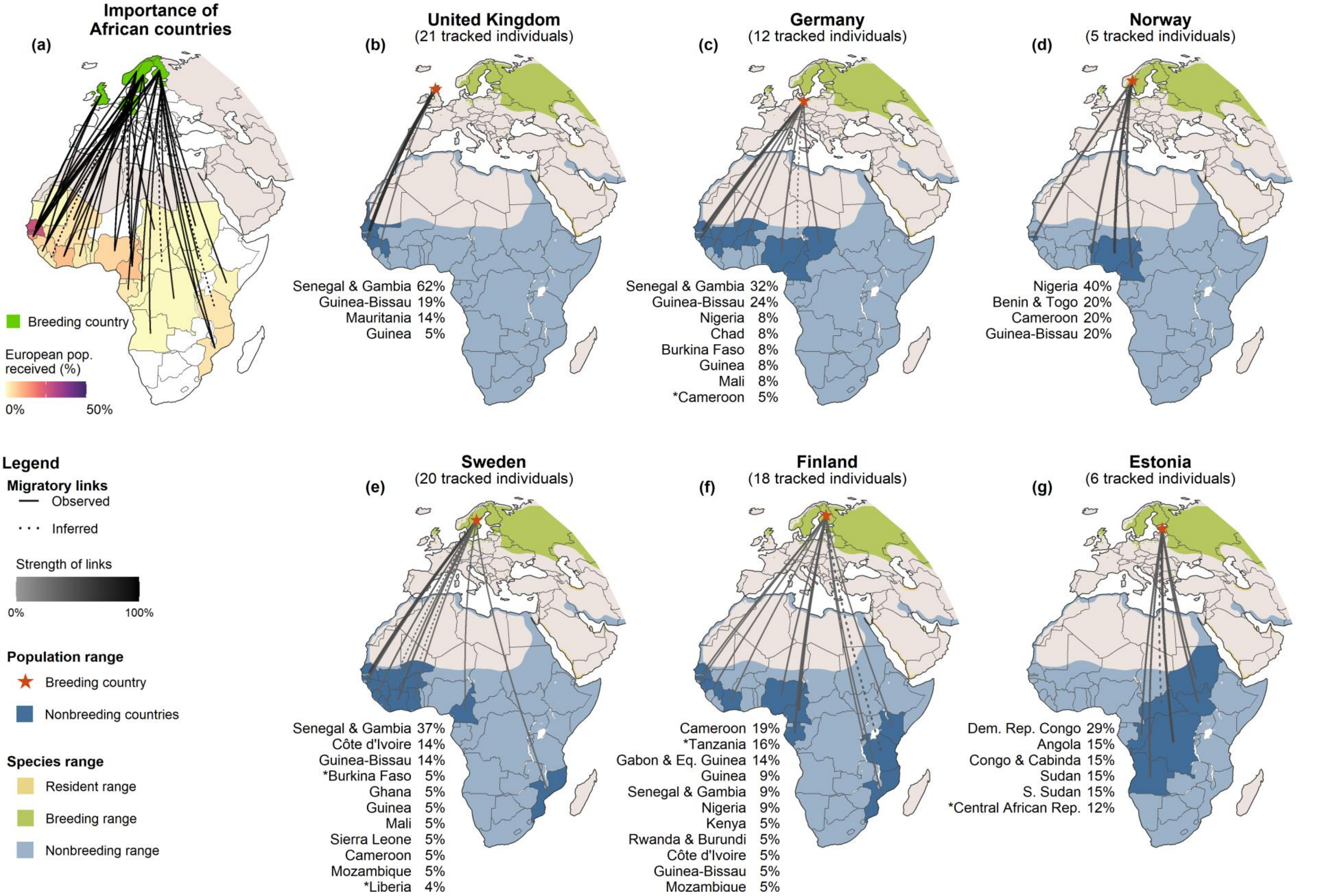

Figure S11.31: European honey-buzzard (*Pernis apivorus*)

Mapping species-level connectivity for the European honey-buzzard. (a) Importance of each country in sub-Saharan Africa as nonbreeding grounds for the European population, as revealed by the migration links (observed: solid line, inferred: dotted line) weighted by the percentage of the European population estimated to establish the link; countries in sub-Saharan Africa are colored according to the percentage of the total European population they host during the nonbreeding season. (b - g) Connectivity for each population in our dataset; lines represent the migratory links (observed and inferred), with their respective strength indicated alongside the map (asterisks indicate inferred links).

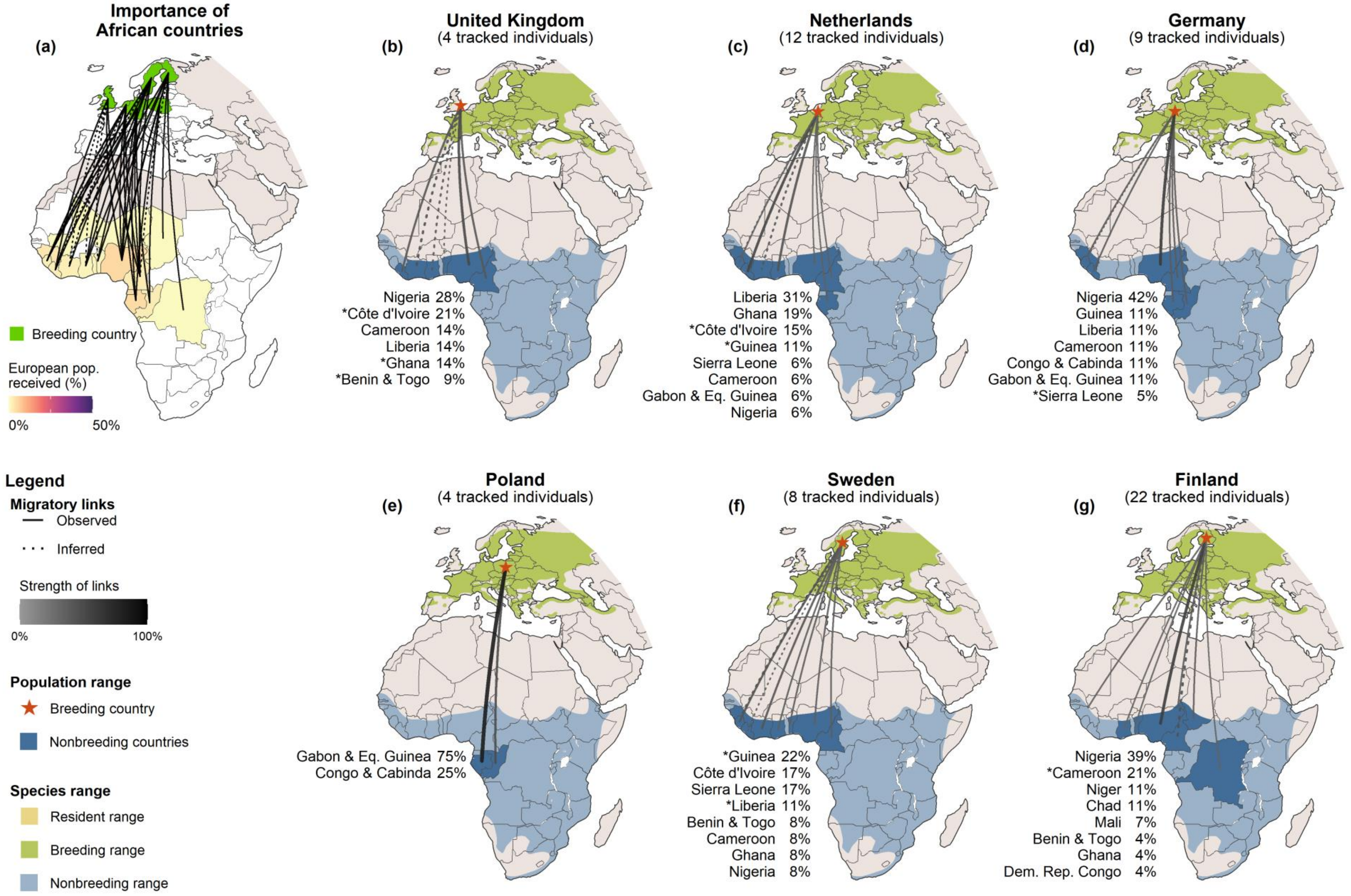

Figure S11.32: Egyptian vulture (*Neophron percnopterus*)

Mapping species-level connectivity for the Egyptian vulture. (a) Importance of each country in sub-Saharan Africa as nonbreeding grounds for the European population, as revealed by the migration links (observed: solid line, inferred: dotted line) weighted by the percentage of the European population estimated to establish the link; countries in sub-Saharan Africa are colored according to the percentage of the total European population they host during the nonbreeding season. (b - f) Connectivity for each population in our dataset; lines represent the migratory links (observed and inferred), with their respective strength indicated alongside the map (asterisks indicate inferred links).

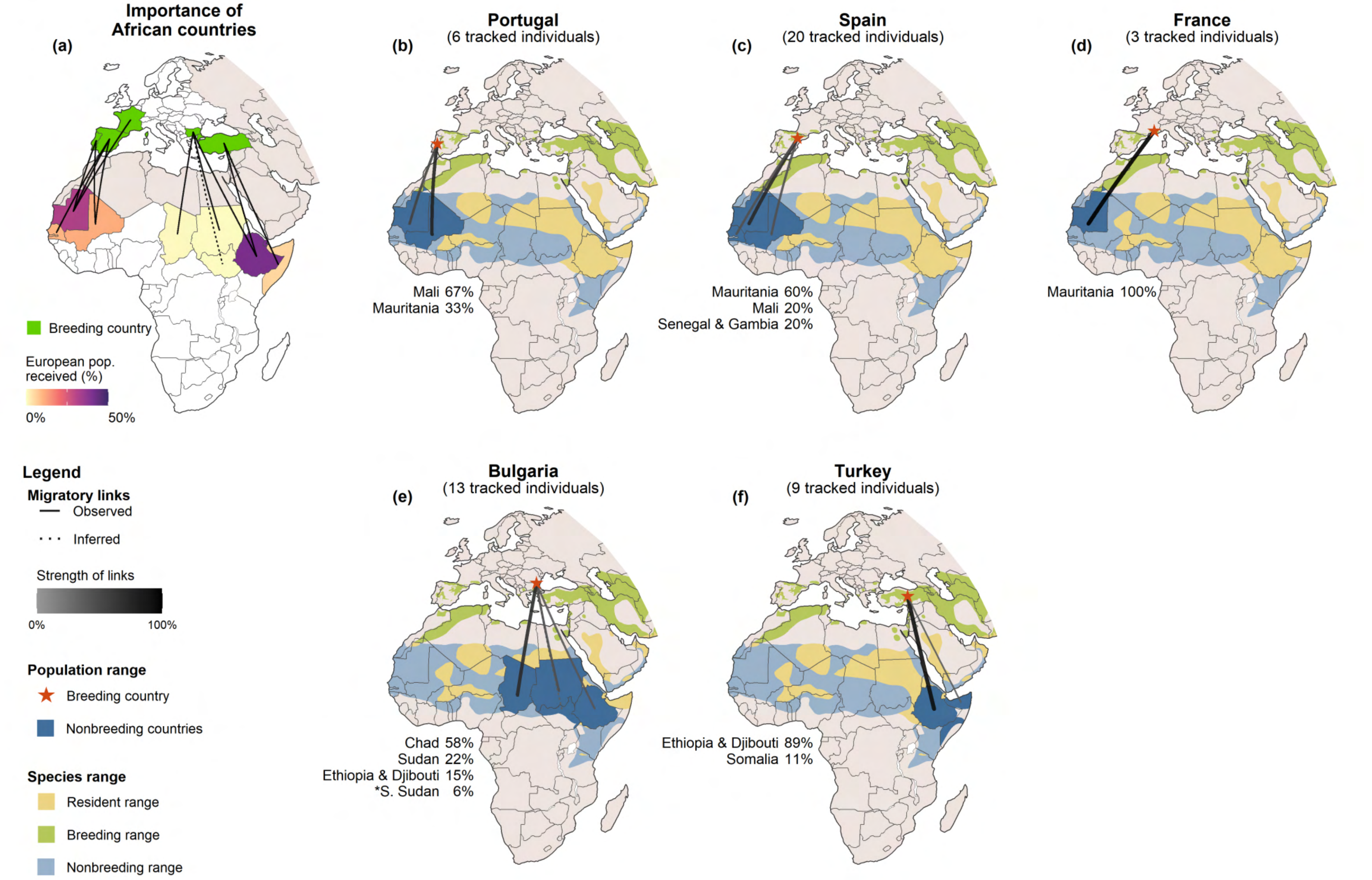

Figure S11.33: Short-toed snake-eagle (*Circaetus gallicus*)

Mapping species-level connectivity for the short-toed snake-eagle. (a) Importance of each country in sub-Saharan Africa as nonbreeding grounds for the European population, as revealed by the migration links (observed: solid line, inferred: dotted line) weighted by the percentage of the European population estimated to establish the link; countries in sub-Saharan Africa are colored according to the percentage of the total European population they host during the nonbreeding season. (b - d) Connectivity for each population in our dataset; lines represent the migratory links (observed and inferred), with their respective strength indicated alongside the map (asterisks indicate inferred links).

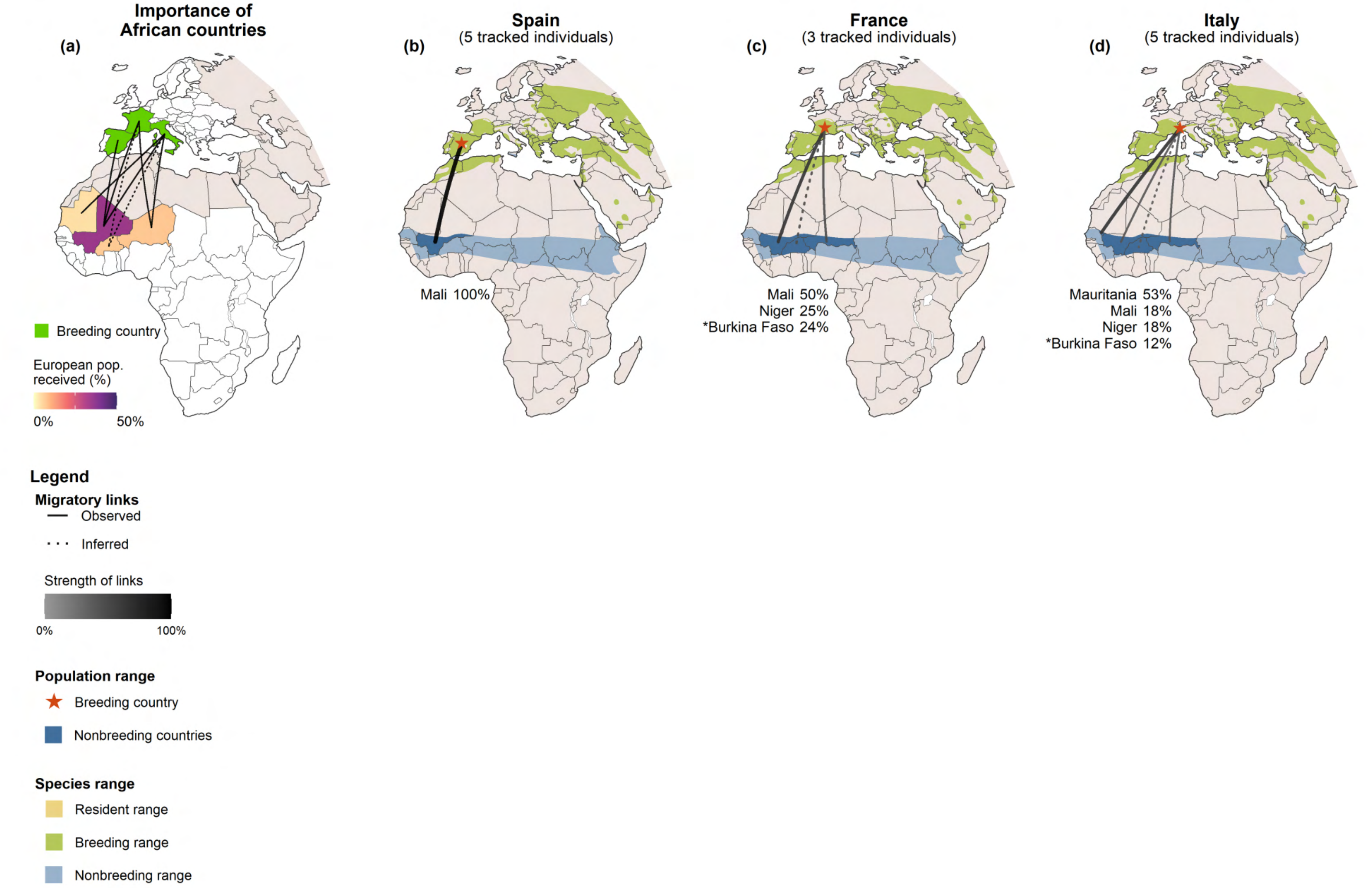

Figure S11.34: Lesser spotted eagle (*Clanga pomarina*)

Mapping species-level connectivity for the lesser spotted eagle. (a) Importance of each country in sub-Saharan Africa as nonbreeding grounds for the European population, as revealed by the migration links (observed: solid line, inferred: dotted line) weighted by the percentage of the European population estimated to establish the link; countries in sub-Saharan Africa are colored according to the percentage of the total European population they host during the nonbreeding season. (b - f) Connectivity for each population in our dataset; lines represent the migratory links (observed and inferred), with their respective strength indicated alongside the map (asterisks indicate inferred links).

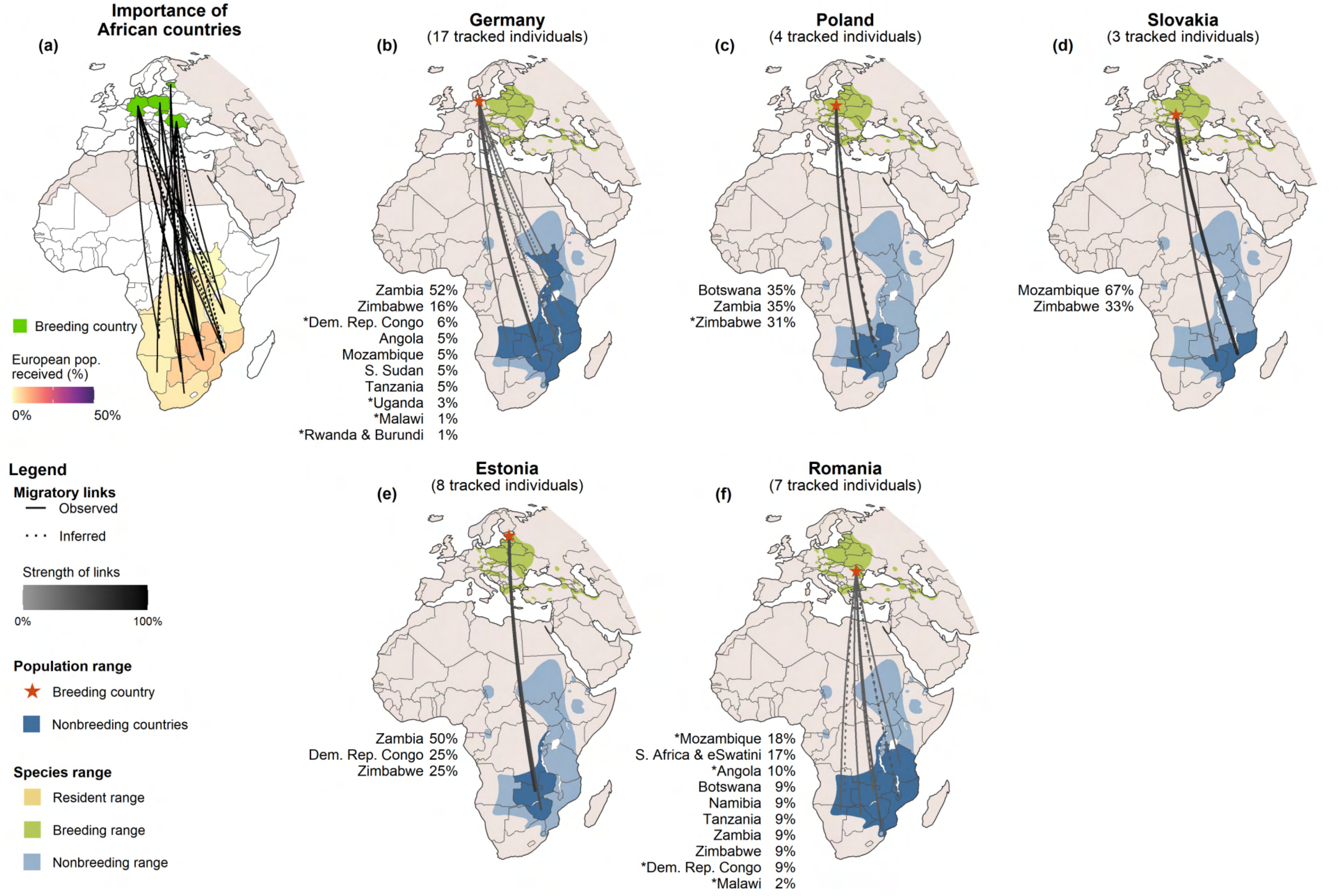

Figure S11.35: Greater spotted eagle (*Clanga clanga*)

Mapping species-level connectivity for the greater spotted eagle. (a) Importance of each country in sub-Saharan Africa as nonbreeding grounds for the European population, as revealed by the migration links (observed: solid line, inferred: dotted line) weighted by the percentage of the European population estimated to establish the link; countries in sub-Saharan Africa are colored according to the percentage of the total European population they host during the nonbreeding season. (b - b) Connectivity for each population in our dataset; lines represent the migratory links (observed and inferred), with their respective strength indicated alongside the map (asterisks indicate inferred links).

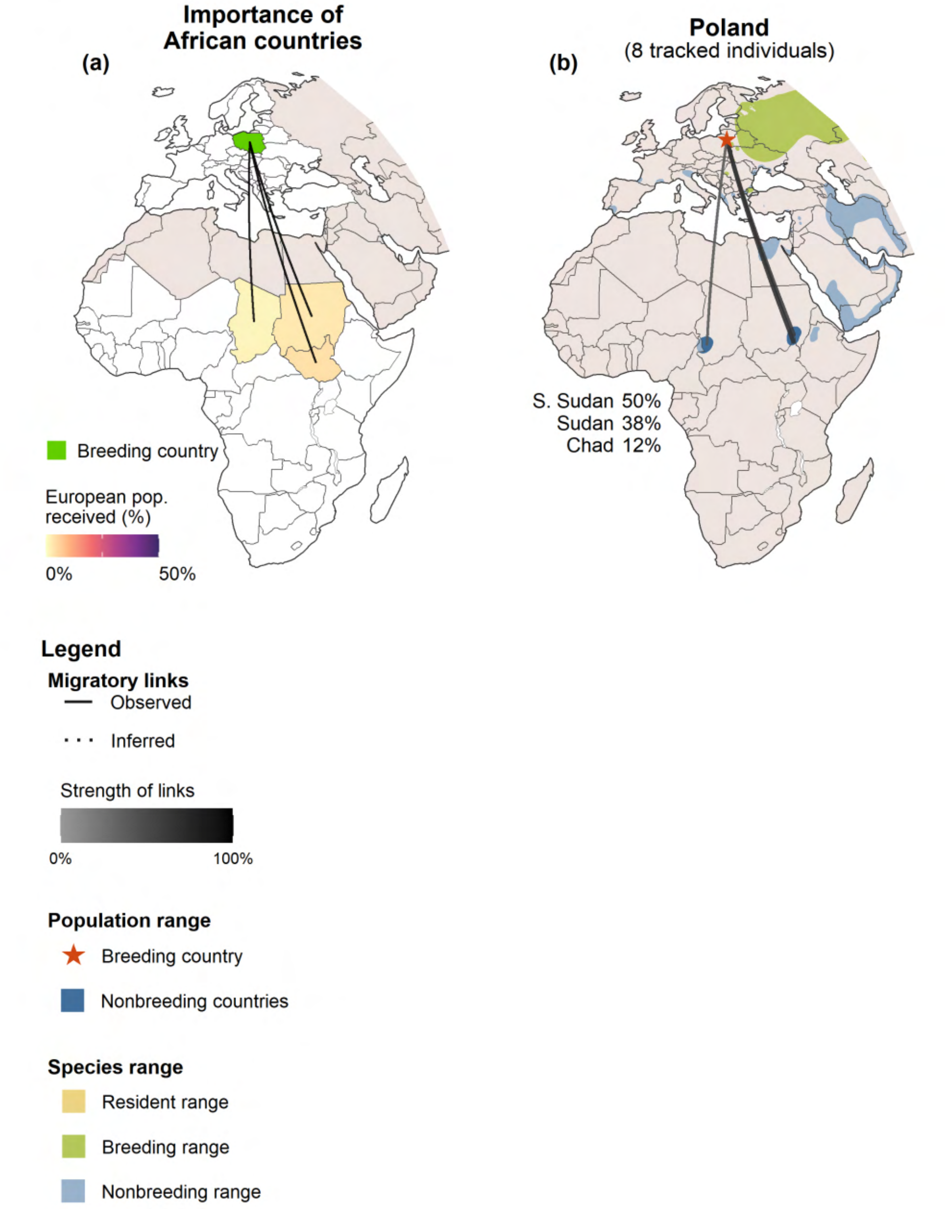

Figure S11.36: Booted eagle (*Hieraaetus pennatus*)

Mapping species-level connectivity for the booted eagle. (a) Importance of each country in sub-Saharan Africa as nonbreeding grounds for the European population, as revealed by the migration links (observed: solid line, inferred: dotted line) weighted by the percentage of the European population estimated to establish the link; countries in sub-Saharan Africa are colored according to the percentage of the total European population they host during the nonbreeding season. (b - b) Connectivity for each population in our dataset; lines represent the migratory links (observed and inferred), with their respective strength indicated alongside the map (asterisks indicate inferred links).

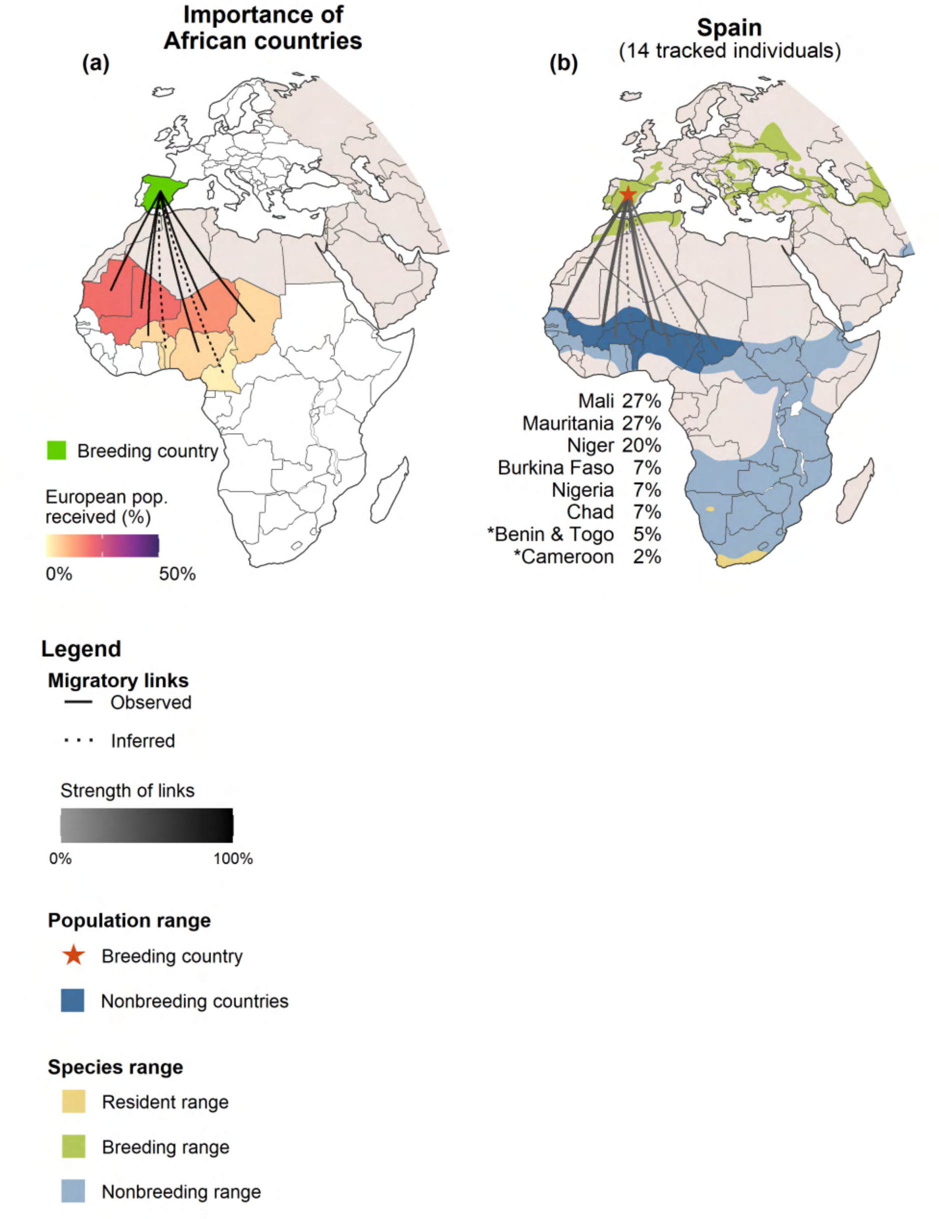

Figure S11.37: Western marsh-harrier (*Circus aeruginosus*)

Mapping species-level connectivity for the Western marsh-harrier. (a) Importance of each country in sub-Saharan Africa as nonbreeding grounds for the European population, as revealed by the migration links (observed: solid line, inferred: dotted line) weighted by the percentage of the European population estimated to establish the link; countries in sub-Saharan Africa are colored according to the percentage of the total European population they host during the nonbreeding season. (b - h) Connectivity for each population in our dataset; lines represent the migratory links (observed and inferred), with their respective strength indicated alongside the map (asterisks indicate inferred links).

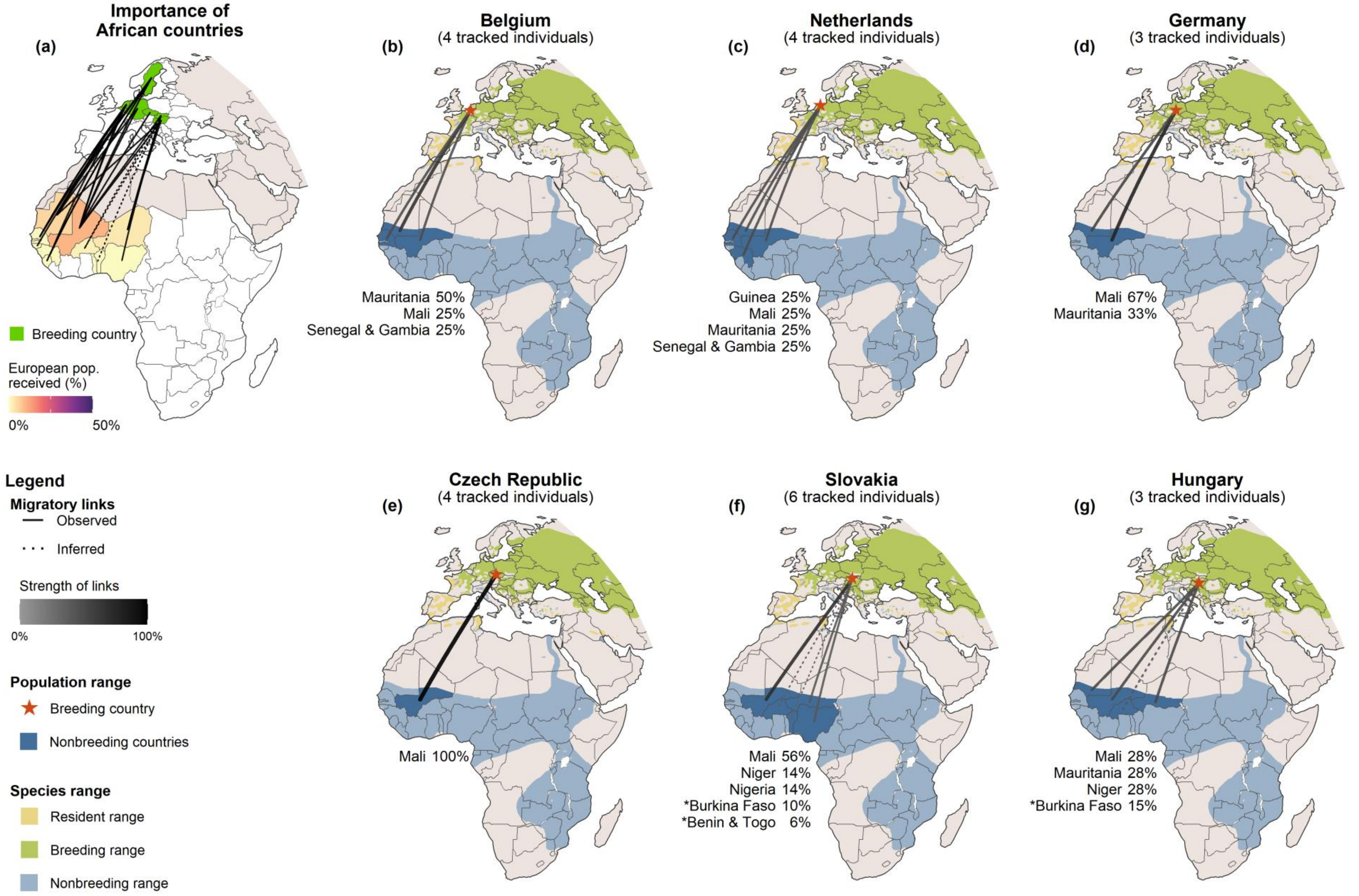

Western Marsh-harrier (continued)

Mapping species-level connectivity for the Western marsh-harrier. (a) Importance of each country in sub-Saharan Africa as nonbreeding grounds for the European population, as revealed by the migration links (observed: solid line, inferred: dotted line) weighted by the percentage of the European population estimated to establish the link; countries in sub-Saharan Africa are colored according to the percentage of the total European population they host during the nonbreeding season. (b - h) Connectivity for each population in our dataset; lines represent the migratory links (observed and inferred), with their respective strength indicated alongside the map (asterisks indicate inferred links).

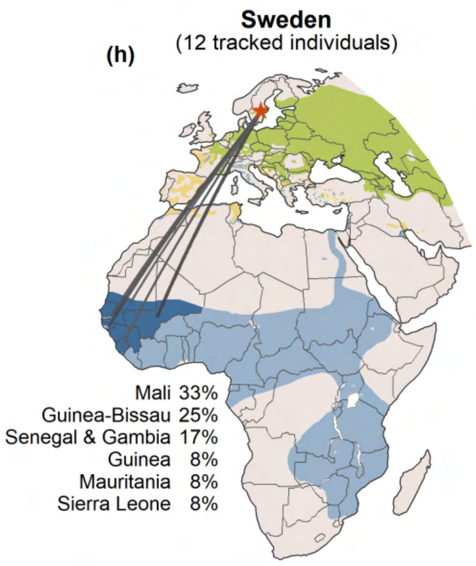

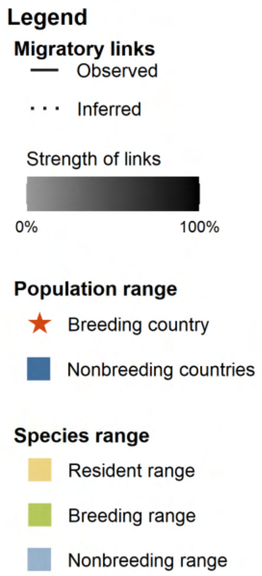

Figure S11.38: Montagu's harrier (*Circus pygargus*)

Mapping species-level connectivity for the Montagu's harrier. (a) Importance of each country in sub-Saharan Africa as nonbreeding grounds for the European population, as revealed by the migration links (observed: solid line, inferred: dotted line) weighted by the percentage of the European population estimated to establish the link; countries in sub-Saharan Africa are colored according to the percentage of the total European population they host during the nonbreeding season. (b - h) Connectivity for each population in our dataset; lines represent the migratory links (observed and inferred), with their respective strength indicated alongside the map (asterisks indicate inferred links).

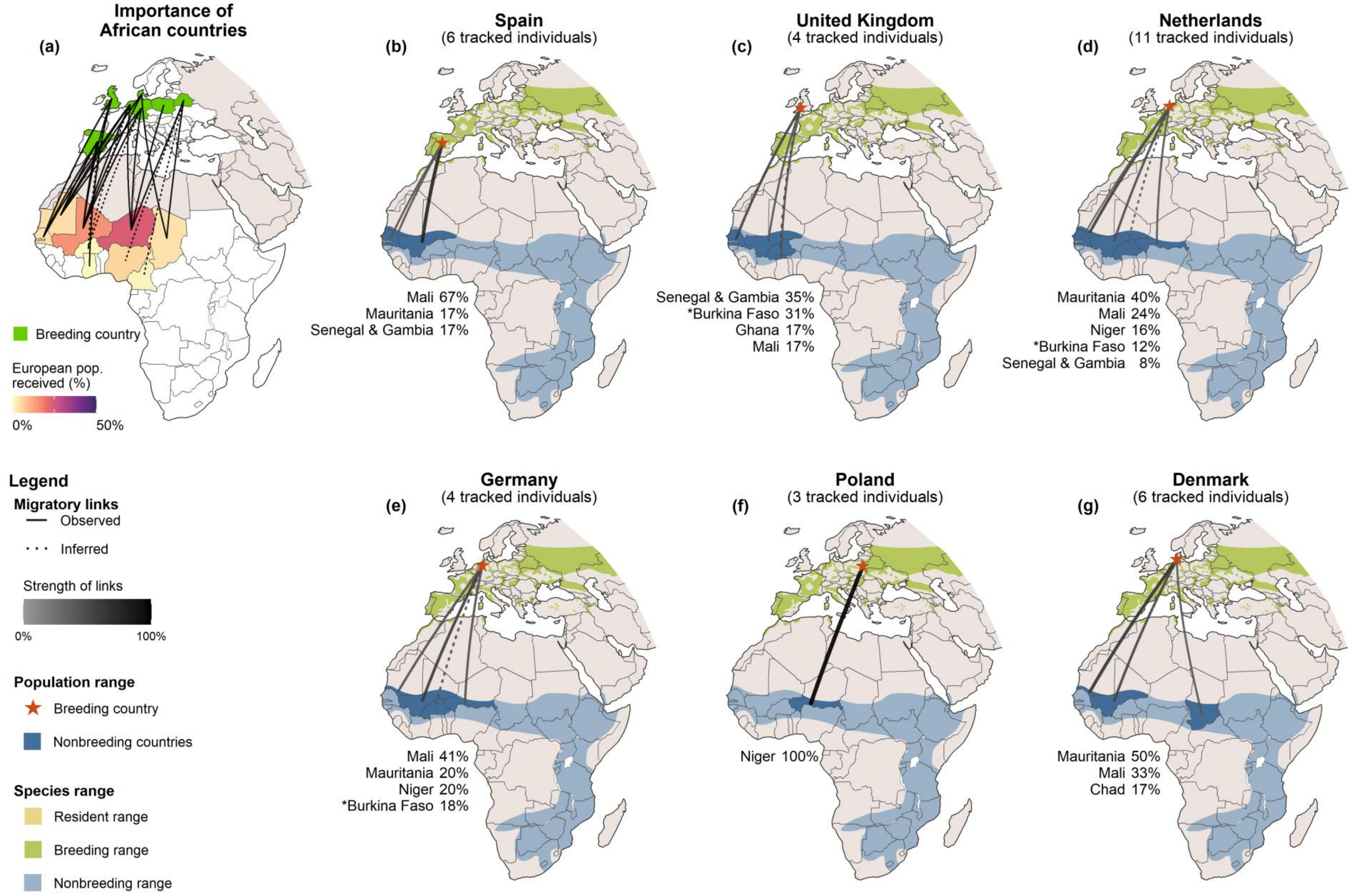

Montagu’s harrier (continued)

Mapping species-level connectivity for the Montagu’s harrier. (a) Importance of each country in sub-Saharan Africa as nonbreeding grounds for the European population, as revealed by the migration links (observed: solid line, inferred: dotted line) weighted by the percentage of the European population estimated to establish the link; countries in sub-Saharan Africa are colored according to the percentage of the total European population they host during the nonbreeding season. (b - h) Connectivity for each population in our dataset; lines represent the migratory links (observed and inferred), with their respective strength indicated alongside the map (asterisks indicate inferred links).

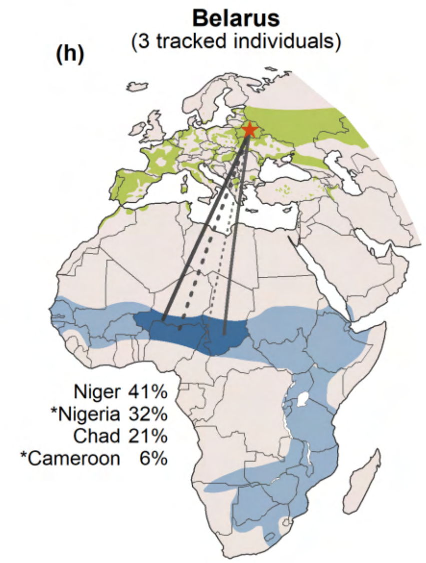

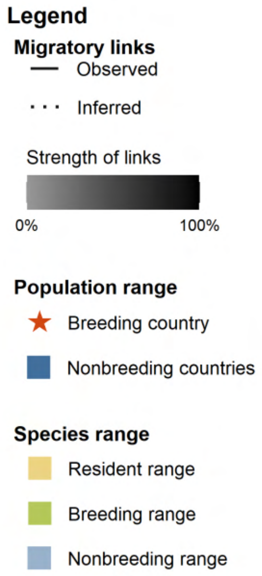

Figure S11.39: Black kite (*Milvus migrans*)

Mapping species-level connectivity for the black kite. (a) Importance of each country in sub-Saharan Africa as nonbreeding grounds for the European population, as revealed by the migration links (observed: solid line, inferred: dotted line) weighted by the percentage of the European population estimated to establish the link; countries in sub-Saharan Africa are colored according to the percentage of the total European population they host during the nonbreeding season. (b - d) Connectivity for each population in our dataset; lines represent the migratory links (observed and inferred), with their respective strength indicated alongside the map (asterisks indicate inferred links).

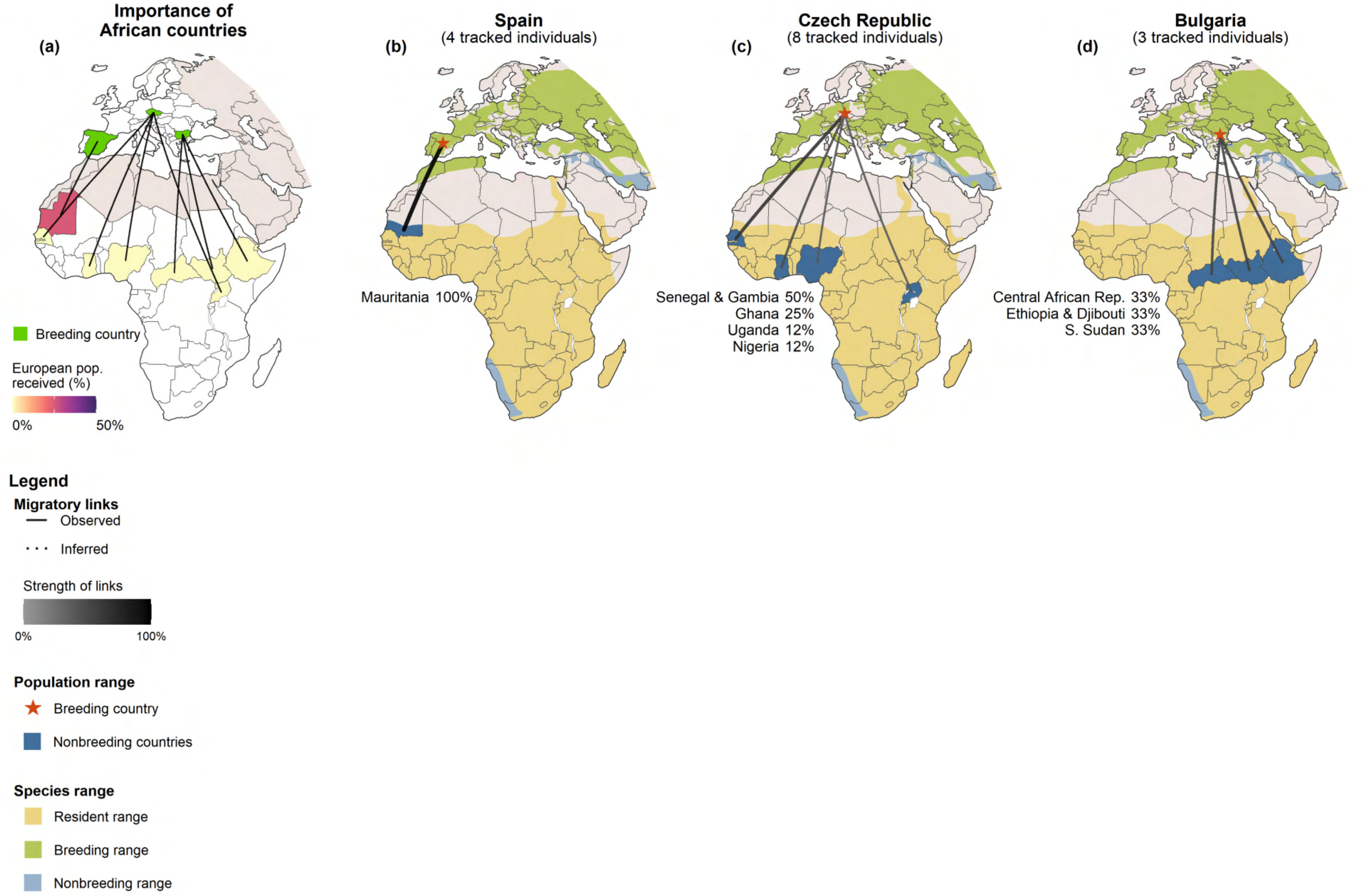

Figure S11.40: Lesser kestrel (*Falco naumanni*)

Mapping species-level connectivity for the lesser kestrel. (a) Importance of each country in sub-Saharan Africa as nonbreeding grounds for the European population, as revealed by the migration links (observed: solid line, inferred: dotted line) weighted by the percentage of the European population estimated to establish the link; countries in sub-Saharan Africa are colored according to the percentage of the total European population they host during the nonbreeding season. (b - g) Connectivity for each population in our dataset; lines represent the migratory links (observed and inferred), with their respective strength indicated alongside the map (asterisks indicate inferred links).

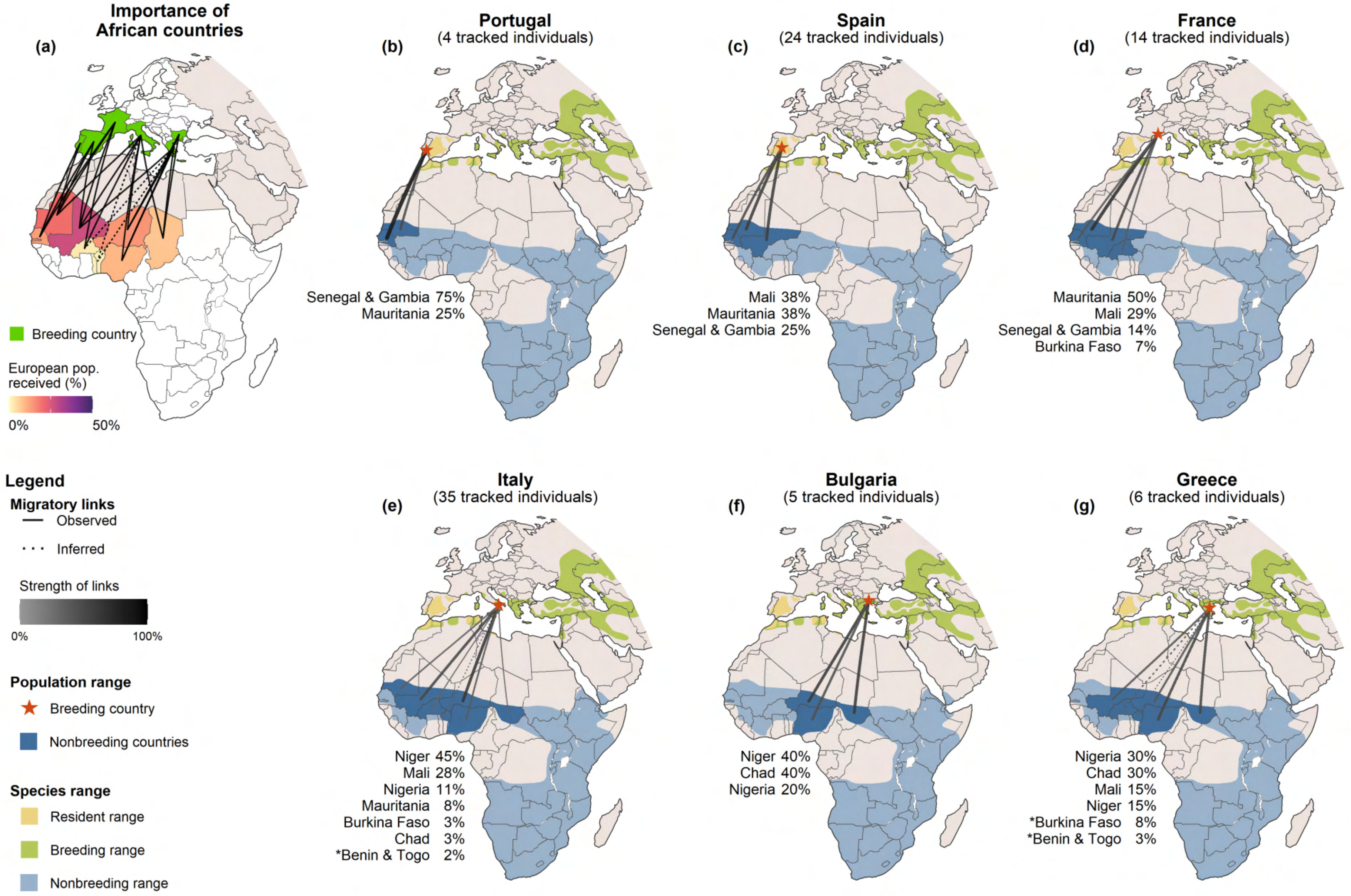

Figure S11.41: Red-footed falcon (*Falco vespertinus*)

Mapping species-level connectivity for the red-footed falcon. (a) Importance of each country in sub-Saharan Africa as nonbreeding grounds for the European population, as revealed by the migration links (observed: solid line, inferred: dotted line) weighted by the percentage of the European population estimated to establish the link; countries in sub-Saharan Africa are colored according to the percentage of the total European population they host during the nonbreeding season. (b - b) Connectivity for each population in our dataset; lines represent the migratory links (observed and inferred), with their respective strength indicated alongside the map (asterisks indicate inferred links).

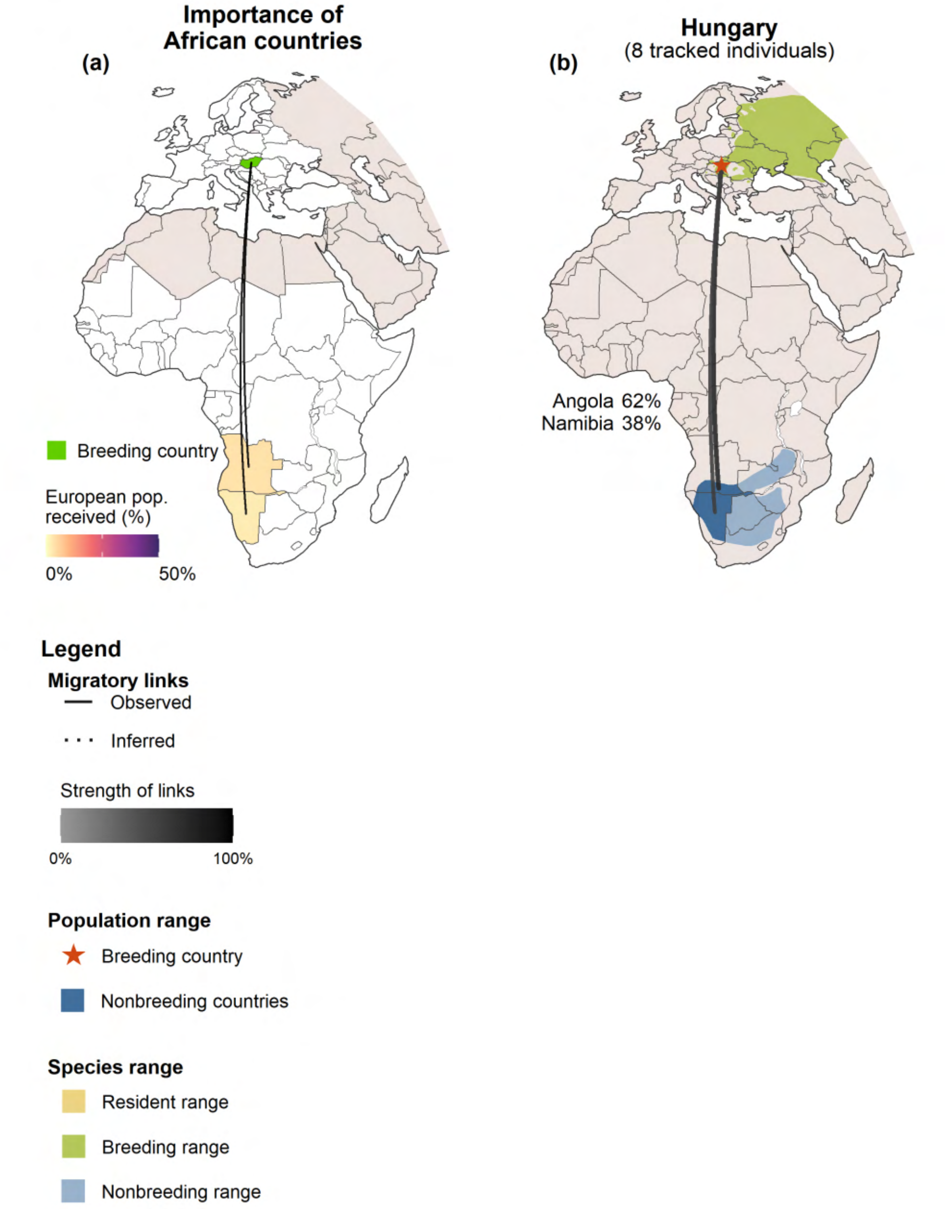

Figure S11.42: Eleonora's falcon (*Falco eleonorae*)

Mapping species-level connectivity for the Eleonora's falcon. (a) Importance of each country in sub-Saharan Africa as nonbreeding grounds for the European population, as revealed by the migration links (observed: solid line, inferred: dotted line) weighted by the percentage of the European population estimated to establish the link; countries in sub-Saharan Africa are colored according to the percentage of the total European population they host during the nonbreeding season. (b - e) Connectivity for each population in our dataset; lines represent the migratory links (observed and inferred), with their respective strength indicated alongside the map (asterisks indicate inferred links).

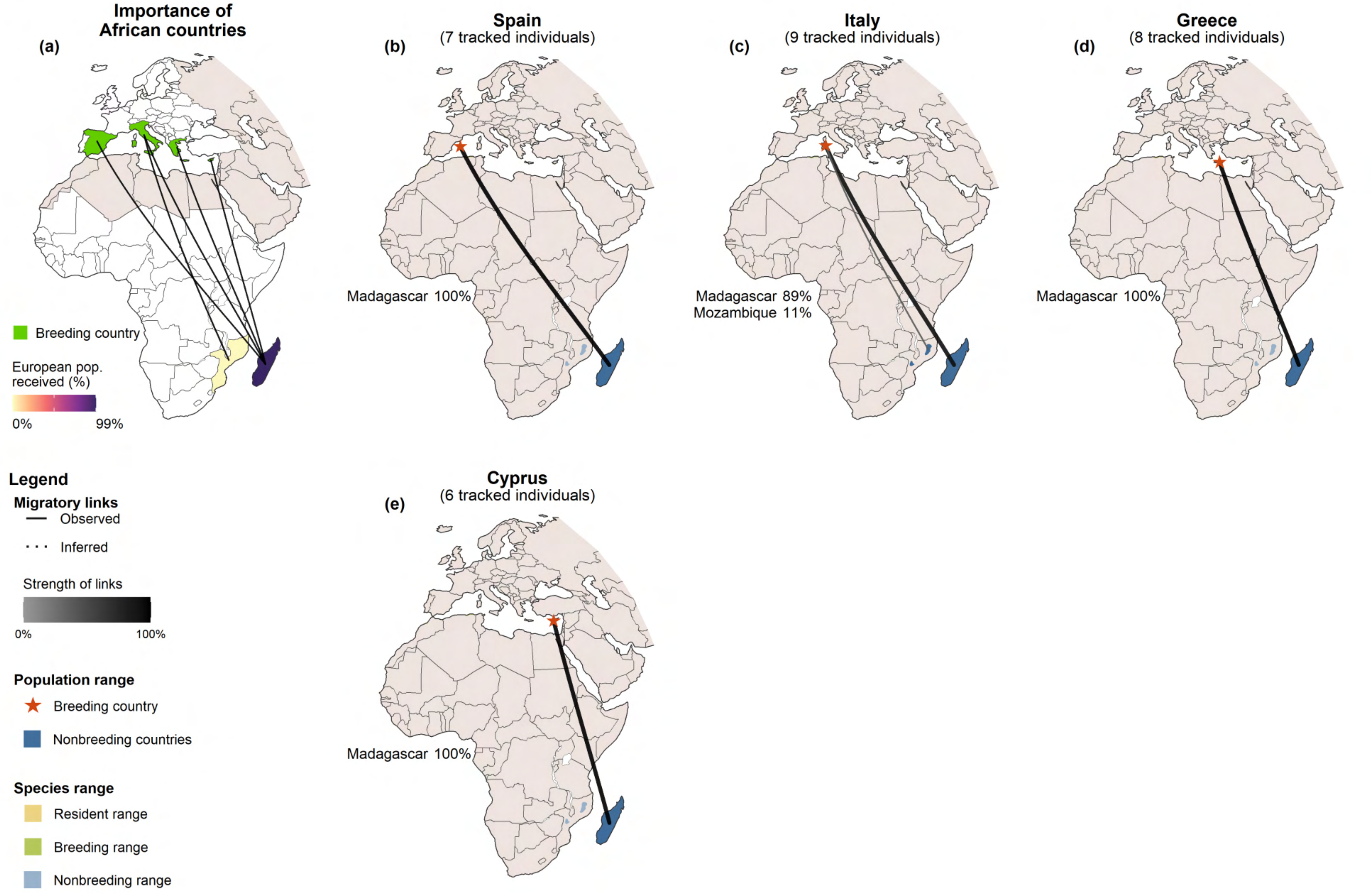

Figure S11.43: Eurasian hobby (*Falco subbuteo*)

Mapping species-level connectivity for the Eurasian hobby. (a) Importance of each country in sub-Saharan Africa as nonbreeding grounds for the European population, as revealed by the migration links (observed: solid line, inferred: dotted line) weighted by the percentage of the European population estimated to establish the link; countries in sub-Saharan Africa are colored according to the percentage of the total European population they host during the nonbreeding season. (b - b) Connectivity for each population in our dataset; lines represent the migratory links (observed and inferred), with their respective strength indicated alongside the map (asterisks indicate inferred links).

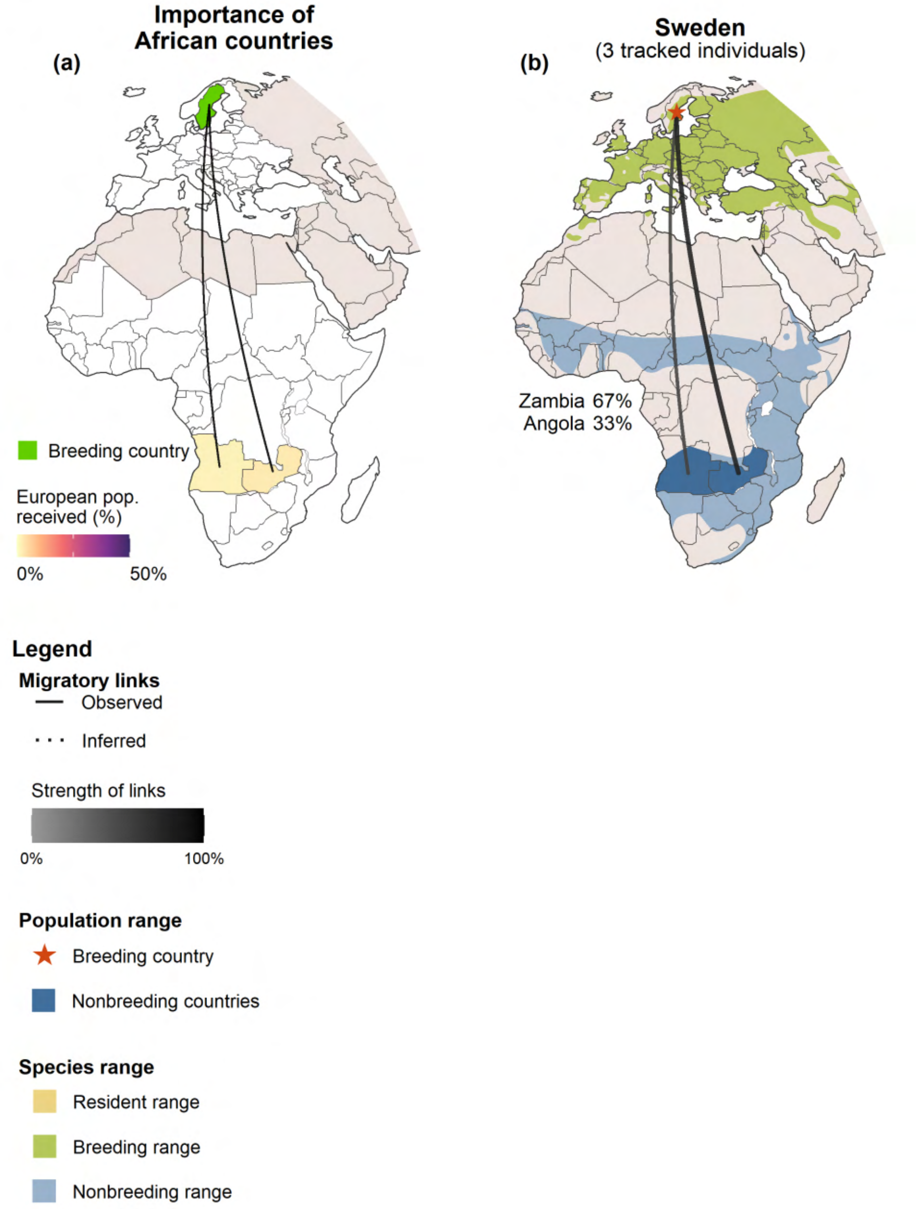

Supplement: Supplementary file 5 — Appendix S11. Mapping species‐level connectivity for species of long‐distance migratory landbirds and raptors [file COBI-37-0-s002.pdf]
